# Comparative Gene Expression Profiling of Primary and Metastatic Renal Cell Carcinoma Stem Cell-Like Cancer Cells

**DOI:** 10.1371/journal.pone.0165718

**Published:** 2016-11-03

**Authors:** Mohammed I. Khan, Anna M. Czarnecka, Sławomir Lewicki, Igor Helbrecht, Klaudia Brodaczewska, Irena Koch, Robert Zdanowski, Magdalena Król, Cezary Szczylik

**Affiliations:** 1 Molecular Oncology Laboratory, Department of Oncology, Military Institute of Medicine, Warsaw, Poland; 2 Department of Regenerative Medicine, Military Institute of Hygiene and Epidemiology, Warsaw, Poland; 3 Department of Physiological Sciences, Faculty of Veterinary Medicine, Warsaw University of Life Sciences—WULS, Warsaw, Poland; 4 Institute of Genetics and Biotechnology, Faculty of Biology, Warsaw University, Warsaw, Poland; 5 Department of Pathomorphology, Institute of Mother and Child, Warsaw, Poland; University of Navarra, SPAIN

## Abstract

**Background:**

Recent advancement in cancer research has shown that tumors are highly heterogeneous, and multiple phenotypically different cell populations are found in a single tumor. Cancer development and tumor growth are driven by specific types of cells—stem cell-like cancer cells (SCLCCs)—which are also responsible for metastatic spread and drug resistance. This research was designed to verify the presence of SCLCCs in renal cell cancer cell lines. Subsequently, we aimed to characterize phenotype and cell biology of CD105+ cells, defined previously as renal cell carcinoma tumor-initiating cells. The main goal of the project was to describe the gene-expression profile of stem cell-like cancer cells of primary tumor and metastatic origin.

**Materials and Methods:**

Real-time PCR analysis of stemness genes (Oct-4, Nanog and Ncam) and soft agar colony formation assay were conducted to check the stemness properties of renal cell carcinoma (RCC) cell lines. FACS analysis of CD105+ and CD133+ cells was performed on RCC cells. Isolated CD105+ cells were verified for expression of mesenchymal markers—CD24, CD146, CD90, CD73, CD44, CD11b, CD19, CD34, CD45, HLA-DR and alkaline phosphatase. Hanging drop assay was used to investigate CD105+ cell-cell cohesion. Analysis of free-floating 3D spheres formed by isolated CD105+ was verified, as spheres have been hypothesized to contain undifferentiated multipotent progenitor cells. Finally, CD105+ cells were sorted from primary (Caki-2) and metastatic (ACHN) renal cell cancer cell lines. Gene-expression profiling of sorted CD105+ cells was performed with Agilent’s human GE 4x44K v2 microarrays. Differentially expressed genes were further categorized into canonical pathways. Network analysis and downstream analysis were performed with Ingenuity Pathway Analysis.

**Results:**

Metastatic RCC cell lines (ACHN and Caki-1) demonstrated higher colony-forming ability in comparison to primary RCC cell lines. Metastatic RCC cell lines harbor numerous CD105+ cell subpopulations and have higher expression of stemness genes (Oct-4 and Nanog). CD105+ cells adopt 3D grape-like floating structures under handing drop conditions. Sorted CD105+ cells are positive for human mesenchymal stem cell (MSC) markers CD90, CD73, CD44, CD146, and alkaline phosphatase activity, but not for CD24 and hematopoietic lineage markers CD34, CD11b, CD19, CD45, and HLA-DR. 1411 genes are commonly differentially expressed in CD105+ cells (both from primary [Caki-2] and metastatic RCC [ACHN] cells) in comparison to a healthy kidney epithelial cell line (ASE-5063). TGF-β, Wnt/β-catenine, epithelial-mesenchymal transition (EMT), Rap1 signaling, PI3K-Akt signaling, and Hippo signaling pathway are deregulated in CD105+ cells. TGFB1, ERBB2, and TNF are the most significant transcriptional regulators activated in these cells.

**Conclusions:**

All together, RCC-CD105+ cells present stemlike properties. These stem cell-like cancer cells may represent a novel target for therapy. A unique gene-expression profile of CD105+ cells could be used as initial data for subsequent functional studies and drug design.

## Introduction

Renal cell carcinoma (RCC) is the most common type of kidney cancer and accounts for 3% of all cancer cases worldwide. The incidence of RCC has been steadily rising over the last 30 years [1]. The prognosis for patients with RCC is poor; it is believed that approximately 30%–40% of primary localized RCC patients will develop metastatic disease if it is not detected early [2]. Late detection and rapid metastasis of RCC spread has a negative impact on a patient’s survival. Metastatic RCC is resistant to conventional therapies, including chemotherapy and radiotherapy. Over the past ten years, targeted therapies have been developed and have shown a significant objective response rate, long progression-free survival (PFS), and overall survival (OS) in phase III clinical trials [3–5]. Resistance may have developed in the course of treatment [6]. At the same time, treatment may result in development of diverse adverse effects [7]. It was recently hypothesized that drug resistance, disease progression, and recurrence are mediated by stem cell-like cancer cells (SCLCCs) also referred to as cancer stem cells/tumor-initiating cells (CSCs/TICs) [8, 9]. This remains in accordance with recent progress in cancer research that has shown tumors as heterogeneous with multiple cell populations and developed as an offspring of SCLCCs [10–12]. Populations of SCLCCs also display a significant phenotypic plasticity and may arise in the process of and/or undergo EMT, which in turn favors metastatic spread and a drug-resistant phenotype [13–16].

In RCC, several techniques for detection and enumeration of SCLCCs have been developed in recent years [17]. The most widely used SCLCCs-isolation approach adapts membrane marker-based methods, including FACS or affinity column isolation. Multiple RCC SCLCCs-specific membrane markers have been suggested in the past, including CD105, CD133, CXCR4, and CD44 [17]. The presence of SCLCCs (mainly CD105 and CD133) has never studied extensively in established RCC cell lines except for few research [18–22], which are widely used in other RCC studies, including its cell biology or drug resistance. Therefore, we selected CD105 and CD133 markers to further investigate the potential presence of SCLCCs in RCC cell lines.

CD133, also known as promin-1, is a novel 5-transmembrane cell-surface antigen encoded by the PROM-1 gene [23]. Many studies have confirmed CD133 as a SCLCCs surface marker in various cancers [24, 25]. However, whether CD133 can be a used as a SCLCCs marker in RCC cell lines is still unclear. Bruno et al. demonstrated that undifferentiated CD133+ progenitor cells were unable to form tumors in a xenograft study; however, these cells promoted formation of new blood vessels and vascularization of tumors in mice [22]. Therefore, our aim was to explore and analyze CD133+ cells in RCC cells.

**Fig 1:**
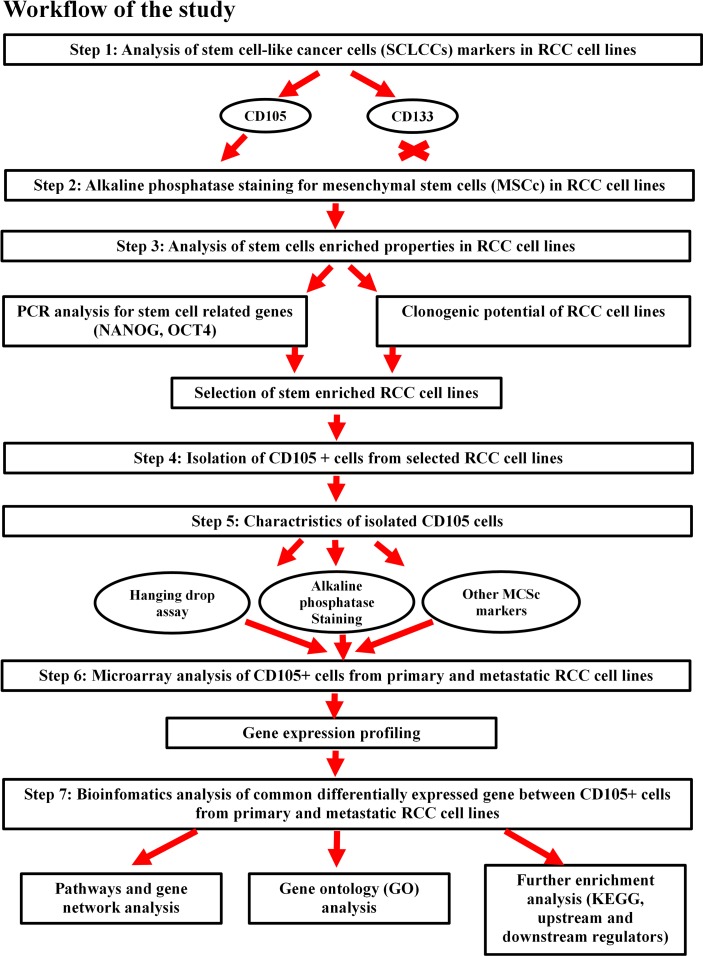
Workflow of the analysis design carried out in this study.

The second membrane marker chosen for SCLCCs identification is an MSC marker, CD105. CD105 (endoglin) is a member of the transforming growth factor (TGF-β1 and β3) receptor complex and modulates TGF-β signaling by influencing receptor cellular localization and cellular migration [26]. CD105 promotes angiogenesis, regulates cytoskeleton organization, and affects cell morphology and migration [27–29]. CD105+ cells may also promote tumor metastasis by circulating tumor cells [16, 30]. Bussolati et al. isolated CD105+ cells and described them as tumor initiating in mice models based on cell isolation in patients undergoing radical nephrectomy [31]. The potential identification of CD105+ and CD133+ cells as SCLCCs in established RCC cell lines, and data based on their gene-expression profiling, could be used in further research for targeting and eliminating cancer cells. **Fig 1** shows the workflow of research carried out in this study.

## Materials and Methods

### Renal cell carcinoma cell lines

Human primary RCC cell lines 786-O, SMKT-R2, SMKT-R3, Caki-2, RCC-6, and 769-P, and metastatic cell lines Caki-1 and ACHN, were used for cell culture and experiments. RCC-6 cells were gifted from Prof. Salem Chouaib (INSERM, Institut Gustave Roussy, Villejuif, France). SMKT-R2 and SMKT-R3 cells were gifted from Prof. T. Tsukamoto and Dr. S. Tochizawa (School of Medicine, Sapporo Medical University, Sapporo, Japan). 786-O, Caki-1, Caki-2, ACHN, and 769-P were bought from ATCC (Virginia, USA), and human healthy kidney epithelial cell line (ASE-5063) was bought from Applied StemCell Inc. (California, USA). All cell lines were maintained in RPMI-1640 + GlutaMAX™-I media (LifeTechnologies, California, USA) with 10% fetal bovine serum (FBS) (Biochrom GmbH, Cambridge, UK) and 1% PenStrep (100 U/mL penicillin and 100μg/mL streptomycin) (AMRESCO LLC, Ohio, USA). Cell lines were expanded in T-75 flasks, and T25 and 96, 24, and 6-well plates (Orange Scientific, Braine-l'Alleud, Belgium), depending on the experiments. Confluent cell monolayers were harvested using Accutase^TM^ Cell Detachment Solution (BD Biosciences, California, USA) for 5–15 min at room temperature and re-suspended in RPMI-1640 + GlutaMAX™-I for all experiments.

### Immunocytochemistry (ICC) staining

Cells were cultured in T25 flasks, as described above. Approximately 80% confluence was collected with Accutase and suspended in phosphate buffer solution (PBS). Cells were then centrifuged and re-suspended in 4% paraformaldehyde (PFA) for 10 min at room temperature. PFA was removed by centrifugation, and cells were placed in ddH2O onto ICC SuperFrost^TM^ microscope slides (ThermoFisher Scientific, Massachusetts, USA) and allowed to dry. The staining was performed in a Flex Autostainer instrument (Dako, Glostrup, Denmark) and with the use of EnVisionFlex kits (Dako, Glostrup, Denmark). ICC was performed using the following primary antibodies: rabbit anti-CD24 polyclonal (dilution 1:200, Bioss, Massachusetts, USA), monoclonal mouse anti-human CD44 (dilution 1:50, Dako, Glostrup, Denmark), monoclonal mouse anti-human CD31 (ready-to -use, Dako, Glostrup, Denmark), monoclonal mouse anti-human CD34 class II (ready-to-use, Dako, Glostrup, Denmark), monoclonal mouse anti-human CD105 (dilution 1:20, Dako, Glostrup, Denmark), and rabbit polyclonal antibody-CD133 (dilution 1:200, Biorbyt, California, USA). After blockade of endogenous peroxidase activity, slides were incubated for 1 h with primary antibodies. After washing, slides were incubated with appropriate secondary antibodies labeled with HRP. HRP activity was amplified with FLEX+ Mouse Linker. Visualization was performed with Flex DAB+ chromogen. Slides were counterstained with haematoxylin and coverslipped with CoverGrip Sealant (Biotium, California, USA), and images were captured using an Olympus microscope.

### Flow cytometry analysis and isolation of CD105+ and CD133+ cells

CD105-FITC (BioLegend, California, USA) and CD133-APC (Miltenyi Biotec GmbH, Bergisch Gladbach, Germany) antibodies were used for all flow cytometry experiments. RCC cell lines were cultured as previously mentioned and washed with PBS prior to harvesting. RCC cells were harvested using Accutase cell detachment solution. FACSCalibur (BD biosciences, California, USA) was used for simple cell acquisition and analyzing samples to establish target protein markers in RCC cell lines. FACSAriaII (BD biosciences, California, USA) was used for cell sorting. Ten thousand cells were acquired for flow cytometry (FACScalibur) before analysis. Flow cytometry data analysis, dot plots, and histograms were prepared using FCS Express 5.1 (DeNovo software, California, USA).

### Analysis of MSC markers on isolated CD105+ cells

BD Stemflow™ Human MSC Analysis Kit (BD biosciences, California, USA), CD24-FITC, and CD146-PE (both bought from Miltenyi Biotec GmbH, Bergisch Gladbach, Germany) were used on sorted CD105+ cells and were acquired using FACS Calibur for enumeration of CD24, CD146, CD73, CD90, CD105, CD11b, CD19, CD34, CD45, and HLA-DR markers. All antibody staining was prepared according to manufacturer protocol. Each sample was run with proper isotype control.

### Alkaline phosphatase assay

A StemTAG^TM^ alkaline phosphatase (AP) staining kit (Cell Biolabs, California, USA) was used to verify the presence of AP expression in RCC cell lines and isolated CD105+ cells. The procedure was conducted according to the manufacturer protocol after cells were cultured in a 24-well plate for 3 days until they become confluent.

### Reverse transcription and real-time PCR

Total RNA from RCC cell lines was isolated using Total RNA Mini Plus (A&A Biotechnology, Gdynia, Poland), as described in the protocol. RNA quality and concentrations were determined by measuring the absorbance of 230, 260 nm, and 280 nm, using μDrop plate from Multiskan™ GO microplate spectrophotometer (ThermoFisher Scientific, Massachusetts, USA). 5μg of total RNA was reverse transcribed using TranScriba kit (A&A Biotechnology, Gdynia, Poland), as mentioned in the protocol. The cDNA was stored in -20°C until the real-time PCR experiment was carried out. 0.5 μl (50 pmol/μL) of pre-designed primer pair solution for Oct-4, Nanog and Ncam, was used from StemTAG^TM^ (Cell Biolabs, California, USA) in a 20 μl volume cDNA template and FastStart Essential DNA Green Master mix kit from Roche according to protocol (Basel, Switzerland). All stem genes were analyzed in separate PCR tubes in triplicate. The real-time PCR was done on a LightCycler Nano Instrument (Roche, Basel, Switzerland). The data were obtained using LightCycler Nano software 1.0 (Roche, Basel, Switzerland). Relative mRNA expression levels were then normalized by using the mRNA level of the reference gene (PPIA) as an endogenous control in each sample. mRNA data were analyzed using the comparative Ct method [32].

Primers sequence used for the PPIA (123 bp) gene:

Forward: 5'-TGTGTCAGGGTGGTGACTTC-3'

Reverse: 5'-TTGCCATGGACAAGATGCCA-3'

### Soft agar colony formation assay under hypoxic vs normoxic condition and serum concentration

Fetal bovine serum (BIOCHROM GmbH, Cambridge, UK) of 2%, 5%, and 10% concentrations was used under normoxic (20% O_2_) vs. hypoxic (1% O_2_) conditions for checking the clonogenic potential of RCC cells. Two thousand cells were seeded in triplicate in a six-well plate and incubated for four weeks. The term *plating efficiency* (PE) was used to indicate the percentage of cells seeded into a six-well plate that finally grow to form a colony. The mean number of colonies observed was counted from triplicates: PE = $\frac{Number\;of\;colonies\;counted}{Number\;of\;cells\;plated}\;X\;100$

### Hanging drop assay and 3D floating spheres for isolated CD105+ cells

Hanging drop assay was used for the aggregation property of cancer cells. It is also a very critical parameter for measuring cell-cell interaction and cell-substratum adhesion through generation of 3D spheroids under physiological conditions. This simple method can also be used to elucidate the role of cell-cell interaction between two (or more) different cell populations. The factors that could critically affect tumor cell metastasis are aggregation and the adhesive properties of the cells. This property is usually altered in the tumor cells. This assay was performed as previously described by Foty et al. [33]

### Microarray procedure

CD105+ cells were isolated from ACHN (metastatic RCC) and Caki-2 (primary RCC). A healthy kidney epithelial cell line (ASE) was used as a control for both RCC cell lines. RNA was extracted using Total RNA Mini Plus (A&A Biotechnology, Gdynia, Poland). RNA quality and integrity were measured by BioAnalyzer 2100 (Agilent, California, USA) before a microarray experiment was carried out. Amplification, labeling, generation of cRNA, and hybridization were done by PERLAN Technologies (Warsaw, Poland) on Agilent’s human GE 4x44K v2 (G4845A) (California, USA) microarrays, as described previously by Stankiewicz et al. [34].

### Bioinformatics analysis

#### Differentially expressed genes

Microarray data normalization, quality control, principal component analysis, and filtered-on flags (detected and not detected) were performed using GeneSpring GX 13.0 (Agilent, California, USA). Statistical analysis of the gene-expression microarray data was performed using moderate t-test and multiple testing corrected with the Bonferonni FWER algorithm. The *p*-value computation was conducted by an asymptotic method. The statistical significance was assessed at *p*<0.05, and a fold change cutoff ≥2.0 (up-/down-regulated) was chosen to identify genes that were differentially expressed and would later perform other bioinformatics analysis.

#### Pathway and network analysis by Ingenuity Pathway Analysis (IPA)

The “core analysis” function included in IPA software (Qiagen, Hilden, Germany) (including biological processes, canonical pathways, upstream transcriptional regulator, and gene networks) was used to interpret the common differentially expressed genes (1411 up- and down-regulated genes) between CD105+ cells from Caki-2 and ACHN. Datasets containing gene identifiers and corresponding expression values were uploaded into the IPA software. Each gene identifier was mapped to its corresponding gene object in the Ingenuity Pathway Knowledge Base (IPKB).

#### Enriched Gene Ontology (GO) terms

A complete list of differentially expressed genes from CD105+ cells from a metastatic RCC cell line (ACHN) and a primary RCC (Caki-2) cell line was uploaded into the PANTHER (Protein ANalysis THrough Evolutionary Relationships) [35] classification system to determine which molecular functions were enriched inside CD105+ cells. The most significantly enriched ontologies were presented in a pie chart based on the up-/down-regulated genes list participating in each GO term.

#### Enriched biological pathways using the Kyoto Encyclopaedia of Genes and Genomes (KEGG) database

The list of common differentially expressed genes from CD105+ cells from primary (Caki-2) and metastatic RCC (ACHN) were uploaded to KEGG [36] to verify contribution in biological pathways activation.

### Statistical analysis

All data were expressed as the mean ± standard deviation from at least three experiments. The statistical analysis, data fitting, and graphics were performed using the StatSoft program STATISTICA 12 (Dell Statistica, Oklahoma, USA) and Microsoft Excel 2013 (Washington, USA). The significance difference was analyzed using student’s t-test or ANOVA. A *p* value <0.05 was considered statistically significant for any experiment.

## Results

### Immunocytochemistry shows CD44, CD105, and CD133 protein expression in RCC cell lines

**Fig 2:**
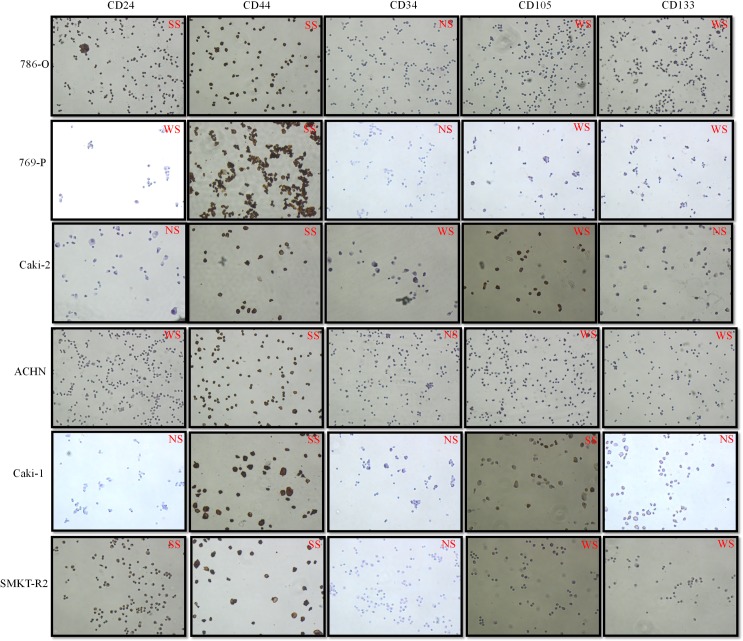
Immunocytochemistry assays for some important SCLCCs markers on primary and metastatic RCC cell lines. Weak staining: WS; Strong staining: SS; No staining: NS.

Immunocytochemistry was used to analyze the expression of potential SCLCCs marker proteins in established RCC cell lines. This assay was crucial for initial screening of different SCLCCs inside a heterogeneous RCC population. We observed constant expression of CD44, CD105, and CD133 in all RCC cell lines **(Fig 2)**. However, CD44 protein was strongly expressed in most of the cell lines, whereas CD105 and CD133 protein expression was weakly/moderately expressed compared to cells with CD44 expression. Protein expression for CD34 and CD24 markers was not constant in all cell lines. Some primary RCCs (786-O and SMKT-R2) show strong CD24 expression, whereas others do not express this marker. In addition, CD34 protein expression was not observed during immunocytochemistry staining.

### Flow cytometry analysis shows the presence of CD105+ as a dominating marker

**Fig 3:**
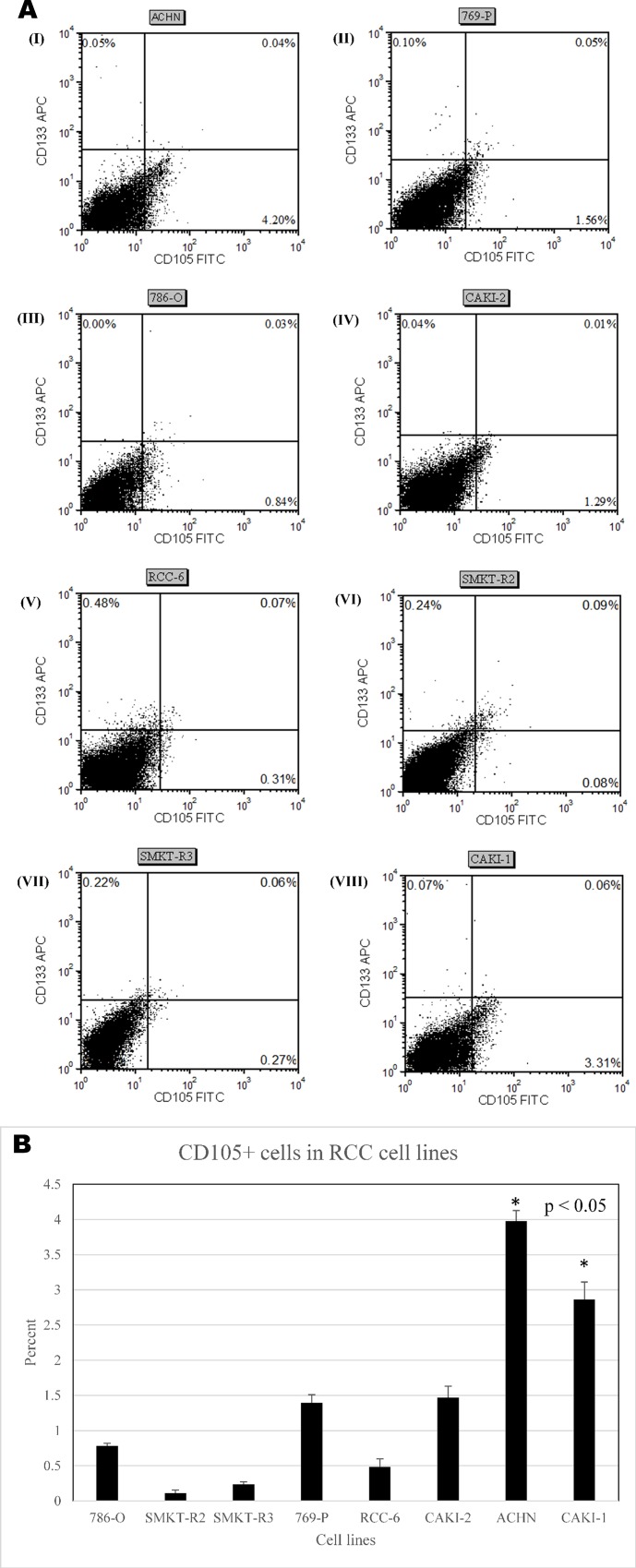
**(A)** Dot plots showing RCC cell lines single positive for CD105-FITC on x-axis and single positive for CD133-APC on y-axis. (I) ACHN; (II) 769-P; (III) 786-O; (IV) Caki-2; (V) RCC-6; (VI) SMKT-R2; (VII) SMKT-R3; Caki-1(VIII). **(B)** Graph showing percentage of CD105+ cells in RCC cell lines. The bar chart depicts the single intensity as percentage of CD105+ cells in all cell lines. Percentage of CD105+ cells in metastatic RCC cell lines (ACHN and Caki-1) was higher comparing to primary RCC cell lines (786-O, SMKT-R2, SMKT-R3, 769-P, RCC-6, Caki-2) (* p<0.05).

We have analyzed the presence of CD105+ and CD133+ cells in human primary and metastatic RCC cell lines. The objective of this experiment was to select the potential surface marker for identifying SCLCCs in established RCC cell lines. The other objective was to select the most promising primary and metastatic RCC cell lines for isolation of SCLCCs for gene-expression profiling. Our flow cytometry analysis showed CD105+ cell presence in most of the cell lines (SMKT-R3, Caki-2, 786-O, 769-P, RCC-6, Caki-1, ACHN). However, CD133+ cells were found in only few cell lines (SMKT-R2, SMKT-R3, RCC-6). **Table 1** and **Fig 3A** were prepared to show cell lines that are positive or negative for CD105 and CD133. The data represented the results of three analyses. CD105+ cells constituted 0.18% to 4.20±0.30%, depending on cell line. Moreover, CD105+ cell population in metastatic RCC cell lines (ACHN and Caki-1) was higher compared to primary RCC cell lines and statistical significance **(Fig 3B)**.

**Table 1:** Showing RCC cell lines expressing CD105+ and CD133+ cells in monolayer culture. **+:** cells found; ˗: No cells found; **P**: primary cell line; **M:** metastatic cell line; **pRCC**: papillary RCC; **ccRCC**: clear cell RCC.

	Markers for stem cell-like cancer cells (SCLCCs)		
Cell lines	CD105+ cells	CD133+ cells	RCC subtype
SMKT-R3 (P)	**+**	**+**	pRCC
Caki-2 (P)	**+**	−	pRCC
786-O (P)	**+**	−	Primary ccRCC
SMKT-R2 (P)	−	**+**	Primary ccRCC
769-P (P)	**+**	−	Primary ccRCC
RCC-6 (P)	**+**	**+**	Primary ccRCC
Caki-1 (M)	**+**	−	Metastatic ccRCC
ACHN (M)	**+**	−	Metastatic pRCC

### AP is expressed in RCC cells in culture

**Fig 4:**
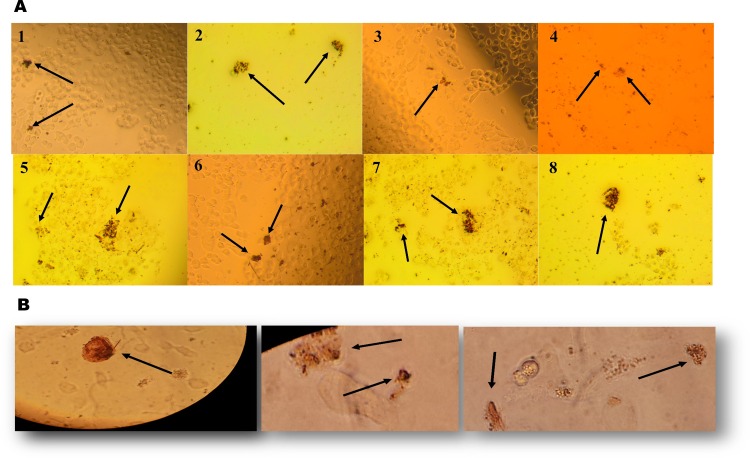
**(A)** Alkaline phosphatase staining of RCC cell lines as indicated by red color (1: 786-O; 2: SMKT-R2; 3: SMKT-R3; 4: 769-P; 5: ACHN; 6: Caki-2; 7: Caki-1; 8: RCC-6). **(B)** Sorted CD105+ cells were found positive for alkaline phosphatase staining. Black arrows indicate red stained CD105+ cells with alkaline phosphatase.

High expression/activity of AP is the universal marker for stem cells. AP activity in somatic stem cells was reported in various sets of MSCs [37, 38]. To investigate the hypothesis that mesenchymal stemlike cells are present in established RCC cell lines, we have performed AP staining to distinguish undifferentiated stemlike cells in a monolayer culture. Our data demonstrate that some RCC cell lines show strong AP activity that is easily visible under light microscope as red stained colonies **(Fig 4A).**

### Isolated CD105+ cells were positive for AP staining

CD105 is a marker for MSCs. Recent reports demonstrated higher expression of AP linked with stemness markers of MSCs [39, 40]. Therefore, exploring whether human renal cell carcinoma is derived from MSCs (CD105+) also shows this stemness property. FACS sorted CD105+ cells were stained with AP, and red stained cells were examined using a light microscope **(Fig 4B)**. Sorted CD105+ cells were found to be positive for AP activity.

### Metastatic ACHN cells have higher expression of Oct-4 and Nanog genes comparing primary RCC cells

**Fig 5:**
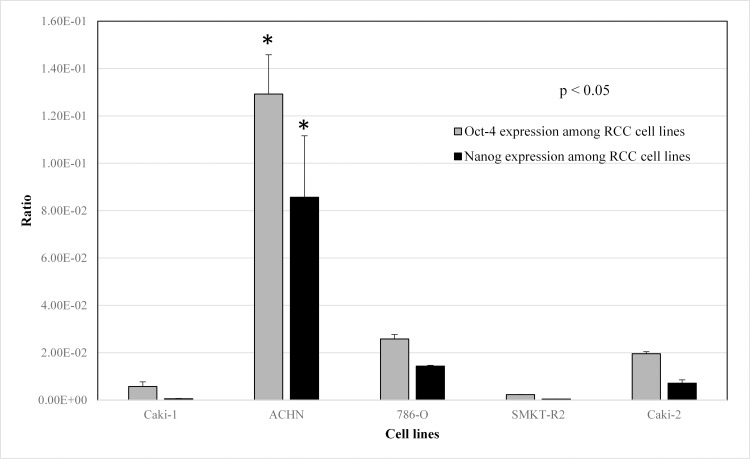
Real-time PCR analysis of Oct-4, Nanog and Ncam stem cell genes in RCC cell lines. Metastatic cell line ACHN showed higher expression for Oct-4 and Nanog comparing to rest of cell lines (* p<0.05). Ncam expression was not observed in any RCC cell line.

The primary goal of performing the RT-PCR experiment was to select the most promising RCC cell lines enriched with stemness-like properties. Therefore, we have selected three stem-cell-related genes (Oct-4, Nanog and Ncam) to verify their expression in our RCC cell lines. The results observed after this analysis were useful in selecting RCC cell lines for isolation of SCLCCs (CD105+) cells for gene expression analysis. The expression of the Oct-4 and Nanog gene was significantly overexpressed in a metastatic RCC cell line (ACHN) compared to all other RCC cell lines **(Fig 5).** However, expression for the Ncam gene has not been detected in any cell lines.

### Higher clonogenic potential of a metastatic RCC cell line under hypoxic conditions

**Fig 6:**
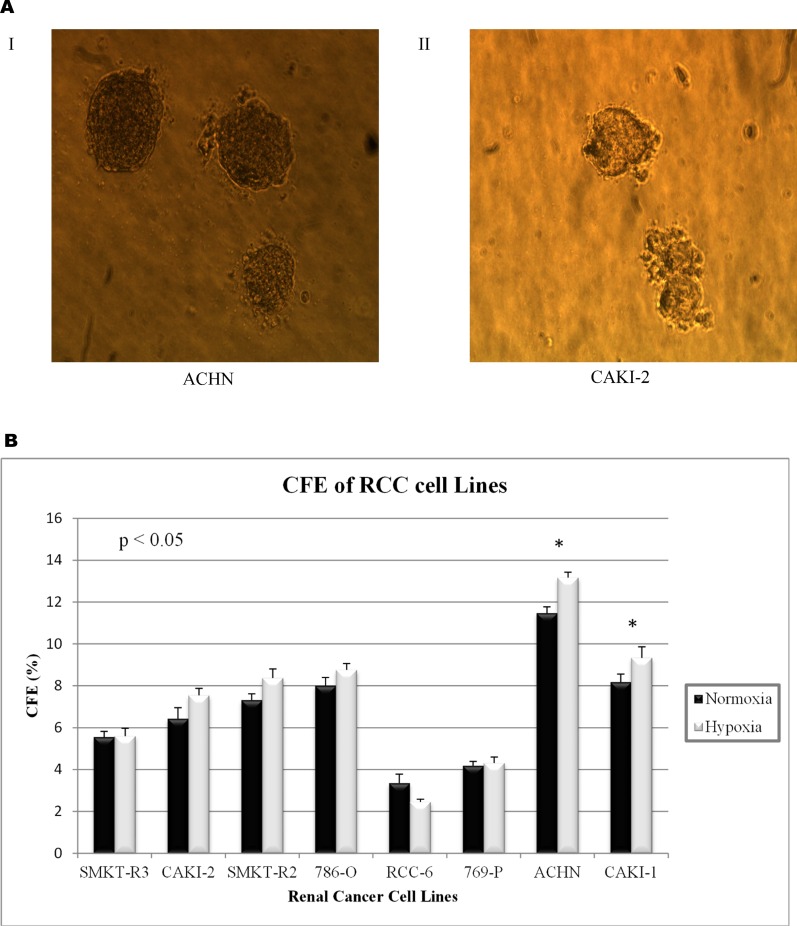
**(A)** Representative picture of colonies formed by metastatic (I: ACHN).and primary (II: Caki-2) cell lines. **(B)** Colony forming efficiency (CFE) of RCC cell line under normoxic (20% O_2_) and hypoxic (1% O_2_) condition. Cell lines derived from metastatic RCC site (ACHN and Caki-1) demonstrate greater potential of forming colonies comparing to primary RCC cell lines (* p<0.05). However, CFE of the same cell line does not have any statistical significance results observed under normoxic vs hypoxic condition.

Colony formation assay (CFA) was used to examine the characteristics of single cells from RCC cell lines grown under different environmental factors (hypoxia: 1% O_2_ vs. normoxia: 20% O_2_ condition and 2%, 5%, and 10% serum concentration). Our result showed that a higher concentration (10% FBS) of serum in culture media was optimal for colony-forming ability **(S1 Fig)**. **Fig 6A** shows a representative picture of colonies formed by two cell lines. In-addition, results were statistically significant in the colony-forming ability of individual cell lines influenced by hypoxia or low oxygen levels **(Fig 6B)**. However, the colony-forming ability was higher in metastatic RCC cell lines (ACHN and Caki-1) when compared with primary RCC cell lines, such as 786-O, SMKT-R2, SMKT-R3, RCC-6, 769-P, and Caki-2. These findings parallel previous results for which we have observed high expression of such genes as Oct-4 and Nanog, and a higher percentage of CD105+ cells in ACHN cell lines.

### *In vitro* characteristics of CD105+ cells

**Fig 7:**
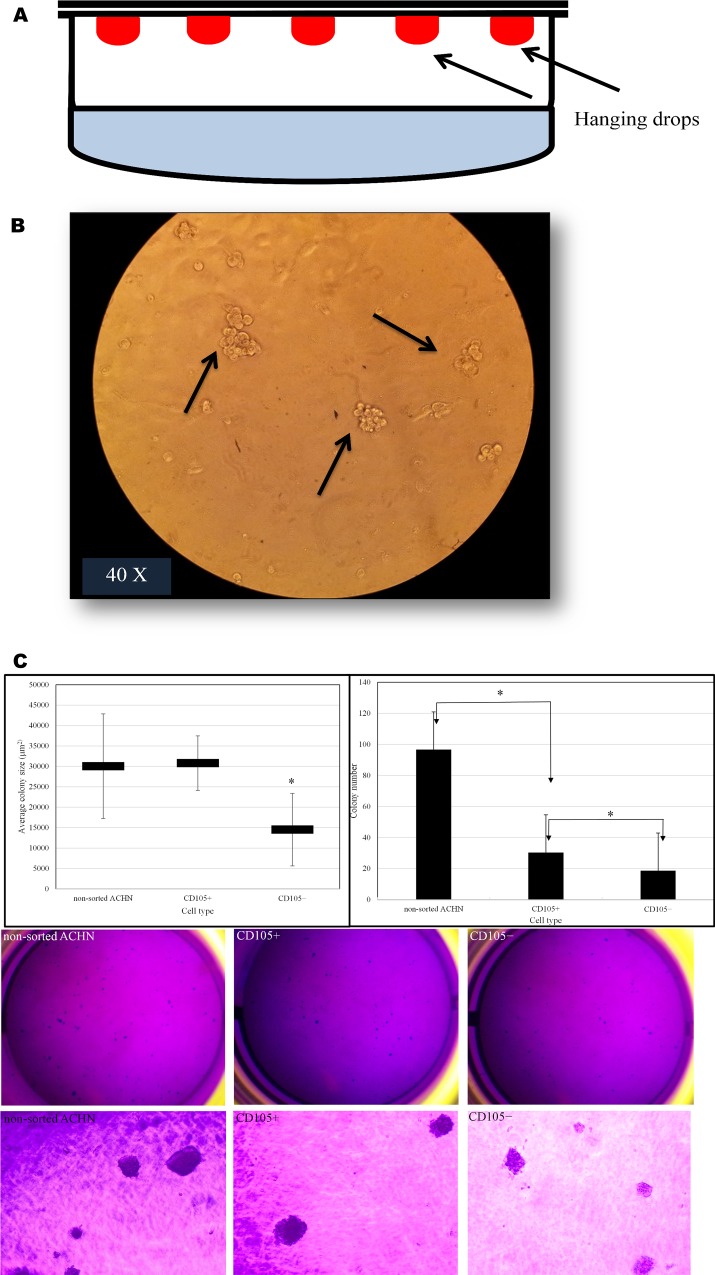
Hanging drop assay. **(A)** General diagram of hanging drop in Petri dish. Red drop showing cultured cells on the roof of petri dish. **(B)** Arrows showing 3D grape-like floating spheres formed by isolated CD105+ cells. **(C)** The colony formation of non-sorted ACHN, CD105+ and CD105− cells from ACHN cell line. The cells were seeded in 6-well plates and cultured for 1 week at 37°C, 5% CO_2_. After staining with crystal violet, the number of colonies formed and size were calculated. The results showed that in non-sorted ACHN and sorted CD105+ cells, the number of clones were more and bigger in colony size than those formed by CD105− cells only (*p<0.05). Representative picture of colonies formed by non-sorted ACHN, CD105+, and CD105− cells.

Hanging drop assay and soft agar colony formation assay was performed on isolated CD105+ cells to characterize their behavior in specific culture conditions. We used hanging drop method to learn how these cells adapt in hanging conditions and whether there is cell-cell interaction between CD105+ cells. Our results showed that more cell-cell interaction was observed between CD105+ cells compared with non-sorted cells from the ACHN cell line; it was easily visible as a 3D grape-like shape formed by CD105+ cells during culturing **(Fig 7B).** These 3D colonies were irregularly shaped. We used soft agar colony formation assay to further analyse the colony forming ability of sorted CD105+ cells comparing to CD105− and non-sorted ACHN cells. Our analysis showed that CD105+ cells were more colony forming when compared to CD105− cells and the results were statistical significant (**Fig 7C**). Colonies formed by CD105+ cells were round and bigger in size, while colonies formed by CD105− cells were smaller in size. However, the number of colony formed by non-sorted ACHN was higher comparing to colonies formed by CD105+ cells or CD105−.

### Human mesenchymal stem cell (hMSC) markers were observed on CD105+ isolated cells

**Fig 8:**
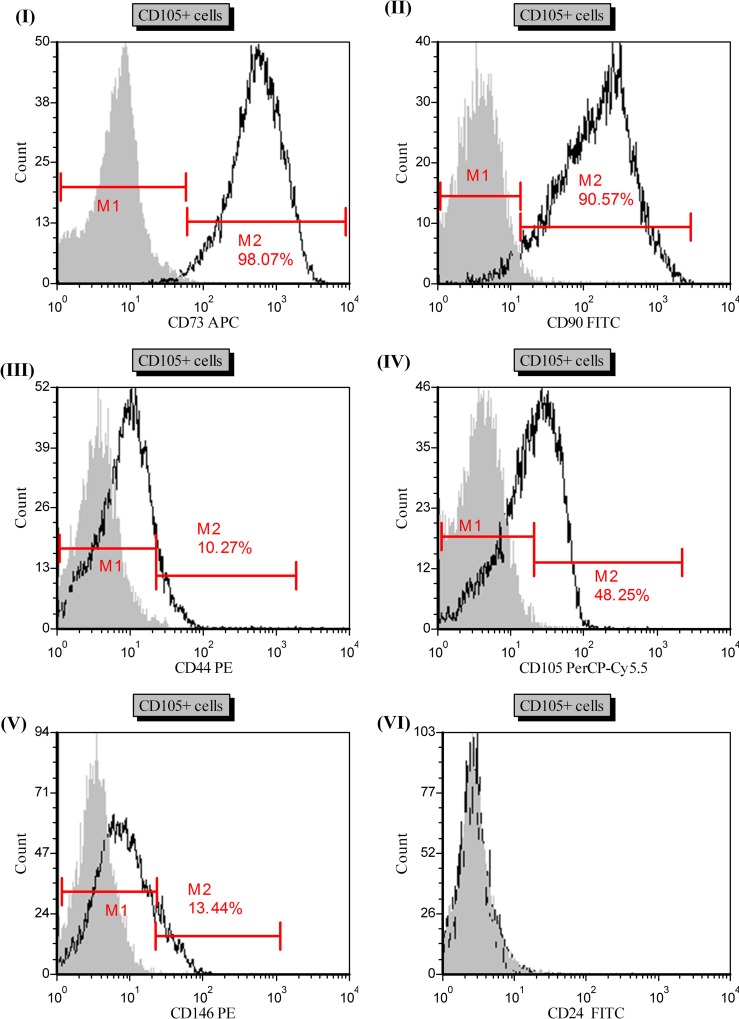
Representative histograms (I-V) of hMSC markers (CD73, CD90, CD44, CD105, CD146) analysis on isolated CD105+ cells. Gated region M1 and M2 as a marker to observe the changing levels of fluorescence intensity. M1 gate shows negative population and M2 gates shows the observed intensity change and percentage of positive cells. Grey filled histogram- isotype control, black line histogram- stained cells.

Because CD105 is an MSC marker, we started looking for mesenchymal stemness characteristics of CD105+ cells isolated from ACHN and Caki-2 cell lines. Histograms (I) and (II) in Fig 8 show that CD73 and CD90 were highly expressed by CD105+ cells at 98.07% and 90.57%, respectively. Histograms (III) and (V) showed that CD105+ cells also expressed CD44 (10.27%) and CD146 (10.28%) markers, respectively. We also re-analyzed isolated CD105+ cells after maintaining them under normal culture (10% FBS) conditions for 5 days. Histogram (IV) showed re-analysis of CD105 after 5 days of culture; less than half CD105+ cells were able to maintain CD105 marker, showing the transient nature of CD105+ cells. We have also checked CD24 markers; however, these cells does not express CD24 (**Fig 8 VI**). **Table 2** was prepared to show the markers that were expressed by CD105+ cells. We also checked expression of CD24 and CD146 markers after subsequent days of culture, but their expression did not change with time (data not shown).

**Table 2:** Expression of other markers on isolated CD105+ cells. **+**: positive for marker; **−**: negative for marker.

	CD90	CD73	CD44	CD105	CD146	AP	CD24	CD34	CD11b	CD19	CD45	HLA-DR
**CD105+ cells**	**+**	**+**	**+**	**+**	**+**	**+**	**−**	**−**	**−**	**−**	**−**	**−**

### Description of differentially expressed genes in CD105+ cells isolated from primary and metastatic RCC

**Fig 9:**
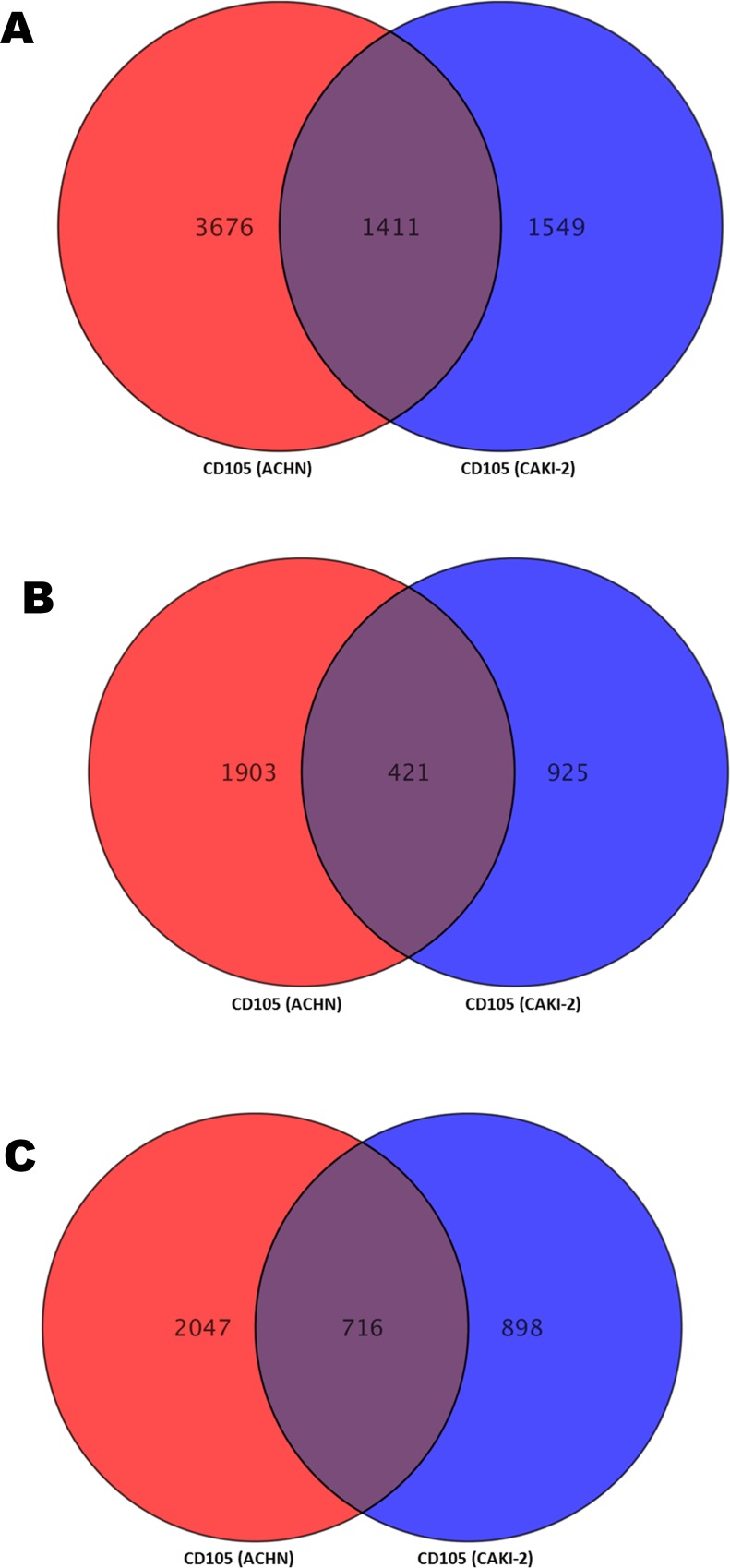
**(A)** Differentially expressed genes between CD105 (ACHN) and CD105 (Caki-2). **(B)** Up-regulated genes between CD105 (ACHN) and CD105 (Caki-2). **(C)** Down-regulated genes between CD105 (ACHN) and CD105 (Caki-2).

Complete list of differentially expressed genes inside CD105+ cells isolated from Caki-2 and ACHN (separately compared with ASE-5063) was submitted as supplementary data (**S1 and S2 Tables**). Five thousand and eighty-seven genes were differently expressed (2,324 up and 2,763 down-regulated) in metastatic CD105 (ACHN) cells and 2,960 genes were differentially expressed (1,346 up- and 1,614 down-regulated) in primary CD105 (Caki-2). Comparing differentially expressed datasets from CD105 (ACHN) and CD105 (Caki-2) reveals that 1411 genes were commonly differentiated (up- and down-regulated) in these cells (**Fig 9A**) (**S3 Table**). A Venn diagram (**Fig 9B and 9C**) was drawn by comparing only up-regulated genes (2,324 vs 1,346 genes) and only down-regulated genes (2,763 vs 1,614 genes) from CD105 (ACHN) and CD105 (Caki-2) cells. Four hundred and twenty-one genes were up-regulated genes only and 716 genes were down-regulated only, whereas 274 genes were either up- or down-regulated between CD105 (ACHN) and CD105 (Caki-2) cells. These common differentially expressed gene lists (1411 genes) were exported for further analysis. The data discussed in this publication have been deposited in NCBI’s Gene Expression Omnibus [41] and are accessible through GEO series accession number GSE84546.

### GO analysis

The most significantly enriched GO terms based on molecular function associated with differential expressed genes are listed below:

#### Molecular function GO terms associated with 5,087 differential expressed genes in metastatic CD105+ cells

**Fig 10:**
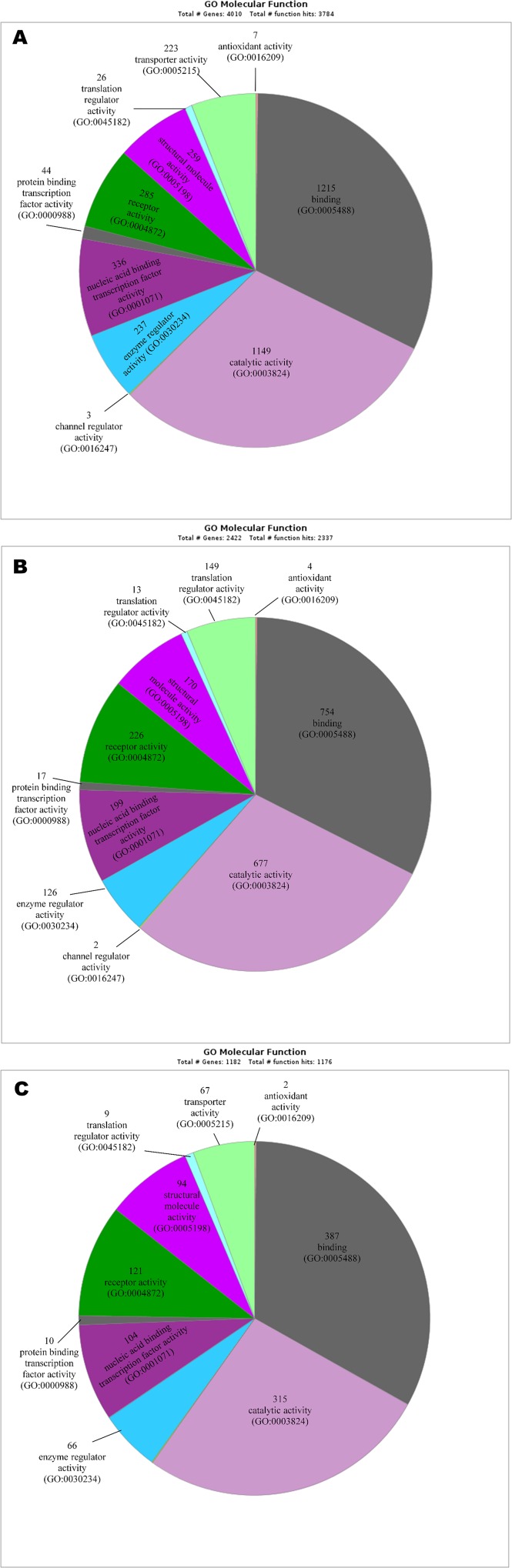
The most significant enriched gene ontologies based on molecular function-MF. **(A)** Up and down-regulated genes expressed in CD105+ cells from metastatic ACHN cell line. **(B)** Up and down-regulated genes expressed in CD105+ cells isolated from primary Caki-2 cell line. **(C)** Common up and down-regulated genes expressed in CD105 (ACHN) and CD105 (Caki-2). The number indicates a count of altered genes that fall into certain category together with GO ID number.

Amino acid trans-membrane transporter activity-GO:0015171, carbohydrate trans-membrane transporter activity-GO:0015144, lipid transporter activity-GO:0005319, trans-membrane transporter activity-GO:0022857, antigen binding-GO:0003823, calcium ion binding-GO:0005509, calcium-dependent phospholipid binding-GO:0005544, chromatin binding-GO:0003682, lipid binding-GO:0008289, nucleic acid binding-GO:0003676, nucleotide binding-GO:0000166, protein binding-GO:0005515, transcription factor binding transcription factor activity-GO:0000989 (**Fig 10A**).

#### Molecular function GO terms associated with 2,960 differential expressed genes in primary CD105+ cells

Antigen binding-GO:0003823, calcium ion binding-GO:0005509, calcium-dependent phospholipid binding-GO:0005544, chromatin binding-GO:0003682, lipid binding-GO:0008289, nucleic acid binding-GO:0003676, nucleotide binding-GO:0000166, protein binding-GO:0005515, G-protein coupled receptor activity-GO:0004930, GABA receptor activity-GO:0016917, acetylcholine receptor activity-GO:0015464, cytokine receptor activity-GO:0004896, glutamate receptor activity-GO:0008066, ligand-activated sequence-specific DNA binding RNA polymerase II transcription factor activity-GO:0004879, transmembrane receptor protein kinase activity-GO:0019199, transmembrane receptor protein serine/threonine kinase activity-GO:0004675, transmembrane receptor protein tyrosine kinase activity-GO:0004714, tumor necrosis factor receptor binding-GO:0005164, and tumor necrosis factor-activated receptor activity-GO:0005031 (**Fig 10B).**

#### Molecular function GO terms associated with 1411 common differential expressed genes in primary and metastatic CD105 cells

Antioxidant activity-GO:0016209, binding -GO:0005488, catalytic activity-GO:0003824, channel regulator activity-GO:0016247, enzyme regulator activity-GO:0030234, nucleic acid binding transcription factor activity-GO:0001071, protein binding transcription factor activity-GO:0000988, receptor activity-GO:0004872-structural molecule activity-GO:0005198, translation regulator activity-GO:0045182, transporter activity GO:0005215, deaminase activity-GO:0019239, enzyme regulator activity-GO:0030234, helicase activity-GO:0004386, hydrolase activity-GO:0016787, isomerase activity-GO:0016853, ligase activity-GO:0016874, lyase activity-GO:0016829, oxidoreductase activity-GO:0016491, and transferase activity-GO:0016740 (**Fig 10C).**

### Proliferation and apoptosis pathways are overexpressed using KEGG analysis in CD105+ cells

The common enriched biological pathways in CD105+ cells from primary and metastatic RCC are presented in **Table 3.** According to the KEGG database, the most significantly enriched common pathways in these cells were “Rap1 signaling pathway,” “PI3K-Akt signaling pathway,” “Cytokine-cytokine receptor interaction,” and “Hippo signaling pathway,” with a gene score more than 12 **(S2 Fig).**

**Table 3:** The most significant enriched pathways in SCLCCs-CD105+ cells examined by KEGG database.

Biological pathways associated with Up-/Down-regulated genes	Total gene number	Data describing participation of altered gene in certain pathways
**ko04015: Rap1 signaling pathway**	11+11 = 22	**Down genes:** ADCY9, APBB1IP, ARAP3, CDC42, FGF2, FGFR2, ID1, ITGB2, LAT, LPAR1, MET
		**Up genes:** ADCY4, CTNNB1, F2R, INSR, ITGB3, KIT, LPAR2, LPAR5, PDGFB, RAP1GAP, SKAP1
**ko04151: PI3K-Akt signaling pathway**	11+16 = 27	**Down genes:** FGF2, FGFR2, IL6, IL7, IL7R, ITGB4, LAMA4, LAMB3, LPAR1, MET, SGK2
		**Up genes:** BCL2, F2R, FN1, INSR, IRS1, ITGA1, ITGA11, ITGB3, ITGB8, KIT, LAMC2, LPAR2, LPAR5, PDGFB, SGK1, SYK
**ko04060: Cytokine-cytokine receptor interaction**	10+9 = 19	**Down genes:** FLT3LG, IL18, IL20RB, IL22RA1, IL6, IL7, IL7R, MET, TNFRSF14, TNFSF14
		**Up genes:** BMP7, CXCL12, IL11, KIT, LTB, PDGFB, TGFBR1, TNFRSF11B, TNFRSF21
**ko04390: Hippo signaling pathway**	9+6 = 15	**Down genes:** AFP, AREG, BIRC5, CSNK1D, ID1, ITGB2, SERPINE1, SOX2, WWTR1
		**Up genes:** BMP7, CTNNB1, SAV1, SNAI2, TCF7, TGFBR1
**ko04010: MAPK signaling pathway**	8+4 = 12	**Down genes:** CACNG6, CDC42, DDIT3, FGF2, FGFR2, MAP3K14, MAPT, PTPRR
		**Up genes:** NTF3, PDGFB, RASGRF2, TGFBR1
**ko04630: Jak-STAT signaling pathway**	7+3 = 10	**Down genes:** IL20RB, IL22RA1, IL6, IL7, IL7R, SOCS3, STAT4
		**Up genes:** CISH, IL11, SOCS2
**ko05206: MicroRNAs in cancer**	7+9 = 16	**Down genes:** CDCA5, E2F1, HMGA2, IRS2, MDM4, MET, PRKCE
		**Up genes:** BCL2, BMF, IRS1, ITGB3, MMP9, PDGFB, SHC4, TIMP3, TPM1
**ko05202: Transcriptional mis-regulation in cancer**	6+9 = 15	**Down genes:** DDIT3, HMGA2, IL6, MET, NUPR1, PBX1
		**Up genes:** CD86, FUT8, HPGD, JUP, MMP9, PROM1, SIX1, SIX4, SPINT1
**ko04668: TNF signaling pathway**	6+5 = 11	**Down genes:** CASP10, CASP8, IL6, MAP3K14, SOCS3, TRADD
		**Up genes:** EDN1, ICAM1, MMP9, RIPK3, TNFAIP3
**ko04068: FoxO signaling pathway**	6+4 = 10	**Down genes:** IL6, IL7R, IRS2, KLF2, SGK2, SOD2
		**Up genes**: INSR, IRS1, SGK1, TGFBR1
**ko04014: Ras signaling pathway**	5+5 = 10	**Down genes:** CDC42, FGF2, FGFR2, LAT, MET
		**Up genes:** INSR, KIT, PDGFB, RASGRF2, SHC4
**ko04064: NF-kappa B signaling pathway**	5+5 = 10	**Down genes:** LAT, LY96, MAP3K14, TNFSF14, TRADD
		**Up genes:** INSR, KIT, PDGFB, RASGRF2, SHC4
**ko04310 Wnt signaling pathway**	1+6 = 7	**Down genes:** FOSL1
		**Up genes:** CTNNB1, MMP7, NFATC4, ROCK2, SFRP1, TCF7
**ko04910 Insulin signaling pathway**	2+4 = 6	**Down genes:** IRS2, SOCS3
		**Up genes:** INSR, IRS1, SHC4, SOCS2
**ko04350 TGF-beta signaling pathway**	2+4 = 6	**Down genes:** ID1, PITX2
		**Up genes:** BMP7, DCN, ID4, TGFBR1

### Ingenuity Pathway Analysis (IPA)

#### Enriched canonical pathways in CD105+ cells as revealed by IPA analysis

IPA of common differentially expressed genes from CD105+ cells isolated from primary and metastatic RCC revealed highly significant overlap of a total of 335 canonical pathways with *p* value < 0.05. To select the most significant pathways within our dataset, we used filter criteria of an overlapping minimum of 8 genes to select 38 pathways, as presented in **Table 4**.

**Table 4:** The significant IPA canonical pathways associated with the common differentially expresses genes in SCLCCs-CD105 cells from primary Caki-2 and metastatic ACHN RCC cell lines. The scoring method used for selection of canonical pathways was Fisher’s Exact Test. The ration (r) is calculated by the number of genes involved and diving by the total number of genes in that canonical pathway in IPA.

Ingenuity canonical pathways	-log(p value)	Ratio (r)	Molecules
**Leukocyte Extravasation Signaling**	6.34E+00	1.66E-01	CD99,MMP7,ICAM1,PTK2B,MMP15,JAM2,CXCL12,ABL1,MMP13,MAPK13,CLDN6,ITGB3,ROCK2,CLDN4,PRKCE,VCL,ACTG2,CTNNB1,MMP1,ACTA1,ACTN1,TIMP3,PIK3C2A,THY1,ITGB2,MMP23B,ICAM3,RAP1GAP,CDC42,PRKCD,ITGA1,MMP9
**ILK Signaling**	4.59E+00	1.49E-01	FLNB,SNAI2,FN1,MYH9,BMP2,ITGB8,ITGB3,PPP2R2C,IRS2,ACTG2,VCL,ITGB4,CTNNB1,ACTA1,ACTN1,DSP,FBLIM1,PIK3C2A,SNAI1,VEGFC,MYL7,ITGB2,CDH1,CDC42,IRS1,TNF,MMP9
**Inhibition of Matrix Metalloproteases**	4.05E+00	2.63E-01	TIMP3,MMP7,MMP23B,ADAM12,MMP15,THBS2,ADAM10,MMP13,MMP9,MMP1
**Regulation of the Epithelial-Mesenchymal Transition Pathway**	3.71E+00	1.37E-01	FZD10,SNAI2,TGFBR1,FGF2,PARD6G,FGF13,NOTCH2,CTNNB1,HMGA2,WNT5B,NOTCH3,ESRP2,PIK3C2A,EGR1,SNAI1,FGFR2,ZEB1,MET,FZD8,CDH1,CDH2,JAG1,MMP9,FZD7,WNT5A
**Bladder Cancer Signaling**	3.04E+00	1.63E-01	CXCL8,MMP7,FGF2,SUV39H1,MMP15,ABL1,MMP13,VEGFC,FGF13,CDH1,MMP23B,E2F1,MMP1,MMP9
**Gα12/13 Signaling**	2.98E+00	1.45E-01	F2RL2,PIK3C2A,F2R,PTK2B,CDH6,CDH11,MYL7,ROCK2,CDH2,CDH1,LPAR1,LPAR2,CDH3,CDC42,LPAR5,CTNNB1,CDH13
**Gα12/13 Signaling**	2.98E+00	1.45E-01	F2RL2,PIK3C2A,F2R,PTK2B,CDH6,CDH11,MYL7,ROCK2,CDH2,CDH1,LPAR1,LPAR2,CDH3,CDC42,LPAR5,CTNNB1,CDH13
**TREM1 Signaling**	2.94E+00	1.74E-01	CXCL8,NLRC5,IL18,ICAM1,TLR1,CASP1,LAT2,CD86,IL6,TLR3,TNF,CASP5
**Paxillin Signaling**	2.93E+00	1.53E-01	PIK3C2A,PTK2B,MAPK13,ITGB8,ITGB3,ITGB2,ARFIP2,CDC42,ITGA11,ITGA1,ACTG2,VCL,ITGB4,ACTN1,ACTA1
**Ovarian Cancer Signaling**	2.88E+00	1.38E-01	FZD10,GJA1,MMP7,PIK3C2A,SUV39H1,PTGS1,ABL1,VEGFC,BCL2,FZD8,EDN1,E2F1,EDNRA,CTNNB1,WNT5B,MMP9,WNT5A,FZD7
**Wnt/β-catenin Signaling**	2.40E+00	1.20E-01	FZD10,MMP7,GJA1,TGFBR1,CSNK1D,SOX11,SOX2,CSNK2A2,FZD8,CDH1,CDH2,CDH3,DKK3,PPP2R2C,SFRP1,DKK1,CTNNB1,WNT5B,WNT5A,FZD7
**HER-2 Signaling in Breast Cancer**	2.11E+00	1.45E-01	ITGB2,PIK3C2A,CDC42,PRKCD,CDK6,PRKCE,PARD6G,ITGB4,ITGB8,AREG,ITGB3
**Basal Cell Carcinoma Signaling**	1.96E+00	1.45E-01	FZD8,FZD10,BMP4,BMP2,BMP7,CTNNB1,WNT5B,FZD7,WNT5A,BMP1
**Colorectal Cancer Metastasis Signaling**	1.96E+00	1.04E-01	FZD10,MMP7,TGFBR1,PIK3C2A,ADCY4,MMP15,VEGFC,MMP13,IL6,BIRC5,ADCY9,FZD8,CDH1,MMP23B,TLR1,TLR3,CTNNB1,TNF,PTGER4,WNT5B,MMP9,MMP1,WNT5A,FZD7
**Type I Diabetes Mellitus Signaling**	1.80E+00	1.23E-01	SOCS3,MAP3K14,MAPK13,BCL2,TRADD,HLA-DMA,CD86,SOCS2,HLA-F,CASP8,TNF,CPE,TNFRSF11B
**TGF-β Signaling**	1.69E+00	1.26E-01	BMP4,TGFBR1,CDC42,BMP2,SMAD6,BMP7,MAPK13,SERPINE1,PITX2,PMEPA1,BCL2
**HIF1α Signaling**	1.63E+00	1.20E-01	SLC2A5,MMP7,MMP23B,EDN1,PIK3C2A,MMP15,MMP13,VEGFC,MAPK13,TCEB1,MMP9,MMP1
**VEGF Signaling**	1.62E+00	1.24E-01	ROCK2,PTK2B,PIK3C2A,VEGFC,VCL,ACTG2,SFN,ACTA1,ACTN1,EIF1AY,BCL2
**TNFR1 Signaling**	1.57E+00	1.49E-01	MAP3K14,TRADD,CDC42,TNFAIP3,CASP8,BIRC3,TNF
**PEDF Signaling**	1.47E+00	1.27E-01	ROCK2,SOD2,GDNF,PIK3C2A,MAPK13,ZEB1,CASP8,TCF7,BCL2
**Role of JAK1 and JAK3 in γc Cytokine Signaling**	1.47E+00	1.33E-01	IL7R,SOCS3,PTK2B,PIK3C2A,IRS1,SYK,IRS2,IL7
**NF-κB Signaling**	1.42E+00	1.01E-01	MAP3K14,BMP4,TGFBR1,PIK3C2A,BMP2,TNFAIP3,FGFR2,TRADD,CSNK2A2,IL18,TLR1,PELI1,INSR,TLR3,CASP8,TNF,TNFRSF11B
**PCP pathway**	1.40E+00	1.29E-01	ROCK2,FZD8,FZD10,EFNB1,CTHRC1,WNT5B,FZD7,WNT5A
**IGF-1 Signaling**	1.38E+00	1.13E-01	SOCS3,IGFBP4,CSNK2A2,IGFBP6,PIK3C2A,IRS1,SOCS2,IGFBP5,IRS2,SFN,IGFBP2
**p53 Signaling**	1.35E+00	1.12E-01	MDM4,SNAI2,PIK3C2A,JMY,E2F1,CSNK1D,SFN,CTNNB1,BIRC5,BCL2,TP53I3
**Interferon Signaling**	1.22E+00	1.47E-01	IFIT1,OAS1,IFI35,IFITM1,BCL2
**Notch Signaling**	1.09E+00	1.35E-01	NOTCH2,NOTCH3,JAG1,DTX2,HEY1
**STAT3 Pathway**	1.06E+00	1.10E-01	SOCS3,TGFBR1,CISH,FGFR2,SOCS2,MAPK13,INSR,BCL2
**BMP signaling pathway**	1.03E+00	1.08E-01	BMP4,BMP2,SMAD6,GREM1,BMP7,MAPK13,PITX2,BMP1
**IL-6 Signaling**	9.42E-01	9.48E-02	COL1A1,CXCL8,SOCS3,MAP3K14,CSNK2A2,IL18,PIK3C2A,MAPK13,IL6,TNF,TNFRSF11B
**IL-12 Signaling and Production in Macrophages**	9.09E-01	9.16E-02	STAT4,IL18,PIK3C2A,PRKCD,MAF,PRKCE,SERPINA1,MAPK13,IL23A,SAA4,TNF,CLU
**Glioma Invasiveness Signaling**	8.30E-01	1.05E-01	TIMP3,F2R,PIK3C2A,PLAU,MMP9,ITGB3
**RAR Activation**	6.96E-01	8.02E-02	NR2F1,ADCY9,CSNK2A2,DHRS3,ALDH1A1,AKR1C3,PRKCD,ADCY4,BMP2,ALDH1A2,SMAD6,PRKCE,MAPK13,RBP1,MMP1
**Ceramide Signaling**	4.07E-01	7.50E-02	PIK3C2A,SPHK1,PPP2R2C,TNF,TNFRSF11B,BCL2
**Prostate Cancer Signaling**	4.07E-01	7.50E-02	PIK3C2A,SUV39H1,E2F1,ABL1,CTNNB1,BCL2
**Breast Cancer Regulation by Stathmin1**	3.76E-01	6.84E-02	PPP1R14C,PIK3C2A,ADCY4,PPP1R14A,ROCK2,ARHGEF19,ADCY9,CDC42,PRKCD,E2F1,PRKCE,PPP2R2C,ARHGEF18
**mTOR Signaling**	3.20E-01	6.59E-02	RPS4Y1,PIK3C2A,PRKCD,IRS1,RPS23,PRKAA2,PRKCE,VEGFC,RPS4Y2,PPP2R2C,INSR,PRR5L
**B Cell Receptor Signaling**	2.87E-01	6.43E-02	MAP3K15,MAP3K14,APBB1IP,PTK2B,PIK3C2A,CDC42,SYK,EGR1,ABL1,MAPK13,NFATC4

#### IPA network analysis

**Fig 11:**
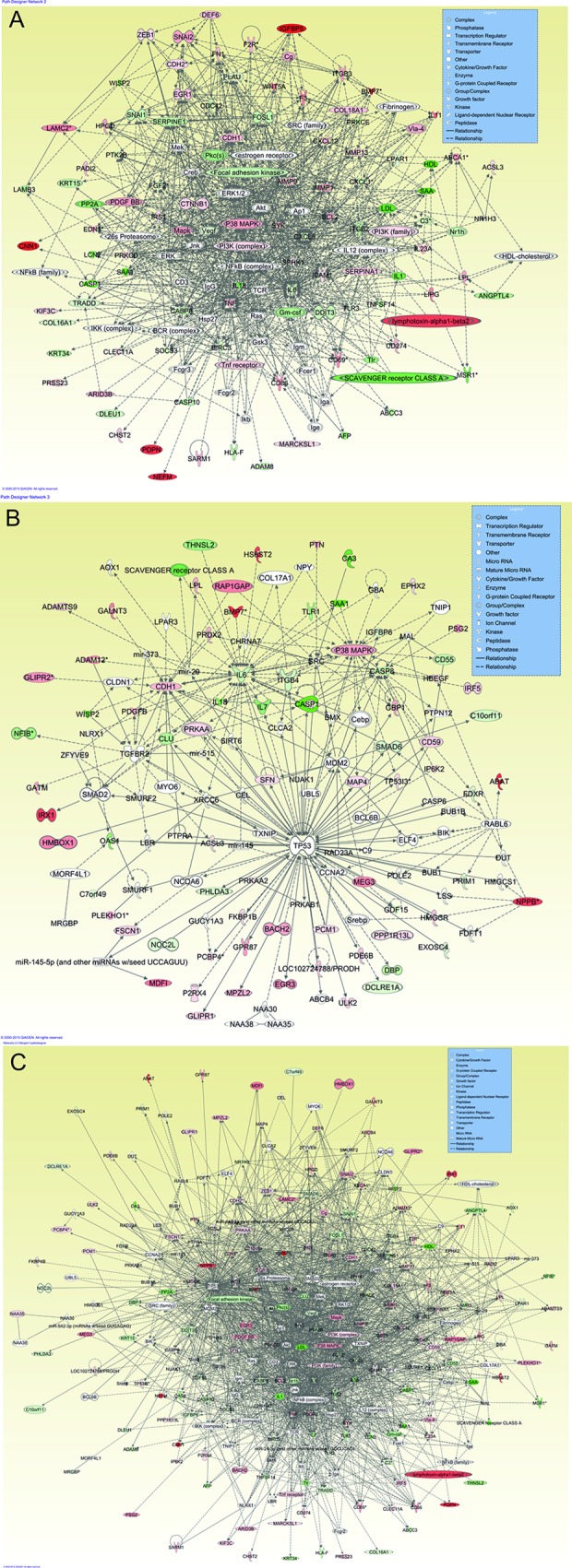
Gene network generated through the use of IPA: The two highest scoring networks: Network **(A)** consist of 65 genes and Network **(B)** consist of 51 genes created from common differentially expressed gene of primary and metastatic CD105+ cells as list are shown. Network **(C)** showed the merged network of Network **(A)** and Network **(B**) composed of IL6 and several of its interaction partners. Color nodes are shaded by their relative expression, green with low expression and red with higher expression, green and red color intensity is relative to expression. The shape of node indicated the major function of the protein. A lines denotes biding of the products of the two genes while a line with an arrow denotes 'act on'. A dotted line denotes an indirect relationship, and solid line denotes direct relationships.

The core analysis function in IPA revealed 13 biological networks comprising a minimum of 34 genes from our dataset. From these 13 networks, we identified two networks with genes responsible for cellular movement, inflammatory response, cell death and survival, cellular growth and proliferation, and cancer **(Fig 11)**.

#### Downstream effects analysis

**Fig 12:**
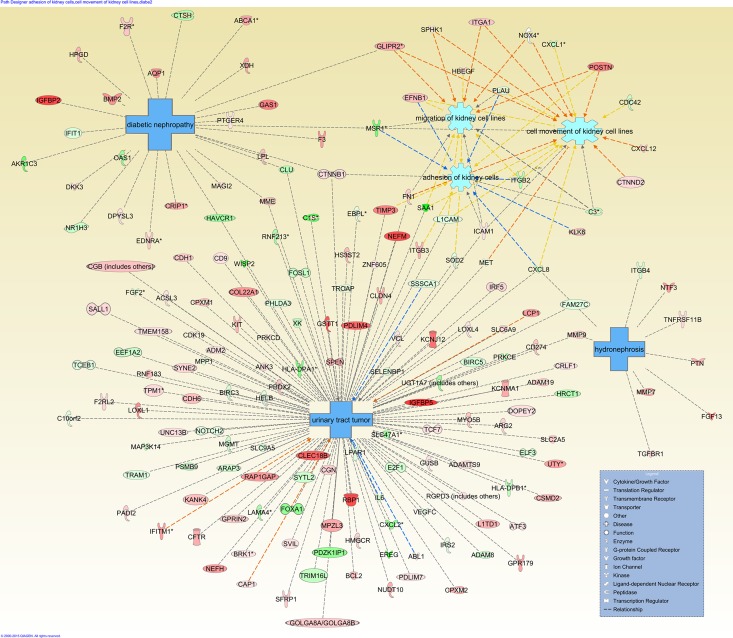
Downstream effect analysis: Ingenuity Pathway Analysis (IPA) Downstream Effect Analysis-based network presenting the mapping of most significant genes connection with renal associated diseases and functions.

Downstream effects analysis in IPA was used to identify biological trends, such as biological processes and diseases associated with common differentially expressed genes of CD105+ cells. We filtered downstream analysis, using the term “renal” to eliminate the results connected with other diseases and functions (**Fig 12**). Fig 12 showed the mapping of the most significant genes connected to renal-associated diseases and functions.

#### Upstream regulator analysis

**Fig 13:**
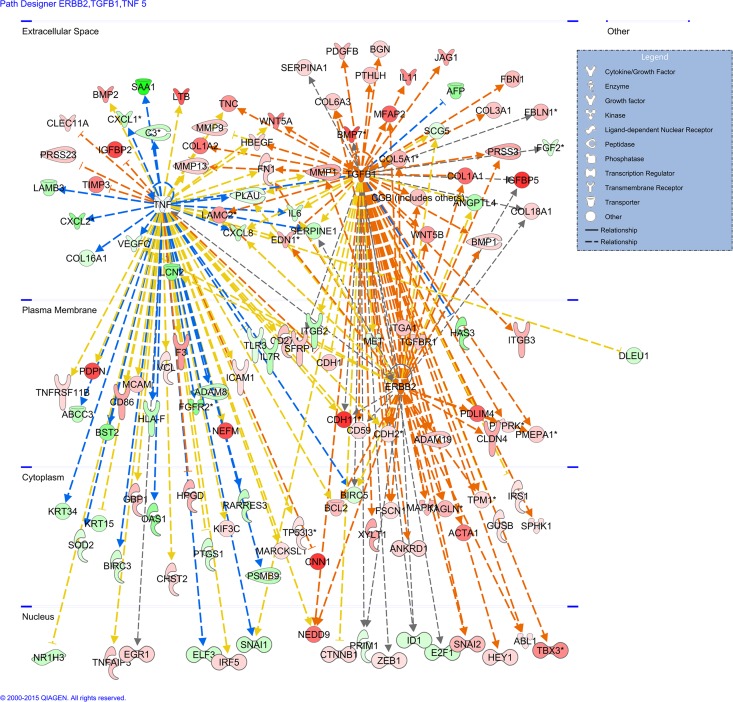
Upstream regulator analysis: Showing the most significant upstream regulators (TGFB1, ERBB2, and TNF) in dataset of SCLCCs-CD105+ cells.

IPA upstream analysis was used to predict the upstream transcriptional regulator from the datasets of common differentially expressed genes of CD105+ cells, based on the literature and compiled in the Ingenuity Knowledge Base. The analysis examines how many known targets of the upstream regulators are present in the SCLCCs-CD105+ cells. An overlapping *p*-value is computed based on significant overlap between genes in the dataset and known targets regulated by the transcriptional regulator. The activation z-score algorithm is used to make predictions. Fig 13 was presented to show the most significant upstream regulator in the dataset of SCLCCs-CD105+ cells. IPA predicted the three top transcriptional regulators activated in our dataset, such as TGFB1, TNF, and ERBB2.

## Discussion and Conclusions

There is increasing evidence to suggest that tumor recurrence is a disease of SCLCCs. A small population of cells consists of SCLCCs with the potential to create new tumors. SCLCCs are similar to normal stem cells and have such properties as high proliferation capability, self-renewing potential, and resistance to chemotherapy, radiotherapy and hormonal therapy. Well-established methods or markers do not currently exist for the isolation and characterisation of SCLCCs from established renal cancer cell lines. Over the years, there has been increased evidence for detection and enumeration of SCLCCs in RCC [17]. Most of this evidence is based on tumor-tissue-specific SCLCCs, while some are based on RCC cell lines [18–20, 42, 43]. SCLCCs have been identified from RCC cell lines based on chemokine receptor CXCR4 [19, 42] and side population cell selection by aldehyde dehydrogenase activity [18, 20, 43], Hoechst 33342 dye [44] and rhodamine 123 [45]. Previously, Bussolati et al. observed renal carcinoma specimens from patients after radical nephrectomy, which showed CD105+ subpopulation is enrich for tumor-initiating cells (TICs) [31] and tumor angiogenesis [46]. These cells were highly tumorigenic *in vivo* experiments and expressed stem cell markers. The frequency of CD105+ cells in all renal carcinoma specimens was (8.06±3.3%) [31]. The other marker chosen for SCLCCs identification was CD133 because CD133+ stem cells were found in normal kidney tissues [47]. However, high expression of CD133 was found in RCC progenitor cells. Inducing ectopic expression of CD133 in human embryonic kidney 293 (HEK293) cells enriched with tumor-initiating properties suggesting that CD133 contributes to the TIC phenotype [48]. In addition, HEK293 CD133^high^ cells were 1000-fold enriched in tumorigenic cells when compared with their CD133^low^ counterpart in SCID mice. In RCC, CD133+ cells were shown to promote angiogenesis in tumors, but not to be responsible for tumor development [22]. CD133+/CD24+/CTR2+ cells derived from RCC specimens were enriched with stem cell-like features but did not express mesenchymal markers like CD105, CD90, except for CD73 marker [21]. Varna et al. identified numerous CD133/CXCR4-coexpressing cells in perinecrotic vs perivascular areas of RCC patients with stem cell-like phenotype [49]. Moreover, CD133/CXCR4 cells were higher in sunitinib treated patients comparing untreated patients. The presence of CD133+ cells in the tumor also showed a prognostic value [50]. To test the hypothesis of SCLCCs existence in RCC, we began to explore whether these two selected subpopulations (CD105+ and CD133+ cells) were also present in established renal cancer cell lines. In the beginning, we used a flow-cytometry approach to analyze the two subpopulations between the eight different RCC cell lines. The results from this analysis will be useful for selecting potentially enriched CD105 or CD133 RCC cell lines. Our FACS analysis confirmed the presence of CD105+ cells in most of the cell lines (**Table 1** and **Fig 3B**). The frequency of CD105+ cells was 0.18% to 4.20±0.30%, depending on cell line. In addition, metastatic RCC cell line ACHN and Caki-1 have the highest percentage of positive cells for CD105. However, CD133+ cells were found in few cell lines **(Fig 3A)**. As a positive control for CD105+ cells, we also analyzed Wharton’s jelly cells using FACS to confirm the accuracy of antibodies used for CD105 analysis (data not shown). Ueda et al. also found 1.5% and 28.9% of CD105+ cells in ACHN and KRC/Y cell lines, respectively, while their side population also expressed higher percentage of CD105+ cells and spheres forming when compared to its non-side population (24.6% vs 4.6%) [18]. Therefore, we chose MSC marker “CD105” for further study in this research. AP activity has been reported in various sets of MSCs [37, 39, 51, 52]. Because we found MSCc-CD105 cells in RCC cell lines, we started to stain MSCs in a monolayer cell culture to distinguish undifferentiated mesenchymal stemlike cells. Our data demonstrate that RCC cell lines have characteristics of MSCs by being positively stained with AP. The results are easily visible a under light microscope as red stained colonies **(Fig 4A).** Many have already reported that AP activity linked with stemness markers for MSCs [37, 39, 40, 52]. In this study, our human RCC-derived MSCs-CD105+ cells also show this stemness property (**Fig 4B**). This property of CD105+ cells will be useful to distinguish undifferentiated mesenchymal stem-like cells from differentiated cells.

Uncontrolled self-renewal is an important mechanism in carcinogenesis [53]. According to cancer stem cell hypothesis, a tumor sustained by a subset of cancer cells with stem-cell-like characteristics. The same molecular pathways that seem to manage the self-renewal process in normal stem cells is also responsible for SCLCCs in tumors [54]. Gene expression of stem cell genes, such as Oct-4, Ncam, and Nanog, is crucial for progression of various human malignancies [55–57]. It is also possible that these genes participate in maintaining the stem-cell-like characteristics in RCC cell lines. Therefore, we investigated these stem markers with the established RCC cell lines to understand whether these cell lines are suitable for stem cell-like cancer cells (SCLCCs) research. The StemTAG tm (CBA-303) PCR primer set offers an efficient system for monitoring stem-cell genes through real-time PCR. The main goal of this experiment was to select the RCC cell lines enriched for stemness genes (Oct-4, Nanog, and Ncam). We verified the expression of these genes between cell lines. Our observation proved that the metastatic cell line ACHN has a higher expression for Nanog and Oct-4 genes compared with primary RCC cell lines, such as 786-O, SMKT-R2, and Caki-2 (**Fig 5**). In addition, ACHN were also enriched for stemness genes when compared with another metastatic cell line, Caki-1. However, we did not detect expression for the Ncam gene in any cell line. This comparison was important for selecting the cell line before isolation of the CD105+ cells. *In vitro* clonogenic assay (or colony-formation assay) is a cell-survival assay based on the ability of a single cell to grow and transform into a separate colony. These colonies are believed to be derived from stem cells, early progenitor cells, and late progenitor cells [58, 59]. In this study, we use this method to examine whether the stemness-like properties exist in different RCC cell lines. CFA is a cell-survival assay based on the ability of a single cell to transform into a single colony. These single colonies are believed to be derived from stem cells [58, 60]. We found that colony-forming efficiency (CFE) was higher in metastatic RCC cell lines (ACHN and Caki-1) than in primary RCC cell lines (**Fig 6B**). These findings parallel other metastatic cancers, such as colon cancer [61], and our results show high expressions of stemness genes, such as Oct-4 and Nanog, and a higher percentage of CD105+ cells associated with metastatic RCC cell line ACHN. Moreover, within the same cell line, hypoxic and normoxic conditions do not have any influence on CFE. Hanging drop assay was used to elucidate the role of cell-cell interaction between isolated CD105+ cells [33, 62]. This method was adopted to generate 3D spheres from CD105+ cells and led to learning how these cells adapt in a hanging culture condition. Our results indicate that there were more cell-cell interactions observed when compared to non-sorted cells from the ACHN cell line. This cell-cell interaction between CD105+ cells leads to the formation of 3D spheres that are highly irregular in shape. Another 3D culture method, colony formation assay was used to test the colony forming ability of CD105+ cells to observe if these cells display SCLCCs properties. Our results show that colony forming capacity resides in CD105+ cell population. The colony formation of CD105+ cells was higher than that of CD105− cells (**Fig 7C**). Moreover, these 3D culture techniques also prevented MSCs from differentiating, compared to the conventional two-dimensional culture [63]. The other advantage of the hanging drop method and soft agar colony formation is that it can provide a more tissue-like environment for sorted cells. In the present study, we found that CD105+ cells are present in established RCC cell lines. This small cell population was previously shown to be tumor-initiating cells (TICs) by Bussolati et al. [31]. Isolated CD105+ cells were farther tested for human MSC markers using FACS analysis and were found positive for CD90, CD73, CD44, and CD146 (**Table 2** and **Fig 8**). All these markers were previously reported in the CD105+ cells isolated from *in vivo* tumors in RCC patients [31]. In addition, CD105+ cells were unable to express the markers for CD24 and hematopoietic lineage markers CD34, CD11b, CD19, CD45, and HLA-DR. We also observed the transient nature of isolated CD105+ cells by showing that less than half of the CD105+ cells were able to maintain CD105 markers under standard culture conditions (**Fig 8 IV**). This result may demonstrate cell differentiation by FBS, and dedicated media should be used to main the CD105+ phenotype. Our results may suggest that the CD105+ cells also exist in established RCC cell lines derived from different tumor sites with MSC-like characteristics.

In this study we have shown for the first time gene-expression profiling of MSCs-CD105+ cells from primary and metastatic RCC. We compared the gene expression profiles of CD105+ cells as SCLCCs isolated from primary and metastatic RCC cell lines compared with a healthy kidney epithelial cell line (ASE). We analyzed the microarray data in three ways. First, we analyzed the differentially expressed genes in CD105+ cells isolated from the primary RCC Caki-2 cell line and compared them with a healthy kidney ASE cell line. Second, we analyzed the differentially expressed genes in CD105+ cells isolated from an ACHN cell line derived from metastatic RCC and compared this with a healthy kidney ASE cell line. Third, because all the experiments shared a common healthy kidney ASE cell line as a reference, the experiments could be compared with each other to identify commonly up- or down-regulated gene signatures between primary CD105 (Caki-2) and metastatic CD105 (ACHN).

Our analysis revealed that 1411 genes were commonly differentiated in CD105+ cells from primary and metastatic RCC. Our analysis showed that there are similarities and differences in expression of these 1411 genes (**S3 Table**). For instance, 421 genes were only up-regulated, 716 genes were only down-regulated, and 247 genes were either up- or down-regulated. These altered genes in CD105+ cells may serve as a molecular marker for the diseased state of the kidney or may have an important role inside the kidney cells. These genes could serve as candidate therapeutic targets. For example, the TGFBR1 (transforming growth factor-β receptor 1) gene was 6.82- and 5.02-fold up-regulated in CD105+ cells from Caki-2 and ACHN cell lines, respectively, when compared with a healthy kidney (ASE cell line). TGFBR1, a protein kinase, plays an important role in tumor progression and migration by taking part in the TGF-β/Smad signaling pathway [64, 65]. It was recently found that elevated TGF-β pathway activity is significantly associated with shortened disease-specific survival in RCC [66]. Another up-regulated gene was TNF-α (tumor necrosis factor-α), which was a 29.41- and 9.60-fold higher expression in CD105+ cells from ACHN and Caki-2, respectively. TNF-α is considered an inflammatory cytokine with anti-tumorigenic properties. Elevated levels of TNF-α in serum from RCC patients are associated with EMT and promote tumorigenicity [67]. In our observation, we found that EMT signaling was enriched inside CD105+ cells, as predicted from KEGG and IPA canonical pathway analysis. Moreover, NOTCH3 and ABCA1 genes were highly up-regulated in metastatic CD105+ cells (59.53- and 26.19-fold, respectively). NOTCH3 overexpression correlates with shorter progression-free/OS in patients with advanced ovarian carcinoma, and inactivation of the NOTCH3 gene leads to decreased cell proliferation and induced apoptosis in ovarian cancer cells [68]. However, there have not been enough studies to show the precise role of NOTCH3 in RCC development. Our results shows that NOTCH3 may be a novel therapeutic in RCC treatment; therefore, further studies must be done. The ABCA1 gene belongs to the ATP-binding cassette family. The ABCA1 protein moves cholesterol and phospholipids across the cell membrane, which is useful for balancing cholesterol levels and maintaining cardiovascular health. In has been reported that overexpression of the ABCA1 gene has been linked to the drug-resistance phenotype in melanoma cells [69]. In this study, we also found that the ABCA1 gene was overexpressed (26.19- and 13.03-fold in metastatic and primary CD105+ cells, respectively). RCC has a drug-resistance phenotype [6, 70], and CSCs/TICs are drug resistant [71], which may suggest the involvement of the ABCA1 gene for developing such profiles through CD105+ cells. Therefore, further study must be performed.

In addition, many commonly down-regulated genes were also observed in CD105+ cells from primary and metastatic RCC. Interleukin-6 (IL6), a cytokine, was highly down-regulated (57.33-fold) inside CD105+ cells from a metastatic ACHN cell line compared to a healthy kidney (ASE) cell line. While in CD105+ cells from a primary Caki-2 cell line, this gene was 4.34-fold down-regulated in comparison to a healthy kidney (ASE) cell line. IL-6 has been associated with a IL-6-JAK1-STAT3 signal transduction pathway, which plays an important role in regulating formation of SCLCCs from non- SCLCCs in human breast cancer cells [72]. In RCC, IL-6 was frequently secreted by cancer cells [73]. In addition, serum levels of IL-6 are associated with anemia and thrombocytosis in RCC patients [73]. The precise function of IL-6 in RCC- SCLCCs is still unknown; therefore, further study is needed to overcome this barrier. The TRADD gene was found to be down-regulated at the same level (2.49- and 2.89-fold in metastatic and primary CD105+ cells, respectively) in CD105+ cells from the previously mentioned tumors. The TRADD protein takes part in the apoptosis process by supressing TNF-α-induced apoptosis through the activation of nuclear factor κB (NF-κB) [74, 75]. Down-regulation of the TRADD gene may be responsible for the TRADD's inability to bind TRAF proteins and suppressed TNF-α-induced apoptosis in RCC. We also found the TRADD gene to be enriched in Wnt signaling using KEGG pathway analysis. However, the exact mechanism of how TRADD effects Wnt signaling is still unknown. Another interesting gene found in this study was ADAM8. ADAM8 overexpression is associated with shorter survival of patients with RCC [76]. In contrast, our study showed ADAM8 was down-regulated in CD105+ cells (3.85-and 4.87-fold in metastatic and primary RCC, respectively). The exact role of ADAM8 in RCC-CSCs/TICs and RCC development is still unknown; therefore, further studies need to be performed.

Knowledge of gene regulatory signaling pathways is considered to be of crucial importance in the understanding of any disease, specifically cancer. This knowledge may lead to a new therapeutic approach that can be applied to new treatment. Our investigation, as revealed by IPA analysis, showed that the most common differentially expressed genes between CD105+ cells among primary and metastatic RCC take part in the leukocyte extravasation signaling pathway, the ILK signaling pathway, the inhibition of matrix metalloproteases pathway, the regulation of the epithelial-mesenchymal transition pathway, the Wnt/β-catenin signaling pathway, the TGF-β signaling pathway, the Type I diabetes mellitus signaling pathway, and the NF-κB signaling pathway (**Table 4**). Similar observations corresponding to our results were also reported in different cancers by others [30, 77–79], although it has been found that tumor cells mimic mechanisms used by leukocytes [80]. Genes responsible for the leukocyte-extravasation process may be important for the immigration of tumor cells from the blood stream into the tissue to form metastatic RCC. Integrin-linked kinase (ILK) signaling regulates several cell-adhesion, integrin-mediated, and growth-factor-regulated functions [81]. ILK expression is upregulated in many types of cancer [79, 82]. In addition, ILK mediates many signaling pathways, and it has been associated in activating P13Kinase/Akt, Wnt, TGF-β, and EMT signaling. We also observed activation of P13Kinase/Akt, Wnt, TGF-β, and EMT signaling in CD105+ cells, which may be responsible for RCC development. These results were further analyzed using KEGG pathway enrichment, and similar findings were obtained.

Our IPA downstream effect analysis of 1411 common genes from CD105+ cells revealed that those genes were also reported in other cancers, suggesting that different cancer types share common pathways. Furthermore, these genes play an important role in renal-associated diseases and functions, such as urinary tract cancer, hydronephrosis, diabetic nephropathy, and migration of kidney cells (**Fig 12**). IPA upstream analysis predicted activation of the three most significant transcriptional regulators in CD105+ cells, such as TGFB1, ERBB2, and TNF. TGFB1 is one of several cytokines that have been previously reported by others to be produced by proximal tubular and renal cancer cells [83, 84]. This may exert tumor-promoting and immunosuppressive effects [85, 86]. Activation of TGFB1 in CD105+ cells might suggest that these cells play a significant role in tumor progression and metastatic development. ERBB2 is a receptor tyrosine kinase belonging to the family of epidermal growth factor (EGF) receptors, and is generally involved in tumor cell differentiation, proliferation, growth, and metastasis [87]. It has also been observed that activation of ERBB2 receptor signaling can enhance chemosensitivity and resistance against EGFR-directed therapeutics in tumors, such as in breast, colorectal, head and neck, and non-small cell lung cancers [88, 89]. There has not been enough data reported on the role of ERBB2 and RCC. Activation of the ERBB2 gene as an important transcriptional regulator in CD105+ cells might suggest its potential role in developing resistance in RCC. Therefore, further investigation is needed for patients who have developed resistance to chemotherapy. Tumor necrosis factor (TNF or TNFα), a multifunctional cytokine, has a dual role in cancer biology [90, 91]. Thus, TNF could be either pro- or anti-tumorigenic in nature. TNF is responsible for intracellular signaling to activate NF-κB and MAPKs. As a major pro-inflammatory cytokine, epidemiological and clinical data suggest that chronic inflammation promotes tumor development and progression. Elevated levels of serum TNF concentration and increased TNF expression have been reported in different cancer patients and tumor tissues [92–95]. It has been shown that TNF-α enhances migration, invasion, and tumorigenicity and induces EMT in RCC [67]. In this research we also found increased expression of TNF in CD105+ cells and an altered EMT pathway. Furthermore, in our findings, TNF was also reported as an important transcriptional regulator via IPA analysis. Others have also reported that TNF can enhance a CSC-like phenotype [96]. This finding indicates that TNF may be involved in RCC development through CD105+ cells and could be used as an indicator of cancer risk, therapy response, and prognosis for RCC patients. The data from the present study showed that RCC-SCLCCs(CD105) have more than one molecular mechanism, and a number of signaling pathways were involved that make an aggressive phenotype.

Additionally, first-time biological interaction network maps for common differentially expressed genes of CD105+ cells were generated through the use of IPA. This bioinformatics tool is based on the IPKB. All differentially regulated probe sets with their corresponding fold change for each comparison were used as input in IPA. Our IPA analysis revealed 13 biological network analyses comprising a minimum of 34 genes from the previously mentioned dataset. From these 13 networks, we identified two networks with genes responsible for cellular movement, inflammatory response, cell death and survival, cellular growth and proliferation, and cancer (**Fig 11**). These biological networks have been generated from relationships between proteins, genes, complexes, cells, tissues, drugs, and diseases obtained from more than 200,000 peer-reviewed scientific publications. The first important gene network identified in CD105+ cells was concentrated around IL6, Akt, NFkB (complex), TNF, P13K (complex), and P38 MAPK (**Fig 11A**). The role of cytokine IL6 in RCC has already been discussed in this paper. IL6 makes direct interaction with NFkB (complex), P38 MAPK, and TNF. Akt/PI3K was also reported in our previous findings, for which it was an important member of a network formed by genes of metastatic clear cell renal cell carcinoma (mccRCC) cell line Caki-1 [97]. Akt regulates molecules involved in cell survival, apoptosis, and Akt inhibition in ccRCC, which could be used as a therapeutic option for patients with elevated Akt activity [98]. Direct interaction was observed between NFkB (nuclear factor kappa B) and TNF-α (tumor necrosis factor-α). This interaction was responsible for inducing EMT in RCC [99], which also suggests an EMT phenotype of CD105+ cells, as observed in our study. P38 MAPK can negatively regulate the initiation of tumors via apoptosis in response to oxidative stress sensing [100]. It has also been observed that P38 MAPK inhibition in breast cancer metastasis was mediated by suppression of MSC migration from primary to metastatic sites [101]. The role of P38 MAPK in SCLCCs-CD105 migration is still unknown; therefore, further studies need to be performed. The second gene network was found around the tumor suppressor gene TP53, together with P38 MAPK and IL6 (**Fig 11B**). P38 MAPK and IL6 were important elements found in both networks (**Fig 11A, 11B and 11C**]), as observed in this research. Inactivation of TP53 has been linked with a poor prognosis and a drug-resistance tumor profile [102]. Drug resistance, a common feature of SCLCCs, makes TP53 an interesting target for further research of SCLCCs-CD105 in RCC. Conversely, the network generated by IPA downstream effects analysis revealed complex prediction of the effects of gene expression changes in CD105+ cells, which contributes to renal-associated diseases and functions. We filtered downstream analysis using the term “renal” to eliminate results related to other diseases and functions. This was done to present mapping of the most significant genes connected to renal- associated diseases and functions. For example, the down-regulation of NR1H3, AKR1C3, and HAVCR1, and the up-regulation of IGFBP2, GAS1, and BMP2, indicate a prediction of future diabetic nephropathy. Up-regulation of MMP9, IGFBP5, CDH6, and NEFM, and down-regulation of FOSL1, NOTCH2, and many others, contributed toward tumors associated with the urinary tract and hydronephrosis. The up-/down-regulation of many other genes (ITGA1, CXCL12, POSTN etc.) contributes to renal-associated function, such as adhesion of kidney cells, migration of kidney cell lines, and cell movement of kidney cell lines.

## Supporting Information

S1 Fig Clonogenic potential of RCC cell lines under different serum concentration and normoxic and hypoxic condition. (TIF)

S2 Fig Representative image of the Hippo signaling pathway according to the imported list of common genes differentially expressed in the CD105+ cells from primary and metastatic RCC. Red and blue color arrows represent up- and down- regulated genes, respectively.
(TIF)

S1 Table List of up- and down-regulated genes in ASE-5063 (healthy kidney) vs CD105(ACHN) cells. (DOCX)

S2 Table List of up- and down-regulated genes in ASE-5063 (healthy kidney) vs CD105(Caki-2) cells. (DOCX)

S3 Table Common differentially (up-and down-regulated) expressed genes between CD105(Caki-2) and CD105(ACHN). (DOCX)

## Abbreviations

RCC: renal cell carcinoma
SCLCCs: stem cell-like cancer cells
CSCs/TICs: cancer stem cells/tumor initiating cells
MSCs: mesenchymal stem cells
CFE: colony forming efficiency
PCR: polymerase chain reaction
FACS: fluorescence-activated cell sorting
FBS: fetal bovine serum
AP: alkaline phosphatase
PFA: paraformaldehyde
3D: three-dimensional
IPA: Ingenuity Pathway Analysis
IPKB: Ingenuity Pathways Knowledge Base
GO: gene ontology
KEGG: Kyoto Encyclopaedia of Genes and Genomes
mccRCC: metastatic clear cell renal cell carcinoma
EMT: epithelial-mesenchymal transition
IL6: interleukin-6
PFS: progression-free survival
OS: overall survival
PANTHER: Protein ANalysis THrough Evolutionary Relationships
