# Supplementary figures and images for: Comparative Gene Expression Profiling of Primary and Metastatic Renal Cell Carcinoma Stem Cell-Like Cancer Cells
Source: PLoS One. 2016 Nov 3;11(11):e0165718. doi: 10.1371/journal.pone.0165718 (PMC5094751; doi:10.1371/journal.pone.0165718)

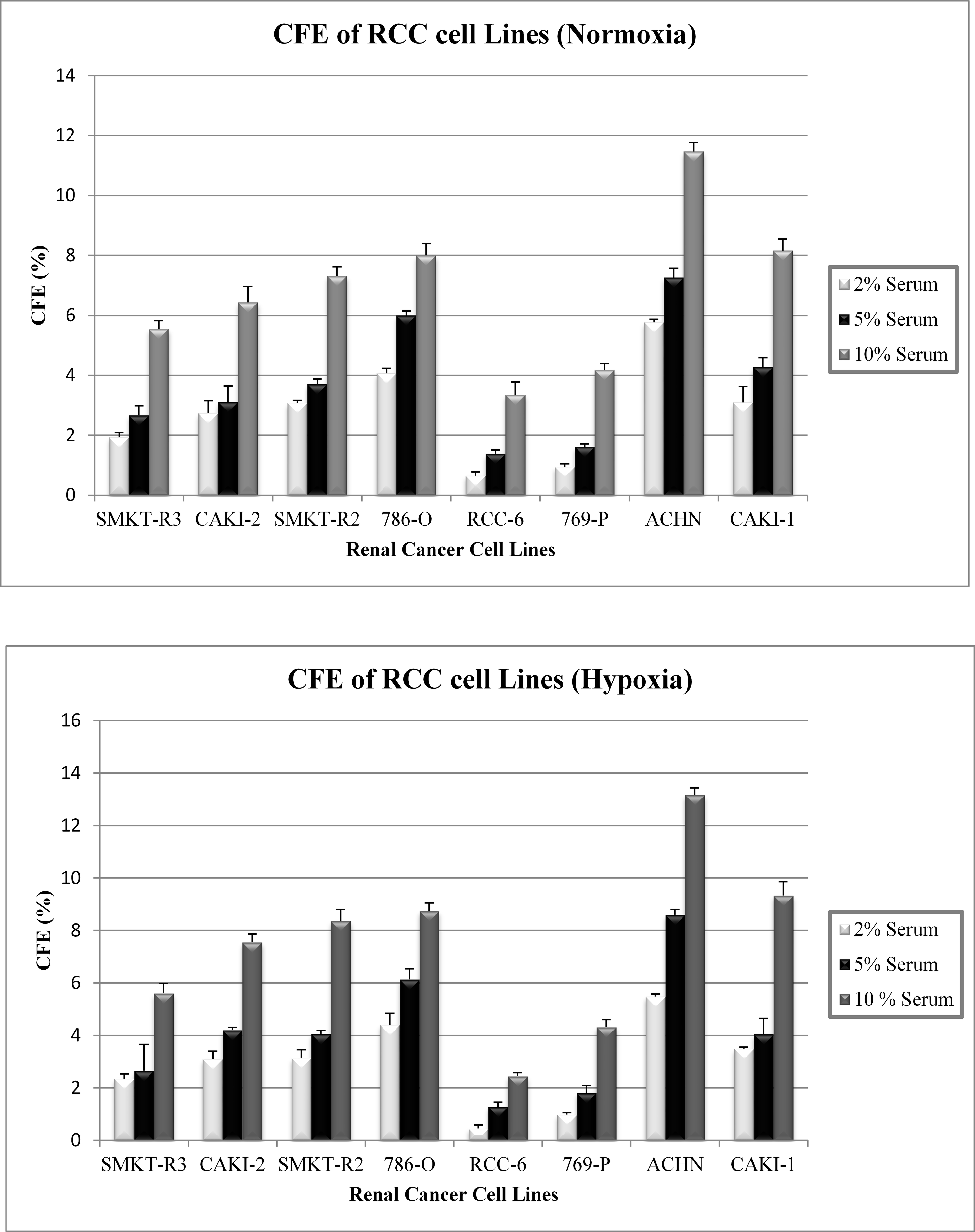

Supplement: S1 Fig — (TIF) [file pone.0165718.s001.tif]

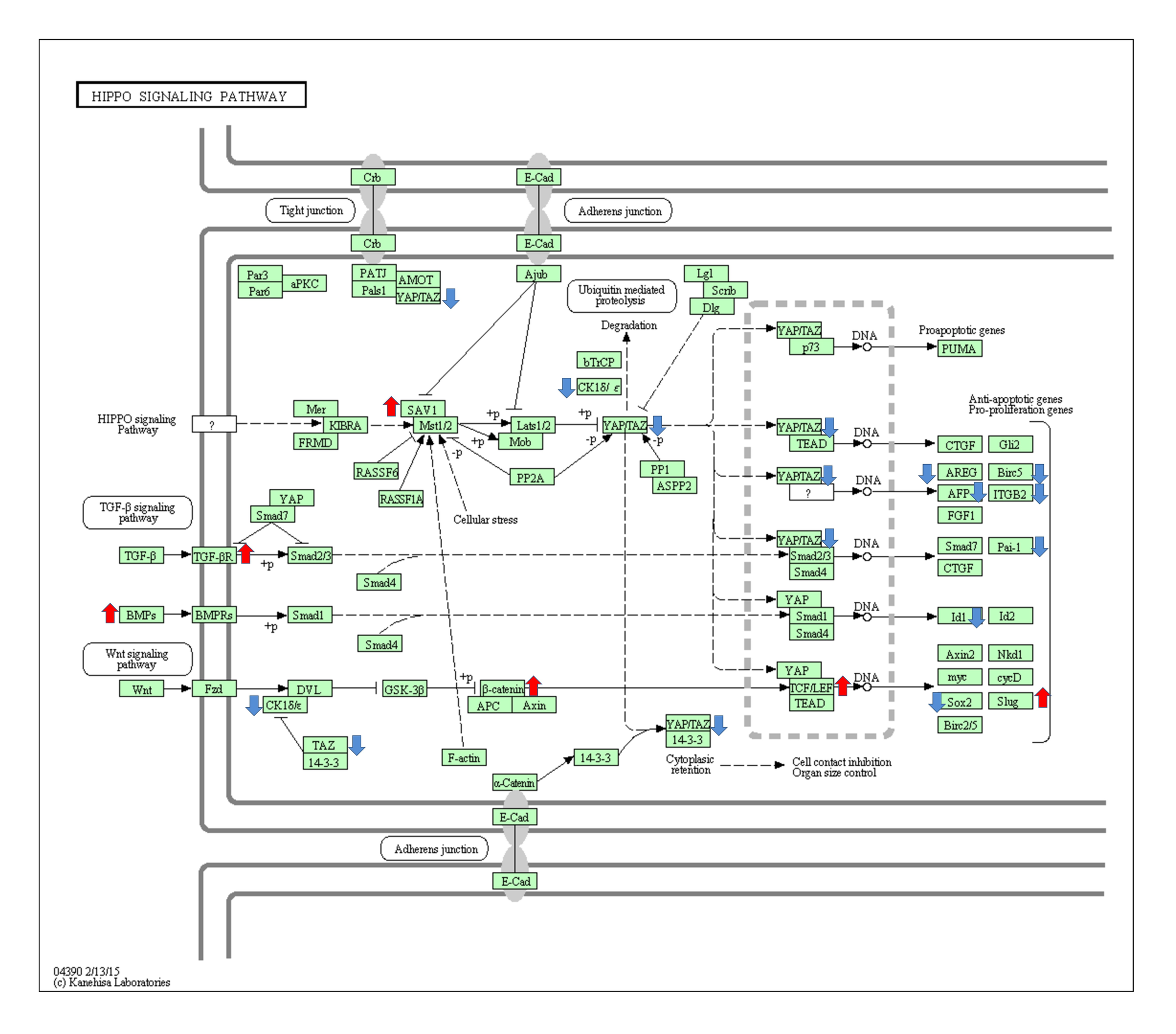

Supplement: S2 Fig — Red and blue color arrows represent up- and down- regulated genes, respectively. (TIF) [file pone.0165718.s002.tif]
