# Supplementary material for: Comparative Gene Expression Profiling of Primary and Metastatic Renal Cell Carcinoma Stem Cell-Like Cancer Cells
Source: PLoS One. 2016 Nov 3;11(11):e0165718. doi: 10.1371/journal.pone.0165718 (PMC5094751; doi:10.1371/journal.pone.0165718)
Supplement: S1 Table — (DOCX) [file pone.0165718.s003.docx]

**Supplementary table S1:** List of up- and down-regulated genes in ASE-5063 (healthy kidney) vs CD105(ACHN) cells.

| GeneSymbol | p | FC (abs) | Regulation |
| --- | --- | --- | --- |
| RGS10 | 8.87E-07 | 2.0026596 | down |
| SATB2 | 1.16E-06 | 2.0027049 | down |
| MIER1 | 1.06E-06 | 2.009342 | down |
| RFX7 | 7.68E-07 | 2.0127137 | down |
| SUMF1 | 9.33E-07 | 2.0154164 | down |
| TMEM101 | 7.54E-07 | 2.019066 | down |
| TMEM41B | 7.15E-07 | 2.0207922 | down |
| ZNF271P | 1.17E-06 | 2.0238779 | down |
| ADRBK2 | 6.09E-07 | 2.025552 | down |
| U2AF1L4 | 1.03E-06 | 2.026633 | down |
| RASSF1 | 6.84E-07 | 2.0316734 | down |
| BOD1L1 | 1.42E-06 | 2.0332294 | down |
| CAMK1D | 8.97E-07 | 2.0343802 | down |
| SIRT5 | 1.41E-06 | 2.0352097 | down |
| C14orf93 | 9.71E-07 | 2.0357275 | down |
| HSD17B7 | 1.24E-06 | 2.0455391 | down |
| DHRS12 | 1.38E-06 | 2.048601 | down |
| FOXN2 | 5.16E-07 | 2.0529897 | down |
| FAM198B | 1.43E-06 | 2.0579028 | down |
| TIMM9 | 1.01E-06 | 2.0580964 | down |
| MRPS18B | 1.07E-06 | 2.0581179 | down |
| MRPL45 | 1.20E-06 | 2.0640018 | down |
| TMEM87A | 9.99E-07 | 2.064846 | down |
| TM2D1 | 1.44E-06 | 2.064968 | down |
| CCDC142 | 1.02E-06 | 2.067007 | down |
| BUB3 | 1.29E-06 | 2.0676236 | down |
| RAB24 | 7.16E-07 | 2.069648 | down |
| STAT6 | 7.32E-07 | 2.0699859 | down |
| PPTC7 | 1.25E-06 | 2.0740483 | down |
| UBE2L6 | 8.30E-07 | 2.0742888 | down |
| ALS2 | 9.38E-07 | 2.0759048 | down |
| C7orf49 | 7.20E-07 | 2.0769212 | down |
| ALG13 | 9.49E-07 | 2.0824523 | down |
| COG7 | 1.17E-06 | 2.082483 | down |
| ZNF271P | 1.46E-06 | 2.0919325 | down |
| SYNGR3 | 9.14E-07 | 2.098936 | down |
| LUC7L3 | 7.00E-07 | 2.101767 | down |
| NEK9 | 7.55E-07 | 2.1021955 | down |
| GNRH1 | 8.63E-07 | 2.1025274 | down |
| SART3 | 7.61E-07 | 2.1074266 | down |
| C1orf123 | 4.13E-07 | 2.1074405 | down |
| JMY | 1.11E-06 | 2.109487 | down |
| SYF2 | 1.19E-06 | 2.11218 | down |
| PTPN12 | 1.05E-06 | 2.1131194 | down |
| CLCN7 | 1.01E-06 | 2.113152 | down |
| PARP8 | 1.31E-06 | 2.1132603 | down |
| NFKBIB | 7.51E-07 | 2.115032 | down |
| AKIP1 | 5.99E-07 | 2.1176066 | down |
| CCDC57 | 4.85E-07 | 2.1194134 | down |
| RPAIN | 1.26E-06 | 2.120836 | down |
| PIM3 | 6.78E-07 | 2.1219301 | down |
| TLCD1 | 1.07E-06 | 2.1219707 | down |
| GUSBP11 | 7.48E-07 | 2.1314204 | down |
| METTL8 | 1.41E-06 | 2.1319172 | down |
| ZNHIT6 | 5.81E-07 | 2.1331027 | down |
| POLL | 9.41E-07 | 2.1339996 | down |
| FAM217B | 8.05E-07 | 2.1343067 | down |
| COX11 | 8.80E-07 | 2.1394312 | down |
| MT1F | 9.93E-07 | 2.1395712 | down |
| CEP192 | 7.03E-07 | 2.140131 | down |
| SPSB2 | 5.03E-07 | 2.143123 | down |
| ZC3HC1 | 1.28E-06 | 2.1436286 | down |
| OGFRL1 | 7.78E-07 | 2.1438046 | down |
| RNASEK | 1.00E-06 | 2.1439276 | down |
| FAM76A | 1.18E-06 | 2.1443667 | down |
| C10orf2 | 1.26E-06 | 2.1443744 | down |
| SUCO | 1.05E-06 | 2.1481466 | down |
| C1orf43 | 4.39E-07 | 2.1521454 | down |
| FDX1 | 5.41E-07 | 2.1537523 | down |
| C1GALT1 | 8.26E-07 | 2.1552138 | down |
| R3HDM2 | 9.10E-07 | 2.1578996 | down |
| SAMD12 | 9.96E-07 | 2.1614172 | down |
| ZEB1 | 6.40E-07 | 2.162674 | down |
| DDX55 | 1.12E-06 | 2.1626878 | down |
| MRM1 | 6.73E-07 | 2.1645637 | down |
| PRIM1 | 5.59E-07 | 2.1647704 | down |
| NDUFA5 | 6.81E-07 | 2.1677163 | down |
| ZNF830 | 7.55E-07 | 2.1690001 | down |
| ORC5 | 6.91E-07 | 2.1711235 | down |
| SNTB1 | 5.98E-07 | 2.1808164 | down |
| HLA-DPB1 | 9.89E-07 | 2.1843073 | down |
| PHF14 | 8.51E-07 | 2.1851995 | down |
| RHOQ | 5.30E-07 | 2.1869557 | down |
| ENOX2 | 3.77E-07 | 2.1891687 | down |
| TMEM60 | 8.45E-07 | 2.19656 | down |
| TCEAL4 | 3.65E-07 | 2.1965988 | down |
| TMEM98 | 5.69E-07 | 2.1974936 | down |
| ZNF394 | 7.52E-07 | 2.2028644 | down |
| CTSO | 4.28E-07 | 2.2066324 | down |
| NUP107 | 1.28E-06 | 2.2072246 | down |
| CCDC169 | 1.17E-06 | 2.2078364 | down |
| RPGR | 1.06E-06 | 2.213803 | down |
| DIEXF | 1.23E-06 | 2.2145662 | down |
| SLC4A1AP | 9.57E-07 | 2.2147439 | down |
| C1GALT1 | 7.71E-07 | 2.2153292 | down |
| SULF2 | 1.26E-06 | 2.215531 | down |
| CHML | 9.99E-07 | 2.2158935 | down |
| N4BP2L2 | 3.38E-07 | 2.2281725 | down |
| TRIAP1 | 4.42E-07 | 2.229923 | down |
| ATRAID | 9.95E-07 | 2.2311532 | down |
| SERTAD4 | 1.12E-06 | 2.2313168 | down |
| ERCC3 | 6.82E-07 | 2.2313495 | down |
| STAG3L4 | 1.14E-06 | 2.2319274 | down |
| RHPN2 | 6.97E-07 | 2.233587 | down |
| CEP135 | 5.21E-07 | 2.2382774 | down |
| TMEM218 | 1.24E-06 | 2.2421782 | down |
| CCDC115 | 6.21E-07 | 2.2434115 | down |
| FAM134B | 9.89E-07 | 2.2451348 | down |
| RASAL2 | 3.03E-07 | 2.2552476 | down |
| SPPL3 | 7.55E-07 | 2.2560692 | down |
| RAB3GAP2 | 6.68E-07 | 2.2561727 | down |
| PHC3 | 5.17E-07 | 2.2564783 | down |
| MRPL33 | 5.89E-07 | 2.2566307 | down |
| OSCP1 | 1.44E-06 | 2.2569299 | down |
| ZNF12 | 1.11E-06 | 2.2586129 | down |
| DARS | 7.56E-07 | 2.25921 | down |
| HPS5 | 7.26E-07 | 2.2609587 | down |
| SNX29 | 1.03E-06 | 2.2640843 | down |
| ARHGAP32 | 1.31E-06 | 2.2696133 | down |
| MTERF4 | 7.35E-07 | 2.2707944 | down |
| MAFG-AS1 | 4.06E-07 | 2.2731557 | down |
| NUDT4 | 1.31E-06 | 2.2744625 | down |
| ATXN7L1 | 4.50E-07 | 2.2793775 | down |
| KIAA0753 | 9.59E-07 | 2.282365 | down |
| UNC119B | 1.31E-06 | 2.2860637 | down |
| C7orf73 | 5.60E-07 | 2.2879627 | down |
| BMP2K | 1.25E-06 | 2.2900589 | down |
| FAM107B | 7.91E-07 | 2.2927327 | down |
| ANKZF1 | 1.37E-06 | 2.2928977 | down |
| RAB13 | 6.33E-07 | 2.2948616 | down |
| KRTAP19-8 | 5.20E-07 | 2.2986858 | down |
| RSL1D1 | 9.92E-07 | 2.2997546 | down |
| NEMF | 3.46E-07 | 2.301061 | down |
| AMZ2P1 | 7.29E-07 | 2.3020935 | down |
| EPHX2 | 2.82E-07 | 2.3026352 | down |
| ZNF594 | 5.19E-07 | 2.3077583 | down |
| ADM2 | 4.11E-07 | 2.312137 | down |
| EML4 | 1.27E-06 | 2.3122635 | down |
| CYLD | 5.39E-07 | 2.3127873 | down |
| EIF4A2 | 7.62E-07 | 2.3129146 | down |
| KIF26B | 8.73E-07 | 2.3130617 | down |
| MPDZ | 1.05E-06 | 2.3151662 | down |
| CTTNBP2 | 1.03E-06 | 2.3172364 | down |
| LRRC37BP1 | 6.40E-07 | 2.3185024 | down |
| MSMP | 9.52E-07 | 2.3189893 | down |
| ATP5G2 | 3.58E-07 | 2.3195167 | down |
| ALDH3B1 | 9.49E-07 | 2.3218772 | down |
| OARD1 | 3.39E-07 | 2.328253 | down |
| UNC5B-AS1 | 8.71E-07 | 2.3292258 | down |
| RNF182 | 1.30E-06 | 2.3314378 | down |
| APAF1 | 4.62E-07 | 2.3353145 | down |
| CEP295 | 6.67E-07 | 2.3358452 | down |
| GCNT2 | 4.78E-07 | 2.336855 | down |
| SLC9A5 | 1.10E-06 | 2.3380582 | down |
| ZNF605 | 3.30E-07 | 2.3382463 | down |
| ZFP36 | 7.58E-07 | 2.3383653 | down |
| BDH2 | 7.58E-07 | 2.343798 | down |
| PPIL3 | 2.01E-07 | 2.3442822 | down |
| LRRC37A3 | 6.21E-07 | 2.3477843 | down |
| SIK3 | 8.29E-07 | 2.3531559 | down |
| PLEKHB2 | 6.23E-07 | 2.3539467 | down |
| NUBPL | 5.20E-07 | 2.3546891 | down |
| CHCHD3 | 1.10E-06 | 2.357279 | down |
| MNT | 5.10E-07 | 2.3590758 | down |
| ZNF37A | 4.34E-07 | 2.3591511 | down |
| RCOR3 | 5.99E-07 | 2.3632522 | down |
| SBDS | 5.74E-07 | 2.3643372 | down |
| LINC01061 | 1.34E-06 | 2.3650534 | down |
| ADAT2 | 1.30E-06 | 2.3663468 | down |
| CRNDE | 1.16E-06 | 2.370482 | down |
| SMAD3 | 8.46E-07 | 2.3720098 | down |
| HOXC10 | 1.09E-06 | 2.3728766 | down |
| GMPR | 6.54E-07 | 2.3750496 | down |
| RPL22 | 2.50E-07 | 2.3761048 | down |
| COPA | 5.31E-07 | 2.3772275 | down |
| FAM149A | 5.58E-07 | 2.3794203 | down |
| PSIP1 | 1.30E-06 | 2.384781 | down |
| ANXA7 | 1.13E-06 | 2.3877573 | down |
| KLHDC4 | 6.65E-07 | 2.3901024 | down |
| SPPL3 | 2.58E-07 | 2.3902774 | down |
| PUS7 | 4.92E-07 | 2.39029 | down |
| SNHG8 | 9.43E-07 | 2.3910227 | down |
| EGR1 | 2.29E-07 | 2.3923068 | down |
| NLRC5 | 7.97E-07 | 2.394491 | down |
| KIAA0408 | 3.60E-07 | 2.3947024 | down |
| IL6ST | 3.98E-07 | 2.3962193 | down |
| GLS | 7.42E-07 | 2.3965895 | down |
| SPIRE1 | 1.30E-06 | 2.40062 | down |
| NDFIP2 | 5.91E-07 | 2.4028246 | down |
| MUT | 4.49E-07 | 2.4073117 | down |
| UPP1 | 1.31E-06 | 2.4073899 | down |
| SLC35G2 | 4.96E-07 | 2.4082239 | down |
| ATM | 7.85E-07 | 2.4118204 | down |
| LPAR1 | 5.25E-07 | 2.4125886 | down |
| DENND5B | 1.97E-07 | 2.4167743 | down |
| GLTSCR2 | 4.55E-07 | 2.419076 | down |
| MT1M | 5.36E-07 | 2.4194508 | down |
| OMA1 | 1.20E-06 | 2.420247 | down |
|  | 7.41E-07 | 2.427216 | down |
| MBD5 | 6.42E-07 | 2.4300754 | down |
| HTATIP2 | 4.06E-07 | 2.430165 | down |
| NT5C3A | 9.31E-07 | 2.4360924 | down |
| TATDN3 | 7.79E-07 | 2.4390275 | down |
| GPX8 | 2.62E-07 | 2.4391694 | down |
| CCDC14 | 2.20E-07 | 2.4399724 | down |
| LAPTM4A | 2.31E-07 | 2.4435618 | down |
| SPR | 9.28E-07 | 2.4441562 | down |
| TRIM45 | 8.00E-07 | 2.4508893 | down |
| LOC84214 | 3.56E-07 | 2.4524043 | down |
| ZFP36L1 | 1.34E-06 | 2.454215 | down |
| TCAIM | 1.20E-06 | 2.4551473 | down |
| CCZ1 | 9.89E-07 | 2.456554 | down |
| QPCT | 5.58E-07 | 2.4575903 | down |
| DHFRL1 | 3.54E-07 | 2.4577072 | down |
| TCHP | 2.61E-07 | 2.4590955 | down |
| PCBD1 | 1.09E-06 | 2.459206 | down |
| SMYD4 | 4.43E-07 | 2.4593341 | down |
| ZCCHC6 | 1.18E-06 | 2.4595013 | down |
| CLUAP1 | 9.28E-07 | 2.4603953 | down |
| PPP1R15B | 5.44E-07 | 2.4612694 | down |
| SP110 | 8.72E-07 | 2.4623635 | down |
| PTPN11 | 1.21E-06 | 2.4647899 | down |
| RNF213 | 1.34E-06 | 2.4649167 | down |
| STK32B | 8.72E-07 | 2.4650116 | down |
| RAB28 | 1.03E-06 | 2.4680605 | down |
| PPT1 | 5.19E-07 | 2.4686677 | down |
| PCF11 | 6.59E-07 | 2.4696732 | down |
| CXCL16 | 5.75E-07 | 2.4710653 | down |
| ATMIN | 9.92E-07 | 2.4733179 | down |
| COA6 | 4.24E-07 | 2.4772127 | down |
| WDFY3 | 6.14E-07 | 2.4773686 | down |
| TTC32 | 8.39E-07 | 2.4776883 | down |
| SH2B2 | 8.15E-07 | 2.4789503 | down |
| IQCD | 8.51E-07 | 2.4797602 | down |
| SPOP | 1.12E-06 | 2.4804335 | down |
| ARL4A | 3.11E-07 | 2.482018 | down |
| EXOC6 | 5.06E-07 | 2.48267 | down |
| ECI1 | 1.04E-06 | 2.4838815 | down |
| HKDC1 | 4.56E-07 | 2.4846523 | down |
| MRPS33 | 8.54E-07 | 2.4893715 | down |
| CEP57 | 1.32E-06 | 2.4902136 | down |
| RAB31 | 1.94E-07 | 2.4923556 | down |
| MDM4 | 9.14E-07 | 2.4961135 | down |
| TRADD | 6.91E-07 | 2.4963818 | down |
| PVT1 | 1.35E-06 | 2.4964302 | down |
| FAM213A | 2.99E-07 | 2.4975486 | down |
| IFNAR2 | 5.86E-07 | 2.5015655 | down |
| POLL | 2.24E-07 | 2.505543 | down |
| FDXR | 3.64E-07 | 2.5062284 | down |
| RASAL2 | 2.03E-07 | 2.5080225 | down |
| RRP15 | 8.05E-07 | 2.5104303 | down |
| ZNF512 | 1.03E-06 | 2.511846 | down |
| CAPG | 9.33E-07 | 2.5120707 | down |
| TOMM7 | 6.69E-07 | 2.5153592 | down |
| CYB5D2 | 3.74E-07 | 2.5156574 | down |
| PAG1 | 9.40E-07 | 2.5161047 | down |
| PMS2 | 1.18E-06 | 2.5166488 | down |
| WDR59 | 5.53E-07 | 2.5178473 | down |
| DBNDD2 | 7.15E-07 | 2.518014 | down |
| TRIM6 | 5.45E-07 | 2.5207133 | down |
| ACP1 | 1.27E-06 | 2.5208824 | down |
| RALGPS2 | 3.92E-07 | 2.5236573 | down |
| KANK1 | 8.86E-07 | 2.524903 | down |
| C17orf67 | 9.66E-07 | 2.5260406 | down |
| LRP6 | 2.52E-07 | 2.5283728 | down |
| NEURL2 | 1.10E-06 | 2.5286033 | down |
| ADAM10 | 1.91E-07 | 2.5296829 | down |
| TARBP1 | 4.23E-07 | 2.5300748 | down |
| ATP1A1-AS1 | 1.20E-06 | 2.5303118 | down |
| SDSL | 4.86E-07 | 2.5311122 | down |
| TMEM117 | 2.96E-07 | 2.5332682 | down |
| GRB10 | 1.42E-07 | 2.5361195 | down |
| DSG2 | 6.62E-07 | 2.5372317 | down |
| CTBS | 1.07E-06 | 2.5377233 | down |
| HEXDC | 5.34E-07 | 2.545257 | down |
| ZAK | 1.89E-07 | 2.5494652 | down |
| FBXW8 | 1.03E-06 | 2.5496335 | down |
| GAS8 | 6.11E-07 | 2.5523598 | down |
| ATG16L1 | 5.85E-07 | 2.5558407 | down |
| C7orf13 | 3.49E-07 | 2.5566564 | down |
| GULP1 | 9.38E-07 | 2.5598896 | down |
| CLUAP1 | 3.96E-07 | 2.5599208 | down |
| PRPF38B | 5.36E-07 | 2.5600662 | down |
| CREB3L4 | 4.49E-07 | 2.560407 | down |
| RRNAD1 | 4.82E-07 | 2.5605452 | down |
| TBC1D2 | 4.45E-07 | 2.5612104 | down |
| GLTSCR2 | 7.53E-07 | 2.5629113 | down |
| PHF14 | 8.29E-07 | 2.5646212 | down |
| ING3 | 4.90E-07 | 2.5674968 | down |
| MCTP1 | 1.18E-06 | 2.5689876 | down |
| TMEM37 | 3.06E-07 | 2.5693471 | down |
| TMEM242 | 3.00E-07 | 2.5694304 | down |
| ENKD1 | 6.46E-07 | 2.5721238 | down |
| TRIM4 | 3.82E-07 | 2.5722888 | down |
| DBNL | 8.65E-07 | 2.573869 | down |
| FAM175A | 2.06E-07 | 2.5745208 | down |
| MTERF1 | 4.44E-07 | 2.5754461 | down |
| GPRC5C | 5.14E-07 | 2.5757816 | down |
| LAMC3 | 5.05E-07 | 2.577072 | down |
| IDUA | 4.78E-07 | 2.5774097 | down |
| MAMDC4 | 4.87E-07 | 2.5789585 | down |
| LOC102723652 | 1.47E-07 | 2.5790012 | down |
| PIR | 8.06E-07 | 2.5802946 | down |
| LOC81691 | 3.80E-07 | 2.5811164 | down |
| NFYB | 2.32E-07 | 2.581488 | down |
| NAT9 | 4.98E-07 | 2.5839674 | down |
| C2orf81 | 1.27E-06 | 2.5851073 | down |
| FAM102A | 9.26E-07 | 2.5864499 | down |
| SNAPC5 | 2.06E-07 | 2.588189 | down |
| SNAPC5 | 2.95E-07 | 2.59015 | down |
| SP140 | 5.67E-07 | 2.5925255 | down |
| MXD1 | 2.19E-07 | 2.5927238 | down |
| NPIPB5 | 1.37E-06 | 2.5976148 | down |
| HIST2H2BF | 7.73E-07 | 2.5976443 | down |
| RIMKLA | 2.30E-07 | 2.598563 | down |
| FAM90A1 | 4.04E-07 | 2.5992067 | down |
| SH3BGR | 8.48E-07 | 2.599298 | down |
| TWF1 | 6.02E-07 | 2.601414 | down |
| LOC100132891 | 1.47E-07 | 2.604176 | down |
| UPP1 | 2.37E-07 | 2.6065435 | down |
| GADD45A | 4.04E-07 | 2.608665 | down |
| LOC100506639 | 1.72E-07 | 2.6120377 | down |
| LOH12CR2 | 4.87E-07 | 2.6126409 | down |
| TLR1 | 3.22E-07 | 2.613111 | down |
| DISP1 | 1.34E-06 | 2.614134 | down |
| KCNS3 | 4.15E-07 | 2.6160805 | down |
| SP100 | 2.69E-07 | 2.6163292 | down |
| TRMT13 | 1.43E-06 | 2.6173334 | down |
| U2SURP | 1.44E-06 | 2.6174917 | down |
| DNM1L | 1.97E-07 | 2.6182675 | down |
| CYP2U1 | 2.75E-07 | 2.61836 | down |
| TMEM254 | 4.50E-07 | 2.6186764 | down |
| KLHL12 | 5.04E-07 | 2.6190896 | down |
| AKT3 | 3.31E-07 | 2.6195576 | down |
| CYB561 | 2.42E-07 | 2.623159 | down |
| FAN1 | 2.99E-07 | 2.6234238 | down |
| MET | 5.53E-07 | 2.6259527 | down |
| VEZT | 9.83E-07 | 2.6282036 | down |
| ARHGEF19 | 4.04E-07 | 2.6286006 | down |
| D2HGDH | 2.34E-07 | 2.6289077 | down |
| TCEANC2 | 2.40E-07 | 2.6290576 | down |
| EIF3J-AS1 | 2.48E-07 | 2.6304188 | down |
| PPWD1 | 2.87E-07 | 2.632367 | down |
| MANBA | 6.91E-07 | 2.6352894 | down |
| BRICD5 | 3.44E-07 | 2.6393561 | down |
| SHMT2 | 6.33E-07 | 2.6405547 | down |
| PNPLA4 | 3.30E-07 | 2.6418908 | down |
| ETNK1 | 3.20E-07 | 2.6443079 | down |
| MYLIP | 3.69E-07 | 2.6447043 | down |
| CFAP36 | 1.59E-07 | 2.6483529 | down |
| IFT172 | 4.50E-07 | 2.650754 | down |
| FAM89A | 2.13E-07 | 2.6529098 | down |
| RMI2 | 3.15E-07 | 2.658958 | down |
| TAPBPL | 3.67E-07 | 2.660514 | down |
| SLC30A6 | 6.14E-07 | 2.6657095 | down |
| SLC38A2 | 7.54E-07 | 2.6669517 | down |
| AUH | 1.08E-06 | 2.6697407 | down |
| EP400 | 1.22E-06 | 2.6725242 | down |
| EIF5 | 5.99E-07 | 2.675349 | down |
| ZCCHC11 | 3.72E-07 | 2.6757474 | down |
| GXYLT1P3 | 1.32E-06 | 2.67754 | down |
| SEL1L | 1.07E-07 | 2.6800117 | down |
| PEX3 | 9.45E-07 | 2.6833568 | down |
| TGOLN2 | 7.44E-07 | 2.684316 | down |
| TMED5 | 4.45E-07 | 2.6855004 | down |
| ADAM17 | 1.31E-06 | 2.6872904 | down |
| TAF1D | 2.67E-07 | 2.6873832 | down |
| PPIG | 3.46E-07 | 2.6892207 | down |
| MGMT | 1.86E-07 | 2.691011 | down |
| MBD6 | 3.26E-07 | 2.6920714 | down |
| BBS10 | 9.50E-07 | 2.6935644 | down |
| RAB11FIP1 | 9.37E-07 | 2.6973486 | down |
| YTHDC2 | 1.26E-06 | 2.7000158 | down |
| WHAMM | 2.19E-07 | 2.70014 | down |
| EPOR | 3.09E-07 | 2.70157 | down |
| H2AFJ | 1.50E-07 | 2.7018871 | down |
| TRMT10B | 8.39E-07 | 2.7020109 | down |
| ACSL3 | 7.37E-07 | 2.7041574 | down |
| IRAK2 | 2.31E-07 | 2.7060735 | down |
| ZNF721 | 3.07E-07 | 2.7061906 | down |
| PRKAB2 | 1.93E-07 | 2.7079413 | down |
| GNPTG | 1.10E-07 | 2.7104182 | down |
| FAM21C | 1.10E-07 | 2.7125206 | down |
| NAP1L1 | 1.77E-07 | 2.7141783 | down |
| HOXB4 | 1.12E-07 | 2.714363 | down |
| C1RL | 1.09E-06 | 2.7157419 | down |
| PHLDA3 | 2.58E-07 | 2.7164083 | down |
| ATRX | 7.09E-07 | 2.716793 | down |
| WDR11 | 1.30E-06 | 2.71686 | down |
| ASB9 | 1.35E-07 | 2.7176237 | down |
| ZNF638 | 1.07E-06 | 2.7182853 | down |
| RAVER2 | 5.33E-07 | 2.7186651 | down |
| CNNM4 | 7.72E-07 | 2.718987 | down |
| NXN | 2.00E-07 | 2.7196786 | down |
| FAM60A | 5.34E-07 | 2.7231882 | down |
| TSPAN1 | 8.35E-08 | 2.7238193 | down |
| DNAJB9 | 3.09E-07 | 2.7313735 | down |
| ARL1 | 2.79E-07 | 2.733379 | down |
| TRAF3IP1 | 1.29E-06 | 2.7355657 | down |
| CXorf57 | 4.64E-07 | 2.7364228 | down |
| ZFAS1 | 2.81E-07 | 2.7421117 | down |
| TRAK1 | 1.19E-07 | 2.7449837 | down |
| ST7L | 4.81E-07 | 2.745134 | down |
| SLC35E3 | 5.69E-07 | 2.7464092 | down |
| HIBADH | 4.05E-07 | 2.7472684 | down |
| PNRC1 | 1.43E-07 | 2.7490714 | down |
| ASAH1 | 1.06E-07 | 2.7511408 | down |
| GEMIN8P4 | 1.03E-06 | 2.7513318 | down |
| DEK | 8.58E-07 | 2.751709 | down |
| TK2 | 1.11E-07 | 2.75193 | down |
| DCAF17 | 1.19E-06 | 2.7551265 | down |
| PLCB4 | 5.70E-07 | 2.7569792 | down |
| LIPA | 3.78E-07 | 2.7581372 | down |
| MIA3 | 7.69E-07 | 2.7601712 | down |
| IFNAR2 | 1.88E-07 | 2.7607408 | down |
| C17orf82 | 9.36E-07 | 2.7610607 | down |
| PPM1D | 1.11E-07 | 2.7614012 | down |
| LRIG1 | 1.38E-06 | 2.76753 | down |
| HECTD4 | 5.70E-07 | 2.7683084 | down |
| IRF2BP2 | 1.75E-07 | 2.7683449 | down |
| FLJ31715 | 9.37E-07 | 2.772038 | down |
| GOT1 | 2.42E-07 | 2.7724128 | down |
| ZNF664 | 3.38E-07 | 2.7729475 | down |
| CDK19 | 2.43E-07 | 2.7769382 | down |
| SSB | 1.15E-07 | 2.7774222 | down |
| CEP83 | 1.20E-06 | 2.7780483 | down |
| ORAI3 | 2.32E-07 | 2.7824488 | down |
| DZIP1L | 4.82E-07 | 2.7843704 | down |
| UTP14C | 4.82E-07 | 2.7844293 | down |
| KMT2C | 1.09E-06 | 2.7881339 | down |
|  | 3.01E-07 | 2.788472 | down |
| ANKRD28 | 5.25E-07 | 2.7886653 | down |
| COL27A1 | 1.15E-06 | 2.789119 | down |
| NBR1 | 9.53E-08 | 2.789216 | down |
| OBFC1 | 2.12E-07 | 2.7896445 | down |
|  | 3.61E-07 | 2.7904897 | down |
| PIH1D2 | 1.84E-07 | 2.7907622 | down |
| FAM117B | 2.81E-07 | 2.792722 | down |
| POLG2 | 2.06E-07 | 2.7962213 | down |
| THUMPD1 | 1.39E-06 | 2.7973335 | down |
| C12orf76 | 1.62E-07 | 2.7978501 | down |
| CDCA7L | 1.19E-07 | 2.8016715 | down |
| BEX2 | 7.99E-08 | 2.801838 | down |
| KIAA1109 | 1.97E-07 | 2.8030248 | down |
| PIBF1 | 6.47E-07 | 2.8058498 | down |
| MYO5B | 1.43E-07 | 2.8059762 | down |
| MOAP1 | 1.37E-06 | 2.8075445 | down |
| PMS2 | 2.36E-07 | 2.8081849 | down |
| POLR3B | 3.18E-07 | 2.8128922 | down |
| ZDHHC1 | 3.27E-07 | 2.8153663 | down |
| RCBTB1 | 2.44E-07 | 2.8158035 | down |
| LPHN2 | 2.60E-07 | 2.8179283 | down |
| WHAMM | 2.13E-07 | 2.818075 | down |
| PRRG1 | 5.93E-07 | 2.8199978 | down |
| MTMR10 | 7.33E-07 | 2.8222373 | down |
| PDXDC2P | 3.17E-07 | 2.8225954 | down |
| ZC3H11A | 1.04E-06 | 2.823307 | down |
| MAP3K1 | 1.62E-07 | 2.82386 | down |
| TRAM1 | 2.04E-07 | 2.8245533 | down |
| CASP8AP2 | 5.28E-07 | 2.8292947 | down |
| MAFF | 6.96E-08 | 2.8338675 | down |
| METTL21A | 8.22E-07 | 2.835927 | down |
| IGF2 | 4.33E-07 | 2.8365912 | down |
| C17orf80 | 1.07E-06 | 2.8368502 | down |
| DCLRE1A | 2.36E-07 | 2.8369331 | down |
| EZH1 | 1.61E-07 | 2.8383641 | down |
| MKRN1 | 4.57E-07 | 2.8387742 | down |
| NMI | 1.21E-06 | 2.8409894 | down |
|  | 7.25E-07 | 2.8412204 | down |
| AEBP2 | 9.14E-07 | 2.841678 | down |
| CTIF | 9.29E-07 | 2.8425605 | down |
| RHOT1 | 1.11E-06 | 2.8432212 | down |
| NOVA1 | 1.81E-07 | 2.8439002 | down |
| AFTPH | 1.83E-07 | 2.8440466 | down |
|  | 1.03E-06 | 2.8484128 | down |
| TSGA10 | 6.21E-07 | 2.852274 | down |
| MTIF2 | 1.73E-07 | 2.8543777 | down |
| OLFML2A | 1.21E-06 | 2.854577 | down |
| GUSB | 9.76E-08 | 2.8568583 | down |
| HERPUD2 | 9.45E-07 | 2.857579 | down |
| PIGV | 3.15E-07 | 2.8579316 | down |
| INTU | 7.62E-07 | 2.8626113 | down |
| EIF4B | 6.60E-07 | 2.8645716 | down |
| PCNXL2 | 2.01E-07 | 2.864658 | down |
| C2CD2 | 6.56E-07 | 2.8694065 | down |
| PRR4 | 3.45E-07 | 2.8695939 | down |
| ACAP2 | 9.18E-07 | 2.8699656 | down |
| AFTPH | 7.97E-07 | 2.8701015 | down |
| IMPA2 | 2.91E-07 | 2.8715763 | down |
| SLC25A37 | 1.04E-07 | 2.875416 | down |
| FRA10AC1 | 9.63E-07 | 2.8776019 | down |
| FBXL16 | 7.62E-07 | 2.877874 | down |
| LOC441455 | 2.28E-07 | 2.8790488 | down |
| PCM1 | 1.94E-07 | 2.879563 | down |
| PARP6 | 3.34E-07 | 2.880674 | down |
| ACSL1 | 9.03E-07 | 2.8833656 | down |
| KRT8 | 1.80E-07 | 2.8859987 | down |
| PDXDC2P | 8.02E-07 | 2.8874254 | down |
| CCL2 | 1.25E-07 | 2.8892503 | down |
| TRPC3 | 4.55E-07 | 2.8900554 | down |
| ARHGAP18 | 1.28E-06 | 2.8902025 | down |
| CDKL3 | 3.24E-07 | 2.8903458 | down |
| SPAG16 | 4.39E-07 | 2.8911834 | down |
| AVPI1 | 3.31E-07 | 2.8939507 | down |
| TTC39A | 3.08E-07 | 2.897109 | down |
| MPZL2 | 3.61E-07 | 2.904044 | down |
| PHGDH | 4.39E-07 | 2.9057825 | down |
| ZC2HC1C | 5.40E-07 | 2.9066803 | down |
| LINC01000 | 4.43E-07 | 2.9070027 | down |
| FAS | 5.40E-07 | 2.9114456 | down |
| CCDC84 | 1.27E-07 | 2.913261 | down |
| DBP | 4.75E-07 | 2.9155815 | down |
| ZNF280D | 1.46E-06 | 2.9183874 | down |
| LOC441081 | 7.89E-07 | 2.9205115 | down |
| GFRA1 | 3.28E-07 | 2.9222836 | down |
| NFYC-AS1 | 5.90E-07 | 2.9239235 | down |
| REEP5 | 2.68E-07 | 2.9245822 | down |
| LRRFIP1 | 6.45E-07 | 2.9257126 | down |
| SETD6 | 3.36E-07 | 2.9268756 | down |
| PFKM | 1.41E-07 | 2.9275935 | down |
| UTRN | 1.12E-07 | 2.9286578 | down |
| ZC3H11A | 2.30E-07 | 2.9302042 | down |
| KLRC4 | 4.38E-07 | 2.9311118 | down |
| DBIL5P | 1.17E-06 | 2.931389 | down |
| NPAS1 | 7.90E-07 | 2.9339988 | down |
| RHOF | 1.32E-06 | 2.9345229 | down |
| AJUBA | 5.33E-07 | 2.934644 | down |
| NPC1 | 1.46E-06 | 2.93527 | down |
| LINC00847 | 2.75E-07 | 2.9385111 | down |
| LOC100129550 | 3.58E-07 | 2.9407256 | down |
| ZBTB20 | 1.56E-07 | 2.9409852 | down |
| TRMT1L | 1.91E-07 | 2.9438317 | down |
| ZSWIM7 | 1.84E-07 | 2.9440298 | down |
| N6AMT1 | 6.70E-07 | 2.9457808 | down |
| BCL2L11 | 6.90E-07 | 2.9479644 | down |
| CCDC148 | 7.23E-07 | 2.9481487 | down |
| HLA-DRB1 | 7.66E-08 | 2.9517908 | down |
| COX15 | 8.35E-08 | 2.9543197 | down |
| MRPL10 | 1.29E-06 | 2.959704 | down |
| KIF17 | 2.38E-07 | 2.9606843 | down |
| IPO9 | 2.10E-07 | 2.9667013 | down |
|  | 1.10E-06 | 2.9677725 | down |
| PLD1 | 5.31E-07 | 2.9683867 | down |
| ANKMY2 | 9.37E-08 | 2.9692795 | down |
| MOGAT1 | 1.11E-06 | 2.9700227 | down |
| WASL | 9.26E-08 | 2.9702985 | down |
| SENP6 | 2.12E-07 | 2.9774039 | down |
| GAA | 2.34E-07 | 2.9788134 | down |
| GLTSCR1L | 1.46E-06 | 2.9844494 | down |
| NT5C2 | 7.11E-07 | 2.9862897 | down |
| FAM107B | 2.88E-07 | 2.9877715 | down |
| VAMP4 | 9.28E-08 | 2.9896736 | down |
| FAM86B3P | 1.07E-07 | 2.989766 | down |
| CWC25 | 5.20E-07 | 2.9899898 | down |
| REV1 | 3.88E-07 | 2.9904087 | down |
| BAIAP2-AS1 | 1.87E-07 | 2.9908555 | down |
| GDF15 | 2.66E-07 | 2.9920907 | down |
| HSD17B8 | 6.11E-08 | 2.992227 | down |
| WDR78 | 1.14E-06 | 2.9924242 | down |
| ATF3 | 2.51E-07 | 2.9938917 | down |
| ADCY7 | 1.19E-06 | 2.994316 | down |
| PAG1 | 7.63E-07 | 2.9951262 | down |
| ITFG2 | 4.41E-07 | 2.9971108 | down |
| BBS4 | 1.22E-07 | 2.9979455 | down |
| UNC119 | 4.61E-07 | 2.9985046 | down |
| GATM | 7.07E-07 | 2.9985938 | down |
| ROCK2 | 4.02E-07 | 3.000032 | down |
| IDNK | 1.92E-07 | 3.0004187 | down |
| SERPINB1 | 2.18E-07 | 3.0005202 | down |
| FAM66A | 5.99E-07 | 3.0012069 | down |
| CASZ1 | 1.14E-06 | 3.0016606 | down |
| ETV1 | 5.71E-07 | 3.002093 | down |
| STAT2 | 6.30E-07 | 3.0032926 | down |
| SCAI | 1.92E-07 | 3.003846 | down |
| LOC728903 | 5.12E-07 | 3.0040584 | down |
| EIF4B | 1.33E-07 | 3.0045645 | down |
| SPATA6 | 1.30E-07 | 3.0054235 | down |
| PTPN13 | 8.22E-07 | 3.0081205 | down |
| KPNA5 | 2.67E-07 | 3.0098968 | down |
| TCTN2 | 6.39E-07 | 3.011117 | down |
| EPRS | 1.77E-07 | 3.0116124 | down |
| CIAO1 | 1.47E-07 | 3.0117857 | down |
| AK8 | 3.42E-07 | 3.0131404 | down |
| HNRNPH3 | 1.88E-07 | 3.0146806 | down |
| SLC11A2 | 4.14E-07 | 3.015655 | down |
| VPS13C | 9.77E-07 | 3.0174656 | down |
| ZNF652 | 6.95E-07 | 3.0210896 | down |
| ABLIM1 | 2.14E-07 | 3.0227761 | down |
| PMS2P1 | 1.60E-07 | 3.0237467 | down |
| SEPHS2 | 8.33E-07 | 3.0244777 | down |
| MAP3K14 | 2.72E-07 | 3.0249195 | down |
| PCGF1 | 5.53E-08 | 3.0293472 | down |
| ICAM2 | 2.84E-07 | 3.0299559 | down |
| CD55 | 6.26E-08 | 3.0312777 | down |
| NOL6 | 4.96E-07 | 3.0317104 | down |
| FAM111A | 9.44E-07 | 3.0339794 | down |
| MUC1 | 3.52E-07 | 3.0349503 | down |
| IMMP2L | 7.02E-08 | 3.0381138 | down |
| SCAF11 | 1.17E-07 | 3.0384612 | down |
| KRBOX4 | 2.30E-07 | 3.0387385 | down |
| KMT2E | 9.22E-07 | 3.0393395 | down |
| CLDN1 | 7.11E-07 | 3.0394416 | down |
| NEIL1 | 2.29E-07 | 3.0400455 | down |
| FCHO2 | 3.45E-07 | 3.0404866 | down |
| CHEK2 | 4.33E-07 | 3.0407014 | down |
| SMEK2 | 7.65E-07 | 3.041086 | down |
| SNX10 | 1.13E-06 | 3.0416243 | down |
| MKRN7P | 7.69E-07 | 3.0438619 | down |
| PBX1 | 3.00E-07 | 3.044785 | down |
| MTURN | 6.79E-07 | 3.0449276 | down |
| TMEM180 | 4.26E-07 | 3.0501437 | down |
| MSRB3 | 1.41E-07 | 3.052008 | down |
| CUX1 | 4.42E-07 | 3.0536673 | down |
| ANTXR1 | 4.21E-07 | 3.0546064 | down |
| AGPAT5 | 9.80E-08 | 3.0556865 | down |
| AMZ1 | 9.81E-07 | 3.0561996 | down |
| ANKRD7 | 3.44E-07 | 3.0570364 | down |
| ZFP90 | 5.98E-08 | 3.0573 | down |
| PRKCQ | 1.80E-07 | 3.0576763 | down |
| EPB41L1 | 6.63E-07 | 3.0593112 | down |
| GP1BB | 1.06E-07 | 3.0599248 | down |
| MTURN | 1.02E-07 | 3.0607004 | down |
| USP31 | 1.39E-07 | 3.0623765 | down |
| DENND4C | 1.45E-06 | 3.0636487 | down |
| ZAK | 6.24E-08 | 3.064053 | down |
| RUFY2 | 3.78E-07 | 3.0660133 | down |
| ATF3 | 1.07E-06 | 3.0703957 | down |
| NF1 | 1.86E-07 | 3.0791209 | down |
| KLHDC9 | 2.08E-07 | 3.0795174 | down |
| GEM | 1.08E-06 | 3.0801306 | down |
| CCDC102A | 6.56E-07 | 3.0837162 | down |
| SGMS1 | 2.13E-07 | 3.0864797 | down |
| NEK3 | 2.92E-07 | 3.086802 | down |
| GALNT18 | 1.46E-07 | 3.0876515 | down |
| SFI1 | 4.89E-08 | 3.088886 | down |
| CLDN12 | 2.96E-07 | 3.0893316 | down |
| SMAD6 | 3.23E-07 | 3.0911283 | down |
| MXI1 | 5.99E-07 | 3.0911465 | down |
| PHF10 | 1.04E-07 | 3.0918992 | down |
| CEP95 | 1.12E-06 | 3.0924475 | down |
| GTF2IRD1 | 9.85E-08 | 3.0964804 | down |
| KCNIP1 | 4.77E-07 | 3.0975797 | down |
| RGPD5 | 2.72E-07 | 3.0985837 | down |
| DNAH11 | 1.13E-06 | 3.099851 | down |
| SLC38A1 | 2.14E-07 | 3.1032135 | down |
| BCAT1 | 1.63E-07 | 3.1065092 | down |
| LOC374443 | 4.06E-07 | 3.110256 | down |
| ALDH3A2 | 8.40E-07 | 3.1106958 | down |
| HELB | 2.02E-07 | 3.1131406 | down |
| EPC2 | 2.34E-07 | 3.1185467 | down |
| FLJ46906 | 5.13E-07 | 3.1190891 | down |
| C10orf32 | 4.08E-07 | 3.1201444 | down |
| RNF6 | 8.08E-07 | 3.1220376 | down |
| C11orf70 | 3.57E-07 | 3.1222396 | down |
| BBIP1 | 1.14E-06 | 3.1227121 | down |
| IKBIP | 4.03E-07 | 3.1233208 | down |
| IRAK1BP1 | 7.43E-08 | 3.127303 | down |
| LOC100129034 | 5.50E-07 | 3.1349132 | down |
| C11orf1 | 2.52E-07 | 3.137425 | down |
| NBPF14 | 5.73E-07 | 3.1377265 | down |
| IFI35 | 5.55E-07 | 3.1393254 | down |
| TMEM38B | 1.93E-07 | 3.1399922 | down |
| TMEM17 | 3.59E-07 | 3.1419659 | down |
| NPTXR | 1.21E-06 | 3.1420252 | down |
| LOC100131257 | 2.50E-07 | 3.1469514 | down |
| NEK1 | 7.00E-07 | 3.1505675 | down |
| TYW1 | 7.92E-07 | 3.152693 | down |
| ADCY3 | 1.46E-07 | 3.1527169 | down |
| TTC30B | 3.23E-07 | 3.1552358 | down |
| MRPL45 | 1.13E-07 | 3.1622372 | down |
| USP40 | 1.34E-06 | 3.1623533 | down |
| TM7SF3 | 2.73E-07 | 3.1691566 | down |
| ZNF84 | 4.59E-07 | 3.1702724 | down |
| CCDC144A | 1.12E-06 | 3.1709273 | down |
| WDSUB1 | 5.59E-07 | 3.1757984 | down |
| C9orf16 | 1.71E-07 | 3.176823 | down |
| DCDC2 | 7.31E-08 | 3.1777427 | down |
| CSNK2A2 | 2.78E-07 | 3.1782928 | down |
| EPB41L4A-AS1 | 7.82E-08 | 3.180147 | down |
| IFT74 | 1.41E-06 | 3.181168 | down |
| CEBPG | 8.41E-07 | 3.1813748 | down |
| CHCHD10 | 1.11E-07 | 3.1821887 | down |
|  | 1.93E-07 | 3.182478 | down |
| PARP12 | 4.40E-07 | 3.1842768 | down |
| PON2 | 8.81E-08 | 3.1848762 | down |
| PRR18 | 2.30E-07 | 3.1851375 | down |
| DOK7 | 1.61E-07 | 3.1866403 | down |
| PNISR | 4.30E-07 | 3.1873229 | down |
| CUL9 | 6.43E-07 | 3.1874099 | down |
| LOC729732 | 2.68E-07 | 3.1901147 | down |
| ARPC4-TTLL3 | 4.82E-07 | 3.1925933 | down |
| KCNN2 | 7.30E-07 | 3.196011 | down |
| NOL3 | 8.67E-08 | 3.196138 | down |
| MIOX | 4.04E-07 | 3.2009897 | down |
| MDK | 3.14E-07 | 3.202939 | down |
| CEP63 | 3.54E-07 | 3.2030175 | down |
| NOM1 | 3.72E-07 | 3.2056658 | down |
| CEBPB | 1.03E-06 | 3.2095602 | down |
| REV3L | 1.57E-07 | 3.209657 | down |
| GADD45G | 4.13E-08 | 3.2100382 | down |
| CSPP1 | 1.27E-07 | 3.2102585 | down |
| EEF1A2 | 9.35E-07 | 3.2142 | down |
| BAZ2B | 7.01E-07 | 3.2163599 | down |
| TNRC6C-AS1 | 7.19E-07 | 3.2164083 | down |
| ADAP2 | 1.56E-07 | 3.2179077 | down |
| SNX13 | 1.21E-07 | 3.2235126 | down |
| SEZ6L2 | 3.23E-07 | 3.2256646 | down |
| TPCN1 | 6.01E-07 | 3.2274406 | down |
| KIRREL3 | 3.83E-07 | 3.227861 | down |
| PPP1R3G | 7.02E-08 | 3.2280672 | down |
| BLOC1S2 | 1.08E-07 | 3.228312 | down |
| BOD1L1 | 1.24E-06 | 3.2291489 | down |
| SCN4B | 1.35E-06 | 3.2293305 | down |
| MXI1 | 1.06E-06 | 3.2293904 | down |
| SAV1 | 9.63E-08 | 3.2309644 | down |
| MBNL2 | 2.89E-07 | 3.2327409 | down |
| NCALD | 1.63E-07 | 3.235843 | down |
| ZC3H12C | 1.80E-07 | 3.2373695 | down |
| ACBD4 | 3.82E-07 | 3.240668 | down |
| ZNF692 | 3.06E-07 | 3.243817 | down |
| RNF149 | 2.39E-07 | 3.2470367 | down |
| WNK1 | 6.60E-07 | 3.249294 | down |
| LRRC37A2 | 8.11E-07 | 3.2494526 | down |
| DPY19L1 | 4.67E-07 | 3.2504923 | down |
| CREBZF | 5.95E-07 | 3.2512372 | down |
| BCAP29 | 6.06E-08 | 3.2530122 | down |
| LINC00909 | 9.15E-07 | 3.2539809 | down |
| TMEM161B | 4.80E-07 | 3.2596993 | down |
| IFNAR1 | 3.26E-07 | 3.261002 | down |
|  | 1.51E-07 | 3.2637815 | down |
| IQCE | 2.80E-07 | 3.264575 | down |
| CASC4 | 3.40E-07 | 3.2646296 | down |
| COA5 | 1.33E-07 | 3.2646837 | down |
| TMEM5 | 6.53E-08 | 3.2669978 | down |
| RASSF6 | 7.77E-07 | 3.2671986 | down |
| C1orf186 | 1.32E-07 | 3.2695818 | down |
| IGFLR1 | 6.82E-08 | 3.2696724 | down |
| RPS6KA3 | 8.73E-07 | 3.2703412 | down |
| ANAPC16 | 1.87E-07 | 3.2703633 | down |
| SETD6 | 1.58E-07 | 3.2719526 | down |
| DMD | 5.26E-08 | 3.2720976 | down |
| BRCA1 | 6.02E-08 | 3.2745762 | down |
| LINC00265 | 3.41E-07 | 3.278331 | down |
| SUPT7L | 1.15E-07 | 3.2797868 | down |
| PPP1R15A | 2.53E-07 | 3.2828221 | down |
| WWC3 | 1.38E-07 | 3.2868006 | down |
| ACADSB | 6.87E-08 | 3.2916465 | down |
| HAUS4 | 1.02E-07 | 3.2925425 | down |
| ABI2 | 1.64E-07 | 3.2941408 | down |
| ZNF768 | 1.15E-06 | 3.2947676 | down |
| DPP9-AS1 | 1.52E-07 | 3.3047688 | down |
| P2RX4 | 2.44E-07 | 3.3077047 | down |
| DNM1L | 6.70E-07 | 3.3104389 | down |
| TRIM55 | 6.00E-07 | 3.3167126 | down |
| ATRX | 2.13E-07 | 3.317653 | down |
| TMF1 | 3.57E-08 | 3.3211915 | down |
| ARAP3 | 1.71E-07 | 3.3227556 | down |
| SLC25A35 | 6.93E-07 | 3.325402 | down |
| ANKRD6 | 8.28E-08 | 3.3266418 | down |
| IER5L | 1.95E-07 | 3.332363 | down |
| PPAP2C | 1.14E-07 | 3.3327 | down |
| CCNL2 | 9.09E-07 | 3.3335147 | down |
| PLGLB1 | 3.54E-08 | 3.3338938 | down |
| TMEM194A | 3.77E-07 | 3.3339264 | down |
| SUMF2 | 1.04E-07 | 3.3425798 | down |
| MPZL1 | 6.02E-08 | 3.3449056 | down |
| ZBTB44 | 1.18E-07 | 3.3452005 | down |
| WHAMMP1 | 9.96E-07 | 3.3461614 | down |
| SPG20 | 1.09E-07 | 3.3486247 | down |
| STAMBPL1 | 2.75E-07 | 3.348862 | down |
| TBK1 | 1.14E-06 | 3.3499184 | down |
| TNFRSF13C | 4.88E-07 | 3.3549294 | down |
| SPNS2 | 9.26E-08 | 3.3598585 | down |
| ZNF19 | 7.62E-08 | 3.3604 | down |
| RADIL | 1.43E-07 | 3.3615413 | down |
| PROS1 | 1.28E-07 | 3.3631647 | down |
| SDK1 | 2.74E-07 | 3.3639915 | down |
| TMEM237 | 8.68E-08 | 3.366996 | down |
| LINC00857 | 4.03E-07 | 3.3672218 | down |
| MEIS3 | 3.11E-07 | 3.3708692 | down |
| SLC6A9 | 3.68E-07 | 3.3713238 | down |
| FAM228B | 1.53E-07 | 3.3724244 | down |
| SLC35A1 | 2.23E-07 | 3.3729076 | down |
| B3GNT9 | 4.54E-07 | 3.3768094 | down |
| C19orf54 | 4.44E-08 | 3.3798802 | down |
| PRKAA2 | 4.34E-08 | 3.3800871 | down |
| ARHGAP4 | 7.41E-07 | 3.3816328 | down |
| TAX1BP1 | 1.11E-07 | 3.3856387 | down |
| CLCN3 | 4.31E-08 | 3.3875818 | down |
| FAS | 3.66E-07 | 3.3880522 | down |
| TPR | 1.06E-06 | 3.3884313 | down |
| APC2 | 3.28E-07 | 3.39082 | down |
| BTN3A1 | 2.63E-07 | 3.3945663 | down |
| SPATA7 | 1.06E-06 | 3.3946064 | down |
| SCRN3 | 1.75E-07 | 3.3960104 | down |
| WDR92 | 1.11E-07 | 3.3966813 | down |
| FANCL | 5.34E-08 | 3.3970337 | down |
| GGTLC1 | 5.59E-08 | 3.3977227 | down |
| VPS41 | 2.91E-07 | 3.3994396 | down |
| KATNAL2 | 3.73E-07 | 3.4002411 | down |
| PMS1 | 5.93E-07 | 3.406091 | down |
| LOC100131564 | 1.37E-07 | 3.4140694 | down |
| ASGR1 | 7.98E-07 | 3.4194865 | down |
| COL12A1 | 1.14E-06 | 3.4248223 | down |
| ZAK | 1.01E-06 | 3.4260263 | down |
| KIFC2 | 1.85E-07 | 3.4267251 | down |
| VLDLR | 8.80E-08 | 3.4288845 | down |
| GGTLC2 | 7.76E-07 | 3.4318924 | down |
| TTC7A | 9.23E-08 | 3.434723 | down |
| NAAA | 2.54E-07 | 3.4377697 | down |
| C2orf42 | 1.86E-07 | 3.4384038 | down |
| SEC63 | 2.78E-07 | 3.4407449 | down |
| RHOT1 | 1.03E-06 | 3.4426668 | down |
| LYSMD2 | 2.70E-07 | 3.4430625 | down |
| FBXL14 | 4.46E-07 | 3.4451263 | down |
| LOC646762 | 2.65E-07 | 3.4486768 | down |
| DMTF1 | 1.15E-07 | 3.456072 | down |
| HCP5 | 7.77E-08 | 3.4585483 | down |
| ZNF518A | 1.28E-06 | 3.4597545 | down |
| PPARG | 1.32E-07 | 3.4598744 | down |
| LINC00999 | 5.04E-08 | 3.4648325 | down |
| TMEM106B | 6.90E-07 | 3.4650576 | down |
| SDCCAG8 | 1.58E-07 | 3.4680884 | down |
| PSMG3-AS1 | 3.72E-07 | 3.4692445 | down |
| EMID1 | 1.31E-07 | 3.473688 | down |
| NFIA | 1.32E-06 | 3.4837446 | down |
| WARS | 1.36E-07 | 3.486092 | down |
| PFKFB2 | 1.64E-07 | 3.4866292 | down |
| CYB5R2 | 6.40E-07 | 3.4876533 | down |
| TRIM16L | 2.59E-07 | 3.488036 | down |
| HEXIM2 | 1.46E-07 | 3.492933 | down |
| GCC2 | 1.83E-07 | 3.5006063 | down |
| FGD4 | 4.18E-07 | 3.5036128 | down |
| LAMA1 | 1.87E-07 | 3.5043106 | down |
| CPSF2 | 1.38E-07 | 3.5051064 | down |
| GOLGA6L4 | 4.60E-07 | 3.5056312 | down |
| RILPL1 | 4.25E-08 | 3.50575 | down |
| ZNF767P | 3.67E-07 | 3.516989 | down |
| ZCWPW1 | 2.39E-07 | 3.5176604 | down |
| COQ10A | 4.87E-07 | 3.5227478 | down |
| FSIP1 | 5.20E-08 | 3.5241342 | down |
| IL10RB | 9.86E-08 | 3.5244539 | down |
| ABLIM1 | 1.05E-07 | 3.52494 | down |
| CAV3 | 1.82E-07 | 3.5271697 | down |
| TMEM245 | 4.59E-08 | 3.528285 | down |
| RPGRIP1L | 4.56E-07 | 3.5335858 | down |
| DOCK10 | 1.12E-07 | 3.5365348 | down |
| SLC35A1 | 1.86E-07 | 3.537484 | down |
| COL16A1 | 1.58E-07 | 3.5377645 | down |
| ZNF117 | 6.36E-08 | 3.5396285 | down |
| PIKFYVE | 7.21E-07 | 3.5418682 | down |
| NAIP | 4.94E-07 | 3.5433395 | down |
| SLCO3A1 | 9.34E-08 | 3.5436323 | down |
| TRIM66 | 4.27E-07 | 3.5457563 | down |
| RDH10 | 6.33E-07 | 3.5471857 | down |
| SYDE2 | 1.35E-07 | 3.5477235 | down |
| SNHG1 | 3.41E-08 | 3.5507524 | down |
| MPC2 | 7.81E-08 | 3.5546484 | down |
| PDE6B | 8.73E-08 | 3.555956 | down |
| FA2H | 8.14E-07 | 3.559024 | down |
| KIAA1033 | 4.05E-08 | 3.5608742 | down |
| WDR92 | 1.19E-07 | 3.5637283 | down |
| TYW1 | 6.38E-08 | 3.5637348 | down |
| DIS3L | 3.43E-07 | 3.5663795 | down |
| CTF1 | 1.02E-06 | 3.5671034 | down |
| MYO5C | 3.69E-07 | 3.567542 | down |
| PRKCA | 9.48E-08 | 3.573334 | down |
| CLDN15 | 1.40E-06 | 3.5772536 | down |
| CYB5R2 | 4.58E-08 | 3.577931 | down |
| USP44 | 4.39E-07 | 3.5783408 | down |
| LOC100133331 | 4.05E-08 | 3.5808716 | down |
| PRNP | 3.68E-07 | 3.5813205 | down |
| GOLGA6L9 | 3.46E-07 | 3.5821626 | down |
| TPR | 3.03E-07 | 3.5836375 | down |
| RTP4 | 7.59E-08 | 3.5862508 | down |
| EFCAB7 | 4.17E-08 | 3.587435 | down |
| C12orf66 | 2.58E-07 | 3.5892148 | down |
| AZIN2 | 6.33E-08 | 3.5893037 | down |
| KIAA1683 | 4.52E-08 | 3.5943289 | down |
| PLCE1 | 4.99E-07 | 3.5994523 | down |
| EML1 | 9.74E-07 | 3.602157 | down |
| DENND4C | 5.48E-08 | 3.6052363 | down |
| ADHFE1 | 1.40E-07 | 3.60933 | down |
| CDKN2AIP | 2.83E-07 | 3.611469 | down |
| SLC31A1 | 1.81E-07 | 3.6115818 | down |
| DLX2 | 9.94E-08 | 3.612097 | down |
| LNPEP | 3.04E-08 | 3.6124663 | down |
| LOC729987 | 7.66E-07 | 3.612698 | down |
| ENPP4 | 5.95E-08 | 3.6137168 | down |
| SP4 | 1.17E-07 | 3.6148036 | down |
| MEGF11 | 1.30E-07 | 3.6166744 | down |
| PPP1R3B | 5.32E-07 | 3.6181438 | down |
| DTX4 | 8.19E-07 | 3.6201744 | down |
| FAM124A | 6.12E-07 | 3.6271045 | down |
| EFNB3 | 2.14E-07 | 3.634388 | down |
| BDKRB1 | 5.30E-07 | 3.6378 | down |
| KIAA1033 | 1.29E-07 | 3.6416483 | down |
| CBX7 | 8.48E-07 | 3.6431146 | down |
| CCP110 | 5.04E-07 | 3.6495726 | down |
| BRINP1 | 6.42E-08 | 3.652001 | down |
| HLA-DMB | 2.18E-07 | 3.655095 | down |
| HSBP1L1 | 1.32E-06 | 3.6571069 | down |
| ATF1 | 3.07E-08 | 3.6578157 | down |
| SLCO4A1 | 3.34E-07 | 3.658573 | down |
| ADRA2C | 8.57E-07 | 3.6601832 | down |
| LIPT1 | 3.91E-07 | 3.6615255 | down |
| BLOC1S5 | 6.09E-08 | 3.6616962 | down |
| IFIT1 | 3.07E-07 | 3.666249 | down |
| IFIH1 | 1.79E-07 | 3.6687193 | down |
| MFSD7 | 7.89E-08 | 3.6731474 | down |
| FAS | 1.32E-07 | 3.6748402 | down |
| JAZF1 | 1.53E-07 | 3.6757576 | down |
| WDR60 | 1.17E-06 | 3.6806269 | down |
| EPDR1 | 7.88E-08 | 3.6811967 | down |
| NKTR | 5.94E-07 | 3.6826024 | down |
| LINC01547 | 2.72E-07 | 3.6850047 | down |
| MTL5 | 1.45E-06 | 3.6916034 | down |
| TTC30A | 9.02E-07 | 3.692394 | down |
| FAM208B | 5.79E-07 | 3.6960518 | down |
| TMEM209 | 1.10E-07 | 3.7017004 | down |
| ZNF138 | 8.44E-07 | 3.7061927 | down |
| AP1M2 | 1.10E-06 | 3.7086642 | down |
| NCOA7 | 3.28E-08 | 3.7124171 | down |
| IFT22 | 3.25E-07 | 3.7134373 | down |
| CBR4 | 8.24E-08 | 3.7186093 | down |
| SLC4A5 | 1.35E-06 | 3.72141 | down |
| ZFAND2A | 2.45E-07 | 3.7218864 | down |
| TTC8 | 4.85E-07 | 3.7220523 | down |
| ZBED6CL | 6.26E-08 | 3.7243717 | down |
| PARN | 1.44E-06 | 3.727829 | down |
| MAP9 | 6.75E-08 | 3.7295358 | down |
| DZIP3 | 1.55E-07 | 3.7329953 | down |
| LINC00923 | 1.36E-06 | 3.7352154 | down |
| MIF4GD | 9.42E-08 | 3.7364984 | down |
| C20orf195 | 8.78E-07 | 3.737216 | down |
| CD109 | 6.55E-08 | 3.7390442 | down |
| THNSL1 | 3.17E-08 | 3.7424278 | down |
| TMEM140 | 2.93E-07 | 3.7442875 | down |
| KIAA1467 | 2.07E-07 | 3.7477915 | down |
| VPS13A | 7.50E-08 | 3.748238 | down |
| FBRSL1 | 1.04E-07 | 3.751027 | down |
| DOCK1 | 2.87E-07 | 3.7520413 | down |
| RPL22 | 3.43E-07 | 3.752101 | down |
| APOBEC3G | 2.38E-07 | 3.7576246 | down |
| GABARAPL1 | 2.33E-07 | 3.759226 | down |
| CAV1 | 1.66E-07 | 3.7753 | down |
| TRIP11 | 1.57E-07 | 3.7786658 | down |
| lnc-NINJ2-2 | 1.13E-06 | 3.7801046 | down |
| CFLAR | 6.01E-07 | 3.7875786 | down |
| PIK3C2A | 6.01E-08 | 3.792523 | down |
| lnc-AC016745,1-2 | 2.47E-07 | 3.793546 | down |
| REPIN1 | 5.67E-07 | 3.7982292 | down |
| SLC38A1 | 7.55E-08 | 3.799482 | down |
| FLJ43315 | 1.33E-06 | 3.800329 | down |
| GCC2 | 4.37E-07 | 3.8011436 | down |
| TNXB | 5.67E-07 | 3.8097236 | down |
| EPB41L1 | 1.28E-07 | 3.8117378 | down |
| CREBBP | 1.35E-07 | 3.8182776 | down |
| TSPAN33 | 4.92E-08 | 3.8205469 | down |
| DNMBP | 8.61E-07 | 3.827522 | down |
| SSX2IP | 5.68E-07 | 3.8387773 | down |
| RNF207 | 3.99E-07 | 3.8394861 | down |
| SPATA6 | 1.24E-07 | 3.8414242 | down |
| TBC1D3B | 9.29E-07 | 3.8443692 | down |
| NPM2 | 9.37E-08 | 3.8444836 | down |
| STK33 | 7.11E-08 | 3.8482726 | down |
| GOLGA2P6 | 7.29E-08 | 3.8495288 | down |
| ADAM8 | 6.64E-08 | 3.85003 | down |
| GOLGA8EP | 6.08E-07 | 3.8508766 | down |
| OCLN | 5.15E-07 | 3.8552723 | down |
| GABARAPL1 | 2.33E-08 | 3.856316 | down |
| IMPACT | 1.03E-06 | 3.864807 | down |
| GAB3 | 5.43E-07 | 3.8662527 | down |
| UNC50 | 9.69E-08 | 3.8689067 | down |
| LOC100129534 | 4.60E-07 | 3.8693779 | down |
| P4HTM | 5.07E-07 | 3.8706188 | down |
| TRIM56 | 1.44E-07 | 3.871816 | down |
| ANKIB1 | 7.43E-08 | 3.872081 | down |
| NAV3 | 6.18E-08 | 3.8755767 | down |
| RIC8B | 3.57E-08 | 3.8784387 | down |
| TEAD4 | 7.81E-08 | 3.8839526 | down |
| MMP11 | 3.21E-07 | 3.8985615 | down |
| KRR1 | 3.38E-08 | 3.9020376 | down |
| NPY1R | 3.20E-07 | 3.9032998 | down |
| POLK | 3.65E-07 | 3.91105 | down |
| NBPF11 | 2.97E-07 | 3.9162197 | down |
| GNS | 1.14E-07 | 3.9213388 | down |
| CNTNAP1 | 4.81E-08 | 3.9233203 | down |
| ZNF518A | 1.23E-07 | 3.9280994 | down |
| STAT5A | 3.94E-07 | 3.929169 | down |
| GALNT16 | 2.73E-07 | 3.929695 | down |
| MAPKAPK5-AS1 | 5.11E-07 | 3.930487 | down |
| LINC01116 | 1.83E-07 | 3.9360518 | down |
| AFF1 | 9.10E-08 | 3.9371226 | down |
| CNEP1R1 | 2.73E-07 | 3.9398034 | down |
| LYPLAL1 | 9.02E-08 | 3.9448912 | down |
| PREPL | 1.10E-07 | 3.950653 | down |
| SPATA7 | 9.56E-08 | 3.9512339 | down |
| TMEM209 | 1.07E-07 | 3.9524314 | down |
| PTGR2 | 3.61E-07 | 3.954885 | down |
| WDR19 | 1.71E-07 | 3.9587193 | down |
| CYP1A2 | 3.49E-08 | 3.9788806 | down |
| TMEM61 | 2.17E-07 | 3.9790301 | down |
| FLJ42627 | 3.86E-08 | 3.979989 | down |
| FAM63B | 6.48E-08 | 3.9811363 | down |
| AZIN2 | 6.10E-07 | 3.9841847 | down |
| SMC3 | 1.06E-07 | 3.9850404 | down |
| HSD3B7 | 1.40E-06 | 3.9884074 | down |
| RNF213 | 1.91E-08 | 3.99604 | down |
| AGAP5 | 1.07E-06 | 3.9976077 | down |
| C1orf56 | 1.99E-07 | 4.0014467 | down |
| R3HCC1L | 2.23E-07 | 4.005511 | down |
| RGS17 | 1.83E-07 | 4.0130076 | down |
| SETBP1 | 7.02E-07 | 4.0146 | down |
| IRAK4 | 6.70E-07 | 4.0175796 | down |
| ZNF277 | 3.12E-08 | 4.0254626 | down |
| NCOA1 | 7.14E-07 | 4.028484 | down |
| TYSND1 | 2.38E-08 | 4.033206 | down |
| HEYL | 3.88E-07 | 4.036891 | down |
| FBLN5 | 8.40E-08 | 4.0410943 | down |
| SLC7A11 | 9.81E-07 | 4.043368 | down |
| ATP6V0E2-AS1 | 6.37E-08 | 4.0494647 | down |
| PARP10 | 1.60E-07 | 4.0498843 | down |
| PDE4A | 2.96E-08 | 4.0509815 | down |
| TRABD2B | 9.58E-08 | 4.051086 | down |
| PLCE1 | 1.81E-08 | 4.0630846 | down |
| LSMEM1 | 1.84E-07 | 4.064062 | down |
| PMEL | 6.36E-08 | 4.06955 | down |
| HS1BP3 | 7.62E-07 | 4.079532 | down |
| LOC101927345 | 5.94E-07 | 4.081176 | down |
| CDKN1B | 1.21E-07 | 4.081546 | down |
| PGAP1 | 2.16E-07 | 4.0844235 | down |
| TFPI2 | 6.73E-08 | 4.086468 | down |
| R3HCC1L | 5.17E-07 | 4.087518 | down |
| METTL15 | 3.52E-08 | 4.0924926 | down |
| SLC7A2 | 9.54E-08 | 4.094984 | down |
| NPHP1 | 7.14E-08 | 4.096157 | down |
| S1PR3 | 8.18E-08 | 4.0986385 | down |
| FKTN | 6.67E-07 | 4.100129 | down |
| THAP6 | 2.78E-07 | 4.1031857 | down |
| NTF3 | 4.68E-08 | 4.1031923 | down |
| CCDC113 | 1.31E-06 | 4.1042714 | down |
| MAP3K15 | 5.07E-08 | 4.1064286 | down |
| RIIAD1 | 3.59E-08 | 4.107503 | down |
| FAM208A | 5.98E-08 | 4.114047 | down |
| ZBED2 | 1.71E-07 | 4.1144648 | down |
| ZMIZ2 | 2.19E-07 | 4.1171155 | down |
| ZGLP1 | 2.24E-08 | 4.1179447 | down |
| BCL3 | 2.65E-07 | 4.1181498 | down |
| BMP4 | 1.45E-07 | 4.1194563 | down |
| BDKRB2 | 5.76E-07 | 4.1228337 | down |
| APOLD1 | 5.35E-08 | 4.1233454 | down |
| GORAB | 3.19E-08 | 4.1263475 | down |
| ZNF692 | 1.39E-07 | 4.127923 | down |
| LEF1 | 3.40E-08 | 4.1335387 | down |
| WSB1 | 3.90E-07 | 4.141297 | down |
| SGK2 | 1.67E-07 | 4.147588 | down |
| SIRPG | 3.22E-07 | 4.1482553 | down |
| ENDOV | 6.38E-08 | 4.148459 | down |
| CD24 | 5.43E-08 | 4.150072 | down |
| CFD | 9.31E-08 | 4.1554723 | down |
| C9orf64 | 1.11E-07 | 4.1609898 | down |
| LINC00174 | 1.53E-08 | 4.163276 | down |
| ICA1 | 1.51E-07 | 4.1658363 | down |
| ZNRF2 | 3.53E-07 | 4.166602 | down |
| SLC26A11 | 1.11E-07 | 4.175022 | down |
| SKAP2 | 3.74E-08 | 4.189981 | down |
| REL | 3.60E-07 | 4.190828 | down |
| ATP6V1E2 | 4.34E-08 | 4.192797 | down |
| HEBP1 | 1.59E-08 | 4.1939225 | down |
| ZDHHC15 | 2.56E-07 | 4.1942925 | down |
| CTSC | 7.33E-08 | 4.1967688 | down |
| PRRT2 | 1.26E-06 | 4.197442 | down |
| THY1 | 9.98E-08 | 4.2060995 | down |
| KCP | 1.07E-06 | 4.2081437 | down |
| LINC01061 | 3.35E-08 | 4.2096033 | down |
| IKZF2 | 8.36E-07 | 4.2113724 | down |
| C11orf70 | 3.68E-08 | 4.2120137 | down |
| PLCL2 | 2.24E-07 | 4.2286344 | down |
| CASD1 | 1.14E-07 | 4.2289476 | down |
| GKAP1 | 1.95E-07 | 4.2313566 | down |
| PLXDC2 | 2.99E-08 | 4.2358456 | down |
| NOV | 3.12E-08 | 4.239596 | down |
| SARM1 | 2.95E-07 | 4.240807 | down |
| GGTLC2 | 2.32E-07 | 4.248004 | down |
| CEP95 | 6.37E-08 | 4.2486057 | down |
| NUDT7 | 1.53E-07 | 4.2490892 | down |
| DPH5 | 9.13E-08 | 4.251285 | down |
| NEURL3 | 2.95E-08 | 4.2516522 | down |
| MBTD1 | 1.54E-07 | 4.25488 | down |
| MORN4 | 1.82E-08 | 4.2591133 | down |
| NAT8L | 8.91E-08 | 4.264952 | down |
| ZCCHC8 | 1.04E-07 | 4.2660537 | down |
| DYNC2H1 | 6.85E-07 | 4.268299 | down |
| ZNF107 | 2.26E-07 | 4.268917 | down |
| WHAMMP3 | 5.11E-07 | 4.270209 | down |
| IGFBP6 | 2.60E-08 | 4.2713532 | down |
| ZNF75A | 2.83E-07 | 4.271847 | down |
| DNPEP | 7.36E-08 | 4.2800894 | down |
| NME5 | 2.53E-07 | 4.2832284 | down |
| PTPRE | 1.44E-07 | 4.285807 | down |
| CCDC186 | 4.00E-08 | 4.288482 | down |
| AFAP1L2 | 1.33E-07 | 4.295588 | down |
| GTPBP10 | 1.94E-07 | 4.2971153 | down |
| CES3 | 6.25E-07 | 4.3079314 | down |
| TPP1 | 1.25E-06 | 4.309736 | down |
| ADCY9 | 1.99E-07 | 4.3115826 | down |
| PRPF4B | 4.63E-08 | 4.3122787 | down |
| RNF135 | 1.60E-08 | 4.317709 | down |
| KCNH2 | 5.46E-07 | 4.323577 | down |
| ZNF140 | 4.00E-07 | 4.3272915 | down |
| FGF2 | 1.86E-07 | 4.3376427 | down |
| FGD6 | 1.65E-07 | 4.3380733 | down |
| NHLRC3 | 6.86E-07 | 4.3422565 | down |
| USP32 | 4.19E-07 | 4.34699 | down |
| RAB11FIP2 | 1.05E-07 | 4.3471956 | down |
| MCTP2 | 1.02E-07 | 4.3509364 | down |
| NR1D2 | 3.24E-07 | 4.351487 | down |
| FAM161A | 3.25E-08 | 4.3601584 | down |
| LSM14A | 5.38E-07 | 4.3629603 | down |
| ZMYM6NB | 7.62E-08 | 4.3642993 | down |
| LPHN2 | 4.94E-08 | 4.3652906 | down |
| RNU4ATAC | 3.31E-08 | 4.3723574 | down |
| FAXC | 9.41E-07 | 4.373494 | down |
| COX15 | 1.77E-08 | 4.37422 | down |
| FGF2 | 2.58E-07 | 4.391761 | down |
| MUC3A | 1.03E-06 | 4.3927884 | down |
| MMAB | 1.25E-07 | 4.3951836 | down |
| CRYM-AS1 | 3.96E-07 | 4.4039183 | down |
| NPR3 | 4.65E-08 | 4.409015 | down |
| ACAD10 | 4.38E-07 | 4.4098115 | down |
| ERP29 | 2.02E-07 | 4.414109 | down |
| CLPSL2 | 6.17E-07 | 4.4173584 | down |
| CFI | 1.05E-06 | 4.419039 | down |
| MST1 | 3.04E-08 | 4.419325 | down |
| TEX19 | 6.06E-07 | 4.420746 | down |
| ICA1 | 4.68E-08 | 4.423139 | down |
| ADCY6 | 2.25E-07 | 4.424166 | down |
| PNPT1 | 1.96E-07 | 4.430573 | down |
| ETV6 | 5.92E-07 | 4.4362125 | down |
| SLC35F3 | 1.81E-08 | 4.440285 | down |
| DYX1C1 | 2.47E-07 | 4.44371 | down |
| PPP1R1C | 7.10E-08 | 4.4517407 | down |
| MPP3 | 4.48E-08 | 4.4519863 | down |
| NAT16 | 2.92E-07 | 4.452776 | down |
| PLEKHH2 | 1.45E-07 | 4.4542975 | down |
| SPTLC3 | 2.71E-08 | 4.4636226 | down |
| GSDMB | 1.16E-06 | 4.4646244 | down |
| TMPRSS7 | 2.51E-07 | 4.467377 | down |
| IRX3 | 1.45E-08 | 4.485165 | down |
| PSAP | 1.24E-06 | 4.4858923 | down |
| FAM78B | 3.43E-07 | 4.489493 | down |
| MGAT3 | 3.31E-08 | 4.4929457 | down |
| DNAJC10 | 3.94E-08 | 4.493089 | down |
| RIMS2 | 1.31E-06 | 4.4938116 | down |
| TSPAN12 | 3.98E-07 | 4.495271 | down |
| NPIPB5 | 3.79E-08 | 4.4954085 | down |
| UPF3B | 2.89E-07 | 4.495721 | down |
| MED13L | 2.94E-07 | 4.4997964 | down |
| SUSD4 | 4.73E-07 | 4.503987 | down |
| PSAT1 | 1.80E-07 | 4.50502 | down |
| CNTN6 | 1.35E-08 | 4.5162024 | down |
| LOC650293 | 3.80E-07 | 4.516338 | down |
| GOLGA6L9 | 1.34E-06 | 4.5270815 | down |
| KLRF1 | 9.43E-07 | 4.5293922 | down |
| AKTIP | 4.32E-08 | 4.5316105 | down |
| KLF2 | 4.94E-07 | 4.5359826 | down |
| PRTG | 3.97E-07 | 4.541071 | down |
| TMTC2 | 1.15E-07 | 4.5470333 | down |
| IDH1 | 1.33E-08 | 4.547787 | down |
| SHC2 | 5.36E-08 | 4.5575204 | down |
| JPH3 | 4.44E-08 | 4.558447 | down |
| LETMD1 | 1.49E-07 | 4.559219 | down |
| SLC46A1 | 2.19E-08 | 4.561125 | down |
| PRH2 | 4.53E-08 | 4.564205 | down |
| FAM184A | 2.94E-08 | 4.568582 | down |
| TRMT10B | 2.96E-07 | 4.5741715 | down |
| SLC4A11 | 5.05E-08 | 4.5817084 | down |
| MAP3K5 | 1.16E-07 | 4.60738 | down |
| FAM223A | 5.21E-07 | 4.6128874 | down |
| PAXIP1-AS2 | 3.98E-08 | 4.6145024 | down |
| NCR3LG1 | 5.80E-08 | 4.6183596 | down |
| TTC28 | 7.42E-08 | 4.6186304 | down |
| C6orf48 | 1.11E-08 | 4.624062 | down |
| TYW5 | 1.20E-07 | 4.6245985 | down |
| GCG | 6.17E-07 | 4.6272087 | down |
| CD9 | 1.75E-08 | 4.632782 | down |
| CLIP4 | 2.50E-07 | 4.637354 | down |
| PPCDC | 4.46E-08 | 4.6377993 | down |
| GPR39 | 2.92E-08 | 4.6384954 | down |
| C2CD5 | 2.98E-07 | 4.6385484 | down |
| BEND7 | 3.06E-07 | 4.641266 | down |
| DLK2 | 4.34E-08 | 4.6421256 | down |
| TTC21A | 2.59E-08 | 4.64252 | down |
| CC2D2A | 4.15E-08 | 4.643664 | down |
| EPB41L4A | 7.98E-08 | 4.6438336 | down |
| GOLGA4 | 6.33E-07 | 4.646277 | down |
| PNMA2 | 2.68E-08 | 4.6577587 | down |
| CPT1A | 5.47E-08 | 4.661938 | down |
| CCNO | 1.01E-07 | 4.663084 | down |
| PLEKHH2 | 6.60E-08 | 4.6658063 | down |
| IL20RB | 5.09E-07 | 4.6676135 | down |
| CLIP1 | 3.30E-08 | 4.668434 | down |
| BBS2 | 1.27E-07 | 4.6713142 | down |
| METTL21A | 1.23E-08 | 4.671471 | down |
| SNAPC5 | 1.20E-06 | 4.67532 | down |
|  | 2.54E-07 | 4.6894517 | down |
| SLC11A2 | 1.37E-08 | 4.690323 | down |
| C6orf48 | 1.39E-08 | 4.6904554 | down |
| CPNE7 | 7.79E-08 | 4.693081 | down |
| ANKRD12 | 5.75E-08 | 4.7009153 | down |
| ITGBL1 | 5.97E-07 | 4.706453 | down |
| SEMA3B | 5.53E-08 | 4.7084365 | down |
| SLC25A37 | 2.31E-08 | 4.7140694 | down |
| KRT80 | 4.17E-08 | 4.7180395 | down |
| MAPK8IP2 | 1.00E-07 | 4.720863 | down |
| GNAI1 | 2.74E-08 | 4.7216816 | down |
| RBM41 | 2.44E-08 | 4.730296 | down |
| CPNE8 | 7.05E-08 | 4.7317457 | down |
| RECK | 3.46E-08 | 4.7398977 | down |
| SPP1 | 1.94E-07 | 4.743642 | down |
| HLA-DPA1 | 1.61E-07 | 4.749139 | down |
| MEG3 | 1.81E-08 | 4.750621 | down |
| GALNT12 | 1.28E-07 | 4.7513523 | down |
| ANKRD34B | 1.33E-06 | 4.761114 | down |
| PLEKHB1 | 3.44E-08 | 4.7620573 | down |
| TMEM79 | 4.52E-07 | 4.77045 | down |
| ZNF655 | 1.84E-08 | 4.7710114 | down |
| RHNO1 | 1.69E-07 | 4.7934537 | down |
| IFIT3 | 5.59E-07 | 4.796842 | down |
| GGT1 | 9.66E-07 | 4.804849 | down |
| LAMB2P1 | 3.64E-07 | 4.8060346 | down |
| CCDC88A | 7.57E-08 | 4.811922 | down |
| LINC01061 | 1.19E-07 | 4.816201 | down |
| RAB38 | 2.13E-08 | 4.826241 | down |
| TPTE2P6 | 1.04E-07 | 4.84312 | down |
| SMG1 | 1.70E-07 | 4.849439 | down |
| LRRC23 | 4.14E-08 | 4.849892 | down |
| SLC7A6 | 8.72E-07 | 4.851049 | down |
| FAM135A | 2.32E-07 | 4.8533134 | down |
| XPC | 3.10E-08 | 4.8565683 | down |
| LOC644662 | 7.19E-07 | 4.8571544 | down |
| KAT2B | 3.71E-07 | 4.861891 | down |
| MDM1 | 6.71E-08 | 4.876962 | down |
| LOC100996634 | 2.83E-07 | 4.877916 | down |
| SHANK2 | 6.12E-07 | 4.878867 | down |
| TXNL4B | 8.05E-07 | 4.881974 | down |
| LOC155060 | 9.93E-07 | 4.8835764 | down |
| AGAP2-AS1 | 2.33E-08 | 4.8863664 | down |
| NME9 | 1.35E-06 | 4.8935475 | down |
| FAT4 | 7.89E-07 | 4.897251 | down |
| RAB29 | 3.90E-08 | 4.9004254 | down |
| MUTYH | 2.26E-08 | 4.905113 | down |
| SERPINA6 | 1.59E-07 | 4.9071865 | down |
| SCPEP1 | 7.82E-07 | 4.9077096 | down |
| ZNF541 | 5.45E-07 | 4.909941 | down |
| FBXO16 | 1.66E-07 | 4.9146824 | down |
| SLTM | 5.13E-08 | 4.919473 | down |
| PITX1 | 2.73E-07 | 4.921071 | down |
| SORL1 | 2.50E-08 | 4.931616 | down |
| PRUNE2 | 1.81E-08 | 4.9364877 | down |
| C11orf45 | 6.64E-08 | 4.939266 | down |
| SEMA5B | 3.52E-07 | 4.9449973 | down |
| IFI44L | 4.23E-07 | 4.946498 | down |
| CREBRF | 1.56E-08 | 4.948811 | down |
| OCLN | 3.28E-07 | 4.9524016 | down |
| FBXO27 | 1.26E-08 | 4.9592543 | down |
| FAM229B | 3.84E-07 | 4.9675016 | down |
| CUZD1 | 8.78E-07 | 4.969539 | down |
| LINC00965 | 5.17E-07 | 4.9703627 | down |
| MLXIPL | 2.11E-07 | 4.9836445 | down |
| STAT1 | 5.08E-08 | 4.991724 | down |
| PRH2 | 1.18E-08 | 4.9992332 | down |
| PYGO1 | 1.09E-07 | 5.0002 | down |
| LIPC | 4.51E-08 | 5.0065813 | down |
| MCTP1 | 8.62E-08 | 5.0224757 | down |
| LINC00116 | 3.28E-07 | 5.032666 | down |
| DRAM1 | 6.99E-08 | 5.034537 | down |
| FLT3LG | 5.57E-07 | 5.0355105 | down |
| RECQL5 | 1.73E-08 | 5.0388885 | down |
| TMEM229B | 1.26E-08 | 5.0464454 | down |
| IQCK | 2.62E-08 | 5.0475607 | down |
| ACAD11 | 1.58E-07 | 5.0477915 | down |
| LOC729737 | 2.46E-08 | 5.0494604 | down |
| KIF27 | 1.02E-06 | 5.053187 | down |
| LOC388242 | 1.01E-07 | 5.0532293 | down |
| ARSD | 1.08E-06 | 5.0536594 | down |
| ZDHHC23 | 7.96E-09 | 5.058918 | down |
| PRR29 | 7.37E-08 | 5.0593896 | down |
| TOM1L1 | 3.30E-08 | 5.061833 | down |
| VLDLR-AS1 | 6.41E-07 | 5.069643 | down |
| RRAGD | 7.40E-08 | 5.071825 | down |
| ATP2B1 | 8.46E-08 | 5.0751805 | down |
| UST | 5.37E-07 | 5.083156 | down |
| LRRC75A | 2.97E-08 | 5.0860167 | down |
| MKLN1 | 4.10E-08 | 5.0972714 | down |
| DIRC1 | 1.88E-08 | 5.098569 | down |
| PCLO | 6.43E-07 | 5.1007576 | down |
| LINC00240 | 8.71E-07 | 5.108492 | down |
| FGF20 | 1.13E-07 | 5.108516 | down |
| LIAS | 8.44E-07 | 5.1157293 | down |
| SYT16 | 1.37E-07 | 5.117642 | down |
| MSX1 | 8.21E-07 | 5.1206384 | down |
| PYGL | 1.71E-08 | 5.1265893 | down |
| HLA-DPB1 | 2.91E-08 | 5.1267257 | down |
| CFI | 1.17E-08 | 5.136642 | down |
| IRS2 | 2.77E-08 | 5.1394405 | down |
| PLEKHA6 | 4.33E-08 | 5.1467266 | down |
| RHPN1 | 1.76E-08 | 5.159435 | down |
| C20orf141 | 2.57E-07 | 5.1707377 | down |
| VLDLR | 1.96E-07 | 5.1730556 | down |
| CACNA2D1 | 1.38E-07 | 5.1917524 | down |
| SYNE2 | 4.17E-07 | 5.191842 | down |
| PLTP | 2.23E-08 | 5.1957173 | down |
| SOS1 | 8.35E-07 | 5.1962385 | down |
| GLI3 | 8.40E-09 | 5.201218 | down |
| NPIPB15 | 1.21E-07 | 5.2014723 | down |
| PCMTD1 | 6.58E-07 | 5.203995 | down |
| NUP210 | 1.38E-07 | 5.226343 | down |
| RIMKLB | 9.79E-09 | 5.2318277 | down |
| ANO5 | 2.25E-07 | 5.2325644 | down |
| MSH5 | 7.24E-08 | 5.2394834 | down |
| TWSG1 | 2.31E-07 | 5.2420297 | down |
| RNF207 | 1.44E-07 | 5.274673 | down |
| SYNE1 | 2.77E-08 | 5.2756453 | down |
| NPIPA1 | 2.76E-07 | 5.2801766 | down |
| SIKE1 | 1.41E-06 | 5.2818356 | down |
| TMEM182 | 2.37E-08 | 5.290547 | down |
| NAMPT | 1.96E-08 | 5.302463 | down |
| PPARGC1A | 1.37E-08 | 5.303145 | down |
| UBA7 | 2.92E-08 | 5.3080535 | down |
| PRKD1 | 1.47E-07 | 5.319632 | down |
| GOLGA8R | 8.78E-08 | 5.3213763 | down |
| TESC | 1.31E-08 | 5.329006 | down |
| KCNH8 | 2.75E-07 | 5.3352256 | down |
| GFRA1 | 5.05E-08 | 5.3543553 | down |
| FOXL2 | 5.82E-08 | 5.363645 | down |
| IGIP | 3.15E-08 | 5.363931 | down |
| LRRC6 | 1.45E-07 | 5.3657203 | down |
| ODF3B | 2.70E-08 | 5.3664317 | down |
| PRB4 | 9.96E-08 | 5.366858 | down |
| PACRGL | 1.38E-06 | 5.3758616 | down |
| KIZ | 2.37E-08 | 5.3885875 | down |
| CTBS | 1.54E-08 | 5.3916454 | down |
| VSIG10 | 1.77E-08 | 5.3980465 | down |
| MAGI2 | 8.73E-09 | 5.399756 | down |
| MCIDAS | 2.78E-07 | 5.4015403 | down |
| LYST | 5.55E-08 | 5.414244 | down |
| CHURC1 | 7.49E-08 | 5.4165735 | down |
| PPM1N | 5.37E-07 | 5.419833 | down |
| ERP29 | 4.11E-08 | 5.428782 | down |
| ATHL1 | 5.94E-07 | 5.4343596 | down |
| MDFIC | 1.30E-07 | 5.4615207 | down |
| ZPLD1 | 2.80E-08 | 5.461723 | down |
| PPP1R9A | 3.29E-08 | 5.4786634 | down |
| NPIPB9 | 2.76E-08 | 5.4830413 | down |
| PDE11A | 1.44E-07 | 5.4834924 | down |
| CYB5RL | 5.19E-07 | 5.48364 | down |
| UGT2B10 | 1.08E-08 | 5.4848566 | down |
| KCNJ8 | 1.13E-08 | 5.4851093 | down |
| IFI44 | 9.87E-08 | 5.488243 | down |
| KIAA1551 | 9.57E-07 | 5.4894123 | down |
| CLMN | 1.11E-08 | 5.49938 | down |
| ADAMTS13 | 6.23E-09 | 5.504733 | down |
| SUPT7L | 3.55E-08 | 5.5623007 | down |
| SLC4A4 | 7.26E-08 | 5.563416 | down |
| RAPGEF3 | 3.31E-08 | 5.567346 | down |
| GFPT1 | 1.23E-08 | 5.5723863 | down |
| HAS3 | 3.78E-07 | 5.6028976 | down |
| PLCB4 | 7.51E-09 | 5.610974 | down |
| KLF11 | 5.98E-08 | 5.6137037 | down |
| FZD1 | 3.04E-08 | 5.61572 | down |
| PITX2 | 6.60E-08 | 5.6177907 | down |
| FAM149B1 | 6.11E-08 | 5.6178613 | down |
| CYP4V2 | 2.57E-08 | 5.630901 | down |
| L1CAM | 5.40E-07 | 5.6318407 | down |
| SCG5 | 2.69E-08 | 5.633117 | down |
| PRPH | 6.15E-07 | 5.638138 | down |
| ITGA7 | 1.56E-08 | 5.648143 | down |
| CCPG1 | 5.21E-07 | 5.659464 | down |
| GRK5 | 2.23E-08 | 5.6803536 | down |
| TUBE1 | 3.14E-08 | 5.6852455 | down |
| IFT81 | 6.70E-09 | 5.6960936 | down |
| NUPL2 | 8.36E-08 | 5.7051835 | down |
| NOVA1 | 4.66E-08 | 5.708311 | down |
| GOLGA8EP | 8.31E-09 | 5.71238 | down |
| GLI1 | 1.34E-07 | 5.7135878 | down |
| NT5M | 1.18E-08 | 5.721745 | down |
| PCGF5 | 3.52E-08 | 5.728977 | down |
| C9orf43 | 1.27E-06 | 5.7349358 | down |
| STYK1 | 4.22E-07 | 5.7434025 | down |
| SMG1P1 | 1.34E-08 | 5.746133 | down |
| GOLGA8M | 5.34E-08 | 5.7488875 | down |
| CALCOCO2 | 3.02E-08 | 5.758117 | down |
| LAMB3 | 3.32E-08 | 5.761559 | down |
| RAB42 | 3.71E-08 | 5.763748 | down |
| RAB3IP | 1.18E-08 | 5.764596 | down |
| RRAD | 4.59E-07 | 5.7730722 | down |
| ZFHX4 | 3.61E-08 | 5.7770176 | down |
| GOLGA6A | 2.74E-07 | 5.7824144 | down |
| TMEM67 | 2.58E-07 | 5.785641 | down |
| GCGR | 6.22E-08 | 5.788669 | down |
| FAM159B | 7.17E-07 | 5.797751 | down |
| GALM | 1.76E-07 | 5.7995524 | down |
| SNCA | 1.58E-08 | 5.799749 | down |
| TNS3 | 3.49E-08 | 5.8086157 | down |
| ENPP5 | 2.22E-07 | 5.816901 | down |
| EGFR | 7.63E-09 | 5.81703 | down |
| MSR1 | 2.64E-07 | 5.8187857 | down |
| LIFR | 1.79E-08 | 5.8243694 | down |
| DLGAP1 | 5.59E-08 | 5.8254833 | down |
| LTBR | 6.13E-07 | 5.8264875 | down |
| TMBIM4 | 2.18E-08 | 5.8275294 | down |
| STEAP2 | 7.80E-07 | 5.833106 | down |
| ALPK1 | 1.07E-07 | 5.8367686 | down |
| PPP4R4 | 3.22E-08 | 5.838282 | down |
| RBKS | 4.45E-09 | 5.841156 | down |
| TCTN1 | 5.85E-09 | 5.845398 | down |
| ARHGEF25 | 1.14E-07 | 5.8471284 | down |
| SCRN3 | 9.35E-09 | 5.8557687 | down |
| PARD6B | 1.34E-07 | 5.859118 | down |
| CAV2 | 3.19E-08 | 5.8668566 | down |
| ITGA1 | 4.18E-08 | 5.8932886 | down |
| GALM | 8.93E-09 | 5.894203 | down |
| CDKL2 | 1.71E-07 | 5.8992114 | down |
| TRIM38 | 3.61E-08 | 5.9054284 | down |
| TRIM36 | 7.37E-08 | 5.9069033 | down |
| LOC399815 | 1.25E-07 | 5.908022 | down |
| ABCC6 | 4.33E-08 | 5.9145575 | down |
| LOC79999 | 2.06E-07 | 5.9223423 | down |
| SPATA18 | 1.27E-08 | 5.923319 | down |
| TMCO4 | 8.05E-09 | 5.946016 | down |
| PROS1 | 8.26E-08 | 5.9511786 | down |
| NXPH4 | 1.01E-07 | 5.9514146 | down |
| TLR3 | 1.61E-08 | 5.954604 | down |
| SUSD4 | 9.93E-07 | 5.954841 | down |
| PREPL | 2.86E-08 | 5.9594297 | down |
| ANKRD20A12P | 5.39E-08 | 5.96342 | down |
| BCL2 | 6.39E-08 | 5.978878 | down |
| TRIM4 | 5.43E-08 | 5.9848638 | down |
| LOC100130691 | 1.52E-07 | 5.9874716 | down |
| SOCS3 | 1.07E-07 | 5.98826 | down |
| RBM43 | 4.78E-07 | 5.992532 | down |
| CCDC88B | 2.40E-08 | 5.9950647 | down |
| GYG2 | 1.35E-08 | 5.9961596 | down |
| SMIM6 | 2.41E-08 | 6.0004954 | down |
| XLOC_l2_003882 | 7.81E-09 | 6.007137 | down |
| CHRNA7 | 1.65E-08 | 6.009526 | down |
| ADAMTS9 | 8.26E-08 | 6.0315022 | down |
| PRDM8 | 1.84E-07 | 6.041479 | down |
| THBS3 | 1.95E-08 | 6.042805 | down |
| ROCK2 | 8.99E-09 | 6.0478215 | down |
| KCNIP3 | 4.07E-08 | 6.060574 | down |
| CEP112 | 5.70E-07 | 6.064002 | down |
| PKD2 | 2.17E-08 | 6.0675125 | down |
| TMEM253 | 4.38E-07 | 6.07132 | down |
| KCNH1 | 8.41E-07 | 6.071328 | down |
| EPHA10 | 9.15E-07 | 6.0778756 | down |
| EPAS1 | 4.89E-08 | 6.0791893 | down |
| FAM110C | 2.65E-08 | 6.0822725 | down |
| CTSH | 4.46E-09 | 6.085993 | down |
| ZNF720 | 1.21E-08 | 6.1068697 | down |
| SORBS2 | 4.08E-08 | 6.1114554 | down |
| CNTLN | 1.29E-08 | 6.116469 | down |
| TTC29 | 3.84E-07 | 6.1273265 | down |
| FBXO15 | 2.77E-08 | 6.1384215 | down |
| C8orf46 | 2.32E-08 | 6.141858 | down |
| RBM47 | 1.23E-08 | 6.1461453 | down |
| HCN3 | 2.42E-07 | 6.158893 | down |
| CCL28 | 1.84E-08 | 6.20004 | down |
| WDR35 | 4.38E-08 | 6.2005296 | down |
| KLF5 | 1.18E-06 | 6.2020082 | down |
| SPNS3 | 9.51E-09 | 6.2078238 | down |
| IFT80 | 4.95E-08 | 6.2141547 | down |
| LOC254896 | 6.36E-08 | 6.2566648 | down |
| DNASE2 | 1.11E-07 | 6.2687774 | down |
| SUSD4 | 6.00E-07 | 6.2784843 | down |
| TPK1 | 1.40E-08 | 6.284525 | down |
| PPM1N | 6.42E-08 | 6.2859845 | down |
| GSTT2 | 4.24E-08 | 6.2976756 | down |
| PPL | 1.23E-07 | 6.3011465 | down |
| SAMD13 | 1.41E-07 | 6.317666 | down |
| NR1H3 | 8.47E-09 | 6.3198576 | down |
| HKDC1 | 3.24E-08 | 6.3234954 | down |
| UGT2B7 | 2.80E-07 | 6.3277354 | down |
| ANXA4 | 1.66E-08 | 6.3330793 | down |
| AKR1B1 | 6.28E-09 | 6.337773 | down |
| PLSCR1 | 7.96E-08 | 6.3406453 | down |
| CCDC88B | 6.84E-08 | 6.3417006 | down |
| WDR19 | 1.17E-08 | 6.345786 | down |
| HCFC1R1 | 8.73E-08 | 6.3713827 | down |
| SLC22A15 | 1.88E-08 | 6.3815484 | down |
| lnc-GOLGA8J-3 | 7.68E-09 | 6.3949986 | down |
| C14orf79 | 1.37E-07 | 6.399298 | down |
| CCDC103 | 1.22E-08 | 6.417376 | down |
| PTPDC1 | 1.02E-06 | 6.420646 | down |
| FLVCR1-AS1 | 3.99E-07 | 6.436513 | down |
| GOLGA6L9 | 3.01E-07 | 6.447964 | down |
| ALDH3A2 | 7.27E-09 | 6.451744 | down |
| DHRS3 | 3.71E-08 | 6.4518347 | down |
| FAM24B | 2.11E-08 | 6.4558544 | down |
| SPINK13 | 6.34E-07 | 6.4645286 | down |
| UGT2B11 | 3.87E-07 | 6.473223 | down |
| SLC37A3 | 3.66E-08 | 6.5002418 | down |
| CDC42BPA | 2.55E-08 | 6.5071225 | down |
| TMEM159 | 2.93E-07 | 6.536019 | down |
| CDH10 | 1.89E-08 | 6.537754 | down |
| RIC8B | 1.45E-07 | 6.5417333 | down |
| HNF4G | 3.62E-07 | 6.553965 | down |
| ZNF33B | 7.56E-08 | 6.5585485 | down |
| EPHB6 | 1.07E-07 | 6.575761 | down |
| lnc-RASSF7-1 | 2.27E-07 | 6.579072 | down |
| DYNLRB2 | 4.93E-07 | 6.5796666 | down |
| HIST1H2AC | 1.88E-07 | 6.5909095 | down |
| CCDC91 | 5.03E-08 | 6.600652 | down |
| TMEM74B | 1.14E-08 | 6.604148 | down |
| ISCU | 1.54E-08 | 6.6267533 | down |
| PSMB9 | 1.27E-08 | 6.631051 | down |
| FETUB | 1.37E-07 | 6.642195 | down |
| PDGFRL | 4.98E-07 | 6.642305 | down |
| ZNF853 | 9.57E-07 | 6.651311 | down |
| PAN2 | 3.16E-07 | 6.654151 | down |
| LOC102724332 | 4.89E-07 | 6.66338 | down |
| NEURL1 | 1.21E-08 | 6.6769385 | down |
| ITPKA | 5.23E-08 | 6.6822457 | down |
| LPHN2 | 1.97E-08 | 6.700689 | down |
| C1QTNF5 | 6.49E-08 | 6.7084727 | down |
| SLC47A2 | 8.70E-07 | 6.7207174 | down |
| DOC2A | 9.48E-07 | 6.7261977 | down |
| SYCE3 | 5.41E-08 | 6.7287135 | down |
| FAM129A | 8.44E-09 | 6.742299 | down |
| FTCDNL1 | 4.06E-07 | 6.7534328 | down |
| HS3ST1 | 9.20E-08 | 6.7547383 | down |
| XK | 5.76E-08 | 6.7610207 | down |
| KLF8 | 1.15E-06 | 6.775833 | down |
| DENND2D | 5.29E-08 | 6.796392 | down |
| SGPP2 | 3.15E-07 | 6.8024306 | down |
| LHFP | 6.46E-09 | 6.8119006 | down |
| JAKMIP3 | 1.28E-07 | 6.8263645 | down |
| FOXL2NB | 1.17E-06 | 6.8471975 | down |
| EEA1 | 2.96E-08 | 6.856385 | down |
| PSPH | 1.20E-08 | 6.8598943 | down |
| SAPCD1 | 2.86E-08 | 6.862122 | down |
| RNF128 | 1.40E-06 | 6.8658166 | down |
| NAPEPLD | 7.38E-09 | 6.8861146 | down |
| ANK3 | 5.21E-09 | 6.9131074 | down |
| SIAE | 9.93E-08 | 6.922891 | down |
| LMF1 | 9.40E-07 | 6.926416 | down |
| KCNJ13 | 6.45E-08 | 6.9303384 | down |
| ELF3 | 8.96E-09 | 6.9451365 | down |
| ALDH3B1 | 7.63E-09 | 6.951395 | down |
| AGAP9 | 1.69E-08 | 6.9611697 | down |
| OXER1 | 3.42E-09 | 6.967349 | down |
| ZNF497 | 1.18E-07 | 6.975297 | down |
| ACSL6 | 4.51E-07 | 6.9769735 | down |
| CCDC64 | 5.37E-07 | 6.9804196 | down |
| PCDH1 | 1.03E-07 | 6.980828 | down |
| DNAH5 | 5.37E-08 | 6.981807 | down |
| AGAP9 | 2.36E-08 | 6.995418 | down |
| EGFL8 | 9.98E-08 | 7.013272 | down |
| DKFZP586I1420 | 2.03E-07 | 7.038785 | down |
| MATN3 | 6.05E-07 | 7.059768 | down |
| ADAMTSL3 | 8.24E-09 | 7.0720096 | down |
| AKR1C4 | 1.72E-07 | 7.078494 | down |
| ANKRD26 | 2.47E-08 | 7.0912576 | down |
| CCL20 | 1.72E-07 | 7.1117187 | down |
| TMEM116 | 8.50E-09 | 7.1478763 | down |
| PPAP2B | 3.60E-08 | 7.1607966 | down |
| IFRD1 | 1.48E-07 | 7.162051 | down |
| FAM189A1 | 2.15E-08 | 7.1691384 | down |
| EPB42 | 3.49E-08 | 7.170591 | down |
| CD96 | 1.07E-06 | 7.1707377 | down |
| ADAMTS3 | 6.92E-08 | 7.177059 | down |
| PIGR | 3.74E-07 | 7.1852694 | down |
| MAL2 | 4.79E-09 | 7.198837 | down |
| SLC48A1 | 2.69E-08 | 7.226373 | down |
| ANKRD12 | 3.33E-08 | 7.2355876 | down |
| AIM1 | 1.26E-08 | 7.2484245 | down |
| CHRDL1 | 1.79E-08 | 7.252875 | down |
| HNF1A | 2.08E-07 | 7.278123 | down |
| XPOT | 1.41E-06 | 7.2798285 | down |
| ARHGAP42 | 9.45E-08 | 7.2800403 | down |
| FAM133A | 1.13E-06 | 7.288691 | down |
| SKAP1 | 6.19E-08 | 7.295215 | down |
| TMEM45B | 8.02E-08 | 7.2973337 | down |
| BFSP1 | 6.26E-09 | 7.297516 | down |
| ATP2A1 | 8.03E-08 | 7.3002515 | down |
| TMEM56 | 1.57E-07 | 7.3061666 | down |
| HLA-DPB1 | 1.55E-08 | 7.3219295 | down |
| TWIST2 | 2.95E-09 | 7.3276987 | down |
| ETNK1 | 2.51E-08 | 7.3367333 | down |
| LCE1E | 2.35E-08 | 7.346817 | down |
| PDCD4 | 7.50E-09 | 7.367357 | down |
| NGEF | 6.54E-09 | 7.389045 | down |
| CHRNB2 | 3.46E-07 | 7.395099 | down |
| LONRF2 | 6.67E-07 | 7.397203 | down |
| OBSCN | 4.39E-08 | 7.4057846 | down |
| NSRP1 | 2.28E-08 | 7.4069853 | down |
| C2orf70 | 1.56E-07 | 7.4238377 | down |
| SGCE | 1.89E-08 | 7.4504 | down |
| STX17-AS1 | 6.86E-09 | 7.4531956 | down |
| FXYD2 | 2.31E-08 | 7.4911075 | down |
| APOL6 | 3.57E-07 | 7.4928236 | down |
| GOLGA8A | 2.60E-08 | 7.5364275 | down |
| LACTB2 | 1.38E-08 | 7.562068 | down |
| MAP3K12 | 1.45E-07 | 7.5698776 | down |
| LATS2 | 8.89E-08 | 7.585259 | down |
| ZDBF2 | 2.42E-08 | 7.5881076 | down |
| VSTM2A | 4.06E-07 | 7.5956154 | down |
| FAXC | 5.35E-09 | 7.630618 | down |
| LRRC61 | 5.77E-09 | 7.6321034 | down |
| KLRG1 | 3.36E-08 | 7.655025 | down |
| NFKBIZ | 6.67E-08 | 7.6628647 | down |
| GDPD3 | 3.42E-08 | 7.6719894 | down |
| BIVM | 4.30E-09 | 7.6804037 | down |
| NFAT5 | 7.77E-09 | 7.6930027 | down |
| WHAMMP3 | 4.85E-07 | 7.7149796 | down |
| MGAT4A | 5.94E-07 | 7.732482 | down |
| GOLGA8A | 5.44E-08 | 7.7573943 | down |
| TRIM9 | 3.18E-07 | 7.7753987 | down |
| LAMA2 | 4.06E-08 | 7.785976 | down |
| GTF2IRD2B | 1.01E-06 | 7.8416257 | down |
| ADTRP | 8.71E-08 | 7.865816 | down |
| XLOC_l2_009136 | 1.35E-06 | 7.899079 | down |
| ARRDC3 | 6.84E-08 | 7.9070683 | down |
| LOC101928958 | 6.33E-07 | 7.9071546 | down |
| C10orf54 | 4.05E-09 | 7.914843 | down |
| SNHG12 | 2.36E-09 | 7.924334 | down |
| RARRES3 | 6.22E-09 | 7.9290423 | down |
| SULT2B1 | 5.60E-08 | 7.9316335 | down |
| LAMC3 | 2.38E-07 | 7.9329467 | down |
| COBL | 1.33E-07 | 7.9379745 | down |
| ELOVL7 | 1.30E-07 | 7.95154 | down |
| ABCC9 | 1.40E-07 | 7.9639177 | down |
| HLA-DPB1 | 3.61E-09 | 7.98352 | down |
| ACSM3 | 2.22E-07 | 7.9845333 | down |
| SORBS2 | 3.85E-07 | 7.9942245 | down |
| SPEF2 | 2.55E-07 | 7.9984584 | down |
| CREG1 | 5.33E-09 | 7.9990797 | down |
| GPATCH4 | 8.88E-07 | 8.007854 | down |
| TUBBP5 | 4.77E-07 | 8.018416 | down |
| C5orf38 | 3.34E-08 | 8.057824 | down |
| IL22RA1 | 1.80E-08 | 8.067759 | down |
| CLIP4 | 1.76E-07 | 8.100399 | down |
| WNT3 | 2.79E-07 | 8.109499 | down |
| LOC440028 | 4.91E-09 | 8.125183 | down |
| KAZALD1 | 8.92E-08 | 8.14068 | down |
| KISS1R | 2.49E-07 | 8.141722 | down |
| HERC5 | 1.62E-08 | 8.148515 | down |
| RNLS | 1.13E-07 | 8.1596365 | down |
| MYH3 | 2.25E-07 | 8.1634655 | down |
| DNHD1 | 2.75E-07 | 8.165294 | down |
| ITPR1 | 3.88E-08 | 8.17521 | down |
| MORN3 | 8.00E-08 | 8.1847725 | down |
| CFAP43 | 9.13E-07 | 8.195993 | down |
| SPATA17 | 9.48E-07 | 8.205435 | down |
| C5 | 5.78E-09 | 8.2224045 | down |
| APOE | 1.00E-08 | 8.256656 | down |
| ANKMY1 | 1.86E-07 | 8.269245 | down |
| COL20A1 | 7.68E-08 | 8.280454 | down |
| LRRC36 | 4.81E-07 | 8.287345 | down |
| GAS5 | 1.11E-07 | 8.290915 | down |
| RASAL1 | 4.73E-08 | 8.309946 | down |
| ENO3 | 1.09E-08 | 8.314254 | down |
| FRY | 1.14E-07 | 8.318481 | down |
| TMEM8C | 1.46E-08 | 8.325435 | down |
| NSRP1 | 1.51E-08 | 8.327249 | down |
| KL | 4.30E-08 | 8.331986 | down |
| FOXD4 | 5.33E-07 | 8.332973 | down |
| KIF21A | 1.01E-06 | 8.335215 | down |
| MAPK10 | 1.19E-08 | 8.350182 | down |
| EMP1 | 4.84E-09 | 8.363301 | down |
| HERC2P2 | 4.12E-07 | 8.363766 | down |
| GPRC5B | 1.54E-08 | 8.379993 | down |
| KRT86 | 6.92E-08 | 8.384306 | down |
| RAD50 | 5.00E-09 | 8.39018 | down |
| ARMC3 | 2.34E-07 | 8.411782 | down |
| XKR7 | 6.60E-07 | 8.412681 | down |
| LRRD1 | 3.77E-09 | 8.42476 | down |
| PRRG4 | 3.95E-08 | 8.44876 | down |
| EHHADH | 3.95E-08 | 8.466581 | down |
| CX3CL1 | 7.30E-08 | 8.472231 | down |
| FAM13A | 4.41E-08 | 8.476322 | down |
| DHX58 | 1.40E-08 | 8.487634 | down |
| DTX3 | 2.40E-08 | 8.505826 | down |
| lnc-KIAA0087-2 | 5.22E-07 | 8.525388 | down |
| HESX1 | 3.30E-07 | 8.544203 | down |
| SYT1 | 1.49E-08 | 8.549076 | down |
| PAX6 | 1.73E-08 | 8.594295 | down |
| MGST1 | 1.17E-08 | 8.601858 | down |
| BTG4 | 5.13E-08 | 8.602923 | down |
| PKP2 | 4.33E-09 | 8.613732 | down |
| FOXQ1 | 2.55E-09 | 8.643848 | down |
| EBF4 | 1.22E-08 | 8.650824 | down |
| MORN4 | 2.38E-09 | 8.658978 | down |
| WDR78 | 3.44E-08 | 8.663659 | down |
| CIART | 2.20E-07 | 8.673841 | down |
| C5orf58 | 3.44E-08 | 8.6999855 | down |
| GATAD1 | 3.72E-09 | 8.701502 | down |
| CERKL | 3.39E-08 | 8.709058 | down |
| lnc-SEC61G-7 | 1.85E-08 | 8.715531 | down |
| CASP10 | 5.14E-09 | 8.716641 | down |
| CAV2 | 3.39E-08 | 8.723557 | down |
| NAMPT | 5.91E-09 | 8.73045 | down |
| LOXL4 | 2.49E-08 | 8.785339 | down |
| WDR63 | 2.04E-07 | 8.785876 | down |
| IL17C | 3.91E-07 | 8.791401 | down |
| FGF12 | 2.58E-07 | 8.823192 | down |
| L1CAM | 8.38E-08 | 8.847532 | down |
| KAZALD1 | 5.13E-08 | 8.857879 | down |
| ESYT2 | 1.52E-08 | 8.861648 | down |
| NFIB | 6.78E-07 | 8.869779 | down |
| SLC19A3 | 8.73E-09 | 8.874515 | down |
| PAPLN | 2.56E-07 | 8.875075 | down |
| TIMP4 | 1.01E-08 | 8.963606 | down |
| ACSS3 | 4.92E-09 | 8.984125 | down |
| TMEM144 | 2.46E-09 | 8.989837 | down |
| GABPB1-AS1 | 4.55E-07 | 9.000662 | down |
| SHISA9 | 1.92E-08 | 9.004301 | down |
| ARHGEF38 | 5.07E-07 | 9.005855 | down |
| MAEL | 3.97E-08 | 9.013147 | down |
| UTP11L | 5.14E-08 | 9.028854 | down |
| COL12A1 | 1.22E-08 | 9.04206 | down |
| BATF3 | 3.03E-09 | 9.092173 | down |
| HLA-F | 8.17E-08 | 9.093075 | down |
| GBA3 | 1.06E-06 | 9.116198 | down |
| MDGA2 | 6.60E-09 | 9.116419 | down |
| PRSS36 | 2.83E-08 | 9.117819 | down |
| LMO1 | 9.71E-08 | 9.130773 | down |
| SPATA6L | 5.54E-07 | 9.134705 | down |
| GSG1L | 3.61E-08 | 9.180554 | down |
| TOX2 | 2.55E-09 | 9.199413 | down |
| MOCOS | 6.27E-09 | 9.199984 | down |
| WNT4 | 7.97E-08 | 9.218484 | down |
| DOCK11 | 1.86E-09 | 9.227396 | down |
| FAM183A | 2.54E-09 | 9.237441 | down |
| AMT | 4.86E-09 | 9.245808 | down |
| FIGNL2 | 5.27E-09 | 9.280198 | down |
| TNFAIP2 | 2.63E-09 | 9.280677 | down |
| JSRP1 | 3.58E-07 | 9.31468 | down |
| RAB39B | 2.05E-07 | 9.319052 | down |
| SOD2 | 7.36E-09 | 9.328696 | down |
| lnc-TMED5-1 | 4.95E-08 | 9.354103 | down |
| lnc-RNF208-1 | 4.26E-09 | 9.370138 | down |
| NPHS1 | 1.15E-07 | 9.396584 | down |
| NDST3 | 1.96E-07 | 9.399562 | down |
| SLC16A4 | 2.89E-09 | 9.408968 | down |
| RAB3IL1 | 4.79E-07 | 9.425829 | down |
| ABCA12 | 1.95E-08 | 9.437038 | down |
| ITGB8 | 2.02E-08 | 9.463654 | down |
| PCDHB8 | 1.15E-08 | 9.474451 | down |
| TMEM139 | 1.70E-09 | 9.493153 | down |
| CKMT1A | 5.72E-08 | 9.506702 | down |
| P2RX7 | 4.32E-07 | 9.523541 | down |
| FAM90A1 | 1.36E-08 | 9.538456 | down |
| GOLGA6C | 1.53E-07 | 9.540886 | down |
| SEMA4D | 3.84E-07 | 9.552251 | down |
| KRTAP19-2 | 2.56E-08 | 9.559769 | down |
| ACACB | 4.40E-08 | 9.572308 | down |
| HNRNPA1 | 6.81E-09 | 9.591592 | down |
| CACNA1A | 1.43E-06 | 9.591753 | down |
| SPATA21 | 5.57E-08 | 9.604559 | down |
| CHRFAM7A | 9.38E-09 | 9.616158 | down |
| ENTPD1 | 3.07E-07 | 9.646805 | down |
| C10orf11 | 1.49E-09 | 9.651108 | down |
| SQRDL | 1.35E-08 | 9.653444 | down |
| KLRG2 | 2.83E-07 | 9.721691 | down |
| DTX2 | 1.27E-09 | 9.744384 | down |
| NSUN7 | 1.87E-09 | 9.748438 | down |
| CRYM | 9.53E-09 | 9.756051 | down |
| FXYD2 | 4.57E-09 | 9.807986 | down |
| ZC3H6 | 1.60E-09 | 9.861618 | down |
| MYH7B | 3.60E-07 | 9.8640785 | down |
| SOSTDC1 | 4.30E-08 | 9.867044 | down |
| COX19 | 2.35E-09 | 9.885105 | down |
| TRIM9 | 3.00E-07 | 9.887004 | down |
| GPR160 | 4.05E-08 | 9.890151 | down |
| ADRB2 | 9.70E-10 | 9.929417 | down |
| LOC100652758 | 7.28E-07 | 9.9305105 | down |
| CHRNB1 | 1.93E-08 | 9.931307 | down |
| CNNM1 | 4.33E-07 | 9.971032 | down |
| PNCK | 1.08E-06 | 9.981297 | down |
| ENKUR | 1.73E-07 | 10.001667 | down |
| HAAO | 7.69E-09 | 10.020102 | down |
| PAXIP1-AS2 | 6.27E-08 | 10.034897 | down |
| C11orf54 | 9.35E-09 | 10.093735 | down |
| AREG | 3.07E-08 | 10.112617 | down |
| SLC48A1 | 9.70E-10 | 10.129703 | down |
| CEP162 | 1.90E-08 | 10.134681 | down |
| SLC2A2 | 1.10E-07 | 10.157026 | down |
| FAM81A | 1.20E-06 | 10.192244 | down |
| MAP3K7CL | 4.24E-07 | 10.207674 | down |
| EPN3 | 4.99E-08 | 10.223694 | down |
| ZDHHC11 | 8.27E-07 | 10.227516 | down |
| LINC01405 | 1.29E-07 | 10.236037 | down |
| TIMD4 | 2.39E-07 | 10.245795 | down |
| PRSS16 | 1.66E-08 | 10.312248 | down |
| AHSA2 | 5.08E-09 | 10.323968 | down |
| ITIH5 | 7.09E-08 | 10.330235 | down |
| SLC16A7 | 1.03E-06 | 10.390306 | down |
| ZDHHC11 | 5.48E-09 | 10.39942 | down |
| AKR1C3 | 2.57E-09 | 10.414461 | down |
| TBC1D8 | 5.15E-07 | 10.440766 | down |
| CLDN3 | 7.78E-09 | 10.470217 | down |
| FOXE1 | 7.23E-07 | 10.502795 | down |
| SLC4A10 | 5.44E-08 | 10.515774 | down |
| AREG | 1.59E-07 | 10.530914 | down |
| ARHGAP9 | 4.03E-07 | 10.548101 | down |
| PILRA | 6.07E-09 | 10.585636 | down |
| ZFYVE28 | 1.35E-08 | 10.61009 | down |
| LAMA3 | 4.28E-08 | 10.610587 | down |
| IQCD | 4.55E-08 | 10.610826 | down |
| RND2 | 5.98E-07 | 10.62072 | down |
| KIAA1644 | 1.17E-07 | 10.642204 | down |
| CA2 | 7.62E-09 | 10.653746 | down |
| HES2 | 2.80E-09 | 10.660063 | down |
| LAT2 | 1.90E-08 | 10.711355 | down |
| PATL2 | 8.92E-07 | 10.716476 | down |
| C4orf19 | 9.00E-08 | 10.717884 | down |
| SSPN | 1.66E-09 | 10.72567 | down |
| LINC-PINT | 8.96E-09 | 10.746725 | down |
| HES7 | 3.02E-07 | 10.749856 | down |
| HOTAIR | 6.47E-08 | 10.750935 | down |
| CEP19 | 5.72E-08 | 10.755535 | down |
| PLCB1 | 4.25E-08 | 10.811069 | down |
| IFIT2 | 1.33E-07 | 10.856954 | down |
| MAB21L2 | 1.55E-07 | 10.87012 | down |
| NFIB | 1.61E-07 | 10.882864 | down |
| LOC100130502 | 4.46E-08 | 10.897118 | down |
| FAM66C | 6.64E-08 | 10.916846 | down |
| ULK4P1 | 1.11E-08 | 10.920543 | down |
| LAT | 1.39E-09 | 10.927293 | down |
| CTSK | 1.30E-08 | 10.93846 | down |
| SLCO4C1 | 1.19E-06 | 10.9428 | down |
| LMNTD2 | 1.69E-09 | 10.96119 | down |
| PCDH20 | 6.91E-08 | 10.979525 | down |
| ATP6AP1L | 1.46E-08 | 10.989353 | down |
| PTPRD | 2.91E-09 | 11.01074 | down |
| GBP2 | 1.24E-08 | 11.088305 | down |
| SEMA3C | 1.90E-07 | 11.168421 | down |
| C1orf194 | 1.03E-08 | 11.169548 | down |
| CHST9 | 3.37E-08 | 11.17184 | down |
| lnc-AP002478,1-1 | 1.22E-08 | 11.195914 | down |
| LOC339192 | 9.24E-09 | 11.209765 | down |
| ACACB | 1.04E-07 | 11.216723 | down |
| CATIP | 5.56E-07 | 11.242991 | down |
| GXYLT1 | 6.98E-08 | 11.249369 | down |
| PRODH | 2.72E-09 | 11.278088 | down |
| TIMD4 | 1.21E-08 | 11.311499 | down |
| DFNB59 | 3.25E-07 | 11.331533 | down |
| CD302 | 1.17E-07 | 11.363481 | down |
| MAATS1 | 8.69E-07 | 11.376208 | down |
| ENKUR | 1.65E-07 | 11.376889 | down |
| MANEAL | 1.18E-08 | 11.386104 | down |
| AKAP9 | 1.46E-09 | 11.466291 | down |
| KIAA1377 | 1.06E-08 | 11.477391 | down |
| FOS | 5.89E-09 | 11.495504 | down |
| NECAB2 | 2.44E-08 | 11.52723 | down |
| BCHE | 2.52E-09 | 11.56983 | down |
| MYO7A | 2.02E-08 | 11.598557 | down |
| LOC283352 | 7.03E-07 | 11.695942 | down |
| RERG | 8.93E-10 | 11.696602 | down |
| FAM66C | 1.93E-09 | 11.734863 | down |
| TSPAN15 | 9.13E-09 | 11.785295 | down |
| CASP8 | 2.32E-09 | 11.787992 | down |
| ZNF862 | 1.50E-08 | 11.790345 | down |
| TRGV7 | 9.44E-08 | 11.872721 | down |
| TNFRSF14 | 1.18E-08 | 11.911657 | down |
| RSPH1 | 7.80E-08 | 11.975367 | down |
| GSTT2B | 1.55E-09 | 12.008932 | down |
| MEIG1 | 5.00E-07 | 12.052906 | down |
| UACA | 2.38E-08 | 12.057749 | down |
| SLC40A1 | 1.46E-09 | 12.097596 | down |
| CFAP53 | 1.09E-06 | 12.097841 | down |
| LINC00304 | 1.19E-07 | 12.146104 | down |
| USP32 | 3.09E-07 | 12.162583 | down |
| C1orf186 | 1.41E-09 | 12.16692 | down |
| CYBRD1 | 2.31E-09 | 12.183913 | down |
| ANKRD26 | 7.14E-07 | 12.22322 | down |
| SHOX2 | 1.78E-08 | 12.250553 | down |
| MGAT5B | 5.21E-07 | 12.252699 | down |
| IFIT2 | 2.86E-08 | 12.29301 | down |
| IL7 | 4.00E-07 | 12.301673 | down |
| KIAA1211L | 1.89E-08 | 12.34673 | down |
| PTK2B | 2.10E-08 | 12.397391 | down |
| SLC13A3 | 5.64E-09 | 12.43862 | down |
| DAW1 | 2.31E-09 | 12.475558 | down |
| HLA-DMA | 1.48E-08 | 12.489492 | down |
| RAB17 | 2.68E-08 | 12.601198 | down |
| CPLX1 | 4.28E-09 | 12.663524 | down |
| RIC3 | 2.37E-07 | 12.76761 | down |
| TNFAIP6 | 1.78E-09 | 12.837104 | down |
| APBB1IP | 2.77E-07 | 12.930275 | down |
| METTL7A | 1.09E-06 | 12.977438 | down |
| LOC401320 | 9.10E-07 | 13.076017 | down |
| MR1 | 1.27E-07 | 13.077163 | down |
| CFAP70 | 1.14E-07 | 13.140368 | down |
| ALDH3B1 | 7.97E-09 | 13.156933 | down |
| PPIL6 | 1.41E-07 | 13.16672 | down |
| GPR162 | 4.65E-09 | 13.193487 | down |
| EFCAB6 | 2.54E-08 | 13.23372 | down |
| TMIE | 2.84E-07 | 13.233864 | down |
| ANKS1B | 8.12E-08 | 13.337631 | down |
| RNF32 | 9.14E-07 | 13.342645 | down |
| LINC00477 | 1.69E-08 | 13.41669 | down |
| ABHD1 | 5.11E-08 | 13.433987 | down |
| RGS11 | 1.38E-08 | 13.462203 | down |
| C1orf27 | 2.48E-07 | 13.497304 | down |
| DPCR1 | 2.35E-08 | 13.5219 | down |
| ERICH2 | 3.14E-07 | 13.539701 | down |
| FAM47E | 1.52E-07 | 13.60926 | down |
| PTPRO | 1.53E-07 | 13.612467 | down |
| LOC100133669 | 7.67E-07 | 13.615599 | down |
| GOLGA7B | 1.20E-06 | 13.6234 | down |
| SRRM3 | 5.53E-07 | 13.755968 | down |
| ZMAT1 | 2.79E-09 | 13.818563 | down |
| NFIB | 1.76E-08 | 13.850648 | down |
| C12orf56 | 1.69E-08 | 13.85622 | down |
| ZMYND12 | 2.43E-07 | 13.946901 | down |
| GOLT1A | 2.86E-09 | 14.017391 | down |
| PAQR6 | 8.62E-09 | 14.060379 | down |
| SLC6A15 | 7.09E-07 | 14.106105 | down |
| LOC440173 | 1.81E-08 | 14.171665 | down |
| GTF2IRD2B | 1.25E-07 | 14.217967 | down |
| DDIT4 | 2.00E-07 | 14.249317 | down |
| PKP3 | 5.18E-09 | 14.273475 | down |
| CALCRL | 5.84E-07 | 14.341992 | down |
| APBB1IP | 2.83E-08 | 14.458037 | down |
| ACSL6 | 2.76E-08 | 14.481295 | down |
| MKX | 3.51E-07 | 14.594853 | down |
| PILRB | 8.05E-10 | 14.645122 | down |
| TFPI | 6.39E-10 | 14.755178 | down |
| lnc-JMJD7-PLA2G4B-1 | 4.47E-08 | 14.800458 | down |
| KLRF1 | 3.59E-08 | 14.843299 | down |
| CYP3A7 | 3.40E-08 | 14.922056 | down |
| DCAF12L1 | 1.19E-08 | 15.011521 | down |
| NAMPT | 1.79E-09 | 15.079358 | down |
| MNX1 | 1.00E-09 | 15.098404 | down |
| GCNT3 | 3.59E-07 | 15.109649 | down |
| ANKRD29 | 8.39E-10 | 15.199131 | down |
| CEBPA | 5.89E-08 | 15.286843 | down |
| CEP290 | 2.13E-09 | 15.560983 | down |
| GSAP | 1.46E-08 | 15.652812 | down |
| HAVCR1 | 2.02E-08 | 15.679779 | down |
| BIRC3 | 1.40E-09 | 15.744205 | down |
| RGL3 | 1.26E-07 | 15.815599 | down |
| SCN9A | 1.28E-08 | 15.834085 | down |
| TNNC1 | 7.83E-08 | 15.837701 | down |
| GTF2IRD2 | 2.60E-08 | 15.955568 | down |
| NDUFA4L2 | 6.81E-08 | 16.029684 | down |
| CASP4 | 2.27E-09 | 16.106361 | down |
| C15orf48 | 2.27E-09 | 16.15271 | down |
| B4GALNT1 | 2.91E-07 | 16.193726 | down |
| NID2 | 1.80E-09 | 16.234047 | down |
| OVOS2 | 4.66E-07 | 16.261219 | down |
| RSPH14 | 2.10E-09 | 16.309572 | down |
| KIAA1407 | 6.19E-07 | 16.388687 | down |
| SPX | 1.50E-08 | 16.425356 | down |
| TNNT1 | 7.63E-09 | 16.464508 | down |
| PCDH9 | 1.71E-08 | 16.50259 | down |
| SLFN13 | 4.01E-08 | 16.506763 | down |
| PLXNC1 | 1.16E-08 | 16.559807 | down |
| FABP3 | 7.73E-07 | 16.603739 | down |
| HGD | 2.01E-09 | 16.60722 | down |
| CASP5 | 4.82E-10 | 16.637117 | down |
| NKX3-2 | 2.62E-08 | 16.8833 | down |
| NOXA1 | 2.20E-08 | 16.90028 | down |
| CXCL1 | 2.64E-09 | 16.913652 | down |
| C17orf67 | 3.09E-07 | 17.064177 | down |
| TTC25 | 1.42E-07 | 17.067802 | down |
| SHISA9 | 6.65E-09 | 17.122192 | down |
| ERBB3 | 4.59E-08 | 17.185976 | down |
| MADCAM1 | 1.21E-07 | 17.285658 | down |
| ZP3 | 2.11E-09 | 17.334867 | down |
| CLU | 1.43E-09 | 17.349243 | down |
| MROH8 | 8.03E-07 | 17.399174 | down |
| ENTPD1 | 9.39E-09 | 17.414719 | down |
| HLA-DPB1 | 3.50E-09 | 17.471207 | down |
| KCNJ13 | 2.78E-09 | 17.536638 | down |
| lnc-RP3-377D14,1,1-3 | 2.67E-08 | 17.547754 | down |
| TM7SF3 | 1.03E-08 | 17.56024 | down |
| ASS1 | 1.52E-09 | 17.583057 | down |
| PCDHB9 | 1.05E-08 | 17.62442 | down |
| LOC101927418 | 5.32E-07 | 17.730026 | down |
| CXCL3 | 1.35E-08 | 17.74086 | down |
| ASS1 | 3.41E-08 | 17.877928 | down |
| COL14A1 | 1.16E-06 | 17.926483 | down |
| KLHDC1 | 1.69E-07 | 17.95339 | down |
| PRAME | 1.22E-09 | 18.195536 | down |
| ABCC3 | 1.69E-09 | 18.27827 | down |
| SEC31B | 9.20E-09 | 18.27914 | down |
| TFPI | 2.94E-09 | 18.292467 | down |
| ADAMTS18 | 2.14E-07 | 18.360664 | down |
| SLC3A1 | 4.15E-08 | 18.656675 | down |
| RNF144B | 2.10E-09 | 18.688448 | down |
| C10orf107 | 1.67E-07 | 18.712015 | down |
| CYP3A7 | 6.24E-08 | 18.737495 | down |
| VAC14-AS1 | 1.61E-08 | 18.825127 | down |
| ANXA13 | 3.61E-09 | 18.895435 | down |
| ULK4 | 4.51E-09 | 18.977495 | down |
| ANGPTL7 | 3.24E-09 | 19.323092 | down |
| CASC2 | 2.43E-08 | 19.44649 | down |
| ULBP1 | 3.32E-09 | 19.47456 | down |
| SEMA4D | 1.73E-08 | 19.47978 | down |
| SPX | 8.64E-08 | 19.498913 | down |
| NTRK3 | 2.08E-08 | 19.508568 | down |
| AHI1 | 7.71E-08 | 19.53104 | down |
| EDN2 | 3.20E-10 | 19.760836 | down |
| CDHR1 | 3.77E-08 | 19.90855 | down |
| ANKRD2 | 1.23E-09 | 20.26199 | down |
| ITGB6 | 3.45E-08 | 20.354378 | down |
| RSPH1 | 1.26E-08 | 20.368422 | down |
| ELOVL2 | 1.19E-09 | 20.479847 | down |
| SOWAHD | 5.15E-09 | 20.529434 | down |
| UCN | 2.07E-08 | 20.535814 | down |
| DRC1 | 3.36E-09 | 20.63987 | down |
| AK7 | 4.35E-08 | 20.698656 | down |
| C11orf54 | 4.30E-09 | 20.704868 | down |
| CFAP43 | 1.02E-08 | 20.765219 | down |
| GNGT1 | 8.03E-09 | 20.814302 | down |
| MACC1 | 1.40E-08 | 20.86119 | down |
| AMY1C | 5.56E-09 | 20.888857 | down |
| OAS1 | 7.22E-09 | 21.257868 | down |
| CFAP221 | 1.17E-08 | 21.271833 | down |
| ULBP1 | 4.84E-09 | 21.295414 | down |
| PABPC1L | 1.22E-09 | 21.31644 | down |
| SLC52A1 | 4.50E-10 | 21.396309 | down |
| FOXA1 | 3.53E-07 | 21.397179 | down |
| TPPP3 | 1.57E-08 | 21.508352 | down |
| RGS5 | 1.01E-09 | 21.547699 | down |
| CXCL1 | 5.73E-10 | 21.641228 | down |
| IRF5 | 5.39E-10 | 21.875374 | down |
| RASGEF1B | 7.73E-07 | 21.909266 | down |
| ERICH2 | 1.46E-09 | 22.115225 | down |
| CASC1 | 9.70E-09 | 22.219515 | down |
| CHGB | 1.57E-07 | 22.760231 | down |
| ANO2 | 8.74E-10 | 22.90228 | down |
| NXPH2 | 5.84E-09 | 23.006182 | down |
| C1R | 1.12E-09 | 23.079014 | down |
| DPYD | 5.82E-09 | 23.314764 | down |
| DSCAML1 | 1.68E-08 | 23.349527 | down |
| WBSCR27 | 5.84E-10 | 23.380804 | down |
| CCNYL2 | 2.19E-08 | 23.653458 | down |
| PDZRN4 | 6.11E-09 | 23.68428 | down |
| KLHDC7B | 1.09E-07 | 23.800089 | down |
| C1R | 5.91E-09 | 23.878038 | down |
| VNN2 | 3.17E-08 | 24.220984 | down |
| SCIN | 5.50E-09 | 24.27898 | down |
| SYTL2 | 3.68E-10 | 24.595179 | down |
| MTHFD2P1 | 9.86E-09 | 24.643082 | down |
| LBX2 | 2.69E-09 | 24.676287 | down |
| CRIP1 | 3.55E-10 | 24.74916 | down |
| FAM110C | 1.07E-09 | 24.77161 | down |
| CASC2 | 1.08E-07 | 24.81688 | down |
| HOGA1 | 2.25E-09 | 24.960382 | down |
| SLC47A1 | 1.74E-08 | 25.030191 | down |
| WFDC21P | 5.76E-09 | 25.306934 | down |
| VAV3 | 6.63E-10 | 25.369024 | down |
| PDCD4-AS1 | 3.87E-09 | 25.439539 | down |
| NFATC1 | 3.83E-07 | 25.443634 | down |
| GRIN2C | 3.37E-08 | 25.530426 | down |
| C2orf82 | 8.45E-10 | 25.559841 | down |
| SLC3A1 | 2.85E-07 | 25.7313 | down |
| LINC00864 | 3.59E-09 | 25.985167 | down |
| ERAP1 | 4.04E-08 | 26.04918 | down |
| TFPI | 2.65E-09 | 26.052132 | down |
| KRT222 | 3.93E-09 | 26.096447 | down |
| FBP1 | 4.92E-07 | 26.352053 | down |
| CEP83 | 7.49E-08 | 26.362236 | down |
| SCN9A | 1.05E-07 | 26.416618 | down |
| SLC6A15 | 1.20E-07 | 26.513256 | down |
| HLA-DPA1 | 3.18E-10 | 26.802366 | down |
| SPIRE2 | 1.49E-08 | 26.87499 | down |
| TRABD2A | 2.37E-07 | 27.06367 | down |
| LOC441204 | 1.15E-06 | 27.222694 | down |
| ABCC9 | 1.52E-08 | 27.292849 | down |
| PTPRD-AS1 | 1.91E-08 | 27.3784 | down |
| STAT4 | 1.58E-07 | 27.582745 | down |
| ZIC5 | 8.23E-08 | 27.969717 | down |
| PLEKHG4 | 1.05E-09 | 28.08761 | down |
| PPFIBP2 | 5.62E-07 | 28.153555 | down |
| MGAT4C | 6.17E-09 | 28.219597 | down |
| lnc-GMDS-2 | 3.75E-09 | 28.235682 | down |
| GLS2 | 3.88E-09 | 28.315512 | down |
| MGAT4C | 3.01E-07 | 28.610643 | down |
| LOC100132057 | 2.26E-07 | 28.73215 | down |
| SPATA41 | 5.93E-08 | 28.797901 | down |
| SAMD9 | 9.93E-08 | 29.067034 | down |
| MDH1B | 6.81E-08 | 29.068878 | down |
| FRY | 6.87E-10 | 29.115175 | down |
| EXOC3L4 | 1.46E-07 | 29.128796 | down |
| HLA-DMA | 1.16E-06 | 29.590986 | down |
| RASGRP2 | 4.86E-09 | 29.806467 | down |
| RASGRP2 | 2.93E-09 | 29.806704 | down |
| CYBRD1 | 2.10E-09 | 29.844852 | down |
| PRDM16 | 1.04E-08 | 30.10441 | down |
| KCNC1 | 1.42E-08 | 30.284134 | down |
| KCNG3 | 5.15E-08 | 30.408457 | down |
| CAPS2 | 2.02E-08 | 30.661095 | down |
| C6orf58 | 8.93E-08 | 30.750689 | down |
| NRCAM | 4.41E-08 | 30.811527 | down |
| OGDHL | 5.15E-10 | 30.970682 | down |
| DNAJC12 | 2.71E-07 | 31.187342 | down |
| CCBE1 | 9.88E-07 | 31.547567 | down |
| DDIT3 | 1.94E-10 | 31.892427 | down |
| VSTM2L | 5.24E-07 | 31.92166 | down |
| IFI16 | 5.33E-09 | 32.008144 | down |
| ITGB4 | 1.80E-09 | 32.054356 | down |
| SLC4A10 | 7.29E-09 | 32.194595 | down |
| EBF3 | 3.55E-10 | 32.32159 | down |
| ALOX5 | 3.12E-10 | 32.627888 | down |
| MT1DP | 1.15E-08 | 32.748646 | down |
| CLRN3 | 1.31E-08 | 33.062347 | down |
| MAATS1 | 1.06E-08 | 33.28816 | down |
| PLEKHG4 | 8.49E-10 | 33.406746 | down |
| TNFSF14 | 5.97E-09 | 33.620117 | down |
| EREG | 9.52E-09 | 33.7053 | down |
| ZMAT1 | 1.55E-09 | 33.78661 | down |
| AGBL2 | 1.49E-08 | 33.827293 | down |
| DLX1 | 1.98E-08 | 34.07825 | down |
| THNSL2 | 1.79E-09 | 34.141483 | down |
| LRRIQ1 | 4.93E-09 | 34.40216 | down |
| PIP5K1B | 4.61E-09 | 34.58046 | down |
| PRKCE | 3.43E-09 | 35.216515 | down |
| TRIM67 | 1.26E-06 | 35.472572 | down |
| TMEM130 | 6.57E-10 | 35.508244 | down |
| DDO | 2.82E-08 | 35.57625 | down |
| LOC101928569 | 7.21E-08 | 35.797703 | down |
| CPS1 | 1.99E-10 | 36.160816 | down |
| LOC388282 | 6.50E-10 | 36.30359 | down |
| ODF3B | 1.63E-09 | 36.35565 | down |
| STAT4 | 4.99E-09 | 36.54354 | down |
| TMEM255A | 8.06E-08 | 37.44646 | down |
| RHBDL2 | 1.72E-09 | 38.664875 | down |
| PDZRN4 | 1.60E-08 | 38.781048 | down |
| LOC100287387 | 3.50E-07 | 39.000698 | down |
| DLL1 | 4.41E-08 | 39.038406 | down |
| CLUHP3 | 7.20E-07 | 39.276897 | down |
| RASGRP2 | 2.04E-07 | 39.32931 | down |
| WFDC2 | 2.31E-10 | 39.77611 | down |
| PAX6 | 8.32E-10 | 40.063877 | down |
| KCNC1 | 3.21E-09 | 40.406498 | down |
| MANSC1 | 1.22E-07 | 40.97112 | down |
| HYAL1 | 6.33E-09 | 41.12515 | down |
| EFCAB10 | 1.64E-09 | 41.31138 | down |
| SLC17A3 | 4.89E-08 | 42.84661 | down |
| SOX2 | 2.27E-09 | 43.4763 | down |
| C3 | 2.37E-10 | 43.492958 | down |
| NLGN1 | 7.20E-09 | 43.830322 | down |
| NKX2-4 | 4.91E-07 | 44.65232 | down |
| CDH16 | 4.64E-10 | 45.216034 | down |
| CAPS2 | 5.95E-09 | 45.277485 | down |
| EFHB | 4.32E-08 | 45.57383 | down |
| GPM6A | 3.77E-09 | 46.410667 | down |
| VSX1 | 2.47E-09 | 46.44882 | down |
| RELN | 1.39E-08 | 46.984715 | down |
| C8orf31 | 2.47E-08 | 47.395653 | down |
| CRIP1 | 4.34E-10 | 47.483253 | down |
| NLGN1 | 2.13E-07 | 47.69552 | down |
| SAA4 | 2.41E-10 | 47.88241 | down |
| SP8 | 1.25E-09 | 48.03302 | down |
| PHF21B | 1.02E-07 | 48.325977 | down |
| FGFR2 | 4.55E-08 | 48.616947 | down |
| RELN | 8.78E-09 | 49.76979 | down |
| GOLGA6L6 | 1.18E-07 | 49.8258 | down |
| SAA2 | 8.21E-09 | 49.860035 | down |
| TSPAN8 | 1.80E-09 | 51.02195 | down |
| SLC35F4 | 4.45E-09 | 52.165318 | down |
| FABP6 | 2.98E-08 | 52.342274 | down |
| C3 | 6.60E-09 | 53.363293 | down |
| COLCA2 | 2.46E-09 | 54.830765 | down |
| PLSCR4 | 4.03E-07 | 54.85612 | down |
| HLA-DRB5 | 8.61E-08 | 55.350117 | down |
| GAD1 | 1.28E-06 | 55.721172 | down |
| SECTM1 | 5.62E-10 | 56.331234 | down |
| SPAG17 | 3.01E-09 | 56.434902 | down |
| NPY | 4.34E-10 | 57.232937 | down |
| IL6 | 5.84E-11 | 57.337566 | down |
| DLX4 | 6.11E-10 | 57.983486 | down |
| WFDC2 | 3.80E-10 | 58.438835 | down |
| ZBBX | 2.11E-09 | 59.83133 | down |
| CHD5 | 1.75E-09 | 59.86377 | down |
| GATA4 | 5.66E-09 | 60.438644 | down |
| CXCL2 | 5.23E-11 | 60.913734 | down |
| HOXD13 | 2.67E-09 | 62.76816 | down |
| SOD3 | 8.28E-10 | 62.97636 | down |
| PDZK1IP1 | 5.16E-10 | 63.04496 | down |
| RAPGEF3 | 3.22E-08 | 63.278816 | down |
| LINC01124 | 1.60E-09 | 64.773125 | down |
| SGPP2 | 5.27E-07 | 65.302055 | down |
| SRRM3 | 3.51E-08 | 65.48844 | down |
| LPPR5 | 1.88E-09 | 65.51938 | down |
| PDZK1 | 2.29E-08 | 67.00808 | down |
| FGFR2 | 1.35E-09 | 69.97136 | down |
| PTGIS | 3.34E-08 | 70.52726 | down |
| GOLGA6L6 | 1.31E-07 | 71.10238 | down |
| FCAMR | 3.13E-07 | 71.175735 | down |
| CCDC146 | 2.16E-08 | 72.51887 | down |
| C5AR1 | 1.24E-08 | 74.33175 | down |
| SV2B | 2.33E-07 | 74.65155 | down |
| ALDH1A1 | 1.12E-08 | 76.811646 | down |
| SEMA3E | 2.63E-09 | 76.89021 | down |
| SMTNL2 | 6.65E-09 | 77.41427 | down |
| GRIA4 | 1.18E-09 | 78.76826 | down |
| LOC100129055 | 1.61E-09 | 79.288155 | down |
| LINC01018 | 4.38E-10 | 80.19998 | down |
| LOC389332 | 2.08E-07 | 80.55326 | down |
| CPT1B | 1.05E-06 | 80.81609 | down |
| LRRK2 | 1.02E-09 | 81.135925 | down |
| NKD2 | 3.61E-08 | 81.665184 | down |
| CXCL8 | 1.09E-08 | 81.80528 | down |
| TM4SF4 | 8.01E-09 | 83.41804 | down |
| ADAMTS8 | 4.16E-07 | 84.39502 | down |
| TFAP2A | 5.84E-10 | 84.66701 | down |
| LINC01124 | 1.03E-09 | 84.908264 | down |
| MAL | 4.67E-11 | 86.52369 | down |
| CFB | 3.78E-09 | 87.09665 | down |
| MAPT | 3.94E-09 | 88.10559 | down |
| CLDN16 | 4.50E-08 | 91.526375 | down |
| NPNT | 3.81E-07 | 92.73965 | down |
| TSPAN19 | 7.16E-09 | 93.259544 | down |
| EFHB | 2.64E-11 | 94.08367 | down |
| MSR1 | 5.84E-10 | 94.83834 | down |
| EPHA10 | 1.29E-09 | 96.36341 | down |
| HOXA13 | 9.12E-10 | 101.23336 | down |
| SLFN11 | 2.88E-09 | 105.37484 | down |
| ENPP3 | 1.44E-06 | 105.82445 | down |
| LAMP3 | 1.59E-09 | 106.99572 | down |
| CARD17 | 5.58E-08 | 108.63285 | down |
| SAMD9L | 3.41E-08 | 111.32013 | down |
| C1S | 7.68E-08 | 120.72365 | down |
| IL18 | 6.31E-10 | 123.00691 | down |
| SAA2 | 3.14E-10 | 123.27198 | down |
| SYT17 | 1.53E-09 | 124.08992 | down |
| PPP2R2C | 2.29E-07 | 128.22264 | down |
| SLC47A1 | 6.08E-10 | 133.75429 | down |
| CXCR4 | 1.21E-08 | 134.44455 | down |
| QRFPR | 2.13E-09 | 135.95412 | down |
| FOXF2 | 1.56E-08 | 137.59468 | down |
| ACKR4 | 3.01E-10 | 151.2928 | down |
| SDPR | 3.39E-07 | 153.43736 | down |
| RHBDL2 | 3.35E-07 | 162.57365 | down |
| TBX15 | 7.97E-10 | 167.008 | down |
| CCM2L | 8.99E-10 | 170.88846 | down |
| NR1H4 | 3.62E-09 | 172.84332 | down |
| SAA1 | 1.21E-10 | 175.55038 | down |
| LY75 | 1.44E-07 | 179.48726 | down |
| CCDC162P | 6.23E-10 | 206.18568 | down |
| KLHDC7B | 1.20E-07 | 212.05551 | down |
| CXCL2 | 2.59E-11 | 212.30475 | down |
| TM4SF18 | 6.40E-07 | 220.66495 | down |
| GATA5 | 3.65E-10 | 243.26741 | down |
| CRTAC1 | 1.61E-11 | 247.05257 | down |
| PADI3 | 8.65E-07 | 248.61826 | down |
| CXCL14 | 5.05E-11 | 249.2406 | down |
| SP5 | 1.90E-10 | 251.61511 | down |
| DPYSL5 | 2.26E-10 | 262.5679 | down |
| PIP5K1B | 1.13E-09 | 325.0678 | down |
| PLEK | 4.44E-10 | 375.0206 | down |
| AFAP1-AS1 | 8.38E-10 | 375.07092 | down |
| DDC | 2.88E-10 | 375.71884 | down |
| NUPR1 | 3.26E-11 | 451.8799 | down |
| CASP1 | 1.72E-10 | 522.1344 | down |
| CDH1 | 7.27E-11 | 544.15845 | down |
| WISP2 | 3.60E-08 | 581.8608 | down |
| SLC34A2 | 1.54E-10 | 605.1768 | down |
| HOXB13 | 1.29E-10 | 635.8253 | down |
| C1S | 3.71E-09 | 644.703 | down |
| IGFN1 | 1.88E-09 | 748.5548 | down |
| DPYSL5 | 1.08E-10 | 862.9584 | down |
| RARRES2 | 1.17E-10 | 894.8606 | down |
| ITGB2 | 1.99E-10 | 1037.9517 | down |
| ALDH1A1 | 1.53E-11 | 1633.7367 | down |
| LCN2 | 2.99E-11 | 11514.873 | down |
| SAR1A | 9.34E-07 | 2.0002394 | up |
| TBCD | 1.00E-06 | 2.000713 | up |
| KRT18 | 1.04E-06 | 2.0011554 | up |
| TSHB | 1.19E-06 | 2.0032856 | up |
| SLX1A | 8.89E-07 | 2.0084627 | up |
| POMT2 | 8.79E-07 | 2.0097785 | up |
| MCAM | 7.39E-07 | 2.0114324 | up |
| SRP14 | 9.39E-07 | 2.0117507 | up |
| BMS1 | 1.10E-06 | 2.0171282 | up |
| DPH3P1 | 1.11E-06 | 2.0203016 | up |
| AKR7A3 | 8.99E-07 | 2.0203404 | up |
| TSEN34 | 6.17E-07 | 2.0240736 | up |
| UTP11L | 1.11E-06 | 2.0261965 | up |
|  | 1.05E-06 | 2.027156 | up |
| XLOC_l2_015464 | 1.03E-06 | 2.0275247 | up |
| SVIL | 8.38E-07 | 2.0305574 | up |
| MXRA7 | 1.39E-06 | 2.0310297 | up |
| BRK1 | 5.44E-07 | 2.0317326 | up |
| CDK5R2 | 1.32E-06 | 2.0357997 | up |
| RABL2B | 1.20E-06 | 2.0377262 | up |
| POU2F2 | 1.08E-06 | 2.0379887 | up |
| PPP1R2 | 1.28E-06 | 2.0410879 | up |
| CPSF6 | 8.44E-07 | 2.0475152 | up |
| IZUMO1R | 1.18E-06 | 2.0524588 | up |
| KRT18 | 6.73E-07 | 2.0568352 | up |
| PSMG1 | 6.70E-07 | 2.0571878 | up |
| HSPA14 | 6.36E-07 | 2.0576508 | up |
| ALDOA | 1.32E-06 | 2.0584579 | up |
| AOX1 | 1.11E-06 | 2.0587862 | up |
| CDCP1 | 6.94E-07 | 2.0596771 | up |
| BRK1 | 1.14E-06 | 2.0605273 | up |
| PRICKLE2 | 1.20E-06 | 2.0605998 | up |
| NIPAL3 | 1.05E-06 | 2.0612738 | up |
| C6orf62 | 1.44E-06 | 2.0628512 | up |
| PAK4 | 9.60E-07 | 2.0645669 | up |
| MELK | 1.46E-06 | 2.0659242 | up |
| LOC644277 | 1.25E-06 | 2.0662618 | up |
| PARL | 7.31E-07 | 2.069585 | up |
| ARPP19 | 1.03E-06 | 2.069746 | up |
| MAPRE1 | 1.32E-06 | 2.0707316 | up |
| TMEM178A | 8.18E-07 | 2.0718756 | up |
| SREBF1 | 1.37E-06 | 2.073607 | up |
| MRPL21 | 1.16E-06 | 2.0758157 | up |
| ACLY | 1.05E-06 | 2.076187 | up |
| LINC00665 | 1.31E-06 | 2.0768664 | up |
| PIAS3 | 1.15E-06 | 2.0807135 | up |
| ISCA1 | 1.13E-06 | 2.0830562 | up |
| HSPB11 | 8.09E-07 | 2.0860088 | up |
| PPP2R2A | 9.25E-07 | 2.0863912 | up |
| FAM188A | 1.12E-06 | 2.0868192 | up |
| BYSL | 1.07E-06 | 2.0929232 | up |
| TMED3 | 5.15E-07 | 2.09377 | up |
| PSPC1 | 1.05E-06 | 2.0938838 | up |
| ZNF430 | 1.11E-06 | 2.094183 | up |
| ACOT9 | 5.13E-07 | 2.0972922 | up |
| TPCN2 | 9.68E-07 | 2.0983694 | up |
| NUDT22 | 1.25E-06 | 2.1002948 | up |
| GEMIN4 | 5.73E-07 | 2.1028078 | up |
| CD81 | 8.93E-07 | 2.10587 | up |
| MTMR3 | 1.35E-06 | 2.1059418 | up |
| ENO1 | 1.45E-06 | 2.1062145 | up |
| KIAA1191 | 1.14E-06 | 2.1090631 | up |
| LOC338797 | 8.10E-07 | 2.1128123 | up |
| TMEM234 | 1.10E-06 | 2.1156394 | up |
| FANCG | 1.40E-06 | 2.1172752 | up |
| ZNF436 | 9.65E-07 | 2.1212943 | up |
| ADAT3 | 1.20E-06 | 2.1219263 | up |
| VPS4A | 5.52E-07 | 2.1251502 | up |
| TMA7 | 6.60E-07 | 2.1307561 | up |
| LOC100131432 | 7.75E-07 | 2.1307929 | up |
| ATP5I | 6.91E-07 | 2.1318185 | up |
| ALPPL2 | 1.29E-06 | 2.1328824 | up |
| MEG3 | 8.35E-07 | 2.1352692 | up |
| SDHAP1 | 1.45E-06 | 2.1360667 | up |
| CCM2 | 1.41E-06 | 2.1362894 | up |
| EBPL | 4.25E-07 | 2.1372187 | up |
| NOTCH2 | 1.01E-06 | 2.141511 | up |
| PSTK | 1.05E-06 | 2.1443195 | up |
| ICAM3 | 5.81E-07 | 2.1449485 | up |
| MEIS2 | 6.04E-07 | 2.145523 | up |
| AAMDC | 5.07E-07 | 2.1499343 | up |
| NRBP1 | 4.82E-07 | 2.151099 | up |
| TMCC1 | 4.21E-07 | 2.1517873 | up |
| NTMT1 | 1.03E-06 | 2.1590807 | up |
| TNIP1 | 7.59E-07 | 2.1608057 | up |
| BEAN1 | 5.80E-07 | 2.1608405 | up |
| ALDH3B2 | 1.43E-06 | 2.161265 | up |
| SLFN5 | 1.15E-06 | 2.163531 | up |
| MRPL23 | 1.31E-06 | 2.1639981 | up |
| TRAPPC2B | 8.40E-07 | 2.164586 | up |
| TSPO | 9.24E-07 | 2.1647704 | up |
| GAS2L1 | 7.10E-07 | 2.164911 | up |
| METTL22 | 3.74E-07 | 2.1653285 | up |
| BCR | 1.37E-06 | 2.165528 | up |
| COX17 | 1.13E-06 | 2.1656651 | up |
| MRPL3 | 4.68E-07 | 2.1677961 | up |
| TRIM27 | 6.98E-07 | 2.1681702 | up |
| MRPS18C | 5.26E-07 | 2.1731 | up |
| HAUS1 | 1.34E-06 | 2.1738696 | up |
| COX7A2 | 8.63E-07 | 2.174203 | up |
| TMEM104 | 8.66E-07 | 2.1745172 | up |
| BID | 9.96E-07 | 2.1768484 | up |
| MIDN | 8.84E-07 | 2.1784623 | up |
| HMG20B | 7.55E-07 | 2.179338 | up |
| MGST2 | 7.50E-07 | 2.1795022 | up |
| CTPS1 | 7.64E-07 | 2.1820378 | up |
| NAA50 | 9.42E-07 | 2.1825058 | up |
| VDAC1 | 9.74E-07 | 2.1851273 | up |
| SRSF3 | 1.07E-06 | 2.185395 | up |
| SLC25A23 | 5.47E-07 | 2.185661 | up |
| PTRH1 | 3.67E-07 | 2.1859894 | up |
| LOC153811 | 3.97E-07 | 2.18607 | up |
| FN3KRP | 6.70E-07 | 2.1872075 | up |
| UBAP2 | 1.24E-06 | 2.1872344 | up |
| PTDSS2 | 1.41E-06 | 2.1873162 | up |
| CAPRIN1 | 9.12E-07 | 2.1886132 | up |
| MPI | 1.08E-06 | 2.1891153 | up |
| SV2C | 4.08E-07 | 2.1951582 | up |
| SNHG18 | 1.36E-06 | 2.1966884 | up |
| FOXD2 | 4.46E-07 | 2.197462 | up |
| GPX5 | 5.92E-07 | 2.1978543 | up |
| SLC35A4 | 1.06E-06 | 2.1985915 | up |
| FSCN1 | 5.91E-07 | 2.20144 | up |
| ACAP3 | 4.83E-07 | 2.2052014 | up |
| EDARADD | 1.03E-06 | 2.2054574 | up |
| TTLL10-AS1 | 1.05E-06 | 2.2060108 | up |
| PEF1 | 7.12E-07 | 2.2097042 | up |
| XLOC_l2_013410 | 9.61E-07 | 2.2108533 | up |
| EXOSC4 | 4.05E-07 | 2.2139416 | up |
| TMEM145 | 1.08E-06 | 2.2154849 | up |
| MEMO1 | 6.09E-07 | 2.2166772 | up |
| IGFBP7 | 6.78E-07 | 2.2182045 | up |
| SAR1A | 8.87E-07 | 2.221761 | up |
| RPS6KL1 | 8.35E-07 | 2.2225902 | up |
| SIX1 | 9.25E-07 | 2.222749 | up |
| CBWD5 | 6.00E-07 | 2.22337 | up |
| GINS2 | 1.30E-06 | 2.2258794 | up |
| MAFIP | 7.16E-07 | 2.2259727 | up |
| UBE2O | 4.10E-07 | 2.2284503 | up |
| CENPBD1P1 | 5.46E-07 | 2.2298012 | up |
| GAB2 | 1.06E-06 | 2.2309594 | up |
| SNX21 | 7.46E-07 | 2.2310145 | up |
| C8orf59 | 6.23E-07 | 2.2320073 | up |
| C1orf50 | 3.74E-07 | 2.2328076 | up |
| GTF3C5 | 6.42E-07 | 2.233236 | up |
| RBL1 | 1.24E-06 | 2.2333975 | up |
| SNX18 | 1.46E-06 | 2.23614 | up |
| SLC2A5 | 7.79E-07 | 2.2366488 | up |
| NSDHL | 1.15E-06 | 2.2369435 | up |
| PGF | 9.95E-07 | 2.2385068 | up |
| RHOBTB2 | 4.81E-07 | 2.2395477 | up |
| MRPL50 | 6.93E-07 | 2.2395585 | up |
| PRELID1 | 5.78E-07 | 2.240441 | up |
| lnc-RP11-195B21,3,1-2 | 1.31E-06 | 2.241666 | up |
| WLS | 6.60E-07 | 2.2419894 | up |
| CYTH1 | 5.18E-07 | 2.2428076 | up |
| EZR | 3.49E-07 | 2.2433388 | up |
| RFPL1 | 2.75E-07 | 2.2448177 | up |
| AAR2 | 1.24E-06 | 2.2460296 | up |
| ZNF283 | 8.87E-07 | 2.246534 | up |
| BCAR1 | 3.21E-07 | 2.2499366 | up |
| UQCR10 | 4.38E-07 | 2.2502837 | up |
| RAD51 | 8.99E-07 | 2.2517672 | up |
| HSPBP1 | 6.93E-07 | 2.251795 | up |
| PLEKHO2 | 7.74E-07 | 2.2576869 | up |
| PSMC4 | 8.18E-07 | 2.2582676 | up |
| WWTR1 | 5.83E-07 | 2.2595193 | up |
| MCTS1 | 6.31E-07 | 2.2603273 | up |
| HMGN2 | 8.74E-07 | 2.2618883 | up |
| ICMT | 5.91E-07 | 2.262145 | up |
| METTL3 | 1.13E-06 | 2.2627122 | up |
| LOC100289120 | 8.80E-07 | 2.2632864 | up |
| CIB1 | 5.41E-07 | 2.2645497 | up |
| LPAR2 | 1.36E-06 | 2.2649171 | up |
| TAF6L | 2.87E-07 | 2.2650647 | up |
| RUVBL2 | 5.53E-07 | 2.2677805 | up |
| TMEM57 | 5.06E-07 | 2.2695277 | up |
| ADSL | 7.89E-07 | 2.270956 | up |
| MLPH | 7.46E-07 | 2.27152 | up |
| LFNG | 9.16E-07 | 2.2763922 | up |
| TMEM91 | 3.92E-07 | 2.2792213 | up |
| MTRF1L | 4.10E-07 | 2.2807653 | up |
| PDIA5 | 1.15E-06 | 2.2826722 | up |
| NARF | 5.49E-07 | 2.2863646 | up |
| RASA3 | 3.82E-07 | 2.288002 | up |
| SDCCAG3 | 3.07E-07 | 2.2907062 | up |
| GPAA1 | 7.17E-07 | 2.2938895 | up |
| MRPL37 | 1.43E-06 | 2.2946224 | up |
| FLJ36000 | 1.12E-06 | 2.296289 | up |
| MRPL13 | 4.56E-07 | 2.2967045 | up |
| TBC1D9B | 2.86E-07 | 2.2969575 | up |
| LOC100128364 | 1.10E-06 | 2.298348 | up |
| ZNF239 | 1.20E-06 | 2.2995174 | up |
| IPO4 | 9.83E-07 | 2.3022826 | up |
| RBM12 | 1.04E-06 | 2.302423 | up |
| SARS2 | 8.05E-07 | 2.304003 | up |
| PDS5A | 1.35E-06 | 2.304864 | up |
| LOC100131150 | 1.17E-06 | 2.3078945 | up |
| HIATL1 | 3.68E-07 | 2.3080273 | up |
| CD83 | 1.11E-06 | 2.3082068 | up |
| PPP1R13L | 2.98E-07 | 2.3104463 | up |
| TAF13 | 9.11E-07 | 2.3119452 | up |
| DHDDS | 6.37E-07 | 2.314842 | up |
| POMGNT1 | 1.01E-06 | 2.317025 | up |
| SEPT11 | 2.13E-07 | 2.3173952 | up |
| TXNDC5 | 1.04E-06 | 2.317578 | up |
| ZFYVE1 | 8.52E-07 | 2.3182645 | up |
| UBE2L3 | 6.15E-07 | 2.318767 | up |
| PSMD8 | 2.17E-07 | 2.3218482 | up |
| CECR5 | 7.25E-07 | 2.324413 | up |
| ELF4 | 1.15E-06 | 2.3259342 | up |
| TMSB10 | 4.16E-07 | 2.3260932 | up |
| P3H2 | 5.72E-07 | 2.3285193 | up |
| MAP4 | 1.34E-06 | 2.328881 | up |
| CYC1 | 9.62E-07 | 2.3289888 | up |
| ATP7B | 5.60E-07 | 2.3318114 | up |
| ZNF580 | 1.42E-06 | 2.331911 | up |
| ATP6 | 1.26E-06 | 2.3324368 | up |
| NDUFB4 | 9.24E-07 | 2.3336608 | up |
| POLR1C | 1.23E-06 | 2.3381295 | up |
| NOL9 | 7.05E-07 | 2.3390996 | up |
| PPP2R3B | 3.60E-07 | 2.3405826 | up |
| GGH | 3.23E-07 | 2.341986 | up |
| NTAN1 | 1.04E-06 | 2.3421824 | up |
| MSRB1 | 2.84E-07 | 2.3422108 | up |
| TMEM154 | 7.06E-07 | 2.342406 | up |
| INHBC | 1.08E-06 | 2.3450255 | up |
| AAK1 | 6.08E-07 | 2.3465097 | up |
| CYTB | 1.23E-06 | 2.346729 | up |
| VASH1 | 8.99E-07 | 2.3476274 | up |
| ZHX1-C8orf76 | 5.19E-07 | 2.348473 | up |
| C8orf82 | 7.62E-07 | 2.3490384 | up |
| LRRC40 | 7.66E-07 | 2.3496969 | up |
| CRLS1 | 3.19E-07 | 2.351012 | up |
| TIMM50 | 6.28E-07 | 2.3532975 | up |
| CST3 | 5.77E-07 | 2.3539095 | up |
| GMPS | 4.56E-07 | 2.3567653 | up |
| PAFAH1B1 | 1.10E-06 | 2.3570204 | up |
| TMEM134 | 6.29E-07 | 2.3571274 | up |
| QSOX1 | 1.34E-06 | 2.3592334 | up |
| TP53I3 | 4.70E-07 | 2.3599093 | up |
| NDUFAF5 | 5.30E-07 | 2.361452 | up |
| ATP6V1D | 2.06E-07 | 2.3621302 | up |
| ETNK1 | 3.68E-07 | 2.363777 | up |
| KLF16 | 6.65E-07 | 2.3672237 | up |
| SCAND1 | 3.46E-07 | 2.368024 | up |
| ATP1B3 | 5.02E-07 | 2.3681295 | up |
| TMCC2 | 7.78E-07 | 2.368212 | up |
| EBPL | 4.31E-07 | 2.370049 | up |
| SLC45A4 | 7.93E-07 | 2.3702922 | up |
| ZDHHC12 | 1.02E-06 | 2.3708792 | up |
| DDX39A | 5.54E-07 | 2.3710635 | up |
| ANXA2P1 | 4.70E-07 | 2.3729093 | up |
| ZBTB7B | 1.20E-06 | 2.3732247 | up |
| TTTY16 | 4.15E-07 | 2.3743176 | up |
| C4orf46 | 5.69E-07 | 2.375443 | up |
| CCNL2 | 5.37E-07 | 2.3757403 | up |
| B4GALT5 | 2.58E-07 | 2.378096 | up |
| VKORC1 | 4.27E-07 | 2.3789244 | up |
| HLA-A | 7.56E-07 | 2.3793168 | up |
| MIF | 6.85E-07 | 2.3795464 | up |
| SDCCAG3 | 6.06E-07 | 2.3797758 | up |
| GPR124 | 5.08E-07 | 2.3799832 | up |
| MRPL47 | 9.01E-07 | 2.3802195 | up |
| PRR5L | 4.10E-07 | 2.3820848 | up |
| UBE3B | 7.00E-07 | 2.3834217 | up |
| MYL6 | 5.38E-07 | 2.3844392 | up |
| AKR7L | 2.57E-07 | 2.3853831 | up |
| CGN | 6.03E-07 | 2.3856013 | up |
| TBC1D7 | 5.34E-07 | 2.3861988 | up |
| ZNF493 | 1.06E-06 | 2.3871887 | up |
| PSMD12 | 5.31E-07 | 2.3908195 | up |
| INPP5D | 6.65E-07 | 2.3925984 | up |
| CCDC109B | 6.37E-07 | 2.3935175 | up |
| NRARP | 5.49E-07 | 2.3957305 | up |
| UHRF1BP1L | 1.05E-06 | 2.3957345 | up |
| TMEM214 | 4.57E-07 | 2.3959422 | up |
| LOC100507420 | 5.04E-07 | 2.3974078 | up |
| COX8A | 7.58E-07 | 2.397639 | up |
| IMPAD1 | 1.26E-06 | 2.398316 | up |
| KLF7 | 5.39E-07 | 2.400741 | up |
| UBE2MP1 | 2.32E-07 | 2.4029171 | up |
| TP53RK | 1.41E-06 | 2.4045594 | up |
| CTPS2 | 4.97E-07 | 2.4050183 | up |
| TOMM5 | 4.82E-07 | 2.4054434 | up |
| SMYD3 | 5.50E-07 | 2.4080338 | up |
| MAVS | 2.76E-07 | 2.40861 | up |
| RPS23 | 4.31E-07 | 2.409151 | up |
| FKBP2 | 1.21E-06 | 2.4096735 | up |
| TMEM109 | 4.93E-07 | 2.4102905 | up |
| NUCB1 | 9.13E-07 | 2.4161072 | up |
| UBAP2 | 4.30E-07 | 2.4162643 | up |
| ATP9A | 5.68E-07 | 2.4162986 | up |
| ZNF124 | 8.73E-07 | 2.419378 | up |
| RIPK2 | 1.02E-06 | 2.4220622 | up |
| TP53I3 | 6.42E-07 | 2.422786 | up |
| MRTO4 | 1.15E-06 | 2.425763 | up |
| TNFAIP8 | 6.81E-07 | 2.4271498 | up |
| GMFB | 1.24E-06 | 2.4279556 | up |
| LOC100129846 | 6.77E-07 | 2.432709 | up |
| COL4A2 | 4.35E-07 | 2.4347057 | up |
| STX8 | 2.20E-07 | 2.4359102 | up |
| TPM3 | 6.18E-07 | 2.4364643 | up |
| MIF-AS1 | 7.43E-07 | 2.4365587 | up |
| OSCP1 | 5.09E-07 | 2.4373717 | up |
| SH3BGRL2 | 7.66E-07 | 2.4393127 | up |
| GFOD2 | 4.43E-07 | 2.4398565 | up |
| SLC25A51 | 9.22E-07 | 2.4398968 | up |
| ETFA | 5.94E-07 | 2.4409373 | up |
| RAB8A | 3.79E-07 | 2.44315 | up |
| CSTF2 | 7.97E-07 | 2.4447305 | up |
| SAAL1 | 3.11E-07 | 2.4450958 | up |
| MXD3 | 2.82E-07 | 2.4467156 | up |
| ATP1A1 | 4.58E-07 | 2.4513037 | up |
| CUTA | 5.63E-07 | 2.4514613 | up |
| PHRF1 | 6.96E-07 | 2.4531083 | up |
| TCP11L1 | 1.96E-07 | 2.4540582 | up |
| LMNB2 | 8.55E-07 | 2.4562466 | up |
| PER1 | 7.80E-07 | 2.4572701 | up |
| PFDN1 | 2.30E-07 | 2.4574835 | up |
| CHTF18 | 3.04E-07 | 2.4581914 | up |
| AP2M1 | 3.13E-07 | 2.4585903 | up |
| ANO10 | 1.06E-06 | 2.4624665 | up |
| EYA3 | 3.21E-07 | 2.4625359 | up |
| ARHGAP22 | 1.00E-06 | 2.4633505 | up |
| PPP2R1A | 1.43E-06 | 2.4640846 | up |
| TPRX1 | 1.33E-06 | 2.4642272 | up |
| PANK3 | 1.05E-06 | 2.4649827 | up |
| CLPTM1L | 2.80E-07 | 2.4655223 | up |
| SEPHS1 | 1.92E-07 | 2.4666727 | up |
| NUDC | 7.66E-07 | 2.4669871 | up |
| ZCCHC17 | 1.07E-06 | 2.4686794 | up |
| SEPT2 | 2.46E-07 | 2.4689717 | up |
| KIAA1161 | 3.11E-07 | 2.4701486 | up |
| UQCR10 | 3.13E-07 | 2.471086 | up |
| ITGAE | 4.49E-07 | 2.472439 | up |
| GTF2F2 | 1.14E-06 | 2.4725456 | up |
| AGAP3 | 2.67E-07 | 2.4728165 | up |
| FOSL1 | 3.08E-07 | 2.475023 | up |
| RTEL1 | 3.59E-07 | 2.4750826 | up |
| MYL12A | 3.19E-07 | 2.476744 | up |
| SLC41A3 | 3.26E-07 | 2.478424 | up |
| CPPED1 | 2.37E-07 | 2.478677 | up |
| COL6A1 | 5.33E-07 | 2.479663 | up |
| C19orf60 | 2.38E-07 | 2.4803853 | up |
| LOC220729 | 6.23E-07 | 2.4804697 | up |
| PIH1D1 | 1.11E-06 | 2.4804971 | up |
| SURF6 | 1.16E-06 | 2.4809113 | up |
| ZNF845 | 2.69E-07 | 2.482895 | up |
| ZNF248 | 1.17E-06 | 2.4847052 | up |
| SF3A2 | 4.52E-07 | 2.487195 | up |
| TCEB1 | 6.40E-07 | 2.4873083 | up |
| CFLAR | 1.23E-06 | 2.4889262 | up |
| MRPL37 | 1.02E-06 | 2.4889734 | up |
| THRAP3 | 1.08E-06 | 2.489042 | up |
| DOPEY2 | 7.62E-07 | 2.4902108 | up |
| SSR1 | 3.46E-07 | 2.4918697 | up |
| FYTTD1 | 8.29E-07 | 2.4920602 | up |
| PSMA3 | 7.18E-07 | 2.4948478 | up |
| C19orf60 | 4.05E-07 | 2.494881 | up |
| BICD2 | 5.69E-07 | 2.4962404 | up |
| BID | 1.15E-06 | 2.4970627 | up |
| TPGS2 | 2.05E-07 | 2.498492 | up |
| ENTPD1 | 3.89E-07 | 2.5033116 | up |
| PNP | 3.36E-07 | 2.5046873 | up |
| SLAMF9 | 1.15E-06 | 2.505339 | up |
| CDKN2D | 1.69E-07 | 2.5060477 | up |
| PGAM4 | 1.36E-06 | 2.507179 | up |
| ZBED4 | 7.93E-07 | 2.5076864 | up |
| SNHG5 | 9.63E-07 | 2.5082157 | up |
| TMEM141 | 1.44E-06 | 2.5112228 | up |
| DEF6 | 1.43E-06 | 2.5134234 | up |
| WDR90 | 6.46E-07 | 2.5134733 | up |
| ASL | 4.25E-07 | 2.5148573 | up |
| TNFRSF14 | 5.40E-07 | 2.515016 | up |
| C1orf106 | 3.57E-07 | 2.5155306 | up |
| FRMD8 | 6.06E-07 | 2.5159373 | up |
| TCEB3 | 5.98E-07 | 2.5177608 | up |
| MCCC2 | 9.22E-07 | 2.5179553 | up |
| SZRD1 | 1.23E-07 | 2.5190012 | up |
| KIR2DL4 | 1.25E-06 | 2.5233462 | up |
| PIF1 | 5.17E-07 | 2.5272548 | up |
| SLC26A6 | 5.82E-07 | 2.5274286 | up |
| MAPRE1 | 6.27E-07 | 2.5278401 | up |
| PXN | 1.70E-07 | 2.5294816 | up |
| UNC45A | 3.06E-07 | 2.5297704 | up |
| LIG1 | 6.67E-07 | 2.531707 | up |
| KLHL18 | 2.46E-07 | 2.5328698 | up |
| PRAF2 | 9.14E-07 | 2.5348513 | up |
| TMEM191B | 2.89E-07 | 2.5366795 | up |
| THOC3 | 2.30E-07 | 2.5385242 | up |
| PRMT1 | 4.34E-07 | 2.539658 | up |
| EXTL3 | 5.82E-07 | 2.5397596 | up |
| BASP1 | 3.70E-07 | 2.5404098 | up |
| ERAP2 | 3.29E-07 | 2.5415146 | up |
| NDC80 | 6.75E-07 | 2.5421019 | up |
| LOC158435 | 1.17E-06 | 2.5424383 | up |
| PGAM2 | 3.77E-07 | 2.5485148 | up |
| TUBA1C | 2.80E-07 | 2.548756 | up |
| SLIRP | 2.01E-07 | 2.5490203 | up |
| MOSPD1 | 1.72E-07 | 2.5506318 | up |
| DNM2 | 1.99E-07 | 2.551382 | up |
| ATP5D | 4.15E-07 | 2.551814 | up |
| TROAP | 7.05E-07 | 2.5543172 | up |
| PTS | 8.84E-07 | 2.55492 | up |
| TIPIN | 2.55E-07 | 2.555 | up |
| ACAA2 | 5.55E-07 | 2.5566304 | up |
| NSG1 | 1.42E-06 | 2.5579915 | up |
| COLGALT1 | 2.01E-07 | 2.5591965 | up |
| TIMP1 | 3.22E-07 | 2.560443 | up |
| CRELD2 | 1.45E-06 | 2.562602 | up |
| ENDOD1 | 7.14E-07 | 2.5627441 | up |
| SLC20A2 | 7.55E-07 | 2.5689003 | up |
| MAP2K1 | 4.60E-07 | 2.5697887 | up |
| FIBP | 1.17E-06 | 2.5724206 | up |
| MRGBP | 1.38E-07 | 2.572989 | up |
| PSRC1 | 3.25E-07 | 2.574743 | up |
| PC | 4.67E-07 | 2.575271 | up |
| GPSM1 | 3.59E-07 | 2.5753112 | up |
| CDCA3 | 1.31E-07 | 2.5767012 | up |
| FXYD5 | 2.19E-07 | 2.578275 | up |
| POLE2 | 7.42E-07 | 2.5799053 | up |
| PRC1 | 2.42E-07 | 2.580745 | up |
| GINS1 | 4.45E-07 | 2.5821238 | up |
| AP1B1 | 6.17E-07 | 2.584387 | up |
| LRRC59 | 2.99E-07 | 2.5844889 | up |
| TRAPPC4 | 7.52E-07 | 2.5874395 | up |
| MXRA5 | 8.88E-07 | 2.5877953 | up |
| SERF1B | 5.10E-07 | 2.5885916 | up |
| TRPC4 | 1.80E-07 | 2.5902414 | up |
| RBM18 | 5.80E-07 | 2.5914266 | up |
| SLC25A23 | 1.21E-06 | 2.5914352 | up |
| C1orf86 | 1.32E-06 | 2.5936735 | up |
| PQBP1 | 6.24E-07 | 2.595086 | up |
| STK17B | 5.09E-07 | 2.5961874 | up |
| CHAF1A | 2.36E-07 | 2.5962162 | up |
| ERC2-IT1 | 9.27E-07 | 2.5963447 | up |
| TPM3 | 9.90E-07 | 2.596439 | up |
| CHKB-AS1 | 4.14E-07 | 2.5971775 | up |
| PSME4 | 3.27E-07 | 2.597997 | up |
| FOXP1 | 1.09E-06 | 2.5980067 | up |
| SRPK3 | 7.10E-07 | 2.5988798 | up |
| EIF4E | 7.33E-07 | 2.6018927 | up |
| ZNF436-AS1 | 2.64E-07 | 2.6024253 | up |
| ZNFX1 | 1.15E-06 | 2.6027749 | up |
| NF2 | 3.49E-07 | 2.603696 | up |
| FRMD8 | 3.24E-07 | 2.6037629 | up |
| SCYL1 | 1.39E-06 | 2.604137 | up |
| CASP3 | 2.50E-07 | 2.605141 | up |
| TMA7 | 1.80E-07 | 2.6078079 | up |
| CD151 | 5.44E-07 | 2.6083763 | up |
| TBCB | 2.08E-07 | 2.610506 | up |
| VRK1 | 8.10E-07 | 2.6121256 | up |
| SZT2 | 6.50E-07 | 2.6143718 | up |
| ETV2 | 8.51E-07 | 2.6151147 | up |
| NKX3-1 | 1.05E-06 | 2.6164067 | up |
| CCDC23 | 4.37E-07 | 2.6171846 | up |
| IL16 | 6.14E-07 | 2.6175532 | up |
| ARFIP2 | 2.25E-07 | 2.6182241 | up |
| EIF4EBP2 | 1.79E-07 | 2.618804 | up |
| CSNK1D | 1.83E-07 | 2.620531 | up |
| IDH2 | 8.35E-07 | 2.6209474 | up |
| FUT8 | 1.29E-06 | 2.6211698 | up |
| LBHD1 | 9.38E-07 | 2.621377 | up |
| IQGAP3 | 2.53E-07 | 2.6224685 | up |
| TMEM52 | 7.09E-07 | 2.6229088 | up |
| HMGA2 | 5.06E-07 | 2.6246393 | up |
| PIAS2 | 1.44E-07 | 2.627526 | up |
| TDP1 | 1.29E-07 | 2.6301806 | up |
| MYD88 | 4.79E-07 | 2.6302555 | up |
| CACNA1I | 7.88E-07 | 2.630487 | up |
| PPP3CC | 2.41E-07 | 2.6309254 | up |
| NOL7 | 2.30E-07 | 2.634644 | up |
| ATL3 | 3.79E-07 | 2.635258 | up |
| TRIM28 | 9.20E-07 | 2.6354322 | up |
| EEPD1 | 2.41E-07 | 2.6361582 | up |
| SIGMAR1 | 4.34E-07 | 2.6389697 | up |
| CST5 | 1.05E-06 | 2.6392472 | up |
| COL16A1 | 3.81E-07 | 2.6408691 | up |
| NCLN | 2.64E-07 | 2.6426353 | up |
| CSF1 | 1.15E-06 | 2.6454666 | up |
| PCDHB2 | 6.96E-07 | 2.6469042 | up |
| PYCRL | 1.97E-07 | 2.647077 | up |
| CEP170B | 5.47E-07 | 2.6472678 | up |
| YIPF2 | 4.25E-07 | 2.6485279 | up |
| PPP6C | 2.64E-07 | 2.6487079 | up |
| SLC25A4 | 1.36E-06 | 2.6495006 | up |
| CYP2A13 | 1.03E-06 | 2.6507027 | up |
| NELFCD | 6.47E-07 | 2.6510344 | up |
| ZDHHC24 | 1.81E-07 | 2.6522357 | up |
| HECTD3 | 1.09E-06 | 2.6525419 | up |
| CERCAM | 2.76E-07 | 2.6545622 | up |
| GPAT2 | 3.14E-07 | 2.6546738 | up |
| BCL2L12 | 6.86E-07 | 2.6551876 | up |
| ADAMTS16 | 1.31E-06 | 2.6559446 | up |
| YIF1A | 7.52E-07 | 2.656505 | up |
| HLA-DQB1 | 1.95E-07 | 2.656572 | up |
| EMX1 | 4.21E-07 | 2.657714 | up |
| HOXA2 | 4.07E-07 | 2.6644583 | up |
| CEP250 | 1.36E-06 | 2.6648118 | up |
| LDHA | 1.40E-07 | 2.6679955 | up |
| ABL1 | 4.31E-07 | 2.668109 | up |
| PPIB | 2.95E-07 | 2.6684527 | up |
| ATP1A4 | 3.92E-07 | 2.669145 | up |
| IPPK | 1.43E-06 | 2.6693327 | up |
| FAM53C | 8.03E-07 | 2.6696613 | up |
| SH2D3A | 4.20E-07 | 2.6727502 | up |
| CREB3 | 2.62E-07 | 2.67294 | up |
| SLC4A7 | 7.60E-07 | 2.6740577 | up |
| SLC41A3 | 2.54E-07 | 2.675824 | up |
| RCC2 | 1.08E-06 | 2.6758888 | up |
| BUB1 | 1.02E-06 | 2.6786425 | up |
| EID3 | 5.47E-07 | 2.679765 | up |
| CCDC124 | 1.96E-07 | 2.6824038 | up |
| KIAA0101 | 9.74E-08 | 2.683779 | up |
| OTUB1 | 1.28E-07 | 2.683877 | up |
| TTLL12 | 5.93E-07 | 2.6843836 | up |
| HAPLN3 | 2.42E-07 | 2.68546 | up |
| TRAPPC5 | 1.40E-07 | 2.6857464 | up |
| C14orf169 | 1.35E-07 | 2.6867518 | up |
| LIN7B | 4.94E-07 | 2.686767 | up |
| NRG1 | 9.52E-07 | 2.6869588 | up |
| ARFGAP1 | 9.44E-08 | 2.6887617 | up |
| RPN1 | 1.23E-06 | 2.6890705 | up |
| SELM | 1.27E-06 | 2.6905863 | up |
| EIF6 | 1.36E-07 | 2.6917207 | up |
| EI24 | 1.58E-07 | 2.692895 | up |
| ADM | 2.90E-07 | 2.6929128 | up |
| KCTD5 | 7.55E-07 | 2.693241 | up |
| lnc-ANP32A-3 | 6.89E-07 | 2.693881 | up |
| CXCL5 | 4.31E-07 | 2.6948328 | up |
| SAMD14 | 7.01E-07 | 2.6955605 | up |
| CDK16 | 5.35E-07 | 2.695713 | up |
| USP46 | 6.67E-07 | 2.703499 | up |
| SLC35G5 | 4.71E-07 | 2.7086382 | up |
| TBC1D7 | 6.57E-07 | 2.7109554 | up |
| PQBP1 | 6.38E-07 | 2.713281 | up |
| ATP6V0B | 2.39E-07 | 2.7147334 | up |
| UBE2J2 | 1.38E-07 | 2.7152429 | up |
| PKN3 | 3.28E-07 | 2.7178948 | up |
| OR10A5 | 1.28E-06 | 2.7179654 | up |
| CDC42 | 1.67E-07 | 2.7190967 | up |
| BAD | 1.40E-07 | 2.7195182 | up |
| TPM3 | 3.77E-07 | 2.720561 | up |
| GEMIN7 | 8.15E-07 | 2.7210386 | up |
| PQLC1 | 1.24E-06 | 2.7212443 | up |
| ATAD3B | 2.50E-07 | 2.721262 | up |
| CRYBB2P1 | 9.90E-07 | 2.7215612 | up |
| ZNF71 | 8.14E-07 | 2.7216697 | up |
| GTF3C4 | 2.38E-07 | 2.7258253 | up |
| FAM27C | 1.03E-06 | 2.7265968 | up |
| NACC2 | 1.23E-07 | 2.7272909 | up |
| TPX2 | 3.92E-07 | 2.7286153 | up |
| FLVCR1 | 4.06E-07 | 2.7286503 | up |
| RELT | 4.14E-07 | 2.7286654 | up |
| LDHC | 2.83E-07 | 2.7291777 | up |
| LOC645166 | 3.86E-07 | 2.72964 | up |
| SLC1A7 | 5.90E-07 | 2.7296877 | up |
| SRSF7 | 6.90E-07 | 2.7311845 | up |
| VEGFC | 1.71E-07 | 2.7312112 | up |
| PDDC1 | 1.24E-07 | 2.7319322 | up |
| GINS4 | 7.25E-07 | 2.7329447 | up |
| DCAF13 | 5.81E-07 | 2.7331586 | up |
| KRT19P2 | 8.58E-08 | 2.7360075 | up |
| MYCBP | 1.41E-06 | 2.7369323 | up |
| C20orf27 | 1.30E-06 | 2.738817 | up |
| SALL1 | 1.13E-06 | 2.7415524 | up |
| LY9 | 3.61E-07 | 2.7417455 | up |
| JUP | 3.13E-07 | 2.7451167 | up |
| MTA2 | 4.44E-07 | 2.745991 | up |
| TUSC5 | 8.46E-07 | 2.7463613 | up |
| UNC13B | 2.55E-07 | 2.7487655 | up |
| TM6SF1 | 1.43E-07 | 2.7496223 | up |
| PINK1 | 9.22E-08 | 2.7523842 | up |
| SNAI3-AS1 | 8.12E-07 | 2.753163 | up |
| NDUFA6 | 3.21E-07 | 2.753282 | up |
| WDR5 | 1.04E-07 | 2.7546837 | up |
| EMP3 | 2.93E-07 | 2.7549105 | up |
| CTSZ | 2.92E-07 | 2.7558897 | up |
| MAPK13 | 1.07E-06 | 2.756279 | up |
| MAP1LC3B | 3.54E-07 | 2.7575157 | up |
| TFRC | 4.21E-07 | 2.7590516 | up |
| C9orf89 | 1.84E-07 | 2.7592807 | up |
| VMA21 | 3.58E-07 | 2.7596042 | up |
| STARD10 | 2.69E-07 | 2.760053 | up |
| MTMR2 | 2.89E-07 | 2.7608654 | up |
| TNIK | 5.69E-07 | 2.7614582 | up |
| PPP1R18 | 8.49E-07 | 2.7622237 | up |
| TUBA1C | 8.24E-07 | 2.762533 | up |
| WASF2 | 1.13E-06 | 2.7634387 | up |
| PMPCA | 1.52E-07 | 2.7669473 | up |
| TFDP2 | 1.36E-06 | 2.771726 | up |
| MZT1 | 1.21E-06 | 2.773617 | up |
| KHDC1 | 1.11E-06 | 2.7744465 | up |
| PHF13 | 1.19E-07 | 2.7745624 | up |
| YIF1A | 4.53E-07 | 2.776447 | up |
| PDLIM5 | 1.15E-06 | 2.77833 | up |
| KLC2 | 1.06E-06 | 2.7810075 | up |
| FPGS | 3.36E-07 | 2.7814746 | up |
| CTNNB1 | 1.38E-06 | 2.7830298 | up |
| FMNL3 | 8.68E-08 | 2.7854843 | up |
| CCDC23 | 5.08E-07 | 2.785997 | up |
| PQBP1 | 1.27E-07 | 2.7869577 | up |
| SMYD3 | 1.03E-07 | 2.7878952 | up |
| SNRPA | 1.19E-07 | 2.7891798 | up |
| U2AF2 | 1.07E-06 | 2.7913182 | up |
| UQCRC1 | 6.02E-07 | 2.7928703 | up |
| UNC13B | 4.85E-07 | 2.793236 | up |
| FCER1G | 1.39E-06 | 2.793932 | up |
| TALDO1 | 6.38E-07 | 2.7941403 | up |
| TECR | 3.20E-07 | 2.7942252 | up |
| GTF2A2 | 1.53E-07 | 2.7942774 | up |
| ZBTB45 | 4.42E-07 | 2.79503 | up |
| RNF26 | 1.42E-07 | 2.795944 | up |
| SRSF3 | 1.02E-06 | 2.7966723 | up |
| FEN1 | 1.21E-07 | 2.7968018 | up |
| PACS1 | 3.08E-07 | 2.7986627 | up |
| TMEM191A | 3.23E-07 | 2.7996378 | up |
| TPM1 | 1.86E-07 | 2.7996948 | up |
| PTER | 2.27E-07 | 2.8034422 | up |
| LOC401557 | 1.09E-06 | 2.8093889 | up |
| PLSCR3 | 1.12E-06 | 2.8094 | up |
| EVI5L | 6.65E-07 | 2.8104186 | up |
| BOLA2B | 2.39E-07 | 2.8131177 | up |
| CENPM | 1.55E-07 | 2.8134305 | up |
| AKT1 | 1.42E-06 | 2.8134718 | up |
| EHD4 | 2.04E-07 | 2.815288 | up |
| CDKL1 | 1.02E-06 | 2.8156133 | up |
| WDR62 | 5.32E-07 | 2.8158865 | up |
| NCKAP5 | 1.18E-06 | 2.816784 | up |
| HNRNPUL2 | 8.07E-07 | 2.816788 | up |
| CCBL2 | 1.06E-06 | 2.817842 | up |
| RANGAP1 | 2.99E-07 | 2.8180146 | up |
| PSMA7 | 3.07E-07 | 2.8190136 | up |
| GRWD1 | 2.33E-07 | 2.819102 | up |
| TXNL4A | 3.60E-07 | 2.821652 | up |
| RRM2 | 1.00E-06 | 2.8220742 | up |
| SNRPA1 | 1.01E-06 | 2.8227775 | up |
| CTXN1 | 5.55E-07 | 2.8241153 | up |
| ISG15 | 1.70E-07 | 2.8250394 | up |
| ENDOG | 1.96E-07 | 2.8254013 | up |
| RNF126 | 4.90E-07 | 2.8259323 | up |
| PCMT1 | 4.57E-07 | 2.830001 | up |
| MAP7D1 | 7.47E-07 | 2.8338315 | up |
| DCUN1D5 | 5.33E-07 | 2.8352299 | up |
| FKBP1A | 8.84E-07 | 2.836752 | up |
| LOC100131929 | 3.99E-07 | 2.8380904 | up |
| FCRLB | 8.04E-07 | 2.8419507 | up |
| TMEM160 | 1.81E-07 | 2.8427477 | up |
| MTMR3 | 4.64E-07 | 2.8445587 | up |
| MRPL36 | 1.13E-07 | 2.844835 | up |
| POLG | 1.85E-07 | 2.846875 | up |
| PRPS1 | 4.95E-07 | 2.8484416 | up |
| FZD7 | 1.42E-06 | 2.849549 | up |
| ENY2 | 1.37E-07 | 2.849578 | up |
| DCAF13 | 3.23E-07 | 2.8508449 | up |
| HIC2 | 1.24E-06 | 2.8511178 | up |
| MMP24-AS1 | 6.75E-07 | 2.8515396 | up |
| EIF4E2 | 1.03E-07 | 2.85256 | up |
| SH3GL1P1 | 5.99E-07 | 2.8544111 | up |
| PRELID1 | 2.67E-07 | 2.8574128 | up |
| ANO10 | 2.32E-07 | 2.8582754 | up |
| ACTR3 | 2.93E-07 | 2.8586464 | up |
| TPM1 | 9.79E-07 | 2.8588734 | up |
| FBXO3 | 8.51E-07 | 2.859554 | up |
| HAUS7 | 4.69E-07 | 2.8606486 | up |
| MEFV | 1.62E-07 | 2.8621724 | up |
| BOLA2B | 5.63E-07 | 2.8639405 | up |
| DIO2 | 5.72E-07 | 2.8639476 | up |
| SLC38A10 | 8.98E-07 | 2.8661785 | up |
| CDC45 | 6.93E-07 | 2.866183 | up |
| CMTM7 | 8.70E-07 | 2.8685346 | up |
| LINC00523 | 2.26E-07 | 2.8690202 | up |
| SLC1A1 | 1.02E-07 | 2.869042 | up |
| ZNF275 | 2.27E-07 | 2.8691382 | up |
| CDCA5 | 9.37E-08 | 2.8710928 | up |
| PPP2R5B | 1.10E-07 | 2.8712645 | up |
| TPM1 | 1.12E-06 | 2.8720036 | up |
| SUB1 | 5.30E-07 | 2.87384 | up |
| MAP2K3 | 9.16E-08 | 2.874216 | up |
| IRS1 | 1.81E-07 | 2.8801348 | up |
| TMEM132A | 8.85E-07 | 2.8813643 | up |
| NUDT16L1 | 2.18E-07 | 2.8817055 | up |
| IRS1 | 8.13E-08 | 2.881739 | up |
| IDO1 | 1.33E-06 | 2.8829396 | up |
| DHCR7 | 7.83E-07 | 2.8832185 | up |
| RECQL4 | 4.44E-07 | 2.8834572 | up |
| BOK | 1.30E-07 | 2.8861506 | up |
| C2orf61 | 6.25E-07 | 2.8868194 | up |
| HLA-DOA | 6.15E-07 | 2.88826 | up |
| KDM2A | 1.42E-06 | 2.890014 | up |
| NEURL1B | 2.00E-07 | 2.8958328 | up |
| UROD | 8.53E-08 | 2.9013975 | up |
| DCAF13P3 | 3.93E-07 | 2.902856 | up |
| TOMM34 | 2.26E-07 | 2.903721 | up |
| UBA1 | 1.21E-06 | 2.9042776 | up |
| SAC3D1 | 2.94E-07 | 2.9050303 | up |
| PKMYT1 | 1.61E-07 | 2.905956 | up |
| FHL2 | 1.40E-07 | 2.9066546 | up |
| LOC653602 | 1.15E-06 | 2.9070475 | up |
| MPP1 | 1.55E-07 | 2.9076104 | up |
| PRPS1 | 1.35E-07 | 2.9095614 | up |
| SAE1 | 1.05E-06 | 2.9119883 | up |
| MYRF | 5.63E-07 | 2.9121172 | up |
| MARK2 | 1.29E-07 | 2.9128926 | up |
| ODF2 | 1.65E-07 | 2.9130156 | up |
| RAD54L | 6.14E-07 | 2.9174838 | up |
| SKA1 | 4.35E-07 | 2.9177296 | up |
| C11orf52 | 1.42E-06 | 2.918595 | up |
| PRKCD | 3.67E-07 | 2.9226844 | up |
| FAM114A1 | 4.68E-07 | 2.9239068 | up |
| SEC13 | 5.16E-07 | 2.9266899 | up |
| DIP2A | 2.03E-07 | 2.9277968 | up |
| PAICS | 3.12E-07 | 2.92883 | up |
| ANKRD30BL | 1.26E-06 | 2.9329035 | up |
| MICAL1 | 1.27E-06 | 2.9390576 | up |
| HIST1H4C | 7.63E-07 | 2.939541 | up |
| GBP1 | 2.63E-07 | 2.9395995 | up |
| KIF23 | 8.46E-08 | 2.9427853 | up |
| CDKN1C | 3.50E-07 | 2.9477897 | up |
| ADCY4 | 8.94E-07 | 2.9516315 | up |
| LOC727721 | 4.76E-07 | 2.95383 | up |
| PRMT6 | 2.45E-07 | 2.9540813 | up |
| SIAH2 | 1.47E-07 | 2.9553149 | up |
| VDAC3 | 1.91E-07 | 2.9569805 | up |
| DCXR | 3.79E-07 | 2.9572349 | up |
| CDC42 | 4.18E-07 | 2.9587917 | up |
| OIP5 | 1.26E-07 | 2.959931 | up |
| GSTP1 | 1.13E-06 | 2.9604492 | up |
| GADD45GIP1 | 1.46E-07 | 2.9619424 | up |
| FBXL18 | 8.22E-07 | 2.962891 | up |
| CCBL2 | 7.90E-07 | 2.9635832 | up |
| HOXA11 | 1.91E-07 | 2.9640448 | up |
| PBK | 1.17E-07 | 2.9642425 | up |
| EMC10 | 2.96E-07 | 2.969257 | up |
| MTMR2 | 8.23E-08 | 2.9731839 | up |
| OLFML2B | 2.22E-07 | 2.973475 | up |
| VAMP3 | 3.17E-07 | 2.9754148 | up |
| SPACA6P | 2.02E-07 | 2.9768908 | up |
| PPARD | 2.53E-07 | 2.980633 | up |
| C19orf70 | 6.41E-07 | 2.981809 | up |
| ARMC4 | 8.72E-07 | 2.9840865 | up |
| CALML3 | 2.18E-07 | 2.986852 | up |
| PPDPF | 9.98E-07 | 2.9872823 | up |
| MPND | 1.22E-06 | 2.9876964 | up |
| TMEM40 | 1.89E-07 | 2.9889696 | up |
| ARHGAP33 | 8.67E-07 | 2.9908946 | up |
| SUV39H1 | 1.86E-07 | 2.9923992 | up |
| GRPEL1 | 2.23E-07 | 2.9954169 | up |
| MICB | 3.22E-07 | 2.9970632 | up |
| TBC1D16 | 1.66E-07 | 2.997235 | up |
| UBTD1 | 5.03E-07 | 2.9973972 | up |
| BAG4 | 1.61E-07 | 3.0013828 | up |
| FBXW9 | 3.50E-07 | 3.0082586 | up |
| MRPL14 | 3.28E-07 | 3.0085888 | up |
| MAPK13 | 1.67E-07 | 3.0090353 | up |
| LOC100130051 | 7.34E-07 | 3.0096273 | up |
| ZNF215 | 1.06E-06 | 3.0099018 | up |
| CSE1L | 1.34E-07 | 3.011595 | up |
| REEP6 | 3.13E-07 | 3.0122256 | up |
| SEC13 | 3.17E-07 | 3.0136194 | up |
| PLGRKT | 2.54E-07 | 3.0195382 | up |
| TUBA1A | 1.29E-07 | 3.0220351 | up |
| FBLL1 | 1.05E-06 | 3.0230365 | up |
| EIF4E2 | 9.62E-08 | 3.0236528 | up |
| HNRNPUL1 | 3.87E-07 | 3.0244565 | up |
| NDUFC2 | 1.18E-06 | 3.0248637 | up |
| PQLC1 | 1.82E-07 | 3.0257003 | up |
| FAM105A | 7.99E-07 | 3.031336 | up |
| TCEB2 | 1.76E-07 | 3.0369492 | up |
| PSMD13 | 1.35E-07 | 3.041452 | up |
| MYO10 | 4.58E-07 | 3.0415351 | up |
| PANK3 | 5.77E-07 | 3.0430837 | up |
| PHC2 | 1.78E-07 | 3.0494668 | up |
| DSTN | 5.06E-08 | 3.0499392 | up |
| PLEKHG2 | 2.45E-07 | 3.0504005 | up |
| PNKP | 9.06E-08 | 3.054664 | up |
| MAP2K3 | 3.12E-07 | 3.0554125 | up |
| NLN | 1.38E-07 | 3.0598395 | up |
| SURF4 | 1.19E-06 | 3.0607338 | up |
| CKS2 | 1.00E-06 | 3.0694394 | up |
| VBP1 | 1.32E-06 | 3.072515 | up |
| PHF20L1 | 1.32E-06 | 3.0732868 | up |
| GMIP | 5.11E-07 | 3.0733058 | up |
| LOC146795 | 3.53E-07 | 3.0748606 | up |
| GDF11 | 1.54E-07 | 3.0755484 | up |
| LDOC1 | 3.49E-07 | 3.0758095 | up |
| CDH4 | 8.09E-07 | 3.0761573 | up |
| SH2D2A | 1.16E-07 | 3.0797303 | up |
| NANOS3 | 1.63E-07 | 3.0797353 | up |
| RPS27L | 5.42E-08 | 3.0820675 | up |
| UBE2J1 | 5.41E-07 | 3.0823169 | up |
| FCHO1 | 3.56E-07 | 3.0847392 | up |
| CCDC134 | 9.77E-07 | 3.084804 | up |
| MED27 | 1.30E-06 | 3.0852468 | up |
| PAFAH1B2 | 1.07E-07 | 3.0867832 | up |
| BORA | 3.79E-07 | 3.0887322 | up |
| DOLPP1 | 1.12E-06 | 3.0894153 | up |
| RBM14 | 3.53E-07 | 3.0906343 | up |
| SIX4 | 9.33E-08 | 3.0909772 | up |
| LOC101928076 | 1.13E-07 | 3.0918624 | up |
| EHD1 | 1.06E-07 | 3.0925405 | up |
| RHOC | 4.87E-07 | 3.0945 | up |
| VAMP3 | 8.46E-07 | 3.0962136 | up |
| ZNF488 | 1.05E-06 | 3.096302 | up |
| C2orf91 | 3.99E-07 | 3.096789 | up |
| NDST1 | 1.77E-07 | 3.0973995 | up |
| ENSA | 4.85E-07 | 3.0975213 | up |
| MED26 | 1.98E-07 | 3.0978665 | up |
| PIN1 | 1.22E-07 | 3.0979242 | up |
| BUB1B | 5.78E-07 | 3.0985053 | up |
| C19orf12 | 6.26E-08 | 3.0988247 | up |
| IL17RA | 5.79E-08 | 3.10021 | up |
| PTPRK | 3.69E-07 | 3.1017444 | up |
| AP1M1 | 3.14E-07 | 3.102199 | up |
| SVIL-AS1 | 2.00E-07 | 3.1024075 | up |
| GFER | 2.83E-07 | 3.104082 | up |
| GNB4 | 4.25E-07 | 3.1049097 | up |
| RNF126 | 5.84E-08 | 3.1054401 | up |
| DNMT3A | 1.14E-06 | 3.1062944 | up |
| STK10 | 1.20E-07 | 3.1071944 | up |
| PGD | 1.02E-06 | 3.1083453 | up |
| MPV17L2 | 1.50E-07 | 3.1087213 | up |
| DCBLD2 | 8.31E-08 | 3.1112466 | up |
| BLM | 2.75E-07 | 3.114929 | up |
| SZRD1 | 1.37E-06 | 3.1174676 | up |
| SSC4D | 2.51E-07 | 3.1209803 | up |
| CCNJL | 2.93E-07 | 3.1259158 | up |
| ATOX1 | 1.88E-07 | 3.126334 | up |
| HIP1R | 3.80E-07 | 3.1297462 | up |
| SKI | 1.08E-06 | 3.1303074 | up |
| CSE1L | 1.17E-07 | 3.1318262 | up |
| TEAD3 | 1.96E-07 | 3.132882 | up |
| C10orf82 | 2.09E-07 | 3.1338758 | up |
| NAV1 | 6.56E-07 | 3.1351936 | up |
| SNN | 5.29E-07 | 3.1361153 | up |
| TNFAIP3 | 1.55E-07 | 3.1389966 | up |
| B3GAT3 | 2.33E-07 | 3.1390762 | up |
| RAB1B | 3.98E-08 | 3.139197 | up |
| MAGEB2 | 5.91E-07 | 3.1425145 | up |
| CECR2 | 1.12E-06 | 3.1436045 | up |
| QPRT | 1.99E-07 | 3.1452312 | up |
| SRSF2 | 8.27E-08 | 3.145568 | up |
| SIDT2 | 4.78E-07 | 3.147757 | up |
| HARS | 2.89E-07 | 3.1540132 | up |
| NACAD | 3.03E-07 | 3.1554646 | up |
| EP400NL | 1.43E-06 | 3.1564713 | up |
| MRPL54 | 1.54E-07 | 3.1566532 | up |
| LYPD1 | 1.09E-06 | 3.1595042 | up |
| RNASEH2A | 8.88E-08 | 3.1608062 | up |
| DLGAP5 | 3.83E-07 | 3.1608927 | up |
| CLN6 | 7.18E-07 | 3.1612375 | up |
| HK2 | 9.17E-08 | 3.162847 | up |
| LOC645195 | 2.21E-07 | 3.163082 | up |
| FASN | 1.07E-07 | 3.1666546 | up |
| ZP1 | 1.37E-06 | 3.166931 | up |
| CBFA2T2 | 6.69E-07 | 3.1712742 | up |
| TCIRG1 | 5.37E-08 | 3.172841 | up |
| UBE2A | 1.21E-07 | 3.1758754 | up |
| PANX1 | 8.14E-07 | 3.1759713 | up |
| TAF1B | 1.19E-06 | 3.1776834 | up |
| MARVELD2 | 1.06E-06 | 3.1778574 | up |
| ARMC6 | 7.51E-08 | 3.1834342 | up |
| HCCS | 1.85E-07 | 3.1896539 | up |
| TXN | 1.94E-07 | 3.1900225 | up |
| LYPLA2 | 3.39E-07 | 3.1901605 | up |
| NPC1L1 | 6.63E-07 | 3.1947916 | up |
| PLAUR | 1.64E-07 | 3.1971219 | up |
| LOC100996924 | 9.86E-07 | 3.198967 | up |
| RABGAP1L | 2.36E-07 | 3.2017486 | up |
| SP100 | 6.94E-08 | 3.2029383 | up |
| DKFZP564C152 | 5.11E-07 | 3.204436 | up |
| SIK1 | 8.00E-07 | 3.2061856 | up |
| PLIN3 | 5.68E-07 | 3.2090373 | up |
| YWHAE | 3.36E-07 | 3.2117224 | up |
| RRAS | 3.89E-08 | 3.2123766 | up |
| POLE | 1.30E-06 | 3.2177439 | up |
| GTPBP6 | 1.21E-07 | 3.2219377 | up |
| VASH2 | 2.60E-07 | 3.2221615 | up |
| CDC25C | 4.10E-07 | 3.2293186 | up |
| DHRS2 | 1.97E-07 | 3.2294514 | up |
| PAQR3 | 5.31E-08 | 3.2309828 | up |
| ACVR2B-AS1 | 3.38E-07 | 3.2410562 | up |
| FAM195B | 1.89E-07 | 3.241293 | up |
| KALRN | 7.90E-07 | 3.2424393 | up |
| JAM2 | 8.37E-07 | 3.242755 | up |
| PGAM1 | 7.07E-07 | 3.2457705 | up |
| ZNF365 | 3.08E-07 | 3.246882 | up |
| POMGNT2 | 2.74E-07 | 3.2481973 | up |
| BIRC5 | 8.44E-08 | 3.249363 | up |
| GCDH | 1.92E-07 | 3.2582731 | up |
| ASF1B | 1.57E-07 | 3.2591207 | up |
| SH3GL1 | 1.89E-07 | 3.260502 | up |
| EPS15L1 | 1.06E-07 | 3.2627225 | up |
| CMTM7 | 1.50E-07 | 3.271495 | up |
| CENPN | 6.48E-07 | 3.2720776 | up |
| CCNB1 | 1.19E-07 | 3.2744584 | up |
| RABGAP1L | 3.27E-07 | 3.2745435 | up |
| HOXA-AS3 | 1.86E-07 | 3.2854464 | up |
| LOC100130673 | 4.83E-07 | 3.2907767 | up |
| CENPO | 5.66E-07 | 3.2914348 | up |
| TAF8 | 3.41E-07 | 3.2930212 | up |
| CAMSAP1 | 3.98E-07 | 3.2951138 | up |
| SOCS2 | 1.02E-07 | 3.2969346 | up |
| NAT6 | 4.81E-07 | 3.301239 | up |
| SCUBE1 | 1.05E-06 | 3.3015761 | up |
| GUCA1C | 1.20E-06 | 3.301795 | up |
| LHB | 2.39E-07 | 3.316051 | up |
| PACSIN3 | 7.94E-07 | 3.316235 | up |
| AURKAPS1 | 7.68E-08 | 3.3173618 | up |
| LDHA | 2.95E-07 | 3.320572 | up |
| C4orf46 | 9.93E-07 | 3.3217025 | up |
| KHSRP | 1.24E-06 | 3.3219116 | up |
| TMEM8B | 2.39E-07 | 3.3231773 | up |
| SNRNP25 | 5.43E-08 | 3.3244085 | up |
| KRT15 | 1.07E-06 | 3.3265827 | up |
| RABGAP1L | 1.15E-06 | 3.328797 | up |
| TBX2 | 1.05E-06 | 3.3333502 | up |
| PRELID2 | 2.52E-07 | 3.3363311 | up |
| TCTA | 2.23E-07 | 3.33644 | up |
| RASSF4 | 2.99E-07 | 3.3388999 | up |
| LINC00889 | 3.78E-07 | 3.3404086 | up |
| PITRM1 | 2.18E-07 | 3.3406663 | up |
| ZSCAN2 | 1.64E-07 | 3.3449063 | up |
| GNAI2 | 2.02E-07 | 3.3453584 | up |
| PHF11 | 1.39E-07 | 3.3457057 | up |
| PAFAH1B3 | 4.04E-08 | 3.348348 | up |
| DYNLT1 | 1.05E-07 | 3.3496556 | up |
| ZNF823 | 3.98E-07 | 3.3500443 | up |
| WEE1 | 1.19E-07 | 3.350634 | up |
| AGPAT3 | 2.29E-07 | 3.3517165 | up |
| KIDINS220 | 1.04E-06 | 3.3523643 | up |
| DKK3 | 3.92E-07 | 3.3524218 | up |
| MICA | 3.71E-08 | 3.3559973 | up |
| SORD | 1.72E-07 | 3.357119 | up |
| PCDHGA8 | 1.16E-07 | 3.359748 | up |
| ATAD3B | 2.33E-07 | 3.3610687 | up |
| ZNF503 | 1.12E-06 | 3.3620844 | up |
| HBQ1 | 1.42E-07 | 3.3635957 | up |
| AP2S1 | 3.91E-08 | 3.3657453 | up |
| RGS3 | 1.23E-07 | 3.366039 | up |
| BZW1 | 1.55E-07 | 3.3670137 | up |
| CLIC5 | 4.34E-08 | 3.3685164 | up |
| ATAD3A | 2.70E-07 | 3.368939 | up |
| KDM4B | 5.12E-08 | 3.3690784 | up |
| FAM171A1 | 4.90E-07 | 3.3712392 | up |
| EHD3 | 8.80E-07 | 3.3735993 | up |
| PDXP | 5.20E-07 | 3.37393 | up |
| AP1B1 | 1.56E-07 | 3.3741286 | up |
| TTK | 1.20E-06 | 3.3771198 | up |
| JAK3 | 1.45E-07 | 3.3822415 | up |
| SPTBN1 | 1.03E-07 | 3.3837893 | up |
| DEFB4A | 1.03E-06 | 3.3838403 | up |
| BCOR | 8.54E-07 | 3.3844535 | up |
| ACOT7 | 5.97E-07 | 3.3881211 | up |
| OR51F1 | 2.07E-07 | 3.3934002 | up |
| PTOV1 | 5.69E-08 | 3.3960803 | up |
| TRAF4 | 1.40E-07 | 3.4001043 | up |
| TP53TG3 | 1.34E-06 | 3.4023616 | up |
| KCTD21 | 3.97E-07 | 3.4058928 | up |
| CBR3 | 2.17E-07 | 3.405962 | up |
| KCNMA1 | 3.50E-08 | 3.4073048 | up |
| PODXL2 | 4.42E-07 | 3.410552 | up |
| CAPZB | 4.52E-07 | 3.411359 | up |
| KBTBD11 | 3.05E-07 | 3.4131155 | up |
| FBLN1 | 2.59E-07 | 3.414735 | up |
| FAM219A | 5.57E-07 | 3.414947 | up |
| GCK | 9.69E-07 | 3.4179688 | up |
| RNASET2 | 1.36E-07 | 3.4191298 | up |
| CHKB | 3.92E-07 | 3.4197845 | up |
| CDH2 | 3.92E-07 | 3.4198434 | up |
| XLOC_l2_013410 | 2.69E-07 | 3.4199944 | up |
| ZNF784 | 1.13E-06 | 3.4216447 | up |
| TMEM189 | 8.44E-07 | 3.4233227 | up |
| LOC102723456 | 1.81E-07 | 3.4334993 | up |
| SRSF2 | 1.46E-07 | 3.4335597 | up |
| RTKN2 | 2.24E-07 | 3.4344504 | up |
| DYNLT3 | 1.77E-07 | 3.4415107 | up |
| ACOX3 | 6.07E-07 | 3.4447715 | up |
| PRPF40A | 2.91E-07 | 3.4463537 | up |
| TUBA8 | 2.24E-07 | 3.4494867 | up |
| CSRP2 | 3.99E-07 | 3.451917 | up |
| SIDT2 | 1.04E-07 | 3.4549215 | up |
| PTPLA | 7.34E-07 | 3.4571357 | up |
| LOC100128714 | 3.67E-07 | 3.4579506 | up |
| PRKAR2A | 1.95E-07 | 3.4596622 | up |
| LOC101927270 | 8.90E-07 | 3.4612417 | up |
| YWHAZ | 1.07E-07 | 3.4662988 | up |
| OPN1MW | 1.49E-07 | 3.4675846 | up |
| TMEM25 | 2.50E-07 | 3.467809 | up |
| PRMT2 | 1.66E-07 | 3.4719813 | up |
| PTPLA | 2.20E-07 | 3.4732249 | up |
| FHL2 | 1.44E-07 | 3.4734676 | up |
| PRADC1 | 5.45E-07 | 3.4742098 | up |
| CENPB | 6.32E-08 | 3.4754229 | up |
| WDR77 | 4.89E-08 | 3.4762828 | up |
| TUBA3FP | 7.26E-07 | 3.4789724 | up |
| H2AFZ | 4.33E-08 | 3.4790022 | up |
| RNASET2 | 3.87E-08 | 3.4811745 | up |
| HTRA1 | 5.60E-08 | 3.4854574 | up |
| LYPD1 | 8.98E-08 | 3.4869063 | up |
| CRYAB | 6.14E-07 | 3.4883466 | up |
| ZNF813 | 5.08E-07 | 3.48841 | up |
| ZNF324 | 3.59E-07 | 3.4942813 | up |
| ANAPC15 | 2.97E-07 | 3.4955785 | up |
| FAM127C | 1.39E-07 | 3.4959126 | up |
| LASP1 | 5.76E-08 | 3.5003755 | up |
| CCNB2 | 3.88E-08 | 3.5011194 | up |
| SIGIRR | 8.50E-08 | 3.5022316 | up |
| XLOC_l2_010433 | 1.24E-07 | 3.5055773 | up |
| RBFA | 1.32E-07 | 3.505758 | up |
| CXorf49B | 8.59E-07 | 3.5109775 | up |
| LYPLA2 | 9.62E-08 | 3.514392 | up |
| MAGEL2 | 6.69E-07 | 3.514569 | up |
| MON1A | 4.27E-08 | 3.5181465 | up |
| GRIN2D | 1.13E-06 | 3.518468 | up |
| ATAD3B | 1.25E-07 | 3.5186543 | up |
| SDC3 | 1.65E-07 | 3.5218363 | up |
| TMEM92 | 8.65E-08 | 3.5235417 | up |
| SNHG1 | 6.78E-07 | 3.5253975 | up |
| FKBP1A | 7.72E-08 | 3.5261743 | up |
| MSANTD3 | 2.60E-07 | 3.5264935 | up |
| FAM83D | 8.79E-07 | 3.5269935 | up |
| TRAPPC10 | 7.14E-07 | 3.5282266 | up |
| MYCL | 5.05E-08 | 3.5296535 | up |
| CLIC4 | 4.69E-08 | 3.535522 | up |
| AKT1S1 | 4.03E-07 | 3.5400567 | up |
| COX2 | 4.05E-07 | 3.5420823 | up |
| HMGCR | 6.77E-08 | 3.5433326 | up |
| SPINT1 | 9.82E-08 | 3.5460992 | up |
| TMEM189 | 9.74E-07 | 3.5503564 | up |
| TSKU | 8.08E-08 | 3.5519924 | up |
| BCAP31 | 6.14E-08 | 3.5532866 | up |
| TMEM201 | 4.22E-08 | 3.5534081 | up |
| SMAP2 | 8.45E-07 | 3.55441 | up |
| ZNF442 | 1.35E-06 | 3.556258 | up |
| ZNF486 | 1.26E-06 | 3.558336 | up |
| POTEKP | 1.40E-07 | 3.559562 | up |
| EFNA5 | 1.78E-07 | 3.5609455 | up |
| SLC25A10 | 5.42E-07 | 3.561167 | up |
| WNT5B | 1.67E-07 | 3.5676954 | up |
| ZNF703 | 4.78E-07 | 3.5700521 | up |
| SLC22A23 | 1.17E-07 | 3.576309 | up |
| SLC25A15 | 2.23E-07 | 3.5824962 | up |
| CLIC1 | 4.04E-08 | 3.5825195 | up |
| PPARD | 8.64E-07 | 3.5831306 | up |
| DLST | 4.22E-07 | 3.5852375 | up |
| WDR18 | 2.18E-08 | 3.5862236 | up |
| CFH | 8.19E-07 | 3.5864165 | up |
| PDIA5 | 1.41E-07 | 3.5869837 | up |
| ZFP64 | 2.04E-07 | 3.5876112 | up |
| POLA2 | 3.07E-08 | 3.58893 | up |
| CDIP1 | 2.47E-07 | 3.5924044 | up |
| CTSB | 3.56E-08 | 3.5982912 | up |
| HIST1H4D | 6.39E-07 | 3.598518 | up |
| EIF2B3 | 1.18E-07 | 3.599849 | up |
| REEP4 | 8.45E-08 | 3.6044834 | up |
| SEC24A | 5.00E-07 | 3.6073027 | up |
| FAM171A1 | 1.32E-06 | 3.6074922 | up |
| HIP1 | 8.31E-08 | 3.6078343 | up |
| LRG1 | 1.30E-07 | 3.6158526 | up |
| FITM2 | 2.72E-07 | 3.6202886 | up |
| SPSB1 | 1.05E-07 | 3.6206152 | up |
| DOCK9 | 3.12E-07 | 3.6215146 | up |
| CDH3 | 5.39E-07 | 3.6218455 | up |
| PVR | 4.11E-07 | 3.631058 | up |
| LOC101927497 | 3.33E-07 | 3.6313372 | up |
| CALM3 | 5.73E-08 | 3.641345 | up |
| MACROD2 | 3.83E-07 | 3.643356 | up |
| SPSB1 | 6.54E-08 | 3.6456227 | up |
| NDUFS5 | 3.18E-08 | 3.6473918 | up |
| ROGDI | 8.16E-08 | 3.6532016 | up |
| NDST2 | 1.35E-06 | 3.6582136 | up |
| CHMP4B | 4.57E-07 | 3.6585896 | up |
| HRAS | 9.74E-07 | 3.659975 | up |
| PCGF3 | 2.33E-08 | 3.6657043 | up |
| MIER2 | 3.72E-08 | 3.6682048 | up |
| KCNK12 | 8.70E-08 | 3.669844 | up |
| EPHX3 | 9.60E-08 | 3.6726284 | up |
| RGAG4 | 5.05E-07 | 3.6748724 | up |
| FAM89B | 2.56E-08 | 3.6756954 | up |
| WNT10A | 3.23E-07 | 3.678074 | up |
| MMP23B | 5.66E-07 | 3.680381 | up |
| CALR | 9.94E-08 | 3.6803885 | up |
| CECR6 | 2.56E-07 | 3.6822972 | up |
| DDA1 | 1.73E-07 | 3.684322 | up |
| CALM3 | 5.29E-08 | 3.6844895 | up |
| LOC101927497 | 2.90E-07 | 3.690881 | up |
| DOK5 | 3.74E-07 | 3.691378 | up |
| H2AFY | 2.30E-08 | 3.6914477 | up |
| KIFC1 | 1.04E-06 | 3.692629 | up |
| ZNF814 | 3.39E-07 | 3.6973662 | up |
| VARS | 3.41E-08 | 3.7025878 | up |
| MICAL1 | 5.10E-08 | 3.708232 | up |
| LOC148709 | 8.23E-08 | 3.7117035 | up |
| LOC401127 | 6.94E-08 | 3.711887 | up |
| LRR1 | 7.25E-07 | 3.7139168 | up |
| MACROD1 | 5.21E-07 | 3.7171938 | up |
| POTEM | 2.56E-07 | 3.7173588 | up |
| TMEM57 | 3.66E-07 | 3.7180557 | up |
| PDLIM4 | 1.41E-07 | 3.7221303 | up |
| FNIP2 | 3.32E-08 | 3.7270012 | up |
| ABLIM3 | 1.12E-06 | 3.7278562 | up |
| HEATR5A | 6.59E-08 | 3.733865 | up |
| STXBP6 | 2.45E-07 | 3.7381139 | up |
| MRPL19 | 1.94E-08 | 3.7392964 | up |
| AGRN | 5.83E-07 | 3.744726 | up |
| EPS15 | 3.55E-07 | 3.7488375 | up |
| GSG2 | 1.19E-07 | 3.7624512 | up |
| ARID5B | 3.01E-08 | 3.7652512 | up |
| ADAMTS14 | 1.02E-07 | 3.7656949 | up |
| HYAL2 | 3.75E-07 | 3.7679296 | up |
| KPNA2 | 2.22E-07 | 3.7695155 | up |
| MICALCL | 4.92E-07 | 3.7779262 | up |
| BICC1 | 2.92E-07 | 3.7780137 | up |
| PFKP | 9.93E-08 | 3.7814944 | up |
| PRDX4 | 4.87E-08 | 3.7816477 | up |
| NAA35 | 8.51E-08 | 3.7817452 | up |
| LIPE | 1.38E-07 | 3.7834184 | up |
| P3H1 | 2.35E-07 | 3.784074 | up |
| C3orf52 | 1.50E-07 | 3.786085 | up |
| RABAC1 | 3.09E-08 | 3.7864304 | up |
| SCGB3A2 | 1.19E-07 | 3.786699 | up |
| CFHR3 | 1.42E-07 | 3.7952285 | up |
| LOC102723882 | 1.64E-07 | 3.8011246 | up |
| PLK1 | 2.45E-07 | 3.802085 | up |
| BSG | 1.38E-06 | 3.8043764 | up |
| PPP2R5D | 1.41E-07 | 3.8075495 | up |
| NKAIN4 | 6.40E-07 | 3.8130836 | up |
| LSP1 | 6.47E-08 | 3.820023 | up |
| HJURP | 2.32E-07 | 3.8221614 | up |
| KLHL21 | 4.34E-08 | 3.822165 | up |
| PLP2 | 2.12E-08 | 3.8232307 | up |
| LCE1A | 5.20E-08 | 3.831918 | up |
| DSCC1 | 1.13E-06 | 3.8409297 | up |
| ARHGAP44 | 9.94E-08 | 3.8436997 | up |
| GRK6 | 5.11E-08 | 3.8524895 | up |
| FTSJ1 | 7.20E-08 | 3.85817 | up |
| DEFB130 | 1.14E-06 | 3.8585985 | up |
| RSU1 | 3.60E-07 | 3.8610256 | up |
| TMEM52 | 6.10E-08 | 3.8614595 | up |
| FANCA | 3.07E-07 | 3.864011 | up |
| CTPS1 | 4.09E-08 | 3.8644197 | up |
| TMEM217 | 6.02E-07 | 3.8686664 | up |
| NRGN | 2.01E-07 | 3.8709328 | up |
| GRIN3A | 8.66E-07 | 3.8763163 | up |
| DHRS1 | 6.17E-08 | 3.881434 | up |
| EVL | 3.10E-08 | 3.881475 | up |
| ZNF516 | 3.74E-07 | 3.8927817 | up |
| CFH | 7.15E-08 | 3.8928893 | up |
| TAGLN2 | 4.76E-08 | 3.8939447 | up |
| C19orf57 | 3.61E-08 | 3.9075637 | up |
| PRO0628 | 6.96E-08 | 3.9084222 | up |
| SACS | 5.30E-08 | 3.9086833 | up |
| ABCB4 | 5.94E-08 | 3.9091005 | up |
| C11orf80 | 4.19E-07 | 3.922006 | up |
| POTEI | 5.30E-08 | 3.9259233 | up |
| BTNL2 | 6.22E-07 | 3.9282372 | up |
| RUFY2 | 4.03E-07 | 3.9370184 | up |
| ARHGAP30 | 1.13E-07 | 3.9405808 | up |
| LRFN4 | 9.78E-08 | 3.9492154 | up |
| ADI1 | 1.52E-07 | 3.9519877 | up |
| RECQL5 | 9.86E-07 | 3.9521632 | up |
| RANBP1 | 2.02E-07 | 3.953464 | up |
| PRSS22 | 5.42E-07 | 3.9734178 | up |
| LOC100130539 | 3.37E-07 | 3.9766917 | up |
| SSSCA1 | 6.69E-08 | 3.9799576 | up |
| NUDT8 | 2.56E-07 | 3.9805074 | up |
| LOC100131262 | 2.04E-08 | 3.9820707 | up |
| DGAT1 | 8.02E-08 | 3.9859025 | up |
| DNAJA4 | 8.39E-08 | 3.9908173 | up |
| ZNF467 | 1.41E-06 | 3.99124 | up |
| LYPLA2 | 3.02E-07 | 3.9925585 | up |
| CD59 | 1.24E-07 | 3.9965572 | up |
| LRP8 | 8.10E-08 | 4.001638 | up |
| GDPD5 | 9.02E-07 | 4.004391 | up |
| HIST1H4C | 9.95E-08 | 4.0092206 | up |
| MB21D2 | 1.77E-08 | 4.009718 | up |
| CD320 | 2.20E-08 | 4.0098014 | up |
| PLK1 | 5.54E-07 | 4.0135345 | up |
| TPM3 | 6.46E-07 | 4.013671 | up |
| ZDHHC8 | 1.15E-07 | 4.0140777 | up |
| PNPLA3 | 9.17E-08 | 4.015095 | up |
| LOC100128320 | 3.58E-07 | 4.015318 | up |
| TOMM40 | 4.74E-07 | 4.01643 | up |
| ARSJ | 2.34E-07 | 4.016568 | up |
| TIMM8B | 3.11E-08 | 4.0172586 | up |
| TSTA3 | 4.10E-08 | 4.0218062 | up |
| KIF2C | 1.03E-07 | 4.024683 | up |
| EIF2B3 | 3.41E-08 | 4.0299587 | up |
| CTNNB1 | 3.97E-08 | 4.0326757 | up |
| C15orf39 | 7.50E-08 | 4.0348887 | up |
| NKAIN1 | 3.51E-07 | 4.0365987 | up |
| JARID2 | 8.74E-07 | 4.0374794 | up |
| EBP | 7.40E-08 | 4.0440636 | up |
| LOC145474 | 1.60E-07 | 4.0457234 | up |
| CDH2 | 2.53E-07 | 4.047875 | up |
| RNVU1-18 | 5.79E-07 | 4.0529566 | up |
| POLR3H | 2.52E-07 | 4.054966 | up |
| SIX2 | 6.99E-07 | 4.056176 | up |
| MMP15 | 3.18E-07 | 4.058387 | up |
| TOMM34 | 2.11E-08 | 4.06166 | up |
| ACTBL2 | 1.63E-07 | 4.0674424 | up |
| EIF5A | 2.35E-07 | 4.073031 | up |
| GPAM | 2.37E-08 | 4.0746675 | up |
| SLC27A4 | 6.49E-07 | 4.07559 | up |
| ZNF266 | 1.10E-06 | 4.0801706 | up |
| SNTA1 | 3.11E-07 | 4.0801787 | up |
| PTTG3P | 3.35E-08 | 4.085251 | up |
| LYSMD4 | 3.30E-08 | 4.086686 | up |
| SERPINI1 | 8.57E-08 | 4.096923 | up |
| TOMM40 | 8.35E-08 | 4.0986605 | up |
| NIPSNAP3A | 2.10E-08 | 4.1019588 | up |
| IGSF9B | 2.36E-07 | 4.1080394 | up |
| SLC35C1 | 8.69E-08 | 4.110441 | up |
| MYH9 | 9.09E-08 | 4.1189575 | up |
| SACS | 1.91E-07 | 4.1234527 | up |
| MTFP1 | 3.71E-08 | 4.125496 | up |
| ARHGEF18 | 3.92E-08 | 4.1277137 | up |
| AP2A1 | 1.24E-06 | 4.128448 | up |
| PRSS23 | 1.61E-07 | 4.1327114 | up |
| TUBB | 1.84E-08 | 4.1335325 | up |
| ZNF626 | 1.02E-07 | 4.1354833 | up |
| CCDC167 | 1.84E-08 | 4.1360855 | up |
| PHF19 | 8.08E-08 | 4.138548 | up |
| CDK2AP2 | 1.10E-07 | 4.1404486 | up |
| PFKFB4 | 3.61E-08 | 4.143165 | up |
| KDELR3 | 3.85E-08 | 4.14786 | up |
| ZNF738 | 1.80E-07 | 4.1490273 | up |
| PTTG1 | 7.64E-08 | 4.1492214 | up |
| ARL10 | 5.42E-08 | 4.1501017 | up |
| SERPINB3 | 1.20E-06 | 4.1555185 | up |
| HYAL3 | 9.10E-08 | 4.1570644 | up |
| ZNF468 | 3.52E-07 | 4.1622615 | up |
| KIF5C | 5.65E-07 | 4.166392 | up |
| SGOL1 | 1.42E-06 | 4.1673083 | up |
| PIK3CD | 7.02E-07 | 4.168569 | up |
| PORCN | 1.08E-07 | 4.18229 | up |
| MSN | 1.63E-07 | 4.18308 | up |
| DENND2C | 1.01E-07 | 4.1836476 | up |
| FOLR1 | 1.88E-07 | 4.18609 | up |
| GGT5 | 3.89E-08 | 4.1868467 | up |
| TNS1 | 9.19E-08 | 4.190632 | up |
| RGS9 | 4.16E-07 | 4.1908574 | up |
| PCNXL2 | 1.25E-06 | 4.1943927 | up |
| E2F1 | 1.98E-08 | 4.201539 | up |
| NACC1 | 3.39E-07 | 4.204364 | up |
| GPRIN1 | 2.44E-07 | 4.2073555 | up |
| SPRY4 | 8.34E-08 | 4.219673 | up |
| SHC3 | 5.69E-07 | 4.2199006 | up |
| SAPCD2 | 3.97E-07 | 4.224037 | up |
| ACTG1P4 | 6.16E-08 | 4.226524 | up |
| TIMM8B | 3.15E-08 | 4.230243 | up |
| HS3ST3A1 | 2.82E-08 | 4.2319283 | up |
| CCDC167 | 2.29E-07 | 4.2412558 | up |
| CDCA2 | 1.03E-07 | 4.2433605 | up |
| TUBB | 8.08E-08 | 4.243638 | up |
| GPR68 | 2.69E-07 | 4.246865 | up |
| SSBP3 | 3.22E-07 | 4.2525015 | up |
| CYP17A1-AS1 | 2.54E-07 | 4.253999 | up |
| ILK | 1.17E-07 | 4.2580323 | up |
| DTNBP1 | 1.29E-06 | 4.264062 | up |
| lnc-SUSD1-1 | 5.82E-08 | 4.2663245 | up |
| CLTB | 8.06E-08 | 4.2669163 | up |
| AURKA | 5.65E-08 | 4.271295 | up |
| PREP | 6.36E-07 | 4.2826614 | up |
| UBE2S | 1.05E-07 | 4.282849 | up |
| NPAP1 | 3.17E-07 | 4.2833667 | up |
| VCL | 1.37E-07 | 4.2869525 | up |
| IRGQ | 1.31E-06 | 4.291831 | up |
| ARID3A | 1.39E-07 | 4.302092 | up |
| FYN | 1.36E-07 | 4.306751 | up |
| GNAI3 | 5.44E-07 | 4.308419 | up |
| LOC151174 | 1.09E-07 | 4.312176 | up |
| SYNJ2 | 4.45E-07 | 4.3138204 | up |
| FAM160B1 | 1.07E-06 | 4.3176346 | up |
| CXXC4 | 4.85E-07 | 4.318432 | up |
| STARD13 | 1.26E-07 | 4.3202934 | up |
| SMOX | 7.23E-07 | 4.3208923 | up |
| STAP2 | 4.24E-08 | 4.3244805 | up |
| NCS1 | 7.81E-08 | 4.3346553 | up |
| CCBL1 | 5.68E-07 | 4.3387694 | up |
| RNASEH2C | 2.58E-07 | 4.3399053 | up |
| CGREF1 | 2.06E-07 | 4.342258 | up |
| DCAF5 | 1.04E-06 | 4.3479314 | up |
| SAMD4A | 3.45E-08 | 4.352122 | up |
| RIC1 | 1.91E-07 | 4.35525 | up |
| SEC24D | 1.09E-07 | 4.363695 | up |
| PID1 | 1.76E-07 | 4.3655486 | up |
| SHC3 | 4.41E-08 | 4.379696 | up |
| NAV1 | 8.31E-07 | 4.3847256 | up |
| ECI2 | 3.92E-08 | 4.3977604 | up |
| RPS6KB2 | 3.09E-08 | 4.403783 | up |
| PCMT1 | 4.84E-08 | 4.407802 | up |
| AMPD2 | 1.15E-06 | 4.4091783 | up |
| FANCA | 9.10E-07 | 4.41357 | up |
| LHX1 | 5.16E-08 | 4.4148293 | up |
| SAMD4A | 2.46E-08 | 4.430562 | up |
| SLC25A51 | 2.79E-07 | 4.431584 | up |
| FKBP1A | 2.78E-07 | 4.434548 | up |
| ITM2C | 3.71E-08 | 4.4408407 | up |
| ERCC6L | 1.50E-07 | 4.4461265 | up |
| CASC5 | 7.74E-07 | 4.4514737 | up |
| PTTG2 | 1.94E-08 | 4.45547 | up |
| DLGAP4 | 6.23E-07 | 4.4627295 | up |
| BOP1 | 1.90E-07 | 4.476464 | up |
| LOC142937 | 6.46E-08 | 4.4780464 | up |
| C20orf196 | 2.76E-07 | 4.489254 | up |
| CDC42EP2 | 5.44E-08 | 4.4908586 | up |
| SMS | 6.52E-07 | 4.4908686 | up |
| PDLIM5 | 1.14E-07 | 4.4967704 | up |
| DCAF15 | 7.53E-08 | 4.5021563 | up |
| LOC91450 | 1.19E-06 | 4.5127773 | up |
| TUBB | 3.26E-08 | 4.5150137 | up |
| BCAS4 | 3.93E-08 | 4.523903 | up |
| OR7C2 | 2.43E-07 | 4.527525 | up |
| C2orf27A | 1.24E-07 | 4.5282054 | up |
| PNMAL2 | 1.17E-06 | 4.530325 | up |
| CENPI | 1.32E-07 | 4.532988 | up |
| CDKN3 | 1.55E-08 | 4.533964 | up |
| NOC2L | 1.80E-08 | 4.5411983 | up |
| DRAP1 | 9.61E-08 | 4.5433874 | up |
| NMB | 1.16E-07 | 4.546258 | up |
| PLEKHG5 | 7.62E-08 | 4.54924 | up |
| LOC101928710 | 2.22E-07 | 4.5494866 | up |
| LGALS1 | 2.72E-07 | 4.5509324 | up |
| PRPS1L1 | 5.34E-08 | 4.551307 | up |
| H2AFX | 3.97E-07 | 4.5559683 | up |
| MREG | 4.93E-08 | 4.558291 | up |
| ARHGAP27 | 9.10E-08 | 4.580292 | up |
| PARD6G | 7.20E-07 | 4.580343 | up |
| WDR1 | 4.65E-08 | 4.585205 | up |
| MARK4 | 4.26E-07 | 4.587692 | up |
| BAK1 | 1.15E-07 | 4.5919166 | up |
| ARL17A | 2.03E-07 | 4.5933747 | up |
| ZNF28 | 2.23E-07 | 4.5953836 | up |
| PCDH19 | 6.10E-07 | 4.5963306 | up |
| GRK6 | 4.95E-08 | 4.5990424 | up |
| RCL1 | 1.39E-06 | 4.601631 | up |
| KIF20A | 2.91E-07 | 4.603022 | up |
| FKBP1A | 5.74E-07 | 4.6091027 | up |
| PFKP | 1.46E-08 | 4.6093144 | up |
| DGCR11 | 2.44E-07 | 4.613898 | up |
| WASH5P | 3.11E-07 | 4.6141806 | up |
| SH3KBP1 | 1.63E-07 | 4.6149774 | up |
| PSMG4 | 1.06E-06 | 4.616854 | up |
| CD276 | 7.11E-08 | 4.623468 | up |
| LOC100131043 | 6.69E-07 | 4.6304793 | up |
| PCDHGA2 | 2.06E-07 | 4.6312723 | up |
| MAGED1 | 1.86E-07 | 4.6352415 | up |
| SLIT3 | 5.81E-08 | 4.6387753 | up |
| HCG22 | 1.12E-06 | 4.6400056 | up |
| VCP | 1.50E-07 | 4.6504107 | up |
| PDK3 | 7.29E-08 | 4.6524315 | up |
| CORO2B | 1.99E-07 | 4.6527224 | up |
| PLOD1 | 1.04E-07 | 4.6551685 | up |
| ZNF587 | 1.65E-07 | 4.655862 | up |
| CTNND2 | 2.62E-08 | 4.658625 | up |
| HYAL3 | 5.77E-08 | 4.672535 | up |
| NFASC | 1.12E-06 | 4.6773577 | up |
| RAB3A | 1.43E-07 | 4.6783905 | up |
| SORCS2 | 1.29E-07 | 4.68787 | up |
| MYO9B | 5.55E-08 | 4.688989 | up |
| FOXN3 | 5.06E-08 | 4.693179 | up |
| HIST2H2BF | 7.92E-07 | 4.6948395 | up |
| SP6 | 4.06E-07 | 4.697297 | up |
| ATP9A | 1.12E-06 | 4.7057495 | up |
| CLEC11A | 2.12E-07 | 4.713565 | up |
| DOCK9 | 4.91E-07 | 4.722704 | up |
| HES4 | 6.09E-08 | 4.725362 | up |
| S1PR2 | 3.64E-08 | 4.7272606 | up |
| AACS | 8.49E-07 | 4.7294583 | up |
| GUCA2B | 2.50E-07 | 4.730299 | up |
| PVR | 2.53E-07 | 4.737424 | up |
| IGFBP4 | 9.27E-08 | 4.7431884 | up |
| C14orf159 | 2.65E-08 | 4.743665 | up |
| TRIP12 | 5.44E-08 | 4.7445087 | up |
| PCMT1 | 4.17E-08 | 4.753833 | up |
| FHL2 | 7.70E-08 | 4.7576146 | up |
| PCDHGC5 | 7.98E-07 | 4.7618203 | up |
| SH3GL1P2 | 2.50E-07 | 4.770496 | up |
| NOC2L | 5.85E-08 | 4.7758684 | up |
| LRRFIP1 | 1.97E-07 | 4.789611 | up |
| AUNIP | 4.86E-08 | 4.7944074 | up |
| LRR1 | 3.38E-07 | 4.796812 | up |
| ST6GAL1 | 6.66E-08 | 4.8054233 | up |
| LOC100132356 | 1.53E-07 | 4.8057766 | up |
| ASXL1 | 4.45E-07 | 4.806599 | up |
| TCOF1 | 1.89E-08 | 4.8082676 | up |
| AGO2 | 1.76E-08 | 4.808438 | up |
| FAM25A | 3.63E-07 | 4.821416 | up |
| TRPV2 | 3.23E-08 | 4.8224645 | up |
| YIF1B | 8.23E-08 | 4.8268147 | up |
| KIF3C | 5.51E-08 | 4.841147 | up |
| FKBP1A | 2.11E-08 | 4.8436294 | up |
| TRIP13 | 2.08E-08 | 4.8456845 | up |
| RNU2-1 | 4.36E-07 | 4.8473706 | up |
| ACTL9 | 7.56E-07 | 4.849425 | up |
| ZNF783 | 2.32E-08 | 4.8506746 | up |
| ABCA1 | 1.02E-07 | 4.8520083 | up |
| ACAT2 | 6.12E-08 | 4.8531632 | up |
| MSI1 | 3.31E-08 | 4.855634 | up |
| PSMD2 | 6.64E-08 | 4.8653984 | up |
| C11orf24 | 1.14E-07 | 4.873407 | up |
| LPPR2 | 3.07E-07 | 4.8749456 | up |
| HERC2P10 | 6.35E-07 | 4.878876 | up |
| PLA2G4C | 2.19E-08 | 4.881009 | up |
| PCK2 | 5.55E-07 | 4.881382 | up |
| SNORA2A | 7.92E-09 | 4.882843 | up |
| AGMAT | 3.90E-08 | 4.8850965 | up |
| GTSE1 | 2.86E-08 | 4.890118 | up |
| PPP1R14B | 1.67E-08 | 4.8956327 | up |
| SLC2A6 | 5.44E-08 | 4.906748 | up |
| CDC25A | 1.17E-07 | 4.907996 | up |
| BEX5 | 4.64E-08 | 4.9108186 | up |
| ABCD3 | 2.66E-07 | 4.91272 | up |
| RINL | 2.24E-07 | 4.913448 | up |
| FOXP1 | 1.79E-07 | 4.913523 | up |
| HDAC6 | 7.23E-07 | 4.922047 | up |
| DAPK3 | 3.50E-08 | 4.9247923 | up |
| CTNNBIP1 | 4.74E-08 | 4.9375224 | up |
| XLOC_l2_006958 | 1.54E-07 | 4.949296 | up |
| FHL3 | 2.55E-08 | 4.9508243 | up |
| ARNTL | 1.81E-07 | 4.9521646 | up |
| GFPT2 | 2.43E-07 | 4.9556856 | up |
| INPP5A | 7.01E-07 | 4.9658504 | up |
| C21orf58 | 8.77E-07 | 4.9678435 | up |
| SZT2 | 1.47E-07 | 4.9685774 | up |
| LOC100134237 | 2.92E-08 | 4.976239 | up |
| CAP1 | 8.85E-08 | 4.980074 | up |
| ENC1 | 1.05E-06 | 4.9805956 | up |
| ZNF154 | 1.00E-06 | 4.9899025 | up |
| ZNF432 | 1.06E-06 | 5.01466 | up |
| SCARNA12 | 6.95E-07 | 5.015455 | up |
| TGFBR1 | 8.99E-09 | 5.024669 | up |
| RNGTT | 4.24E-07 | 5.0253053 | up |
| NRP2 | 3.60E-07 | 5.031252 | up |
| MAGEH1 | 8.68E-09 | 5.031599 | up |
| CDCA8 | 3.86E-08 | 5.0379987 | up |
| MAP6D1 | 1.02E-06 | 5.053439 | up |
| CD276 | 7.01E-07 | 5.058372 | up |
| TFPT | 2.35E-08 | 5.063874 | up |
| SCFD2 | 1.80E-07 | 5.0697317 | up |
| RIPK4 | 3.44E-08 | 5.0814505 | up |
| STMN1 | 2.13E-08 | 5.086138 | up |
| STARD8 | 6.29E-08 | 5.0987916 | up |
| XXYLT1 | 1.01E-08 | 5.1028953 | up |
| FLNB | 6.75E-08 | 5.116086 | up |
| CDC42 | 3.83E-08 | 5.1188955 | up |
| B3GNT5 | 2.98E-07 | 5.1286774 | up |
| PDSS1 | 6.08E-08 | 5.1299944 | up |
| EGR3 | 2.82E-07 | 5.1311274 | up |
| EIF2B3 | 3.90E-08 | 5.1313734 | up |
| INSIG1 | 5.81E-07 | 5.143347 | up |
| FRMD4A | 1.88E-07 | 5.1455817 | up |
| LILRB3 | 1.35E-06 | 5.1505623 | up |
| HS2ST1 | 1.03E-08 | 5.168514 | up |
| CDK14 | 2.72E-07 | 5.175574 | up |
| SKP2 | 8.21E-08 | 5.183053 | up |
| LOC101929494 | 7.02E-07 | 5.18307 | up |
| CRB2 | 2.95E-07 | 5.1869783 | up |
| VWA1 | 3.82E-07 | 5.1949635 | up |
| FZR1 | 2.13E-08 | 5.1964455 | up |
| SPC25 | 1.15E-08 | 5.2044964 | up |
| DNAJB5 | 2.89E-08 | 5.2082634 | up |
| COL9A3 | 2.12E-07 | 5.218057 | up |
| MAMLD1 | 1.64E-07 | 5.2356453 | up |
| NQO2 | 2.54E-08 | 5.2357726 | up |
| CDKN3 | 1.43E-08 | 5.2364388 | up |
| P3H1 | 9.96E-07 | 5.2374578 | up |
| AGO1 | 2.12E-07 | 5.2515426 | up |
| CAP1 | 4.04E-08 | 5.251566 | up |
| CD99P1 | 1.31E-07 | 5.252112 | up |
| DAAM2 | 3.32E-07 | 5.2701683 | up |
| TSPAN14 | 1.66E-08 | 5.270203 | up |
| RPL39L | 2.08E-08 | 5.2708173 | up |
| NRIP3 | 9.98E-08 | 5.2722645 | up |
| UHRF1 | 4.77E-08 | 5.273623 | up |
| ASPH | 5.15E-08 | 5.2873225 | up |
| CLDN7 | 3.75E-08 | 5.2934356 | up |
| HMGB3 | 8.85E-09 | 5.305034 | up |
| NREP | 5.74E-09 | 5.3130846 | up |
| SURF4 | 5.29E-08 | 5.330515 | up |
| HHIP-AS1 | 1.57E-08 | 5.3322067 | up |
| MOB3B | 7.48E-07 | 5.351524 | up |
| DIAPH3 | 8.15E-07 | 5.3523183 | up |
| PGM2L1 | 4.32E-08 | 5.3542786 | up |
| CFL1 | 2.66E-07 | 5.3584113 | up |
| MAD2L1 | 4.01E-07 | 5.376043 | up |
| LOC100131289 | 6.13E-07 | 5.3813515 | up |
| PPME1 | 6.11E-07 | 5.386558 | up |
| SH3RF2 | 7.62E-09 | 5.393891 | up |
| JAM2 | 2.25E-08 | 5.399533 | up |
| GDF5 | 4.48E-07 | 5.4024606 | up |
| TUBA3C | 1.22E-07 | 5.417817 | up |
| RRBP1 | 8.19E-07 | 5.4216347 | up |
| CCNC | 7.48E-08 | 5.4268966 | up |
| PFKFB3 | 2.01E-08 | 5.428147 | up |
| WASF3 | 1.61E-07 | 5.435509 | up |
| KLHL25 | 1.21E-07 | 5.4371605 | up |
| RASSF10 | 1.31E-06 | 5.437661 | up |
| NOL4L | 1.37E-08 | 5.480991 | up |
| VWCE | 5.55E-08 | 5.4833984 | up |
| BVES | 8.06E-08 | 5.4847794 | up |
| ABLIM3 | 1.74E-07 | 5.494138 | up |
| LCTL | 3.28E-07 | 5.496491 | up |
| PLXNA3 | 1.22E-08 | 5.5038924 | up |
| SMARCA4 | 1.21E-07 | 5.509472 | up |
| HOXA11-AS | 2.37E-07 | 5.521415 | up |
| PLAGL1 | 3.24E-08 | 5.525938 | up |
| TUBB8 | 3.89E-08 | 5.5273547 | up |
| SRRD | 6.20E-08 | 5.5317674 | up |
| PLAT | 2.07E-08 | 5.533579 | up |
| DBN1 | 1.46E-08 | 5.5345345 | up |
| ARL9 | 1.12E-07 | 5.5370173 | up |
| SOX13 | 9.38E-07 | 5.551539 | up |
| PTPRK | 1.20E-07 | 5.555767 | up |
| HIPK2 | 3.93E-08 | 5.5639725 | up |
| APOA1 | 5.67E-08 | 5.576825 | up |
| FBLN1 | 1.04E-08 | 5.5831923 | up |
| KIAA0754 | 6.91E-07 | 5.585563 | up |
| CLPB | 4.82E-08 | 5.594683 | up |
| WDR1 | 7.21E-08 | 5.595658 | up |
| ARHGAP31 | 1.33E-07 | 5.599137 | up |
| MFAP5 | 6.00E-07 | 5.6062403 | up |
| TUBB6 | 1.16E-07 | 5.608248 | up |
| ARHGAP23 | 9.18E-09 | 5.62295 | up |
| C14orf132 | 2.28E-08 | 5.625349 | up |
| FEM1B | 2.37E-07 | 5.635929 | up |
| BEGAIN | 5.61E-08 | 5.6363807 | up |
| JAM2 | 1.50E-07 | 5.6403008 | up |
| NKAIN4 | 1.94E-08 | 5.6455336 | up |
| RPS15AP10 | 3.25E-08 | 5.6483626 | up |
| SCARNA7 | 8.73E-07 | 5.6708407 | up |
| UBE2C | 3.44E-07 | 5.676475 | up |
| ADAM12 | 1.36E-06 | 5.689684 | up |
| SNAI1 | 1.78E-07 | 5.7122326 | up |
| SATB1 | 1.41E-06 | 5.7618847 | up |
| LYPD6 | 2.09E-07 | 5.7713666 | up |
| LOC283856 | 9.26E-07 | 5.7721224 | up |
| DEF8 | 9.30E-09 | 5.791333 | up |
| ZFP64 | 7.04E-08 | 5.791605 | up |
| EBPL | 1.74E-07 | 5.7957296 | up |
| CFL1 | 4.41E-08 | 5.80296 | up |
| NRIP3 | 3.35E-07 | 5.8149786 | up |
| MEX3A | 8.30E-09 | 5.816036 | up |
| GSTM4 | 4.61E-08 | 5.82554 | up |
| PLEKHG3 | 1.50E-08 | 5.8325663 | up |
| GAL | 1.20E-08 | 5.8332787 | up |
| PPP1R14B | 1.81E-08 | 5.8405256 | up |
| SEPW1 | 3.82E-08 | 5.8409195 | up |
| CDC20 | 7.47E-08 | 5.84332 | up |
| L3MBTL1 | 9.01E-07 | 5.8441696 | up |
| SPHK1 | 1.43E-08 | 5.8519425 | up |
| M1AP | 7.21E-07 | 5.8578854 | up |
| FAM129B | 2.69E-08 | 5.858872 | up |
| S100A2 | 4.17E-08 | 5.8748803 | up |
| SH3GL3 | 1.44E-07 | 5.878495 | up |
| PLEKHG3 | 7.25E-09 | 5.893583 | up |
| ARG2 | 1.01E-07 | 5.906965 | up |
| CD99 | 3.61E-08 | 5.9096775 | up |
| ASMTL | 5.10E-08 | 5.9255905 | up |
| GJD3 | 7.55E-07 | 5.9280634 | up |
| TCF7 | 6.72E-09 | 5.9303164 | up |
| FOXD1 | 1.67E-07 | 5.9438562 | up |
| B4GALNT4 | 1.03E-06 | 5.9440627 | up |
| NES | 3.70E-07 | 5.9459195 | up |
| CDH11 | 4.36E-07 | 5.947595 | up |
| BCAR1 | 6.71E-07 | 5.950913 | up |
| DBH-AS1 | 9.65E-08 | 5.9715934 | up |
| AJAP1 | 8.88E-07 | 5.9724393 | up |
| CCDC134 | 8.01E-07 | 6.004463 | up |
| TGFB3 | 8.11E-07 | 6.025668 | up |
| LOC100129675 | 1.26E-06 | 6.0366306 | up |
| TMEM190 | 1.52E-07 | 6.039172 | up |
| CPAMD8 | 2.30E-08 | 6.0414333 | up |
| CDC37 | 2.35E-07 | 6.060518 | up |
| MFSD2A | 8.18E-08 | 6.0907497 | up |
| EHD2 | 6.94E-09 | 6.0908623 | up |
| MICA | 7.90E-08 | 6.094856 | up |
| TUBB4B | 6.19E-08 | 6.113177 | up |
| MARCH2 | 2.44E-08 | 6.1180997 | up |
| ZNF813 | 8.05E-08 | 6.1211233 | up |
| ASTN2 | 3.29E-07 | 6.1239524 | up |
| RN7SL1 | 1.47E-07 | 6.130436 | up |
| C10orf55 | 1.37E-06 | 6.1586895 | up |
| ANGPTL2 | 1.13E-08 | 6.1600294 | up |
| PRDM5 | 3.39E-07 | 6.162642 | up |
| DEF8 | 2.96E-08 | 6.171218 | up |
| LOC100506191 | 1.10E-06 | 6.189789 | up |
| UHRF1 | 1.50E-08 | 6.1939096 | up |
| DSEL | 3.54E-07 | 6.1983414 | up |
| COL5A1 | 2.06E-07 | 6.1999006 | up |
| CD82 | 1.65E-08 | 6.204066 | up |
| UBR4 | 8.51E-07 | 6.2119017 | up |
| PTPRS | 5.29E-07 | 6.2166557 | up |
| SOX9-AS1 | 3.66E-07 | 6.222358 | up |
| DTNA | 2.69E-07 | 6.2235203 | up |
| ENOX1 | 1.26E-06 | 6.2289686 | up |
| TRIP13 | 7.99E-07 | 6.237514 | up |
| GPR143 | 5.99E-07 | 6.2418427 | up |
| NFASC | 8.49E-08 | 6.265433 | up |
| INSR | 2.03E-07 | 6.2892585 | up |
| AP1S2 | 8.73E-08 | 6.2915945 | up |
| DDX3Y | 2.93E-07 | 6.302842 | up |
| SEC14L2 | 2.60E-07 | 6.303637 | up |
| MBOAT7 | 1.39E-08 | 6.320655 | up |
| NTN1 | 3.72E-07 | 6.328121 | up |
| ZNF600 | 2.04E-07 | 6.3304915 | up |
| lnc-MRPL14-1 | 6.51E-07 | 6.3419104 | up |
| LOC100131541 | 5.55E-09 | 6.3428526 | up |
| ZNF814 | 1.13E-07 | 6.3436275 | up |
| LOC102723367 | 7.74E-07 | 6.3522587 | up |
| SPC24 | 5.20E-07 | 6.3628516 | up |
| TSPAN14 | 7.36E-08 | 6.3631415 | up |
| SSC5D | 3.96E-07 | 6.369294 | up |
| MPZL3 | 6.95E-07 | 6.3744917 | up |
| IRAK3 | 3.37E-07 | 6.37526 | up |
| LINC00842 | 1.30E-07 | 6.379461 | up |
| IP6K2 | 7.21E-07 | 6.380065 | up |
| PCYT1A | 6.76E-08 | 6.3861513 | up |
| LETM1 | 1.55E-07 | 6.3865356 | up |
| MISP | 9.30E-07 | 6.4036407 | up |
| LOC100132495 | 1.42E-06 | 6.4052663 | up |
| TMEM63C | 6.43E-08 | 6.410703 | up |
| REN | 7.46E-08 | 6.4124284 | up |
| ZNF454 | 1.14E-06 | 6.4260836 | up |
| MPZL3 | 1.23E-08 | 6.4414535 | up |
| RCAN3 | 2.11E-08 | 6.445727 | up |
| FAM214B | 4.26E-07 | 6.4522247 | up |
| ABCC10 | 1.04E-06 | 6.454054 | up |
| MFSD12 | 1.19E-06 | 6.454649 | up |
| CCNB1 | 2.11E-08 | 6.459788 | up |
| SEPW1 | 7.40E-09 | 6.4615636 | up |
| SLMAP | 1.49E-08 | 6.468663 | up |
| GSTA4 | 1.74E-08 | 6.4870486 | up |
| ZNF521 | 1.10E-07 | 6.5082474 | up |
| HN1 | 1.50E-08 | 6.5205526 | up |
| PLEK2 | 1.18E-08 | 6.526767 | up |
| HN1 | 4.23E-09 | 6.5273814 | up |
| CCNA2 | 2.87E-08 | 6.5320463 | up |
| SMTN | 3.50E-08 | 6.5436244 | up |
| LINC00087 | 3.52E-09 | 6.549279 | up |
| ST7-OT4 | 2.98E-07 | 6.5545554 | up |
| AUTS2 | 1.78E-08 | 6.5632105 | up |
| ATP8A1 | 1.07E-07 | 6.568982 | up |
| GCH1 | 2.39E-07 | 6.5794806 | up |
| ZNF521 | 7.70E-07 | 6.5922246 | up |
| AQP1 | 4.20E-08 | 6.6238084 | up |
| CISH | 2.42E-07 | 6.6266975 | up |
| TNS1 | 1.29E-06 | 6.6298842 | up |
| UCA1 | 4.23E-09 | 6.6479025 | up |
| SHBG | 3.07E-07 | 6.656134 | up |
| EFR3B | 4.48E-08 | 6.670789 | up |
| EVA1A | 9.48E-09 | 6.672954 | up |
| STAR | 6.13E-07 | 6.705502 | up |
| lnc-AF131215,3,1-1 | 6.38E-08 | 6.7258954 | up |
| HOMER1 | 5.26E-08 | 6.7298913 | up |
| GCNT4 | 1.36E-07 | 6.7307687 | up |
| RRBP1 | 1.28E-06 | 6.7618093 | up |
| C1orf21 | 5.66E-08 | 6.7620363 | up |
| S100A3 | 1.63E-08 | 6.767165 | up |
| HS3ST3B1 | 6.39E-09 | 6.767205 | up |
| CCDC28B | 3.56E-09 | 6.795398 | up |
| FAM84A | 1.64E-08 | 6.8127966 | up |
| ASB2 | 4.64E-07 | 6.8224974 | up |
| PMEPA1 | 1.81E-08 | 6.8228154 | up |
| ZNF491 | 1.36E-06 | 6.8315163 | up |
| DUSP4 | 4.65E-09 | 6.8412495 | up |
| SCARNA16 | 1.34E-07 | 6.8414574 | up |
| PRDX2 | 4.29E-08 | 6.8429317 | up |
| CLSTN2 | 1.02E-07 | 6.8458524 | up |
| MLLT11 | 7.79E-09 | 6.8545003 | up |
| LRRN2 | 8.61E-09 | 6.858187 | up |
| LOC648987 | 2.23E-08 | 6.888811 | up |
| P4HA3 | 1.16E-06 | 6.8942275 | up |
| LHFPL3-AS2 | 1.07E-07 | 6.905507 | up |
| NRN1 | 4.70E-08 | 6.91186 | up |
| CNTNAP3 | 1.71E-08 | 6.9158883 | up |
| SH3BGRL3 | 2.73E-08 | 6.923546 | up |
| PCBP4 | 7.19E-09 | 6.926283 | up |
| TUBA4A | 1.45E-07 | 6.9387136 | up |
| FLJ11710 | 4.05E-08 | 6.940803 | up |
| AP1S2 | 2.52E-08 | 6.9450555 | up |
| LOC400743 | 1.15E-06 | 6.9456453 | up |
| HAS1 | 1.09E-06 | 6.9487276 | up |
| PSMD2 | 4.08E-07 | 6.9498615 | up |
| UBASH3B | 1.08E-08 | 6.9526415 | up |
| ZNF316 | 1.35E-07 | 6.954604 | up |
| GSTA4 | 4.35E-08 | 6.962993 | up |
| TSHZ2 | 1.33E-06 | 6.991112 | up |
| TACC3 | 4.47E-08 | 6.9938116 | up |
| SLC16A8 | 6.41E-07 | 6.9969816 | up |
| ART5 | 4.07E-08 | 6.9989047 | up |
| PTHLH | 6.18E-09 | 6.9989657 | up |
| GSN | 1.12E-08 | 7.0008445 | up |
| LOC100128517 | 1.10E-07 | 7.026316 | up |
| PIK3R3 | 6.29E-08 | 7.040531 | up |
| KALRN | 5.09E-07 | 7.0453944 | up |
| ASIC1 | 1.09E-07 | 7.055321 | up |
| GRASP | 1.89E-07 | 7.0588317 | up |
| SPATA5L1 | 5.67E-07 | 7.0678244 | up |
| LOC728061 | 3.48E-09 | 7.069073 | up |
| SPEF2 | 1.01E-06 | 7.077652 | up |
| PDGFB | 6.28E-08 | 7.083788 | up |
| AMTN | 5.57E-07 | 7.1158876 | up |
| KIAA2022 | 8.27E-07 | 7.130117 | up |
| KIAA1549L | 5.75E-09 | 7.1504364 | up |
| RTKN2 | 1.10E-07 | 7.1531606 | up |
| SERPINE2 | 1.15E-07 | 7.1592546 | up |
| ZNF578 | 1.03E-06 | 7.168161 | up |
| EDNRA | 2.41E-07 | 7.172786 | up |
| RTN2 | 5.12E-09 | 7.1849604 | up |
| LOC284219 | 5.95E-09 | 7.188842 | up |
| DBT | 4.30E-07 | 7.192787 | up |
| SFMBT2 | 1.31E-06 | 7.2003713 | up |
| SYNPO2L | 8.79E-08 | 7.2085567 | up |
| POP1 | 4.29E-09 | 7.240079 | up |
| KIF21B | 1.61E-07 | 7.249517 | up |
| LRRC8A | 1.83E-08 | 7.252629 | up |
| S100A16 | 7.42E-09 | 7.260466 | up |
| TSPAN14 | 6.18E-08 | 7.260874 | up |
| GCH1 | 5.41E-08 | 7.261061 | up |
| RABL6 | 8.22E-09 | 7.2625732 | up |
| CACNG6 | 3.16E-07 | 7.2768335 | up |
| LAMC2 | 1.34E-08 | 7.279547 | up |
| PPP1R14C | 2.20E-08 | 7.2844605 | up |
| TUBA4A | 1.54E-08 | 7.2893753 | up |
| PDLIM3 | 5.31E-07 | 7.2987666 | up |
| GPR171 | 5.80E-07 | 7.3235765 | up |
| FRAS1 | 1.02E-06 | 7.3285728 | up |
| NUDT10 | 7.81E-09 | 7.3294463 | up |
| LAMA4 | 3.42E-07 | 7.3636966 | up |
| REC8 | 1.02E-08 | 7.3676686 | up |
| SLC16A2 | 3.38E-09 | 7.387378 | up |
| CEP44 | 8.13E-08 | 7.397968 | up |
| GCAT | 2.06E-08 | 7.4199777 | up |
| lnc-ZNF674-3 | 1.44E-08 | 7.426774 | up |
| NF2 | 1.35E-08 | 7.428872 | up |
| TFEB | 6.44E-08 | 7.430829 | up |
| ANGPTL4 | 6.93E-09 | 7.4323545 | up |
| FCER2 | 4.88E-08 | 7.4705443 | up |
| HS1BP3 | 5.20E-09 | 7.4722176 | up |
| SND1-IT1 | 2.69E-08 | 7.487685 | up |
| LOC729683 | 9.37E-07 | 7.504147 | up |
| CENPW | 8.12E-08 | 7.506045 | up |
| PMEPA1 | 7.24E-08 | 7.507426 | up |
| PCBP4 | 3.33E-09 | 7.53383 | up |
| GSN | 2.10E-07 | 7.53646 | up |
| ITGA5 | 7.14E-09 | 7.542305 | up |
| LIN28B | 2.68E-07 | 7.5474653 | up |
| LYPD6 | 1.24E-07 | 7.5628304 | up |
| SPOCK2 | 6.12E-09 | 7.569282 | up |
| VPS9D1-AS1 | 1.21E-06 | 7.5719895 | up |
| TBC1D19 | 3.09E-09 | 7.577416 | up |
| KRTAP4-2 | 2.41E-07 | 7.5878644 | up |
| ZNF667 | 2.47E-07 | 7.592336 | up |
| CSRP1 | 6.24E-08 | 7.6084046 | up |
| ARID5A | 6.35E-09 | 7.609467 | up |
| OSTF1 | 8.29E-09 | 7.619447 | up |
| ACTN1 | 7.39E-09 | 7.6414223 | up |
| BCL11B | 1.65E-07 | 7.64863 | up |
| HSPA2 | 2.27E-08 | 7.6837707 | up |
| AMIGO2 | 3.56E-08 | 7.690033 | up |
| DANCR | 1.08E-08 | 7.71289 | up |
| CA3 | 1.22E-06 | 7.7326226 | up |
| BEND4 | 9.61E-07 | 7.736392 | up |
| HMGB3P1 | 7.53E-07 | 7.7534647 | up |
| RIPK3 | 7.25E-07 | 7.758238 | up |
| RNF125 | 2.96E-09 | 7.7861304 | up |
| ACTN3 | 5.27E-08 | 7.8175693 | up |
| TCEA3 | 2.11E-07 | 7.826758 | up |
| C14orf37 | 5.39E-09 | 7.8345637 | up |
| KLHL4 | 1.43E-06 | 7.8683305 | up |
| PAK3 | 7.20E-08 | 7.8812933 | up |
| KDF1 | 2.49E-07 | 7.899395 | up |
| HOXD1 | 3.76E-08 | 7.9016366 | up |
| PIANP | 1.56E-07 | 7.905065 | up |
| EVA1A | 1.20E-08 | 7.915442 | up |
| ZNF808 | 4.14E-08 | 7.927411 | up |
| LUZP1 | 4.01E-07 | 7.936718 | up |
| SLC2A1 | 2.34E-08 | 7.9492064 | up |
| QPCTL | 2.10E-07 | 7.9687862 | up |
| DCBLD2 | 6.75E-09 | 7.97499 | up |
| NCMAP | 8.90E-07 | 8.001543 | up |
| COL5A2 | 8.66E-09 | 8.002084 | up |
| SERPINA1 | 1.57E-08 | 8.014078 | up |
| FLJ16779 | 9.52E-09 | 8.01534 | up |
| RNF122 | 2.70E-08 | 8.026483 | up |
| IGSF23 | 1.15E-07 | 8.033031 | up |
| RNF150 | 7.90E-08 | 8.044275 | up |
| GPAT2 | 1.85E-07 | 8.081989 | up |
| TMEM158 | 8.89E-08 | 8.08551 | up |
| ZNF600 | 6.64E-09 | 8.086189 | up |
| FOXC1 | 2.03E-08 | 8.099493 | up |
| HMSD | 9.30E-07 | 8.113025 | up |
| KRT14 | 1.08E-06 | 8.116503 | up |
| IFNE | 5.16E-07 | 8.126406 | up |
| HEY1 | 2.02E-08 | 8.128345 | up |
| FBLIM1 | 4.79E-07 | 8.139618 | up |
| CORO1A | 5.23E-07 | 8.153687 | up |
| FHDC1 | 1.22E-07 | 8.161473 | up |
| KCNQ5-IT1 | 3.11E-08 | 8.161783 | up |
| MYB | 1.03E-06 | 8.162545 | up |
| MMP13 | 8.17E-07 | 8.187754 | up |
| SNORD3B-1 | 4.05E-08 | 8.1899395 | up |
| LIMA1 | 2.64E-09 | 8.197415 | up |
| NCK2 | 3.45E-09 | 8.199234 | up |
| PLCXD1 | 7.50E-09 | 8.221141 | up |
| INADL | 2.05E-08 | 8.284604 | up |
| M1AP | 1.99E-07 | 8.320495 | up |
| MYL2 | 3.27E-07 | 8.325773 | up |
| SERINC2 | 3.26E-07 | 8.336711 | up |
| INPP4B | 1.39E-06 | 8.371037 | up |
| GREB1L | 6.15E-07 | 8.374751 | up |
| B3GNT3 | 1.35E-06 | 8.403227 | up |
| SUSD3 | 3.74E-08 | 8.429712 | up |
| ZNF320 | 2.71E-08 | 8.431563 | up |
| lnc-MAB21L2-1 | 1.08E-08 | 8.432043 | up |
| LSR | 1.64E-08 | 8.435187 | up |
| JPH1 | 5.73E-08 | 8.453844 | up |
| GLIPR2 | 1.36E-07 | 8.454258 | up |
| EFHD1 | 1.62E-07 | 8.457785 | up |
| MYL7 | 5.25E-08 | 8.461902 | up |
| TOX3 | 2.11E-08 | 8.496836 | up |
| PHACTR1 | 5.28E-07 | 8.500037 | up |
| ZNF677 | 4.72E-07 | 8.51672 | up |
| CPE | 5.02E-09 | 8.531974 | up |
| PTGS1 | 7.29E-07 | 8.534202 | up |
| GPR183 | 1.30E-06 | 8.5399 | up |
| TUBA4A | 4.72E-08 | 8.541042 | up |
| CD69 | 2.25E-07 | 8.561541 | up |
| SLC22A25 | 4.21E-08 | 8.574926 | up |
| RGN | 1.18E-06 | 8.576441 | up |
| DSEL | 8.22E-08 | 8.601591 | up |
| KCNK6 | 5.90E-08 | 8.601723 | up |
| SNCAIP | 2.32E-07 | 8.608142 | up |
| ADAM12 | 5.56E-08 | 8.611002 | up |
| PPM1F | 5.04E-08 | 8.6198225 | up |
| ID3 | 1.40E-06 | 8.638916 | up |
| GPER1 | 1.18E-07 | 8.656955 | up |
| SLC38A5 | 7.25E-08 | 8.685711 | up |
| DNAJB5 | 1.83E-07 | 8.691637 | up |
| C1orf21 | 1.46E-08 | 8.69193 | up |
| ST3GAL5 | 3.01E-08 | 8.725065 | up |
| ADAMTS7 | 4.84E-08 | 8.758438 | up |
| SLITRK4 | 2.40E-09 | 8.78304 | up |
| TNFRSF21 | 1.51E-09 | 8.812837 | up |
| BST2 | 5.61E-08 | 8.82007 | up |
| FAR2 | 4.84E-09 | 8.829683 | up |
| ZNF528 | 6.60E-07 | 8.859553 | up |
| AKAP12 | 3.97E-09 | 8.887473 | up |
| PCDHGA5 | 1.77E-07 | 8.906711 | up |
| C14orf37 | 5.64E-08 | 8.943627 | up |
| HAVCR2 | 2.27E-07 | 8.943878 | up |
| TINAG | 2.66E-07 | 8.968016 | up |
| MEF2BNB | 4.19E-08 | 9.041585 | up |
| ZNF93 | 5.94E-08 | 9.072358 | up |
| TGFBI | 5.87E-09 | 9.072536 | up |
| FGF5 | 3.28E-08 | 9.0848465 | up |
| AKAP12 | 3.12E-08 | 9.10381 | up |
| TUBB2A | 2.12E-09 | 9.108583 | up |
| FGFBP1 | 6.40E-07 | 9.139853 | up |
| SNAI2 | 1.67E-07 | 9.144138 | up |
| WDR37 | 4.41E-07 | 9.197222 | up |
| TBX3 | 5.68E-07 | 9.250362 | up |
| MYBL2 | 9.04E-08 | 9.259083 | up |
| TUBB3 | 3.82E-08 | 9.265077 | up |
| NREP | 2.89E-08 | 9.265691 | up |
| GDNF | 4.71E-07 | 9.2809305 | up |
| PTPRR | 1.07E-07 | 9.291497 | up |
| IFFO2 | 3.31E-09 | 9.298261 | up |
| ELL2 | 3.48E-09 | 9.31488 | up |
| FAM64A | 4.27E-08 | 9.329907 | up |
| PDE1C | 1.87E-08 | 9.336484 | up |
| BMP1 | 1.10E-07 | 9.358431 | up |
| WDR6 | 9.27E-07 | 9.370615 | up |
| SHC4 | 2.27E-07 | 9.374663 | up |
| LOC100129940 | 6.44E-07 | 9.389433 | up |
| SPESP1 | 3.45E-07 | 9.391028 | up |
| TFF3 | 1.40E-06 | 9.395007 | up |
| FBN1 | 1.24E-08 | 9.426623 | up |
| GAS7 | 1.54E-07 | 9.455751 | up |
| FOXO1 | 3.40E-08 | 9.457973 | up |
| SH3PXD2B | 2.55E-07 | 9.502768 | up |
| GLIPR2 | 4.20E-09 | 9.510084 | up |
| TCL1B | 4.49E-08 | 9.586019 | up |
| THSD4 | 3.28E-07 | 9.588088 | up |
| LCK | 1.69E-07 | 9.601275 | up |
| LOC100131829 | 4.79E-09 | 9.620285 | up |
| SIGLEC15 | 7.82E-09 | 9.627386 | up |
| FAM110A | 4.72E-08 | 9.644326 | up |
| HRCT1 | 3.19E-07 | 9.68027 | up |
| TBX3 | 1.01E-06 | 9.687283 | up |
| lnc-AF131215,4,1-1 | 4.67E-07 | 9.687302 | up |
| HSD17B1 | 3.03E-08 | 9.707934 | up |
| TPM4 | 2.45E-09 | 9.7294655 | up |
| C2CD4B | 1.86E-07 | 9.747587 | up |
| CBLN3 | 4.52E-07 | 9.766737 | up |
| LOC100128001 | 6.77E-07 | 9.76731 | up |
| GYS1 | 1.71E-09 | 9.777237 | up |
| RASSF5 | 3.38E-07 | 9.79761 | up |
| NNAT | 3.41E-09 | 9.809561 | up |
| SNORA62 | 7.38E-08 | 9.877494 | up |
| SETD3 | 2.06E-07 | 9.893852 | up |
| FAM132B | 8.09E-09 | 9.935636 | up |
| HPDL | 4.30E-09 | 9.948652 | up |
| DCLK2 | 2.17E-07 | 9.951979 | up |
| COL3A1 | 2.30E-07 | 9.999993 | up |
| PROC | 4.18E-09 | 10.003977 | up |
| MAP7D3 | 2.48E-07 | 10.009535 | up |
| DZIP1 | 3.18E-09 | 10.049561 | up |
| C19orf18 | 5.54E-07 | 10.103929 | up |
| FAM110B | 1.51E-09 | 10.114059 | up |
| DSC2 | 5.39E-07 | 10.162354 | up |
| FILIP1L | 1.87E-08 | 10.198611 | up |
| C4orf33 | 3.44E-07 | 10.228947 | up |
| WNK4 | 4.78E-07 | 10.252225 | up |
| PDE1C | 1.32E-07 | 10.258271 | up |
| DACT2 | 4.53E-07 | 10.334093 | up |
| GALNT3 | 1.20E-07 | 10.39269 | up |
| ZNF28 | 1.21E-07 | 10.429085 | up |
| XYLT1 | 8.35E-09 | 10.432158 | up |
| SPC24 | 3.23E-09 | 10.439572 | up |
| DSP | 2.06E-08 | 10.45018 | up |
| CFAP45 | 3.64E-09 | 10.458367 | up |
| UCN2 | 2.09E-09 | 10.493917 | up |
| GJA1 | 2.50E-08 | 10.52944 | up |
| EFHD1 | 6.19E-09 | 10.530233 | up |
| PDGFRB | 9.04E-08 | 10.543539 | up |
| LAMC2 | 1.76E-08 | 10.657039 | up |
| AKAP12 | 6.02E-09 | 10.670864 | up |
| LMCD1 | 2.04E-08 | 10.674363 | up |
| PRRX1 | 1.79E-07 | 10.696815 | up |
| TAP2 | 2.44E-07 | 10.714495 | up |
| SDR42E1 | 1.05E-08 | 10.810549 | up |
| GAP43 | 1.06E-07 | 10.819977 | up |
| KCNK3 | 1.27E-08 | 10.820369 | up |
| ANKRD65 | 6.10E-07 | 10.867415 | up |
| NRK | 1.64E-08 | 10.872874 | up |
| FAM46B | 1.10E-07 | 10.899773 | up |
| MMP7 | 1.97E-09 | 10.956842 | up |
| ABAT | 3.58E-09 | 10.957562 | up |
| SGK1 | 1.12E-09 | 10.975938 | up |
| NCAM1 | 2.37E-09 | 10.990428 | up |
| THSD7A | 1.17E-07 | 11.004332 | up |
| ZNF320 | 1.58E-07 | 11.004453 | up |
| FABP5 | 3.16E-09 | 11.018463 | up |
| DENND1C | 7.41E-08 | 11.052313 | up |
| PHACTR1 | 1.33E-07 | 11.103926 | up |
| IFITM1 | 1.27E-08 | 11.161489 | up |
| PDE1C | 5.28E-09 | 11.186197 | up |
| IFITM1 | 2.22E-09 | 11.19669 | up |
| TPM4 | 4.88E-08 | 11.233032 | up |
| GYG2 | 5.93E-08 | 11.268346 | up |
| CPXM1 | 1.32E-06 | 11.282278 | up |
| UTY | 1.16E-07 | 11.316974 | up |
| LRRC38 | 7.44E-07 | 11.355686 | up |
| MYLK2 | 1.24E-08 | 11.378724 | up |
| IQSEC3 | 1.38E-06 | 11.419052 | up |
| FABP5 | 1.05E-07 | 11.436907 | up |
| ZNF468 | 2.36E-08 | 11.44852 | up |
| FABP5 | 1.82E-08 | 11.450091 | up |
| RPL23AP32 | 1.59E-09 | 11.467436 | up |
| ADCY4 | 6.27E-08 | 11.488204 | up |
| DOCK8 | 1.28E-07 | 11.492676 | up |
| KHDRBS3 | 6.54E-09 | 11.50379 | up |
| SAMD5 | 1.06E-06 | 11.50684 | up |
| GPER1 | 7.42E-09 | 11.513585 | up |
| TMSB15B | 2.05E-09 | 11.516854 | up |
| BCL11A | 3.53E-09 | 11.519622 | up |
| C17orf104 | 3.73E-07 | 11.560783 | up |
| PKNOX2 | 1.67E-07 | 11.57112 | up |
| FST | 1.89E-07 | 11.580327 | up |
| GLIPR1 | 1.08E-09 | 11.648916 | up |
| LOC100133299 | 1.59E-08 | 11.681021 | up |
| ASAP1-IT1 | 1.82E-07 | 11.699047 | up |
| DAAM2 | 4.42E-08 | 11.7 | up |
| FAM153C | 1.15E-06 | 11.702438 | up |
| HIVEP3 | 1.19E-08 | 11.731078 | up |
| CGB | 1.23E-08 | 11.756518 | up |
| CPNE1 | 9.46E-09 | 11.775885 | up |
| OGFRP1 | 4.29E-07 | 11.812169 | up |
| BACH2 | 7.24E-08 | 11.817328 | up |
| ZNF702P | 4.76E-07 | 11.855381 | up |
| RNA5-8S5 | 4.02E-07 | 11.903486 | up |
| MPPED2 | 5.50E-07 | 11.904057 | up |
| PSG10P | 9.99E-08 | 11.909188 | up |
| ANXA2R | 3.20E-08 | 11.952174 | up |
| MSN | 3.96E-08 | 11.998143 | up |
| LINC01191 | 8.00E-07 | 12.004347 | up |
| ALDH1B1 | 2.22E-09 | 12.026389 | up |
| EBF1 | 1.14E-07 | 12.064123 | up |
| NT5DC2 | 1.04E-08 | 12.075455 | up |
| LINC00900 | 2.10E-07 | 12.084094 | up |
| ARSI | 1.82E-07 | 12.088943 | up |
| LOC100129473 | 1.33E-08 | 12.09607 | up |
| CD274 | 5.75E-08 | 12.119205 | up |
| PF4 | 5.85E-07 | 12.162905 | up |
| ENHO | 6.31E-08 | 12.169799 | up |
| SESN3 | 1.91E-07 | 12.197265 | up |
| KLK6 | 9.77E-08 | 12.197357 | up |
| CNKSR1 | 4.89E-08 | 12.201966 | up |
| GPRIN2 | 7.29E-09 | 12.209845 | up |
| PTGER4 | 4.36E-09 | 12.252284 | up |
| MRVI1 | 1.85E-07 | 12.378502 | up |
| MMP16 | 1.92E-07 | 12.389601 | up |
| KLF12 | 1.02E-07 | 12.392991 | up |
| FAM43A | 6.22E-09 | 12.413718 | up |
| PRSS1 | 2.47E-08 | 12.421468 | up |
| DSP | 2.19E-09 | 12.432557 | up |
| TIAM2 | 3.43E-09 | 12.490562 | up |
| SLC16A10 | 3.28E-09 | 12.507113 | up |
| PRSS8 | 1.26E-08 | 12.572027 | up |
| GABRB1 | 6.40E-08 | 12.642059 | up |
| FKBP1B | 1.11E-09 | 12.748929 | up |
| TGM2 | 5.28E-09 | 12.755972 | up |
| PEG3 | 4.28E-07 | 12.816487 | up |
| FGF5 | 3.95E-07 | 12.867023 | up |
| DCLK2 | 2.64E-08 | 12.882441 | up |
| ADAM23 | 1.66E-07 | 12.925436 | up |
| SYNJ2 | 2.50E-08 | 12.942544 | up |
| KANK4 | 9.10E-08 | 12.943934 | up |
| ARID3B | 2.80E-09 | 12.961038 | up |
| KIAA1549L | 8.75E-07 | 13.006303 | up |
| ASXL3 | 6.97E-07 | 13.025158 | up |
| ZNF718 | 9.56E-08 | 13.034648 | up |
| MYL9 | 6.98E-07 | 13.069727 | up |
| UTY | 5.66E-07 | 13.119806 | up |
| HOMER2 | 3.71E-09 | 13.196139 | up |
| TNFRSF11B | 8.26E-08 | 13.260222 | up |
| STAG3 | 9.46E-08 | 13.275085 | up |
| PSG3 | 4.56E-07 | 13.28377 | up |
| MICAL2 | 9.68E-07 | 13.33032 | up |
| TCF21 | 9.51E-08 | 13.391823 | up |
| ZNF433 | 4.62E-07 | 13.480659 | up |
| MARCKSL1 | 4.54E-08 | 13.495071 | up |
| LOC645427 | 7.52E-07 | 13.503076 | up |
| PPP2R2B | 1.32E-06 | 13.524933 | up |
| CLDN10 | 5.62E-08 | 13.565895 | up |
| NLGN4Y | 5.12E-07 | 13.594249 | up |
| LPL | 1.12E-07 | 13.624017 | up |
| BHMT2 | 2.31E-07 | 13.629919 | up |
| SULT1C4 | 8.79E-08 | 13.716656 | up |
| GPR115 | 8.43E-07 | 13.761036 | up |
| PELI2 | 1.03E-07 | 13.768128 | up |
| SFMBT2 | 1.88E-07 | 13.848661 | up |
| KDM5D | 3.74E-08 | 13.879221 | up |
| ESPNL | 7.18E-08 | 14.066545 | up |
| INF2 | 3.44E-08 | 14.174415 | up |
| POPDC3 | 6.22E-09 | 14.177601 | up |
| IL4I1 | 1.27E-09 | 14.29791 | up |
| LINC00862 | 7.54E-07 | 14.316255 | up |
| NAP1L2 | 7.82E-07 | 14.402844 | up |
| SNAR-A3 | 3.02E-08 | 14.492891 | up |
| ADAM18 | 1.67E-07 | 14.492985 | up |
| LRRC4 | 7.31E-07 | 14.495536 | up |
| KHDRBS3 | 4.95E-08 | 14.495934 | up |
| PHLDA2 | 1.68E-09 | 14.516325 | up |
| DUSP26 | 1.22E-08 | 14.552825 | up |
| RNF183 | 4.56E-07 | 14.606747 | up |
| FADS2 | 7.63E-10 | 14.66791 | up |
| MT1G | 3.99E-09 | 14.684936 | up |
| PDZRN3 | 7.89E-08 | 14.722113 | up |
| SLCO2A1 | 5.91E-08 | 14.756784 | up |
| ZNF681 | 1.12E-07 | 14.766514 | up |
| CDKN2A | 3.34E-09 | 15.013479 | up |
| KCNJ12 | 1.28E-08 | 15.059012 | up |
| CABP7 | 7.05E-07 | 15.154467 | up |
| KCNJ6 | 2.36E-07 | 15.203087 | up |
| PALM2 | 2.88E-08 | 15.223929 | up |
| PDLIM7 | 1.69E-08 | 15.318217 | up |
| LOXL3 | 2.78E-08 | 15.326272 | up |
| IGSF22 | 3.09E-08 | 15.406097 | up |
| ZNF577 | 1.22E-07 | 15.441486 | up |
| ZNF532 | 2.57E-07 | 15.453401 | up |
| GPA33 | 8.54E-08 | 15.496196 | up |
| UPK1B | 8.21E-08 | 15.53269 | up |
| EDNRA | 1.62E-07 | 15.58192 | up |
| CRLF1 | 9.19E-09 | 15.616175 | up |
| TEX12 | 3.38E-08 | 15.842311 | up |
| NKAIN3 | 1.06E-07 | 15.904137 | up |
| ESRP2 | 1.57E-09 | 15.923325 | up |
| ID1 | 1.95E-09 | 16.052015 | up |
| CARD6 | 1.88E-07 | 16.065285 | up |
| PDLIM7 | 1.58E-08 | 16.077126 | up |
| LOC100128242 | 3.15E-07 | 16.144747 | up |
| TSPAN7 | 7.58E-08 | 16.181479 | up |
| A1BG | 4.88E-08 | 16.190086 | up |
| MYH16 | 2.62E-07 | 16.204231 | up |
| PCDH7 | 3.70E-09 | 16.22289 | up |
| CSMD2 | 3.91E-08 | 16.23314 | up |
| SLC51B | 6.12E-07 | 16.312796 | up |
| LBH | 5.02E-09 | 16.45038 | up |
|  | 1.20E-07 | 16.531757 | up |
| BCL11A | 8.88E-08 | 16.588211 | up |
| HRASLS | 2.63E-09 | 16.590563 | up |
| LOC100130587 | 3.82E-07 | 16.63008 | up |
| CD200 | 2.88E-08 | 16.637217 | up |
| POMC | 8.94E-09 | 16.656975 | up |
| CDA | 5.21E-10 | 16.667944 | up |
| SMIM10 | 6.62E-08 | 16.675587 | up |
| PPP1R1A | 1.07E-06 | 16.76363 | up |
| CCRL2 | 5.39E-08 | 16.79864 | up |
| ZNF711 | 1.03E-07 | 16.93634 | up |
| COL25A1 | 2.65E-08 | 16.972782 | up |
| HS3ST2 | 1.82E-07 | 16.983889 | up |
| LARGE | 3.16E-10 | 17.090157 | up |
| MARK1 | 6.97E-08 | 17.098915 | up |
| RNF152 | 6.73E-08 | 17.140293 | up |
| LOXL1 | 4.05E-10 | 17.231085 | up |
| VSNL1 | 5.56E-08 | 17.236246 | up |
| CCDC81 | 9.44E-08 | 17.241636 | up |
| ZNF257 | 3.46E-08 | 17.306107 | up |
| LSAMP | 1.33E-07 | 17.34864 | up |
| DOK6 | 6.57E-08 | 17.421558 | up |
| PTPRK | 9.29E-08 | 17.542784 | up |
| TNC | 4.57E-08 | 17.616402 | up |
| CECR1 | 4.95E-07 | 17.627094 | up |
| KCNMB4 | 2.32E-08 | 17.636768 | up |
| HENMT1 | 9.31E-09 | 17.783064 | up |
| IGF2BP1 | 3.04E-09 | 17.788078 | up |
| C1orf159 | 2.12E-07 | 17.823223 | up |
| BGN | 7.17E-09 | 17.859194 | up |
| ZMAT4 | 6.61E-08 | 17.86537 | up |
| KIT | 2.12E-08 | 17.94305 | up |
| LY96 | 5.93E-09 | 18.001543 | up |
| DPYSL3 | 5.80E-10 | 18.040009 | up |
| IL17RD | 1.61E-09 | 18.066162 | up |
| GPM6B | 1.47E-07 | 18.07815 | up |
| CD274 | 2.68E-09 | 18.096653 | up |
| IFI27 | 1.12E-08 | 18.18886 | up |
| LIPG | 2.02E-08 | 18.461082 | up |
| ZNF563 | 7.72E-08 | 18.461826 | up |
| HCG4 | 1.21E-07 | 18.510975 | up |
| ROBO4 | 1.06E-06 | 18.559183 | up |
| TPM2 | 1.51E-09 | 18.563482 | up |
| EFEMP1 | 8.33E-10 | 18.66918 | up |
| FN1 | 6.08E-08 | 18.746315 | up |
| TPM2 | 5.61E-10 | 18.752396 | up |
| EPHX4 | 8.78E-09 | 18.764032 | up |
| LAMP5 | 6.92E-07 | 18.824368 | up |
| LOXL2 | 1.74E-09 | 18.871601 | up |
| LRFN5 | 8.38E-07 | 19.025917 | up |
| PADI2 | 7.23E-09 | 19.077356 | up |
| LOC286068 | 8.96E-08 | 19.105469 | up |
| COL6A3 | 6.46E-07 | 19.158003 | up |
| L1TD1 | 3.70E-08 | 19.203737 | up |
| ZNF383 | 7.85E-08 | 19.229836 | up |
| THBS2 | 7.37E-08 | 19.264875 | up |
| CFTR | 2.19E-07 | 19.353477 | up |
| VSTM4 | 8.74E-07 | 19.41728 | up |
| LOC729860 | 1.00E-07 | 19.439133 | up |
| SERPINE1 | 2.25E-08 | 19.459131 | up |
| AHRR | 2.58E-07 | 19.481693 | up |
| C4orf51 | 3.32E-07 | 19.512327 | up |
| LRP3 | 6.36E-07 | 19.527231 | up |
| DCHS1 | 3.24E-08 | 19.554558 | up |
| lnc-FAM133B-1 | 1.13E-07 | 19.622843 | up |
| PLEKHO1 | 4.74E-09 | 19.622921 | up |
| LOC441666 | 1.37E-07 | 19.655916 | up |
| HPGD | 5.72E-08 | 19.685608 | up |
| CXCL12 | 2.03E-09 | 19.743992 | up |
| SGCD | 1.34E-07 | 19.839184 | up |
| JAG1 | 9.61E-09 | 20.109228 | up |
| BACH2 | 4.86E-08 | 20.155428 | up |
| SNORA23 | 3.01E-08 | 20.234097 | up |
| LOC100131826 | 8.54E-08 | 20.352453 | up |
| SSTR2 | 8.73E-08 | 20.429796 | up |
| NTNG1 | 2.66E-07 | 20.622387 | up |
| REP15 | 2.16E-07 | 20.65612 | up |
| FAM101B | 3.90E-09 | 20.820784 | up |
| FOXS1 | 7.82E-10 | 20.83907 | up |
| ZNF69 | 7.72E-08 | 21.091755 | up |
| KAL1 | 4.00E-08 | 21.229038 | up |
| PLEKHO1 | 8.69E-10 | 21.34836 | up |
| EGLN3 | 1.56E-07 | 21.395447 | up |
| ZNF573 | 4.25E-08 | 21.417131 | up |
| RAP1GAP | 2.33E-07 | 21.435602 | up |
| NUDT10 | 9.16E-08 | 21.441837 | up |
| ZNF662 | 8.26E-07 | 21.600765 | up |
| CYP27C1 | 3.82E-08 | 21.602964 | up |
| PCDHB2 | 1.53E-07 | 21.65118 | up |
| TMEM108 | 8.82E-07 | 21.818121 | up |
| TNFRSF10C | 2.15E-08 | 21.841145 | up |
| NCAM1 | 1.59E-07 | 22.06441 | up |
| PTGS1 | 8.47E-08 | 22.275583 | up |
| ZNF808 | 6.82E-09 | 22.359348 | up |
| TSPYL5 | 5.55E-10 | 22.362585 | up |
| DACT1 | 9.07E-09 | 22.44472 | up |
| STMN2 | 7.32E-08 | 22.458513 | up |
| SNORA28 | 3.24E-07 | 22.5451 | up |
| SLC2A9 | 2.03E-08 | 22.613241 | up |
| LOC100130938 | 4.32E-08 | 22.758299 | up |
| CHST1 | 1.85E-08 | 22.778196 | up |
| ITGB3 | 4.86E-08 | 22.84977 | up |
| CPXM2 | 1.29E-08 | 22.97028 | up |
| DKK1 | 1.83E-09 | 23.126623 | up |
| SPANXC | 1.49E-07 | 23.194675 | up |
| ZNF439 | 1.08E-07 | 23.200642 | up |
| BMF | 6.42E-10 | 23.254866 | up |
| SCG2 | 7.08E-08 | 23.474545 | up |
| RPL27A | 1.62E-07 | 23.603521 | up |
| LPAR5 | 2.87E-07 | 23.794338 | up |
| BMP7 | 6.58E-07 | 23.845694 | up |
| COL13A1 | 1.24E-09 | 23.86528 | up |
| DKFZP586B0319 | 8.74E-10 | 23.896221 | up |
| CRMP1 | 7.03E-08 | 24.07141 | up |
| MFGE8 | 2.75E-10 | 24.091402 | up |
| CDK15 | 3.54E-08 | 24.10429 | up |
| PSG2 | 4.64E-08 | 24.289783 | up |
| F2RL2 | 2.04E-09 | 24.43335 | up |
| ZNF135 | 1.91E-08 | 24.458776 | up |
| AANAT | 3.81E-07 | 24.818192 | up |
| DNM3 | 8.73E-08 | 24.971762 | up |
| MDFI | 1.30E-07 | 25.024277 | up |
| UGT1A6 | 2.18E-08 | 25.227497 | up |
| UNC13C | 3.92E-08 | 25.26549 | up |
| BMP7 | 2.15E-08 | 25.478222 | up |
| SPANXN3 | 7.01E-08 | 25.572601 | up |
| BST1 | 2.30E-08 | 25.619673 | up |
| PDLIM7 | 5.35E-10 | 25.718836 | up |
| ZNF880 | 7.04E-08 | 25.720282 | up |
| KCNA5 | 3.96E-07 | 25.828735 | up |
| CCDC3 | 5.74E-07 | 26.139332 | up |
| ABCA1 | 7.78E-09 | 26.19639 | up |
| F2R | 2.13E-08 | 26.307043 | up |
| LOC441666 | 3.18E-08 | 26.439844 | up |
| UGT1A6 | 5.78E-10 | 26.440737 | up |
| ZNF334 | 3.91E-08 | 26.507439 | up |
| MIR100HG | 2.09E-08 | 26.781822 | up |
| ANXA10 | 5.18E-08 | 26.858366 | up |
| LOC101929056 | 6.43E-08 | 27.235672 | up |
| PCSK5 | 6.76E-08 | 27.312061 | up |
| MYADM | 1.79E-09 | 27.473135 | up |
| C11orf96 | 7.76E-10 | 27.480192 | up |
| LPHN3 | 1.43E-06 | 27.534624 | up |
| BAMBI | 4.88E-10 | 27.681984 | up |
| CCT3 | 3.42E-07 | 27.847366 | up |
| ST3GAL1 | 6.25E-10 | 28.029547 | up |
| RHOJ | 2.05E-08 | 28.106472 | up |
| LINC01336 | 1.54E-08 | 28.164677 | up |
| LINC00312 | 3.64E-09 | 28.176231 | up |
| RASIP1 | 3.07E-09 | 28.244978 | up |
| ZNF470 | 3.05E-08 | 28.35821 | up |
| CENPVP2 | 7.67E-08 | 28.416523 | up |
| UGT1A8 | 1.69E-08 | 28.48048 | up |
| WDR33 | 1.13E-06 | 28.751709 | up |
| SLC16A14 | 9.99E-07 | 29.383163 | up |
| TNF | 8.86E-09 | 29.414822 | up |
| BCAR1 | 1.66E-07 | 29.536005 | up |
| CD69 | 5.01E-08 | 29.589128 | up |
| VPS18 | 1.72E-07 | 29.931538 | up |
| HS6ST2 | 1.33E-09 | 29.971958 | up |
| BNC1 | 8.00E-07 | 30.202827 | up |
| ZNF204P | 3.29E-08 | 30.292244 | up |
| CD200 | 4.04E-07 | 30.71527 | up |
| ZNF682 | 2.29E-08 | 30.72005 | up |
| MMP10 | 5.30E-08 | 30.762878 | up |
| GABBR2 | 1.45E-09 | 31.00523 | up |
| CHI3L1 | 3.99E-07 | 31.077652 | up |
| SLC37A2 | 1.70E-08 | 31.219748 | up |
| PRSS3 | 4.01E-08 | 31.38987 | up |
| TRHDE | 2.12E-08 | 31.407616 | up |
| MMP9 | 1.40E-09 | 31.546629 | up |
| LOC101928880 | 2.45E-08 | 31.55977 | up |
| FLRT2 | 3.08E-08 | 31.775637 | up |
| CLDN2 | 2.17E-09 | 31.832525 | up |
| SLCO2B1 | 7.50E-08 | 32.700535 | up |
| CSF1R | 2.74E-08 | 32.915478 | up |
| MYOZ3 | 5.96E-10 | 32.92045 | up |
| DCN | 5.84E-08 | 33.03018 | up |
| DGKI | 2.94E-09 | 33.42763 | up |
| TDRD9 | 3.34E-08 | 33.54546 | up |
| CPT1C | 3.49E-08 | 33.70914 | up |
| TLDC2 | 4.02E-08 | 33.74458 | up |
| TMEM132D | 2.49E-08 | 33.926586 | up |
| IRX1 | 8.08E-09 | 33.932484 | up |
| SFN | 4.23E-09 | 34.030006 | up |
| FAM83B | 5.93E-07 | 34.04424 | up |
| NEDD9 | 1.33E-08 | 34.438557 | up |
| LOC151760 | 1.07E-08 | 34.439705 | up |
| NLGN4X | 8.24E-07 | 34.807034 | up |
| ADAM19 | 2.09E-10 | 34.863346 | up |
| TSPAN13 | 9.25E-10 | 34.960365 | up |
| FAM50B | 9.19E-10 | 35.274647 | up |
| UTY | 1.72E-08 | 35.43921 | up |
| ZNF879 | 7.60E-07 | 35.900112 | up |
| LAMA4 | 3.77E-08 | 36.36339 | up |
| MT1H | 9.19E-09 | 37.1173 | up |
| EPS8L1 | 2.75E-09 | 37.188988 | up |
| FAM174B | 4.40E-09 | 37.235714 | up |
| CENPV | 2.12E-08 | 37.274563 | up |
| LOC100128398 | 4.94E-08 | 37.302883 | up |
| RAMP1 | 5.04E-10 | 37.44207 | up |
| ICAM1 | 5.54E-10 | 37.483227 | up |
| FZD8 | 3.70E-08 | 37.54654 | up |
| RASGRF2 | 2.87E-08 | 37.577763 | up |
| SLC24A3 | 2.43E-08 | 37.855267 | up |
| LOC100996405 | 9.46E-07 | 37.920734 | up |
| ITM2A | 2.56E-08 | 38.006687 | up |
| ACTA1 | 4.00E-09 | 38.038757 | up |
| WNT5A | 6.47E-10 | 38.10628 | up |
| ALDH1A2 | 1.41E-08 | 38.223328 | up |
| NTNG1 | 2.88E-08 | 38.260147 | up |
| MTSS1 | 1.58E-08 | 38.302433 | up |
| SFRP1 | 5.07E-09 | 38.377098 | up |
| LTBP1 | 7.90E-09 | 38.56313 | up |
| SOX9 | 2.13E-08 | 39.06005 | up |
| MFAP2 | 9.92E-10 | 39.52456 | up |
| LRRN1 | 2.79E-08 | 39.578262 | up |
| F2R | 1.15E-09 | 39.771263 | up |
| FLI1 | 1.48E-08 | 40.31549 | up |
| FZD10 | 1.63E-08 | 40.665882 | up |
| MEDAG | 8.60E-09 | 41.08363 | up |
| NEDD9 | 8.87E-08 | 41.465084 | up |
| ZNF610 | 2.27E-08 | 41.494778 | up |
| EVC2 | 2.18E-08 | 41.6845 | up |
| LOC729162 | 2.07E-07 | 42.20242 | up |
| EFNB2 | 2.67E-10 | 42.547462 | up |
| CA12 | 2.03E-07 | 42.672314 | up |
| ZNF347 | 1.41E-08 | 42.991432 | up |
| ZNF606 | 9.50E-09 | 43.00658 | up |
| A1BG-AS1 | 6.24E-09 | 44.14876 | up |
| NR2F1 | 3.18E-08 | 44.534527 | up |
| DMRT2 | 8.19E-07 | 44.56221 | up |
| NEFH | 1.59E-08 | 44.59452 | up |
| ZCCHC13 | 3.23E-07 | 45.416588 | up |
| MLPH | 5.55E-09 | 45.443123 | up |
| EFNB1 | 5.71E-10 | 45.536415 | up |
| CNTNAP2 | 8.14E-09 | 45.61191 | up |
| ZNF544 | 2.25E-07 | 45.671272 | up |
| MAF | 2.04E-09 | 45.752464 | up |
| SPOCD1 | 6.65E-11 | 45.80005 | up |
| NTNG1 | 4.30E-08 | 45.86496 | up |
| C6orf141 | 1.10E-08 | 45.928963 | up |
| ZIK1 | 1.16E-08 | 46.02967 | up |
| ZFP82 | 4.59E-09 | 46.324326 | up |
| ACTG2 | 2.88E-09 | 46.391804 | up |
| VGLL3 | 1.68E-07 | 46.619602 | up |
| COL22A1 | 7.55E-09 | 47.208824 | up |
| SULT1C4 | 4.02E-09 | 47.278122 | up |
| GDNF | 3.27E-07 | 47.307274 | up |
| PRSS3P2 | 1.21E-09 | 47.627766 | up |
| TMEM173 | 1.55E-09 | 47.630585 | up |
| SPEN | 3.58E-07 | 47.776318 | up |
| ZYG11A | 1.77E-07 | 47.867928 | up |
| NRG3 | 2.10E-08 | 47.98559 | up |
| TRHDE-AS1 | 5.82E-09 | 48.283096 | up |
| LPCAT2 | 1.37E-08 | 48.63072 | up |
| LOC100129397 | 3.43E-08 | 48.6476 | up |
| XDH | 4.14E-09 | 48.864986 | up |
| ZNF469 | 1.79E-10 | 49.149094 | up |
| MGC12916 | 5.05E-08 | 49.210278 | up |
| GRIA3 | 1.04E-08 | 50.015465 | up |
| MMP2 | 1.00E-08 | 50.032032 | up |
| NLGN4Y | 5.64E-09 | 50.03337 | up |
| PTPLAD2 | 3.34E-09 | 50.253284 | up |
| UCP2 | 9.49E-09 | 50.8998 | up |
| HYI | 8.19E-11 | 51.98913 | up |
| CYP27C1 | 1.75E-08 | 51.99148 | up |
| LPCAT2 | 1.81E-08 | 52.28421 | up |
| ZNF667-AS1 | 1.57E-07 | 53.033962 | up |
| TLL1 | 5.83E-09 | 53.44993 | up |
| ANKRD1 | 5.77E-10 | 53.75897 | up |
| ZNF793 | 5.68E-09 | 54.17079 | up |
| ARHGDIB | 1.85E-10 | 54.20742 | up |
| HYI | 7.61E-11 | 54.300846 | up |
| PTPRB | 1.24E-07 | 54.54932 | up |
| SCOC-AS1 | 2.50E-08 | 55.94328 | up |
| THBD | 2.27E-10 | 56.06432 | up |
| ZNF415 | 1.60E-08 | 56.0696 | up |
| KRT34 | 1.24E-07 | 56.94978 | up |
| MGP | 3.94E-08 | 57.118397 | up |
| MRGPRF | 1.06E-08 | 57.331955 | up |
| C4orf26 | 1.71E-09 | 57.53583 | up |
| EDN1 | 1.65E-08 | 58.12707 | up |
| CYTIP | 1.12E-08 | 58.14373 | up |
| F3 | 6.00E-09 | 58.17074 | up |
| lnc-EIF3M-2 | 1.35E-08 | 58.754013 | up |
| MYOM2 | 8.52E-09 | 58.842243 | up |
| WT1 | 1.26E-10 | 58.93228 | up |
| AFP | 9.70E-09 | 59.258663 | up |
| SLC7A8 | 2.02E-08 | 59.32645 | up |
| NOTCH3 | 5.04E-10 | 59.534092 | up |
| GDNF | 6.96E-09 | 59.851482 | up |
| CTHRC1 | 1.74E-08 | 60.139744 | up |
| PPAPDC3 | 3.13E-07 | 61.13508 | up |
| MGC20647 | 5.08E-09 | 61.247486 | up |
| PPAPDC1A | 9.79E-10 | 61.49583 | up |
| ARPC5 | 5.81E-07 | 63.242287 | up |
| PSG8 | 3.84E-09 | 63.300423 | up |
| LOC152225 | 5.12E-09 | 64.322334 | up |
| FBLN2 | 1.27E-10 | 64.40507 | up |
| HBEGF | 1.02E-10 | 64.87882 | up |
| MEIOB | 3.14E-08 | 65.55756 | up |
| BMP2 | 1.12E-09 | 65.96892 | up |
| PQLC2L | 7.16E-08 | 66.52395 | up |
| DYSF | 1.43E-08 | 66.72023 | up |
| GPR87 | 2.05E-08 | 66.761215 | up |
| LTB | 8.01E-09 | 67.27282 | up |
| DIRC3 | 3.49E-09 | 67.548676 | up |
| NOX4 | 4.01E-10 | 67.9212 | up |
| IRF6 | 6.47E-09 | 69.44795 | up |
| MSX2P1 | 5.43E-10 | 69.44816 | up |
| ZNF876P | 6.68E-09 | 69.74836 | up |
| LOC100127909 | 1.28E-08 | 70.01976 | up |
| UBD | 1.01E-07 | 70.12875 | up |
| ANKRD35 | 1.77E-07 | 70.241234 | up |
| TMEM233 | 4.91E-09 | 70.32545 | up |
| FOXL1 | 2.54E-10 | 70.41304 | up |
| LRRC34 | 1.90E-07 | 70.41674 | up |
| NFATC4 | 5.04E-09 | 70.840195 | up |
| RSPO4 | 1.94E-08 | 71.07355 | up |
| ZNF441 | 6.17E-09 | 71.48203 | up |
| FLNA | 2.30E-09 | 71.830894 | up |
| MAOB | 2.44E-09 | 72.05074 | up |
| XAGE-4 | 5.64E-07 | 72.13972 | up |
| ATXN3L | 1.68E-07 | 72.488144 | up |
| FNDC1 | 2.38E-10 | 73.0385 | up |
| MME | 8.20E-09 | 73.90935 | up |
| PPP6R1 | 8.03E-07 | 74.511925 | up |
| CLIC3 | 1.12E-08 | 74.71523 | up |
| KRBOX1 | 3.69E-09 | 74.98335 | up |
| ZNF730 | 5.03E-09 | 75.2125 | up |
| ZNF20 | 7.38E-09 | 76.64473 | up |
| TXLNGY | 5.71E-09 | 77.74296 | up |
| SCEL | 3.09E-09 | 78.45914 | up |
| VPS9D1-AS1 | 9.71E-07 | 79.75225 | up |
| SYK | 7.88E-11 | 82.00911 | up |
| FGF13 | 4.11E-09 | 84.200294 | up |
| DCN | 1.23E-08 | 85.463295 | up |
| FOXC2 | 7.23E-09 | 86.298065 | up |
| CDO1 | 3.23E-09 | 86.31352 | up |
| SGCD | 6.77E-09 | 86.3672 | up |
| PCSK5 | 4.11E-09 | 86.415375 | up |
| FAM26F | 1.80E-09 | 86.66302 | up |
| LOC283731 | 1.18E-06 | 86.73792 | up |
| ALDOC | 1.15E-07 | 86.773994 | up |
| SLC2A5 | 2.17E-07 | 87.31256 | up |
| THBS2 | 1.35E-08 | 88.86851 | up |
| KCNJ15 | 4.14E-09 | 89.727646 | up |
| SLC16A3 | 4.24E-10 | 89.79431 | up |
| TIMP3 | 3.02E-07 | 90.46507 | up |
| EDN1 | 1.67E-09 | 91.24608 | up |
| LAD1 | 1.64E-10 | 91.49767 | up |
| PHACTR1 | 1.37E-08 | 91.5268 | up |
| ZNF256 | 6.56E-07 | 91.55802 | up |
| RCAN2 | 9.12E-10 | 92.26575 | up |
| CHST2 | 8.07E-09 | 92.3112 | up |
| MLLT3 | 3.46E-09 | 95.706024 | up |
| POSTN | 7.50E-09 | 97.10501 | up |
| ZFY | 5.99E-09 | 97.26964 | up |
| ID4 | 7.85E-07 | 97.395546 | up |
| TTTY14 | 3.54E-09 | 97.79492 | up |
| TMSB15A | 5.86E-11 | 98.392944 | up |
| FAM101A | 1.06E-08 | 98.917885 | up |
| COL6A3 | 4.60E-09 | 100.14288 | up |
| CDH13 | 1.33E-10 | 100.55534 | up |
| SELENBP1 | 3.40E-09 | 100.79079 | up |
| NAP1L5 | 5.93E-09 | 101.09954 | up |
| ZNF880 | 5.16E-07 | 101.71925 | up |
| SEMA7A | 2.40E-08 | 101.79193 | up |
| TOX | 1.89E-09 | 102.97063 | up |
| ELTD1 | 1.87E-09 | 103.45271 | up |
| ZNF426 | 2.15E-09 | 103.74901 | up |
| IL7R | 1.22E-08 | 106.81078 | up |
| EIF1AY | 2.30E-09 | 107.98555 | up |
| CARD6 | 6.18E-09 | 108.52279 | up |
| ZNF649 | 2.93E-09 | 109.15837 | up |
| ZNF816 | 2.34E-09 | 109.16241 | up |
| ITGA11 | 1.26E-09 | 111.90539 | up |
| LOC100240735 | 2.19E-07 | 113.07271 | up |
| NR2F1-AS1 | 6.28E-09 | 113.64907 | up |
| KCNS1 | 3.25E-09 | 114.89958 | up |
| MUM1L1 | 4.44E-09 | 116.38032 | up |
| HHIP | 1.01E-09 | 116.93157 | up |
| CPA4 | 1.65E-08 | 117.13786 | up |
| NIN | 2.88E-09 | 118.87657 | up |
| MIR205HG | 3.85E-09 | 119.98412 | up |
| STRA6 | 1.44E-08 | 121.20266 | up |
| lnc-SNX24-1 | 5.13E-07 | 123.29643 | up |
| PKIB | 3.13E-08 | 125.37759 | up |
| GNAS-AS1 | 2.49E-09 | 127.1275 | up |
| ZFP42 | 3.05E-09 | 128.12143 | up |
| NRG3 | 2.38E-09 | 131.40103 | up |
| CELF2 | 1.97E-09 | 141.05788 | up |
| LIMS3L | 5.75E-09 | 141.82043 | up |
| RBP1 | 7.90E-11 | 143.20576 | up |
| RAMP1 | 1.84E-09 | 143.26295 | up |
| KALRN | 2.26E-07 | 145.83266 | up |
| USP9Y | 1.74E-09 | 146.37286 | up |
| COL5A1 | 7.82E-11 | 152.43863 | up |
| SSTR1 | 2.50E-09 | 153.59613 | up |
| ST6GAL2 | 3.93E-09 | 157.3001 | up |
| PPP1R14A | 2.80E-09 | 161.00612 | up |
| COL1A2 | 5.74E-10 | 161.4505 | up |
| LUM | 1.35E-09 | 161.62215 | up |
| SORCS3 | 5.70E-11 | 162.14645 | up |
| CD86 | 4.24E-07 | 164.99081 | up |
| COL5A1 | 1.68E-09 | 174.96568 | up |
| DIRAS2 | 2.64E-09 | 178.32709 | up |
| SLC17A9 | 6.46E-09 | 179.46259 | up |
| MIR31HG | 2.46E-09 | 179.93239 | up |
| ACP5 | 3.23E-08 | 181.03477 | up |
| CELF2 | 4.80E-10 | 181.43587 | up |
| CLEC18C | 6.22E-08 | 183.97987 | up |
| GAS1 | 4.87E-09 | 184.00092 | up |
| GPR179 | 4.62E-07 | 185.90695 | up |
| TRHDE-AS1 | 1.09E-06 | 186.16145 | up |
| MLLT3 | 1.74E-09 | 186.9926 | up |
| RGS4 | 1.14E-08 | 189.54163 | up |
| SLN | 3.92E-07 | 192.99277 | up |
| DZIP1 | 1.27E-08 | 194.88834 | up |
| ANKRD30BP2 | 6.60E-07 | 201.14366 | up |
| LZTS1 | 9.61E-08 | 202.2315 | up |
| IRX4 | 3.27E-11 | 205.81421 | up |
| CLDN6 | 1.80E-08 | 205.93063 | up |
| PROM1 | 6.47E-10 | 206.87164 | up |
| EPPK1 | 1.08E-08 | 206.9886 | up |
| KRT79 | 1.37E-06 | 207.60243 | up |
| CAND2 | 1.38E-09 | 209.88625 | up |
| HYMAI | 1.55E-09 | 211.1145 | up |
| MGP | 3.39E-09 | 212.20514 | up |
| ZNF569 | 3.11E-09 | 214.63405 | up |
| SYT13 | 7.29E-08 | 217.65173 | up |
| SST | 7.64E-08 | 217.76277 | up |
| ZNF542P | 1.25E-09 | 219.02779 | up |
| PDLIM3 | 6.44E-09 | 220.75854 | up |
| ZNF667-AS1 | 3.89E-09 | 223.61188 | up |
| SOX17 | 1.41E-09 | 225.0726 | up |
| MTAP | 2.12E-09 | 228.7785 | up |
| DMRT3 | 8.22E-10 | 237.76941 | up |
| CYTL1 | 5.02E-09 | 238.65263 | up |
| NEFM | 8.82E-10 | 239.6413 | up |
| DNAJC15 | 3.87E-07 | 240.47345 | up |
| HMBOX1 | 1.52E-07 | 252.04044 | up |
| LRRC17 | 4.13E-07 | 265.39566 | up |
| KCNJ15 | 1.11E-09 | 274.61285 | up |
| CYTL1 | 1.45E-09 | 304.39978 | up |
| MTAP | 1.09E-09 | 317.06226 | up |
| SLC7A7 | 1.19E-09 | 317.3231 | up |
| TAGLN | 1.91E-11 | 325.5919 | up |
| GREM1 | 2.97E-08 | 339.11642 | up |
| NOX4 | 9.64E-10 | 346.67224 | up |
| PTN | 7.52E-11 | 347.07166 | up |
| MTSS1 | 6.20E-10 | 348.25293 | up |
| CLDN4 | 2.94E-09 | 354.12265 | up |
| ZNF671 | 1.57E-09 | 357.13074 | up |
| EMX2OS | 3.74E-07 | 361.1416 | up |
| CTSF | 3.01E-07 | 365.63016 | up |
| APCDD1L | 8.10E-10 | 379.23993 | up |
| TAGLN | 3.53E-11 | 382.85748 | up |
| LRRC17 | 1.49E-11 | 386.16226 | up |
| KRTAP2-3 | 6.12E-09 | 391.0088 | up |
| MMP1 | 2.15E-09 | 435.6564 | up |
| MALL | 3.78E-10 | 437.84857 | up |
| MXRA8 | 1.14E-09 | 439.5212 | up |
| TRIM58 | 2.06E-08 | 441.65897 | up |
| FGF1 | 2.04E-09 | 442.38068 | up |
| GREM1 | 1.48E-09 | 482.3497 | up |
| ZNF518B | 7.78E-10 | 490.25983 | up |
| SPON2 | 1.33E-10 | 490.4718 | up |
| FUOM | 7.28E-09 | 512.7451 | up |
| PRKCDBP | 8.90E-11 | 516.87714 | up |
| GSTT1 | 5.98E-11 | 518.86163 | up |
| IGFBP2 | 4.20E-11 | 518.994 | up |
| CLEC18B | 3.52E-10 | 540.80414 | up |
| PNOC | 5.65E-08 | 555.95966 | up |
| DDX3Y | 3.49E-10 | 589.87506 | up |
| CNN1 | 1.24E-09 | 590.5972 | up |
| MTAP | 5.48E-10 | 612.52057 | up |
| SRGN | 1.36E-10 | 622.7358 | up |
| PDPN | 5.84E-10 | 656.44574 | up |
| NPPB | 2.55E-09 | 658.9935 | up |
| HIST1H1B | 5.86E-09 | 659.9072 | up |
| BMP7 | 5.61E-09 | 685.6174 | up |
| NPPB | 2.09E-10 | 685.7674 | up |
| EMX2 | 5.18E-10 | 718.6404 | up |
| ZSCAN18 | 1.22E-07 | 735.7765 | up |
| TACSTD2 | 3.67E-10 | 736.73285 | up |
| SPINT2 | 4.70E-09 | 906.4438 | up |
| KIF1A | 8.29E-09 | 966.8521 | up |
| COL1A1 | 1.61E-10 | 1013.8806 | up |
| CDH6 | 2.28E-07 | 1073.3291 | up |
| BTBD11 | 2.80E-10 | 1204.9498 | up |
| AEBP1 | 5.11E-10 | 1218.3971 | up |
| NDN | 1.42E-10 | 1303.5846 | up |
| DPYSL4 | 3.36E-11 | 1315.4003 | up |
| SOX11 | 1.86E-10 | 1414.277 | up |
| IL11 | 9.50E-07 | 1536.8881 | up |
| LCP1 | 7.00E-08 | 1549.8038 | up |
| KLHL9 | 5.02E-08 | 1737.7172 | up |
| TENM2 | 5.81E-09 | 2037.7706 | up |
| CDH11 | 3.93E-07 | 2039.2178 | up |
| HSPA1A | 5.58E-10 | 2071.5332 | up |
| ALPK3 | 1.60E-09 | 2217.4985 | up |
| FOCAD | 3.18E-10 | 2461.82 | up |
| IGFBP5 | 1.03E-10 | 4112.031 | up |
| UCHL1 | 2.48E-08 | 6779.7056 | up |
| RPS4Y2 | 1.46E-10 | 9231.681 | up |
| MYADM | 9.93E-11 | 9740.568 | up |
| RPS4Y1 | 1.28E-10 | 10168.842 | up |
