# Supplementary material for: Comparative Gene Expression Profiling of Primary and Metastatic Renal Cell Carcinoma Stem Cell-Like Cancer Cells
Source: PLoS One. 2016 Nov 3;11(11):e0165718. doi: 10.1371/journal.pone.0165718 (PMC5094751; doi:10.1371/journal.pone.0165718)
Supplement: S2 Table — (DOCX) [file pone.0165718.s004.docx]

**Supplementary table S2:** List of up- and down-regulated genes in ASE-5063 (healthy kidney) vs CD105(Caki-2) cells.

| GeneSymbol | p | FC (abs) | Regulation |
| --- | --- | --- | --- |
| PITX1 | 5.80E-11 | 1259.4725 | down |
| AFAP1-AS1 | 3.52E-10 | 1256.9475 | down |
| SAA1 | 3.68E-11 | 1030.5543 | down |
| PLAC8 | 1.31E-07 | 747.05695 | down |
| HBE1 | 1.99E-07 | 527.0541 | down |
| TBX15 | 5.18E-10 | 479.57794 | down |
| SAA2 | 5.73E-10 | 443.8247 | down |
| CASP1 | 2.08E-10 | 410.08685 | down |
| TM4SF18 | 3.16E-07 | 385.1344 | down |
| GNG4 | 4.02E-11 | 370.8708 | down |
| BMP5 | 8.36E-08 | 355.5958 | down |
| GNG4 | 1.79E-10 | 286.19495 | down |
| CARD17 | 1.54E-08 | 281.20456 | down |
| ALDH1A1 | 2.84E-11 | 268.71854 | down |
| ANKRD20A12P | 7.14E-07 | 268.36456 | down |
| HOXB13 | 2.67E-10 | 237.4759 | down |
| HOXA13 | 4.08E-10 | 178.23175 | down |
| LOC344887 | 4.32E-10 | 169.86801 | down |
| HBG1 | 2.45E-09 | 140.01443 | down |
| G0S2 | 6.38E-11 | 131.20526 | down |
| EREG | 9.91E-10 | 119.66313 | down |
| MILR1 | 3.77E-10 | 118.4753 | down |
| LINC01296 | 5.13E-10 | 117.86273 | down |
| ACKR4 | 7.56E-10 | 114.31113 | down |
| C10orf99 | 8.43E-07 | 112.14973 | down |
| FGFBP1 | 3.06E-09 | 110.26179 | down |
| SAA2 | 7.61E-10 | 103.14898 | down |
| WISP2 | 1.70E-07 | 99.078156 | down |
| IFI27 | 7.03E-10 | 93.01528 | down |
| TFAP2A | 1.19E-09 | 89.26121 | down |
| C1S | 2.16E-08 | 87.05888 | down |
| PRDM13 | 4.13E-09 | 86.83892 | down |
| CYP24A1 | 6.80E-08 | 84.19067 | down |
| CLDN11 | 1.47E-09 | 83.11459 | down |
| SAA4 | 6.65E-11 | 82.1694 | down |
| SLFN11 | 2.88E-09 | 81.23137 | down |
| AKR1B10 | 1.47E-09 | 80.13687 | down |
| TRIML2 | 6.10E-07 | 76.68844 | down |
| JAKMIP2 | 1.36E-06 | 76.07435 | down |
| AKR1B15 | 1.37E-09 | 75.14042 | down |
| ISL1 | 4.73E-09 | 70.31738 | down |
| CCM2L | 1.57E-09 | 64.18205 | down |
| ANKRD20A12P | 1.48E-10 | 63.203983 | down |
| CA3 | 5.01E-09 | 61.56577 | down |
| RHBDL2 | 9.04E-07 | 61.418987 | down |
| PAX3 | 1.01E-09 | 58.90831 | down |
| SP5 | 1.13E-09 | 57.23676 | down |
| lnc-RP3-377D14,1,1-3 | 2.33E-09 | 55.049667 | down |
| IL20RB | 1.77E-09 | 54.34539 | down |
| LINC01296 | 7.67E-09 | 53.03382 | down |
| AKR1B10 | 2.81E-08 | 52.937275 | down |
| AKR1C3 | 5.88E-10 | 51.346424 | down |
| SPINK13 | 1.44E-08 | 51.228947 | down |
| SPX | 7.17E-09 | 50.30046 | down |
| RHBDL2 | 1.00E-09 | 49.803474 | down |
| NUPR1 | 2.74E-10 | 49.677166 | down |
| C4BPB | 2.03E-09 | 49.597992 | down |
| LOC101927345 | 2.93E-09 | 49.18205 | down |
| LOC283028 | 1.76E-07 | 48.906044 | down |
| HLF | 2.37E-09 | 47.786583 | down |
| CACNG6 | 2.01E-09 | 46.953854 | down |
| GDA | 5.52E-09 | 46.783947 | down |
| BHMT2 | 5.41E-09 | 45.731857 | down |
| SECTM1 | 3.86E-10 | 45.566254 | down |
| lnc-DAOA-4 | 5.66E-08 | 43.797913 | down |
| OR51I1 | 4.61E-09 | 42.972904 | down |
| IL1A | 1.76E-07 | 42.13861 | down |
| IL18 | 1.97E-09 | 40.215225 | down |
| BLID | 1.40E-08 | 39.71517 | down |
| WFDC21P | 2.31E-09 | 39.593243 | down |
| DKK1 | 1.03E-09 | 39.250668 | down |
| CYP24A1 | 3.88E-07 | 39.085285 | down |
| MSR1 | 1.76E-09 | 38.73383 | down |
| LINC00704 | 6.57E-07 | 38.37584 | down |
| MYPN | 9.58E-10 | 37.8502 | down |
| FOXG1 | 2.69E-07 | 37.495483 | down |
| SBSPON | 7.16E-10 | 35.62872 | down |
| AKR1C4 | 2.72E-09 | 35.627872 | down |
| CHI3L2 | 1.54E-07 | 35.248783 | down |
| DLX1 | 1.96E-08 | 35.022217 | down |
| CCDC73 | 4.12E-09 | 34.972454 | down |
| TXK | 9.33E-08 | 34.514263 | down |
| PPFIBP2 | 3.89E-07 | 31.48052 | down |
| TFPI | 2.75E-09 | 30.63894 | down |
| HLA-DPA1 | 2.71E-10 | 30.001682 | down |
| ANKRD20A12P | 4.46E-08 | 29.599625 | down |
| LOC642366 | 7.80E-09 | 29.562178 | down |
| LOC102724462 | 1.66E-08 | 29.207733 | down |
| UGT1A6 | 8.41E-10 | 27.670906 | down |
| MIR100HG | 3.01E-10 | 27.538528 | down |
| LIMCH1 | 1.88E-08 | 27.292912 | down |
| SAMD9L | 1.93E-07 | 27.159569 | down |
| B4GALNT1 | 1.30E-07 | 26.164381 | down |
| TMEM130 | 1.04E-09 | 25.743525 | down |
| NINJ2 | 4.16E-07 | 25.73756 | down |
| THBS2 | 3.85E-10 | 25.680382 | down |
| FETUB | 1.00E-08 | 25.632988 | down |
| CCL5 | 4.89E-09 | 25.480621 | down |
| SPX | 5.61E-08 | 25.100727 | down |
| DNAJC12 | 3.40E-07 | 24.55709 | down |
| APBB1IP | 6.25E-09 | 24.469482 | down |
| TRABD2A | 2.29E-07 | 24.076899 | down |
| LIMCH1 | 4.20E-09 | 24.003162 | down |
| LCN2 | 1.00E-08 | 23.906965 | down |
| GREB1 | 8.04E-07 | 23.439121 | down |
| MOK | 2.07E-09 | 23.33756 | down |
| C1S | 9.87E-07 | 23.200611 | down |
| APBB1IP | 1.07E-08 | 22.131306 | down |
| C5orf58 | 3.10E-08 | 22.129433 | down |
| C17orf67 | 7.79E-08 | 21.97195 | down |
| FGFR2 | 4.42E-09 | 21.828024 | down |
| LRRC7 | 2.23E-08 | 21.345226 | down |
| PAX3 | 2.76E-08 | 20.885544 | down |
| LOC344887 | 8.31E-09 | 20.853386 | down |
| C1R | 1.24E-09 | 20.6178 | down |
| CXCL2 | 4.79E-10 | 20.4736 | down |
| BST2 | 8.50E-10 | 20.446442 | down |
| XLOC_l2_009136 | 8.85E-08 | 20.421412 | down |
| TFAP2A | 1.85E-08 | 20.351793 | down |
| RASGEF1A | 9.69E-10 | 20.196707 | down |
| PPP2R2C | 7.08E-07 | 20.149107 | down |
| C1orf186 | 3.95E-10 | 20.122213 | down |
| GSTT2B | 7.16E-10 | 20.061398 | down |
| BST2 | 3.40E-09 | 19.963318 | down |
| RASL11A | 1.55E-08 | 19.848248 | down |
| PLEKHG4 | 3.09E-09 | 19.694304 | down |
| FABP6 | 1.29E-07 | 19.679903 | down |
| KCNN4 | 8.72E-10 | 19.311356 | down |
| LOC100133669 | 3.30E-07 | 19.190052 | down |
| MOK | 6.37E-08 | 18.996618 | down |
| SLC22A18AS | 5.54E-07 | 18.757181 | down |
| LMO1 | 1.91E-08 | 18.730963 | down |
| ATP6V0A4 | 6.92E-08 | 18.446 | down |
| UGT1A8 | 1.87E-09 | 18.237585 | down |
| FOXA1 | 4.38E-07 | 18.105501 | down |
| KRTAP1-1 | 3.11E-08 | 17.862572 | down |
| PDZK1IP1 | 6.80E-09 | 17.852224 | down |
| VSTM1 | 3.01E-08 | 17.836342 | down |
| HHIPL2 | 4.40E-09 | 17.802876 | down |
| PRAME | 7.02E-09 | 17.799816 | down |
| AQP9 | 7.51E-09 | 17.6982 | down |
| STK32A | 1.69E-07 | 17.546171 | down |
| PHEX | 1.36E-08 | 17.466415 | down |
| C1R | 6.06E-09 | 17.357187 | down |
| TMTC1 | 8.29E-07 | 17.24186 | down |
| CD163L1 | 2.80E-08 | 17.172632 | down |
| LPXN | 3.26E-08 | 17.10649 | down |
| IGFN1 | 1.59E-07 | 16.630629 | down |
| P2RY6 | 1.73E-09 | 16.59247 | down |
| DCAF12L1 | 7.57E-09 | 16.487652 | down |
| LOC389895 | 8.56E-07 | 16.477257 | down |
| RASGEF1A | 1.08E-08 | 16.473297 | down |
| THBS2 | 2.42E-08 | 16.473103 | down |
| TWIST1 | 5.10E-08 | 16.44069 | down |
| IL7 | 3.07E-07 | 16.436995 | down |
| NFIB | 6.25E-07 | 16.285612 | down |
| RTP4 | 1.89E-09 | 16.128202 | down |
| AKR1C1 | 5.99E-09 | 15.96359 | down |
| OAS1 | 9.60E-09 | 15.897738 | down |
| AFP | 1.45E-08 | 15.531939 | down |
| GP6 | 6.26E-08 | 15.27838 | down |
| HAS3 | 2.56E-08 | 15.276851 | down |
| CAPZB | 2.60E-07 | 15.274593 | down |
| ITM2A | 1.11E-08 | 15.246884 | down |
| EHF | 1.78E-08 | 15.244919 | down |
| FAM43A | 1.50E-09 | 15.229722 | down |
| PNLIPRP3 | 2.37E-08 | 15.175215 | down |
| SLC15A3 | 3.27E-07 | 15.154388 | down |
| EFHB | 4.03E-09 | 14.906687 | down |
| SHOX2 | 1.12E-08 | 14.893936 | down |
| AKR1C1 | 6.31E-09 | 14.892927 | down |
| PLEKHG4 | 1.15E-08 | 14.570255 | down |
| PDE4DIP | 2.41E-08 | 14.352786 | down |
| ALDH1A1 | 1.65E-07 | 14.318216 | down |
| C8orf31 | 1.77E-07 | 14.291888 | down |
| ADAMTS18 | 3.61E-07 | 14.181519 | down |
| B3GNT3 | 1.49E-08 | 14.153217 | down |
| UGT1A6 | 9.22E-10 | 14.132948 | down |
| CA2 | 1.29E-09 | 13.929842 | down |
| FMOD | 6.34E-07 | 13.767867 | down |
| PPP1R1C | 9.07E-10 | 13.751307 | down |
| HAVCR1 | 2.72E-08 | 13.620075 | down |
| TM4SF19 | 7.45E-09 | 13.59288 | down |
| CALHM2 | 1.00E-09 | 13.537087 | down |
| JRK | 4.77E-07 | 13.510943 | down |
| MIR99AHG | 3.13E-07 | 13.462096 | down |
| HNMT | 1.11E-06 | 13.153567 | down |
| SLC47A1 | 5.10E-08 | 13.08543 | down |
| PLEK | 3.97E-08 | 13.031936 | down |
| CDKN2C | 1.88E-08 | 12.933626 | down |
| CA8 | 3.47E-07 | 12.797112 | down |
| OASL | 3.85E-08 | 12.719616 | down |
| GFPT2 | 9.72E-10 | 12.641689 | down |
| AVPR2 | 1.07E-08 | 12.610208 | down |
| KRT32 | 4.92E-08 | 12.44029 | down |
| TNFSF14 | 1.29E-08 | 12.393551 | down |
| TPK1 | 3.15E-09 | 12.383279 | down |
| DNM3 | 3.05E-09 | 12.320075 | down |
| USF1 | 9.14E-09 | 12.218746 | down |
| THNSL2 | 1.84E-08 | 12.17379 | down |
| DGAT2 | 4.22E-09 | 12.102085 | down |
| VNN1 | 1.89E-07 | 12.094985 | down |
| PDE2A | 6.50E-08 | 12.061707 | down |
| FCHO1 | 8.85E-10 | 11.921249 | down |
| TLR1 | 4.39E-09 | 11.918064 | down |
| C1QTNF1-AS1 | 7.62E-08 | 11.587888 | down |
| TDRD9 | 2.37E-08 | 11.549288 | down |
| IFI16 | 7.00E-09 | 11.526948 | down |
| AOAH | 6.96E-08 | 11.408459 | down |
| lnc-TOMM7-1 | 7.74E-07 | 11.365785 | down |
| KIAA1324 | 1.04E-09 | 11.290552 | down |
| OR51E2 | 8.52E-08 | 11.28013 | down |
| QRFPR | 2.93E-07 | 11.16069 | down |
| FLVCR1-AS1 | 7.62E-08 | 11.111274 | down |
| ABCC13 | 2.84E-08 | 11.106747 | down |
| CYP4F11 | 4.59E-07 | 11.09481 | down |
| IL22RA1 | 3.29E-08 | 11.036603 | down |
| RGS22 | 3.50E-07 | 10.97106 | down |
| LCN1 | 3.96E-08 | 10.924087 | down |
| MANEAL | 1.86E-08 | 10.87538 | down |
| GDA | 8.89E-08 | 10.859679 | down |
| TNFRSF1B | 1.20E-06 | 10.753032 | down |
| STAMBPL1 | 5.56E-09 | 10.692004 | down |
| LAMA4 | 9.30E-08 | 10.674629 | down |
| ANKRD20A8P | 1.12E-06 | 10.568502 | down |
| MANSC1 | 1.34E-06 | 10.512441 | down |
| SOD3 | 1.57E-08 | 10.509057 | down |
| TEX19 | 7.86E-08 | 10.471175 | down |
| DMGDH | 3.39E-08 | 10.451653 | down |
| PRKCE | 1.50E-08 | 10.429932 | down |
| C6orf58 | 4.49E-07 | 10.41306 | down |
| HERC5 | 1.23E-08 | 10.389185 | down |
| GCNT3 | 6.49E-07 | 10.317641 | down |
| KIF6 | 9.03E-07 | 10.299139 | down |
| TSPAN8 | 1.43E-07 | 10.272833 | down |
| NFIB | 4.12E-08 | 10.231256 | down |
| OAS2 | 2.12E-07 | 10.163556 | down |
| RNF213 | 2.64E-09 | 10.130569 | down |
| NRG2 | 1.44E-06 | 10.05881 | down |
| PDE4DIP | 2.60E-08 | 10.057273 | down |
| ZFYVE28 | 2.59E-09 | 10.009983 | down |
| SPATA3-AS1 | 8.25E-08 | 9.975815 | down |
| PAX8-AS1 | 1.10E-07 | 9.968129 | down |
| SRGN | 1.50E-08 | 9.850788 | down |
| ATOH8 | 1.68E-08 | 9.838261 | down |
| HLA-DMA | 5.02E-08 | 9.822597 | down |
| KCND1 | 2.37E-07 | 9.726073 | down |
| KCNJ2 | 9.70E-08 | 9.677745 | down |
| GSTT2 | 1.23E-08 | 9.670595 | down |
| C15orf48 | 6.07E-09 | 9.661258 | down |
| SCIN | 1.73E-08 | 9.646683 | down |
| LINC00923 | 1.09E-06 | 9.539703 | down |
| SOX2 | 7.52E-08 | 9.518933 | down |
| AKNAD1 | 9.06E-08 | 9.496169 | down |
| SCARNA16 | 3.10E-08 | 9.40115 | down |
| NUDT16P1 | 1.23E-06 | 9.39716 | down |
| PITX1 | 7.79E-08 | 9.345581 | down |
| EPN3 | 7.99E-08 | 9.339803 | down |
| DDC | 5.24E-07 | 9.328867 | down |
| MR1 | 2.53E-07 | 9.31652 | down |
| CAPS2 | 5.59E-08 | 9.263916 | down |
| UNC13C | 2.33E-08 | 9.26097 | down |
| FGFR2 | 8.07E-07 | 9.257834 | down |
| BMP4 | 8.12E-09 | 9.252166 | down |
| FAM101A | 6.44E-08 | 9.228537 | down |
| LINC00886 | 8.58E-07 | 9.222109 | down |
| RAB42 | 1.39E-08 | 9.200649 | down |
| SELL | 1.69E-07 | 9.123803 | down |
| POU4F1 | 1.62E-07 | 9.104167 | down |
| HGD | 6.68E-09 | 9.054109 | down |
| LIN28B | 1.37E-08 | 8.906838 | down |
| NFIB | 4.26E-08 | 8.903262 | down |
| TMEM255A | 1.10E-06 | 8.898723 | down |
| SOWAHD | 2.65E-08 | 8.893701 | down |
| CCDC88B | 1.78E-08 | 8.86281 | down |
| MEIG1 | 9.39E-07 | 8.773432 | down |
| DHRS3 | 7.66E-09 | 8.701437 | down |
| LOC254896 | 3.33E-09 | 8.568885 | down |
| CPLX1 | 4.57E-08 | 8.558904 | down |
| AKR1C1 | 1.14E-06 | 8.557283 | down |
| GABRE | 3.05E-08 | 8.548103 | down |
| EXOC7 | 2.19E-07 | 8.531246 | down |
| LRRC7 | 5.50E-07 | 8.515859 | down |
| RAB3IL1 | 6.59E-07 | 8.498176 | down |
| KIAA1324 | 3.16E-09 | 8.48727 | down |
| IFI44 | 1.07E-08 | 8.485934 | down |
| ANGPTL4 | 2.77E-09 | 8.469397 | down |
| TMTC1 | 1.44E-06 | 8.444904 | down |
| CXCL2 | 2.46E-09 | 8.440975 | down |
| AQP11 | 1.24E-07 | 8.416381 | down |
| LINC-PINT | 1.54E-08 | 8.3891735 | down |
| LAT2 | 2.20E-08 | 8.376555 | down |
| SLC19A3 | 8.53E-09 | 8.376531 | down |
| TCHH | 1.53E-07 | 8.357435 | down |
| ANKRD20A11P | 1.36E-07 | 8.280973 | down |
| NOS1AP | 1.17E-06 | 8.222561 | down |
| RASSF10 | 5.09E-09 | 8.204376 | down |
| SIRPG | 2.50E-08 | 8.163807 | down |
| PIR | 8.60E-09 | 8.104712 | down |
| B3GNTL1 | 1.32E-08 | 8.078415 | down |
| CTSH | 2.16E-09 | 8.047358 | down |
| CNBD1 | 3.50E-07 | 8.022235 | down |
| JAKMIP3 | 3.18E-07 | 7.990258 | down |
| FOXE1 | 1.23E-06 | 7.951796 | down |
| MS4A4A | 9.98E-07 | 7.924184 | down |
| TOR4A | 4.50E-08 | 7.8937874 | down |
| HTATIP2 | 9.93E-09 | 7.8324537 | down |
| MAPT | 3.27E-07 | 7.830533 | down |
| SLC9A9 | 8.00E-08 | 7.825014 | down |
| C10orf90 | 6.08E-07 | 7.8198643 | down |
| HRCT1 | 9.44E-08 | 7.8042145 | down |
| CCDC88B | 1.52E-08 | 7.77846 | down |
| C4orf19 | 1.33E-07 | 7.7715077 | down |
| GRIN2B | 2.77E-07 | 7.762556 | down |
| DPP9-AS1 | 1.69E-08 | 7.7612824 | down |
| SPIRE2 | 3.39E-07 | 7.743881 | down |
| FAM90A1 | 1.94E-08 | 7.713357 | down |
| C7orf69 | 1.58E-08 | 7.6867194 | down |
| PKP3 | 2.00E-08 | 7.5980988 | down |
| RNF213 | 1.73E-08 | 7.5874043 | down |
| PITX2 | 3.77E-08 | 7.5270376 | down |
| NOL6 | 1.17E-07 | 7.519188 | down |
| PXDNL | 2.75E-08 | 7.514003 | down |
| CYP3A7 | 3.74E-07 | 7.4772515 | down |
| ASB9 | 3.77E-09 | 7.453373 | down |
| NFIB | 4.60E-07 | 7.4396834 | down |
| WDR66 | 1.70E-07 | 7.361267 | down |
| TSPAN15 | 3.10E-09 | 7.3546715 | down |
| SLC25A19 | 6.34E-09 | 7.308188 | down |
| HNF4G | 5.50E-07 | 7.3070226 | down |
| LAMA4 | 2.93E-08 | 7.277132 | down |
| ANKRD2 | 7.53E-09 | 7.220253 | down |
| DLX2 | 4.57E-08 | 7.2136908 | down |
| IFI44L | 6.82E-08 | 7.2048874 | down |
| PDGFRL | 4.01E-07 | 7.125563 | down |
| HPCAL1 | 1.16E-07 | 7.0484033 | down |
| SLIT2 | 7.61E-07 | 7.0373044 | down |
| RHCE | 4.98E-07 | 7.013899 | down |
| LAMB3 | 1.78E-08 | 7.0079207 | down |
| TTYH2 | 4.57E-07 | 6.99847 | down |
| ITGB2 | 8.67E-07 | 6.9944763 | down |
| SCARNA10 | 9.59E-07 | 6.9487805 | down |
| IL7R | 5.50E-07 | 6.948062 | down |
| TFPI | 7.25E-09 | 6.9449887 | down |
| CASP10 | 7.44E-09 | 6.9322915 | down |
| ITGBL1 | 7.00E-07 | 6.891749 | down |
| NLRP3 | 1.26E-07 | 6.867225 | down |
| lnc-FOXI2-1 | 2.11E-08 | 6.859349 | down |
| RIN1 | 5.24E-09 | 6.856713 | down |
| NPLOC4 | 1.96E-08 | 6.8055634 | down |
| UAP1 | 4.84E-09 | 6.7970896 | down |
| TMEM229B | 1.30E-08 | 6.7907305 | down |
| CHST4 | 1.37E-07 | 6.790121 | down |
| SATB1-AS1 | 7.11E-07 | 6.7716713 | down |
| STAT4 | 1.14E-07 | 6.7688875 | down |
| C10orf54 | 4.22E-08 | 6.765697 | down |
| TOX2 | 1.18E-08 | 6.7536345 | down |
| ZFAND2A | 2.90E-08 | 6.737977 | down |
| RAD21L1 | 1.32E-07 | 6.703688 | down |
| CATSPER1 | 5.74E-08 | 6.684992 | down |
| TTPA | 3.02E-07 | 6.6794925 | down |
| FMN2 | 6.83E-07 | 6.6667166 | down |
| CLU | 2.34E-08 | 6.6612124 | down |
| TXNRD1 | 4.66E-08 | 6.649412 | down |
| CHST9 | 1.09E-07 | 6.6346903 | down |
| GRM8 | 3.49E-08 | 6.5952287 | down |
| SYNPO | 1.33E-07 | 6.5802474 | down |
| SP140 | 8.03E-09 | 6.5740333 | down |
| HLA-DPB1 | 3.03E-08 | 6.567733 | down |
| SULT2B1 | 6.29E-07 | 6.550812 | down |
| PSMB9 | 7.39E-09 | 6.5239735 | down |
| MIRLET7BHG | 5.68E-08 | 6.512283 | down |
| MOK | 1.64E-07 | 6.5035186 | down |
| HOXD13 | 4.56E-07 | 6.487314 | down |
| GPR19 | 6.86E-07 | 6.472542 | down |
| GAP43 | 2.21E-08 | 6.4653354 | down |
| LY96 | 7.90E-08 | 6.464535 | down |
| MSR1 | 2.63E-07 | 6.440423 | down |
| HRASLS2 | 9.05E-07 | 6.3930407 | down |
| OR5L2 | 9.88E-07 | 6.3627696 | down |
| CASP4 | 1.17E-08 | 6.3228006 | down |
| ZNF664-FAM101A | 5.33E-09 | 6.306322 | down |
| TIMD4 | 2.86E-07 | 6.3004155 | down |
| ALDH3B1 | 1.34E-08 | 6.2858057 | down |
| OSGIN1 | 4.15E-08 | 6.229509 | down |
| HLA-F | 2.04E-07 | 6.199139 | down |
| MAP3K14 | 2.19E-08 | 6.1308312 | down |
| PADI1 | 1.49E-08 | 6.1132827 | down |
| CRABP1 | 8.08E-07 | 6.111637 | down |
| ABCC3 | 3.17E-08 | 6.070709 | down |
| NLRP3 | 1.26E-07 | 6.037398 | down |
| CASP5 | 4.84E-08 | 6.008223 | down |
| CASP8 | 8.22E-07 | 6.0026436 | down |
| SLC47A1 | 6.36E-07 | 5.984535 | down |
| ACTL8 | 4.94E-07 | 5.945866 | down |
| REPS2 | 1.22E-06 | 5.9452844 | down |
| NEK10 | 4.98E-07 | 5.9446135 | down |
| RNF148 | 6.85E-08 | 5.9202294 | down |
| PRR7-AS1 | 2.28E-07 | 5.9120626 | down |
| IL1B | 2.98E-07 | 5.910314 | down |
| IRX5 | 1.07E-07 | 5.8882995 | down |
| DDO | 1.24E-06 | 5.866687 | down |
| TMEM178B | 3.85E-07 | 5.86275 | down |
| TLR6 | 5.57E-07 | 5.822912 | down |
| TRGV7 | 5.23E-07 | 5.807063 | down |
| TIMD4 | 1.04E-06 | 5.8004727 | down |
| CASP8 | 8.00E-09 | 5.7850504 | down |
| HLA-DPA1 | 8.52E-08 | 5.761582 | down |
| OR51B2 | 4.52E-07 | 5.7081146 | down |
| LOC100996273 | 1.15E-06 | 5.696891 | down |
| CXCL1 | 2.31E-08 | 5.690961 | down |
| LINC00857 | 1.10E-07 | 5.684693 | down |
| KRT33B | 9.92E-08 | 5.6715083 | down |
| USP40 | 1.59E-07 | 5.656783 | down |
| GDPD3 | 2.27E-07 | 5.6407604 | down |
| AKR1B1 | 2.06E-08 | 5.6352158 | down |
| CXCL1 | 1.11E-08 | 5.5978923 | down |
| MSX1 | 7.01E-07 | 5.596275 | down |
| UBE2L6 | 8.93E-09 | 5.558738 | down |
| GAB3 | 3.88E-07 | 5.5513015 | down |
| AGTR1 | 6.28E-07 | 5.547729 | down |
| DBP | 9.00E-08 | 5.543903 | down |
| KMO | 3.54E-08 | 5.5350685 | down |
| SLC3A1 | 8.19E-07 | 5.4883556 | down |
| SERPINE1 | 1.10E-06 | 5.474392 | down |
| NQO1 | 8.94E-09 | 5.4573054 | down |
| RARRES3 | 3.44E-08 | 5.4270444 | down |
| ZC3HAV1 | 1.09E-06 | 5.407161 | down |
| lnc-ITGA2-1 | 1.00E-07 | 5.400887 | down |
| CXCL8 | 6.83E-07 | 5.3475456 | down |
| FLJ45513 | 2.20E-07 | 5.3193164 | down |
| NECAB2 | 1.37E-06 | 5.3129115 | down |
| ABI3BP | 8.44E-07 | 5.296244 | down |
| XK | 3.21E-08 | 5.28331 | down |
| TSPAN10 | 2.68E-07 | 5.2809267 | down |
| KRT34 | 1.30E-08 | 5.280153 | down |
| VNN2 | 1.04E-06 | 5.2779293 | down |
| KLF2 | 2.45E-07 | 5.245522 | down |
| IFRD1 | 3.03E-07 | 5.2341886 | down |
| PAPSS2 | 1.39E-07 | 5.2271886 | down |
| ADD2 | 2.80E-08 | 5.22286 | down |
| TRIM16L | 1.15E-07 | 5.1921334 | down |
| PRR20B | 6.74E-07 | 5.1891246 | down |
| MTRR | 9.06E-07 | 5.1791763 | down |
| CYB5A | 3.23E-08 | 5.1417174 | down |
| ACSL5 | 1.91E-07 | 5.1415043 | down |
| AMZ2 | 3.99E-07 | 5.1380363 | down |
| PCGF3 | 4.78E-08 | 5.137052 | down |
| CASP10 | 6.26E-07 | 5.120162 | down |
| MAFG-AS1 | 1.43E-08 | 5.1028867 | down |
| GAS6-AS1 | 1.00E-07 | 5.077788 | down |
| CYP3A5 | 1.03E-06 | 5.0642204 | down |
| EMR1 | 2.49E-08 | 5.0563116 | down |
| XLOC_l2_003882 | 1.43E-07 | 5.0538197 | down |
| CCDC80 | 6.58E-08 | 5.042534 | down |
| GPRC5C | 3.49E-08 | 5.0392017 | down |
| MCOLN2 | 9.85E-08 | 5.0375624 | down |
| TLCD1 | 2.75E-08 | 5.0374656 | down |
| LINC00467 | 1.54E-08 | 5.0182924 | down |
| ANXA2R | 6.74E-08 | 5.013969 | down |
| PRDM8 | 9.46E-07 | 5.012612 | down |
| LINC01270 | 1.20E-07 | 5.012249 | down |
| CXCL6 | 8.69E-07 | 5.0067368 | down |
| KRT15 | 1.03E-07 | 4.95596 | down |
| GRID2IP | 1.26E-06 | 4.9516144 | down |
| C3 | 7.42E-07 | 4.9307427 | down |
| LRRC63 | 1.49E-07 | 4.9306955 | down |
| SPTBN4 | 2.27E-07 | 4.9278345 | down |
| FAM72A | 4.93E-08 | 4.9178524 | down |
| CD68 | 7.40E-08 | 4.9034057 | down |
| LOC101928669 | 2.49E-07 | 4.876196 | down |
| STK32C | 1.23E-06 | 4.8753686 | down |
| ADAM8 | 9.13E-08 | 4.872669 | down |
| JADE2 | 2.83E-08 | 4.870503 | down |
| CPT1A | 7.40E-08 | 4.86068 | down |
| RNA5-8S5 | 1.44E-06 | 4.854636 | down |
| LINC00467 | 5.72E-08 | 4.8460097 | down |
| ENTPD1 | 2.76E-07 | 4.8451138 | down |
| ECE2 | 5.94E-08 | 4.842695 | down |
| MGST1 | 8.55E-08 | 4.8384037 | down |
| CYB5A | 5.13E-08 | 4.826014 | down |
| CD55 | 1.16E-08 | 4.8166556 | down |
| RBP4 | 5.27E-08 | 4.7961597 | down |
| SQRDL | 8.82E-08 | 4.791069 | down |
| CIART | 1.04E-06 | 4.790145 | down |
| WWTR1 | 1.39E-08 | 4.7868667 | down |
| KYNU | 9.57E-07 | 4.775829 | down |
| FOSL1 | 9.40E-09 | 4.766869 | down |
| CREG1 | 2.58E-08 | 4.7324157 | down |
| QDPR | 2.07E-07 | 4.731701 | down |
| MGMT | 5.35E-08 | 4.7225046 | down |
| PTPLAD2 | 1.11E-08 | 4.721687 | down |
| METTL23 | 1.20E-08 | 4.6968617 | down |
| GPRC5C | 1.32E-08 | 4.6922913 | down |
| PRRG1 | 9.19E-08 | 4.681301 | down |
| HOXB4 | 1.30E-08 | 4.672986 | down |
| LOC730102 | 9.21E-07 | 4.671002 | down |
| VPS9D1-AS1 | 9.49E-07 | 4.667249 | down |
| NFIX | 2.17E-07 | 4.6621656 | down |
| MERTK | 8.69E-07 | 4.654826 | down |
| NR0B1 | 1.07E-08 | 4.650452 | down |
| PARP10 | 2.29E-07 | 4.6481094 | down |
| CENPVP2 | 8.49E-07 | 4.601197 | down |
| TK1 | 2.95E-08 | 4.5913982 | down |
| TRIM55 | 4.32E-08 | 4.5877485 | down |
| PPAPDC3 | 7.40E-08 | 4.578088 | down |
| LOC388282 | 2.84E-08 | 4.5387673 | down |
| SNHG20 | 1.56E-07 | 4.5243044 | down |
| LOC728819 | 4.66E-07 | 4.521511 | down |
| NR1H3 | 4.59E-08 | 4.4978304 | down |
| PTK2B | 1.03E-07 | 4.487769 | down |
| AMZ2P1 | 3.15E-08 | 4.462686 | down |
| TRIM55 | 4.28E-08 | 4.4411225 | down |
| SNAI1 | 2.47E-08 | 4.439304 | down |
| LOC440386 | 6.88E-07 | 4.4272447 | down |
| PYCR2 | 2.08E-08 | 4.4229155 | down |
| COA6 | 3.49E-08 | 4.4087405 | down |
| CACYBP | 2.48E-08 | 4.407005 | down |
| PLTP | 6.56E-08 | 4.3971457 | down |
| MRPL38 | 2.10E-07 | 4.3955665 | down |
| SNHG7 | 1.06E-07 | 4.3902087 | down |
| SQSTM1 | 6.25E-08 | 4.387464 | down |
| SNORA23 | 4.43E-07 | 4.384781 | down |
| WBSCR27 | 3.18E-08 | 4.369386 | down |
| GSAP | 4.35E-08 | 4.3626313 | down |
| FLRT2 | 2.97E-07 | 4.3543873 | down |
| IL6 | 1.47E-08 | 4.3486967 | down |
| SLC25A19 | 8.12E-08 | 4.3340697 | down |
| EEF1A2 | 3.29E-07 | 4.3302927 | down |
| CDC42 | 8.08E-08 | 4.317669 | down |
| STEAP1 | 2.07E-07 | 4.316176 | down |
| BSCL2 | 1.94E-07 | 4.306827 | down |
| MRPL12 | 4.12E-08 | 4.300155 | down |
| AREG | 3.53E-07 | 4.2920012 | down |
| CACYBP | 4.80E-08 | 4.285625 | down |
| CDA | 2.06E-08 | 4.2823863 | down |
| SLC25A22 | 6.84E-08 | 4.281694 | down |
| EID3 | 4.10E-08 | 4.2746468 | down |
| LOC440028 | 2.37E-07 | 4.2641354 | down |
| LAT | 1.99E-08 | 4.2533417 | down |
| HLA-DPB1 | 2.19E-08 | 4.252635 | down |
| CAPG | 1.53E-07 | 4.250876 | down |
| PREX1 | 9.19E-07 | 4.2388964 | down |
| ALDH3B1 | 5.23E-08 | 4.2344337 | down |
| SPSB2 | 2.42E-08 | 4.2272196 | down |
| USP30-AS1 | 1.29E-07 | 4.2261963 | down |
| ID2 | 1.64E-07 | 4.224787 | down |
| DDIT3 | 1.63E-08 | 4.1937604 | down |
| MRPL12 | 6.07E-08 | 4.18818 | down |
| GPRC5A | 2.30E-07 | 4.187273 | down |
| HIST2H3A | 8.54E-07 | 4.1848993 | down |
| ZDHHC23 | 3.72E-08 | 4.182723 | down |
| CYB561 | 2.88E-08 | 4.1695957 | down |
| C3 | 2.05E-08 | 4.1678386 | down |
| GMPR | 1.24E-07 | 4.153266 | down |
| CITED2 | 2.53E-08 | 4.1529384 | down |
| TMEM180 | 1.35E-07 | 4.151722 | down |
| LOC100422737 | 4.41E-07 | 4.1500196 | down |
| SNORA12 | 1.17E-07 | 4.1346726 | down |
| PARP1 | 5.43E-07 | 4.119618 | down |
| TMEM164 | 2.28E-08 | 4.113812 | down |
| SULT1A2 | 2.45E-08 | 4.099501 | down |
| ST7-OT4 | 1.17E-06 | 4.092437 | down |
| ADCY9 | 3.10E-07 | 4.0905504 | down |
| C8orf46 | 7.37E-07 | 4.0896783 | down |
| SLC41A1 | 6.97E-07 | 4.088133 | down |
| ID2 | 9.29E-08 | 4.075533 | down |
| LACTB2 | 3.76E-08 | 4.0726056 | down |
| GALNT18 | 1.15E-07 | 4.065036 | down |
| PRRX1 | 3.68E-07 | 4.059406 | down |
| EAF2 | 8.52E-08 | 4.058761 | down |
| STEAP1 | 2.86E-07 | 4.05473 | down |
| SLC43A3 | 9.49E-07 | 4.0482154 | down |
| SNCA | 3.31E-08 | 4.046579 | down |
| PTPRR | 1.01E-07 | 4.0416045 | down |
| GGCT | 4.12E-07 | 4.0386868 | down |
| SYNPO | 5.80E-08 | 4.037536 | down |
| SCARNA12 | 4.41E-07 | 4.034454 | down |
| HDGF | 1.54E-07 | 4.0323324 | down |
| BCHE | 3.45E-08 | 4.021937 | down |
| SNHG1 | 2.81E-08 | 4.0181255 | down |
| PYCR2 | 2.01E-07 | 4.0028615 | down |
| C1orf226 | 8.60E-07 | 3.9871316 | down |
| LOC644662 | 1.38E-06 | 3.9763618 | down |
| PCOLCE2 | 3.66E-08 | 3.9582698 | down |
| AGPAT9 | 5.20E-08 | 3.956955 | down |
| LAMA3 | 3.80E-07 | 3.9496806 | down |
| RFESD | 4.81E-07 | 3.9394767 | down |
| GYG2 | 2.15E-07 | 3.9337888 | down |
| MAP3K15 | 2.06E-07 | 3.929638 | down |
| TNFSF9 | 4.21E-08 | 3.9238229 | down |
| AOX1 | 1.55E-07 | 3.9142656 | down |
| ZBTB7B | 2.89E-08 | 3.9080977 | down |
| EPB41L1 | 1.77E-07 | 3.90106 | down |
| AKNAD1 | 2.64E-08 | 3.89766 | down |
| HSPA4L | 1.05E-07 | 3.8863106 | down |
| TUB | 1.58E-07 | 3.88283 | down |
| LOC645166 | 2.68E-07 | 3.8758771 | down |
| DOCK10 | 5.37E-08 | 3.8748736 | down |
| LOC101928000 | 8.45E-08 | 3.874684 | down |
| SLC48A1 | 1.92E-08 | 3.8738222 | down |
| PBX1 | 5.46E-08 | 3.8711665 | down |
| KATNAL2 | 1.65E-07 | 3.8701582 | down |
| MARCH3 | 5.60E-08 | 3.869155 | down |
| SULT1A4 | 5.01E-08 | 3.8504584 | down |
| C2orf42 | 2.16E-07 | 3.8485441 | down |
| SLC48A1 | 7.19E-08 | 3.841643 | down |
| CITED2 | 1.19E-06 | 3.8413818 | down |
| LOC399815 | 5.15E-07 | 3.839855 | down |
| SYBU | 3.78E-07 | 3.8358274 | down |
| C17orf67 | 1.73E-07 | 3.831794 | down |
| IGFLR1 | 4.41E-08 | 3.8270597 | down |
| ACOT11 | 2.74E-07 | 3.8257794 | down |
| AZIN2 | 6.21E-07 | 3.8237543 | down |
| IFI30 | 4.41E-08 | 3.8225129 | down |
| LINC01315 | 1.18E-07 | 3.8185577 | down |
| SOCS3 | 6.41E-07 | 3.8092003 | down |
| DLEU1 | 2.63E-08 | 3.7923374 | down |
| AMZ2 | 2.72E-07 | 3.7922096 | down |
| ARAP3 | 8.38E-08 | 3.7862291 | down |
| FOXK2 | 7.44E-08 | 3.7795687 | down |
| CDYL2 | 2.31E-07 | 3.771078 | down |
| PTPRR | 6.04E-07 | 3.7625077 | down |
| RAD51C | 1.26E-07 | 3.757785 | down |
| JMJD6 | 6.73E-07 | 3.7546144 | down |
| DAW1 | 2.28E-07 | 3.7520103 | down |
| CYB5R2 | 6.41E-07 | 3.7463527 | down |
| GLRX2 | 4.33E-08 | 3.739805 | down |
| RPTOR | 5.89E-08 | 3.7340977 | down |
| SYTL2 | 3.51E-07 | 3.7329445 | down |
| BAX | 1.39E-06 | 3.7309005 | down |
| AOX1 | 3.83E-08 | 3.727258 | down |
| GPR39 | 7.49E-08 | 3.7170022 | down |
| GDF15 | 9.33E-08 | 3.715446 | down |
| RECQL5 | 1.19E-07 | 3.7145913 | down |
| SPDL1 | 2.78E-07 | 3.7136974 | down |
| ELF3 | 3.48E-07 | 3.70797 | down |
| PTGS1 | 9.92E-07 | 3.698989 | down |
| LAGE3 | 2.80E-08 | 3.6880257 | down |
| ANKRD37 | 6.23E-08 | 3.676113 | down |
| DHODH | 4.03E-08 | 3.6641605 | down |
| FANCA | 7.75E-07 | 3.657286 | down |
| PLD6 | 1.37E-07 | 3.6557865 | down |
| RASSF10 | 3.44E-08 | 3.654546 | down |
| PLEKHB1 | 1.08E-07 | 3.6532805 | down |
| FAM222A | 5.71E-08 | 3.6524866 | down |
| PKP2 | 1.13E-07 | 3.6508083 | down |
| GPRC5B | 6.67E-08 | 3.6448293 | down |
| TMEM180 | 5.83E-08 | 3.6401608 | down |
| GPR160 | 1.40E-06 | 3.6347575 | down |
| TMEM79 | 9.63E-07 | 3.6262388 | down |
| TMEM139 | 3.67E-08 | 3.6180472 | down |
| E2F1 | 4.97E-08 | 3.6099818 | down |
| ANTXR1 | 6.99E-07 | 3.6034286 | down |
| JPH1 | 1.03E-06 | 3.593566 | down |
| CAPG | 8.23E-08 | 3.5887117 | down |
| BIRC3 | 2.46E-07 | 3.5718348 | down |
| HERC4 | 6.52E-08 | 3.5680609 | down |
| CHORDC1 | 6.99E-07 | 3.5640824 | down |
| OXLD1 | 4.10E-07 | 3.561933 | down |
| SNORA2A | 1.21E-06 | 3.5488102 | down |
| AFMID | 4.98E-07 | 3.546172 | down |
| RGS2 | 5.58E-08 | 3.545571 | down |
| PRR13 | 1.70E-07 | 3.541993 | down |
| TRIM47 | 8.44E-08 | 3.5358896 | down |
| SLMO1 | 3.52E-07 | 3.5318787 | down |
| RRP7A | 7.31E-08 | 3.5306246 | down |
| RBM33 | 5.79E-08 | 3.5284214 | down |
| SSH2 | 2.86E-07 | 3.528157 | down |
| ARHGAP28 | 6.69E-07 | 3.527957 | down |
| SERTAD4 | 1.08E-07 | 3.5272963 | down |
| FGF2 | 6.74E-07 | 3.520659 | down |
| CCDC148 | 3.08E-07 | 3.515791 | down |
| DPYD | 4.23E-07 | 3.5149128 | down |
| MTHFSD | 1.14E-07 | 3.5144286 | down |
| BLVRB | 9.58E-07 | 3.5056767 | down |
| AFMID | 1.00E-06 | 3.5005243 | down |
| PTER | 8.37E-08 | 3.4994164 | down |
| CYB5R2 | 4.95E-08 | 3.4960341 | down |
| SEH1L | 2.26E-07 | 3.4868703 | down |
| IFIT2 | 4.45E-07 | 3.4710083 | down |
| IFI35 | 4.53E-07 | 3.4667654 | down |
| SLC27A2 | 5.69E-07 | 3.4540389 | down |
| DTX2 | 3.43E-08 | 3.4539533 | down |
| TMEM99 | 2.03E-07 | 3.4531837 | down |
| CHMP6 | 9.41E-08 | 3.4490194 | down |
| SPDL1 | 8.23E-07 | 3.4471598 | down |
| GABRQ | 6.26E-07 | 3.4450352 | down |
| ZMIZ1-AS1 | 7.36E-08 | 3.4431908 | down |
| TM7SF3 | 1.04E-07 | 3.4405632 | down |
| EP400NL | 2.06E-07 | 3.4390454 | down |
| PACRGL | 2.77E-07 | 3.4366803 | down |
| PPAP2C | 8.24E-08 | 3.434713 | down |
| MROH1 | 9.21E-07 | 3.4285574 | down |
| FGF2 | 1.15E-06 | 3.42781 | down |
| RILPL2 | 5.76E-08 | 3.4277012 | down |
| NMU | 1.50E-07 | 3.42104 | down |
| NAT8L | 3.91E-07 | 3.4187226 | down |
| OTUD4 | 2.08E-07 | 3.412701 | down |
| DKFZp686M1136 | 6.07E-07 | 3.4105623 | down |
| NOL3 | 1.25E-07 | 3.4000604 | down |
| NANOS1 | 2.69E-07 | 3.3913221 | down |
| B3GALNT2 | 6.72E-08 | 3.3881333 | down |
| LYN | 3.69E-08 | 3.387173 | down |
| CRNDE | 5.91E-07 | 3.3783467 | down |
| ITGB4 | 7.17E-07 | 3.3703237 | down |
| NECAB3 | 1.49E-07 | 3.3695424 | down |
| ACTG1P4 | 4.36E-07 | 3.367074 | down |
| TMPRSS7 | 1.33E-06 | 3.3644772 | down |
| ELK4 | 9.99E-07 | 3.3612819 | down |
| STRA13 | 3.78E-08 | 3.3594131 | down |
| FAM72D | 5.30E-07 | 3.3529396 | down |
| RNLS | 5.88E-07 | 3.3408759 | down |
| C17orf89 | 3.42E-07 | 3.3389773 | down |
| SLC39A4 | 4.96E-07 | 3.3356063 | down |
| GBP2 | 1.16E-06 | 3.3311555 | down |
| NR1D1 | 8.45E-08 | 3.330863 | down |
| CCDC80 | 6.47E-08 | 3.3305233 | down |
| FHOD1 | 1.05E-07 | 3.3298805 | down |
| HDGF | 1.26E-06 | 3.3298695 | down |
| AKIP1 | 5.48E-08 | 3.3256226 | down |
| FAM89A | 8.99E-08 | 3.3194168 | down |
| HIST2H2AB | 9.43E-07 | 3.3186443 | down |
| AARS | 1.57E-07 | 3.3173144 | down |
| SLC7A5 | 8.33E-08 | 3.3165374 | down |
| SFXN1 | 6.11E-07 | 3.3147328 | down |
| LETM2 | 2.27E-07 | 3.3121505 | down |
| SYTL3 | 8.24E-08 | 3.3092988 | down |
| SNORA10 | 8.56E-08 | 3.3085902 | down |
| SPR | 1.40E-07 | 3.306882 | down |
| SRGAP2 | 4.58E-07 | 3.3013794 | down |
| IMPA2 | 3.62E-07 | 3.2968497 | down |
| COPZ2 | 2.92E-07 | 3.2964494 | down |
| EMG1 | 1.87E-07 | 3.289826 | down |
| SGK2 | 6.18E-07 | 3.2832181 | down |
| SYNGR2 | 2.89E-07 | 3.273796 | down |
| GSTM2 | 9.52E-08 | 3.2675607 | down |
| ENKUR | 1.06E-07 | 3.257022 | down |
| GSTM2 | 1.15E-07 | 3.2563734 | down |
| MLX | 6.13E-08 | 3.2483847 | down |
| BCOR | 1.43E-06 | 3.2412128 | down |
| NOP16 | 6.61E-08 | 3.2348394 | down |
| EPAS1 | 3.14E-07 | 3.224488 | down |
| PAAF1 | 1.69E-07 | 3.2236347 | down |
| SIRT7 | 4.08E-07 | 3.2227228 | down |
| SLC5A12 | 1.03E-07 | 3.2211723 | down |
| lnc-INADL-2 | 1.26E-07 | 3.2186418 | down |
| C17orf89 | 2.17E-07 | 3.2179947 | down |
| ICT1 | 7.97E-08 | 3.2160497 | down |
| HMGA2 | 4.71E-08 | 3.2139518 | down |
| HRSP12 | 1.34E-07 | 3.2134197 | down |
| ELOVL5 | 2.40E-07 | 3.2046404 | down |
| CMSS1 | 7.21E-07 | 3.204502 | down |
| JTB | 6.78E-07 | 3.1922936 | down |
| MAPK9 | 1.07E-06 | 3.1906471 | down |
| BATF3 | 2.05E-07 | 3.1898663 | down |
| SRP68 | 4.77E-07 | 3.1896586 | down |
| ARSE | 4.55E-08 | 3.1848354 | down |
| CHORDC1 | 1.03E-07 | 3.1817093 | down |
| HBG1 | 5.83E-07 | 3.168113 | down |
| ADAMTSL1 | 1.25E-06 | 3.1677697 | down |
| SNHG8 | 5.25E-07 | 3.1651065 | down |
| OGFOD2 | 1.19E-07 | 3.1544483 | down |
| HAX1 | 6.42E-08 | 3.1529725 | down |
| LRRN3 | 2.44E-07 | 3.1513414 | down |
| LRIG1 | 4.40E-07 | 3.1510222 | down |
| TNFRSF14 | 5.35E-07 | 3.149845 | down |
| NAP1L4 | 5.69E-07 | 3.1468678 | down |
| GSR | 9.75E-07 | 3.145948 | down |
| PGS1 | 3.51E-07 | 3.1371973 | down |
| UNG | 2.00E-07 | 3.1313024 | down |
| C10orf11 | 1.60E-07 | 3.130437 | down |
| SELM | 8.54E-07 | 3.1271255 | down |
| FGFR3 | 6.71E-07 | 3.1156085 | down |
| DUSP23 | 1.02E-07 | 3.1143105 | down |
| USP36 | 3.49E-07 | 3.1091347 | down |
| MRPL55 | 2.81E-07 | 3.1071007 | down |
| MC1R | 1.10E-06 | 3.1050081 | down |
| LOC100132356 | 9.50E-07 | 3.1006708 | down |
| HPS5 | 1.23E-07 | 3.096604 | down |
| SMPD2 | 1.55E-07 | 3.094276 | down |
| ULK4 | 1.16E-06 | 3.0912142 | down |
| EXO1 | 4.34E-07 | 3.0891273 | down |
| RAB40B | 6.63E-08 | 3.088616 | down |
| CAMK2G | 1.30E-07 | 3.0871153 | down |
| NEK2 | 6.39E-07 | 3.0741248 | down |
| FLT3LG | 7.99E-07 | 3.0727046 | down |
| SCARB1 | 2.07E-07 | 3.0719204 | down |
| C7orf13 | 2.18E-07 | 3.067436 | down |
| MAL2 | 6.67E-08 | 3.0671227 | down |
| ARL16 | 1.58E-07 | 3.0668192 | down |
| LOC100128239 | 1.26E-07 | 3.066182 | down |
| NAA50 | 3.41E-07 | 3.0623002 | down |
| KLF11 | 5.99E-07 | 3.0603468 | down |
| PCBD1 | 3.80E-07 | 3.059947 | down |
| PTGFR | 9.06E-07 | 3.0552065 | down |
| PAAF1 | 7.75E-08 | 3.0550308 | down |
| ENTHD2 | 1.64E-07 | 3.0505214 | down |
| BIRC5 | 7.85E-08 | 3.0416696 | down |
| C1orf53 | 4.65E-07 | 3.0380635 | down |
| B3GALNT2 | 8.90E-07 | 3.035181 | down |
| TMEM138 | 2.23E-07 | 3.0325723 | down |
| HLA-DPB1 | 1.72E-07 | 3.0280368 | down |
| RUNX1-IT1 | 5.84E-07 | 3.0249622 | down |
| CTSD | 8.33E-07 | 3.024873 | down |
| STRADA | 1.64E-07 | 3.0241897 | down |
| JMJD4 | 1.52E-07 | 3.0201426 | down |
| PID1 | 1.26E-07 | 3.0143952 | down |
| ARHGEF19 | 5.25E-07 | 3.0134437 | down |
| ZMYND8 | 1.20E-06 | 3.0077343 | down |
| FZD2 | 9.89E-08 | 3.0049884 | down |
| FLYWCH1 | 1.13E-06 | 3.001585 | down |
| F8A1 | 1.22E-07 | 2.9935462 | down |
| DMRTA1 | 4.79E-07 | 2.9933705 | down |
| WFS1 | 8.90E-08 | 2.9890797 | down |
| F8A2 | 1.38E-07 | 2.9865348 | down |
| CCDC169 | 9.61E-07 | 2.9827483 | down |
| MGC34796 | 4.82E-07 | 2.9810605 | down |
| LINC01116 | 3.18E-07 | 2.9734972 | down |
| HEBP1 | 7.66E-07 | 2.9692836 | down |
| BAG3 | 7.36E-07 | 2.9652884 | down |
| GEMIN6 | 2.66E-07 | 2.963372 | down |
| NPLOC4 | 2.66E-07 | 2.9629588 | down |
| GNL3L | 2.73E-07 | 2.962741 | down |
| UCK2 | 9.10E-07 | 2.9622974 | down |
| MPZL1 | 2.23E-07 | 2.959811 | down |
| AZIN2 | 1.52E-07 | 2.958189 | down |
| TEAD4 | 1.49E-07 | 2.9578173 | down |
| CXorf38 | 4.00E-07 | 2.9440243 | down |
| ID1 | 2.85E-07 | 2.9397871 | down |
| RNASEK | 1.14E-06 | 2.9386148 | down |
| NT5C3A | 1.02E-07 | 2.9380393 | down |
| HTATIP2 | 9.76E-08 | 2.9373877 | down |
| GNB1L | 7.59E-07 | 2.9372447 | down |
| ZFAND1 | 7.86E-07 | 2.9371405 | down |
| MAOA | 1.02E-07 | 2.934229 | down |
| MRPS23 | 2.55E-07 | 2.9306002 | down |
| NDRG3 | 2.60E-07 | 2.9255924 | down |
| MAP2K6 | 4.24E-07 | 2.9206712 | down |
| SMG5 | 1.22E-06 | 2.9195154 | down |
| HCP5 | 9.86E-07 | 2.9164822 | down |
| FAM156A | 3.22E-07 | 2.9146261 | down |
| PI4KB | 3.66E-07 | 2.9133947 | down |
| DUS3L | 8.91E-07 | 2.9108534 | down |
| RGS9 | 1.14E-06 | 2.9107494 | down |
| SPC24 | 1.05E-06 | 2.9107108 | down |
| KLHL15 | 1.42E-06 | 2.9083939 | down |
| MED30 | 2.35E-07 | 2.9055388 | down |
| CTU2 | 3.73E-07 | 2.9042296 | down |
| GEM | 1.30E-06 | 2.9006426 | down |
| TRADD | 4.39E-07 | 2.8986523 | down |
| SNAPC5 | 9.49E-08 | 2.898294 | down |
| NT5C3A | 1.43E-06 | 2.8973348 | down |
| NOC3L | 4.34E-07 | 2.895668 | down |
| DHX58 | 3.37E-07 | 2.8946135 | down |
| PI4KB | 9.55E-08 | 2.8903306 | down |
| SNAPC5 | 1.44E-07 | 2.8863978 | down |
| PDCD4-AS1 | 1.39E-06 | 2.8849719 | down |
| FAM102A | 1.03E-06 | 2.879419 | down |
| UNKL | 3.35E-07 | 2.8754709 | down |
| SMAD6 | 3.22E-07 | 2.8737679 | down |
| lnc-GOLGA8J-3 | 6.24E-07 | 2.871176 | down |
| SFXN4 | 9.70E-08 | 2.8653052 | down |
| CISD1 | 1.10E-07 | 2.860359 | down |
| LY6K | 1.96E-07 | 2.8590899 | down |
| SCAMP3 | 8.81E-07 | 2.8558097 | down |
| SLC38A10 | 8.14E-07 | 2.8505487 | down |
| CUTC | 3.18E-07 | 2.8464892 | down |
| EPB41L1 | 5.63E-07 | 2.8449667 | down |
| CAMK2G | 1.91E-07 | 2.8390138 | down |
| COA7 | 4.13E-07 | 2.8381348 | down |
| NT5C3B | 3.23E-07 | 2.8359926 | down |
| lnc-AEBP1-1 | 3.46E-07 | 2.835592 | down |
| TRERF1 | 3.18E-07 | 2.8351305 | down |
| EXOC7 | 1.07E-07 | 2.82981 | down |
| CALML4 | 1.23E-06 | 2.8296025 | down |
| HMGN5 | 5.53E-07 | 2.8251896 | down |
| NR2C2AP | 8.57E-07 | 2.8219686 | down |
| SNHG12 | 9.50E-08 | 2.815954 | down |
| UBAP2L | 9.25E-07 | 2.813762 | down |
| MPC2 | 1.95E-07 | 2.8130753 | down |
| BACE2 | 7.65E-07 | 2.8129628 | down |
| SLC9A5 | 1.92E-07 | 2.8125317 | down |
| CKLF | 1.04E-07 | 2.8124213 | down |
| MSI2 | 5.16E-07 | 2.8116634 | down |
| MFSD11 | 1.43E-07 | 2.81155 | down |
| MPP1 | 4.77E-07 | 2.8112016 | down |
| VDR | 3.98E-07 | 2.8104973 | down |
| SLC26A11 | 6.44E-07 | 2.8099675 | down |
| GUK1 | 1.43E-07 | 2.8064022 | down |
| UBE2T | 2.79E-07 | 2.8060808 | down |
| HS3ST1 | 1.12E-06 | 2.804102 | down |
| RNF166 | 4.05E-07 | 2.8030086 | down |
| CBX8 | 2.61E-07 | 2.8027992 | down |
| PHLDA3 | 3.05E-07 | 2.8027303 | down |
| GEMIN5 | 3.16E-07 | 2.801038 | down |
| PRSS16 | 1.04E-06 | 2.8007948 | down |
| GINS2 | 2.93E-07 | 2.798236 | down |
| NUP98 | 4.97E-07 | 2.7963686 | down |
| KCNJ8 | 1.62E-07 | 2.7959607 | down |
| SELENBP1 | 4.95E-07 | 2.7947292 | down |
| KRT8 | 6.41E-07 | 2.7763178 | down |
| LOC100131626 | 1.32E-06 | 2.7754018 | down |
| ISCU | 9.11E-07 | 2.772119 | down |
| ATP6V1C1 | 1.22E-07 | 2.771664 | down |
| ITGA4 | 1.27E-06 | 2.770913 | down |
| ADAP2 | 7.82E-07 | 2.7697642 | down |
| CAV3 | 8.06E-07 | 2.769074 | down |
| LOC399900 | 3.86E-07 | 2.759343 | down |
| PRR13 | 1.38E-07 | 2.7565978 | down |
| HPS1 | 1.24E-07 | 2.751899 | down |
| STAG3 | 6.43E-07 | 2.751406 | down |
| ALYREF | 2.15E-07 | 2.7485356 | down |
| SMG5 | 4.95E-07 | 2.747933 | down |
| PSMB8 | 2.04E-07 | 2.7435265 | down |
| FOXK2 | 7.10E-07 | 2.7429438 | down |
| IFIT1 | 8.64E-07 | 2.7405732 | down |
| COL16A1 | 1.40E-07 | 2.7391982 | down |
| GPD2 | 4.68E-07 | 2.7385428 | down |
| NAT10 | 1.51E-07 | 2.7328537 | down |
| CCDC102A | 9.67E-07 | 2.7303658 | down |
| BLOC1S2 | 2.47E-07 | 2.728631 | down |
| KLHDC4 | 3.61E-07 | 2.7238872 | down |
| PFDN2 | 4.72E-07 | 2.723407 | down |
| FAM156B | 9.99E-07 | 2.7226264 | down |
| MND1 | 9.29E-07 | 2.7209184 | down |
| SOAT1 | 4.09E-07 | 2.7204819 | down |
| ATP6V0E2-AS1 | 1.36E-06 | 2.7160707 | down |
| CENPN | 4.74E-07 | 2.7147117 | down |
| ADIPOR1 | 5.85E-07 | 2.711788 | down |
| ORAI1 | 1.04E-06 | 2.7103984 | down |
| PMF1 | 3.95E-07 | 2.710222 | down |
| HSPA14 | 1.45E-07 | 2.7078032 | down |
| CENPL | 3.76E-07 | 2.7054107 | down |
| ARHGAP28 | 2.67E-07 | 2.7042263 | down |
| POLR2H | 8.56E-07 | 2.703854 | down |
| NENF | 4.89E-07 | 2.7003987 | down |
| PTRH2 | 1.92E-07 | 2.6997778 | down |
| FAM66A | 5.11E-07 | 2.6928866 | down |
| NLRC5 | 2.68E-07 | 2.6911895 | down |
| UBQLN4 | 1.33E-06 | 2.6911492 | down |
| AKAP1 | 9.62E-07 | 2.6884947 | down |
| CKS1B | 1.06E-06 | 2.682604 | down |
| METTL13 | 3.76E-07 | 2.6810424 | down |
| LOC340335 | 9.74E-07 | 2.6803496 | down |
| LSM6 | 1.52E-07 | 2.6757526 | down |
| B4GALT3 | 2.32E-07 | 2.6755133 | down |
| PLA2G16 | 8.03E-07 | 2.6724753 | down |
| FTL | 3.67E-07 | 2.671787 | down |
| PTBP2 | 6.00E-07 | 2.668419 | down |
| PRR11 | 7.54E-07 | 2.665701 | down |
| TRAM1 | 3.99E-07 | 2.6638253 | down |
| LSP1 | 5.79E-07 | 2.663609 | down |
| NEK11 | 4.00E-07 | 2.6608152 | down |
| SOD2 | 3.93E-07 | 2.6605809 | down |
| ZNF44 | 4.35E-07 | 2.6578603 | down |
| DCAF13P3 | 9.80E-07 | 2.6572928 | down |
| FAH | 2.36E-07 | 2.6561606 | down |
| UBAP2L | 4.88E-07 | 2.6544142 | down |
| CCDC137 | 1.15E-06 | 2.652307 | down |
| CD3EAP | 7.36E-07 | 2.651685 | down |
| MPZL1 | 3.01E-07 | 2.651038 | down |
| DYNLL2 | 3.76E-07 | 2.6497982 | down |
| TRNP1 | 4.61E-07 | 2.649407 | down |
| PTDSS1 | 2.08E-07 | 2.6488192 | down |
| TROAP | 3.86E-07 | 2.6454282 | down |
| COIL | 1.21E-07 | 2.644519 | down |
| FAM50B | 3.56E-07 | 2.6430182 | down |
| HIST1H4C | 7.71E-07 | 2.640355 | down |
| DNAJC19 | 5.50E-07 | 2.6398573 | down |
| SLC3A2 | 5.34E-07 | 2.6347477 | down |
| HOXC8 | 1.55E-07 | 2.6339087 | down |
| ANKRD27 | 8.54E-07 | 2.6314013 | down |
| MRPS7 | 4.10E-07 | 2.6298406 | down |
| ARMCX6 | 5.67E-07 | 2.6284995 | down |
| FOXK2 | 2.10E-07 | 2.6265135 | down |
| IMP4 | 3.14E-07 | 2.621225 | down |
| LRRC75A | 6.97E-07 | 2.6205044 | down |
| C11orf1 | 5.06E-07 | 2.6173255 | down |
| NOB1 | 9.76E-07 | 2.6149948 | down |
| POLR3G | 4.82E-07 | 2.6126904 | down |
| SLC16A4 | 2.60E-07 | 2.6101964 | down |
| IGFBP6 | 2.44E-07 | 2.6048229 | down |
| DHX34 | 2.93E-07 | 2.6019807 | down |
| DNPEP | 4.67E-07 | 2.5994124 | down |
| DYRK3 | 3.65E-07 | 2.599117 | down |
| FAM216A | 1.13E-06 | 2.598718 | down |
| CERS6 | 1.13E-06 | 2.594613 | down |
| ADAMTS12 | 9.50E-07 | 2.5943558 | down |
| C20orf24 | 1.90E-07 | 2.5919342 | down |
| MRPL24 | 1.99E-07 | 2.5877392 | down |
| B4GALT3 | 1.00E-06 | 2.5870764 | down |
| TMEM136 | 5.64E-07 | 2.586901 | down |
| CKS1B | 1.67E-07 | 2.5855803 | down |
| DCAF13 | 2.32E-07 | 2.5825877 | down |
| COA3 | 9.46E-07 | 2.5818794 | down |
| STC2 | 1.01E-06 | 2.5818384 | down |
| GNAS-AS1 | 6.90E-07 | 2.580258 | down |
| PDHX | 1.72E-07 | 2.5763123 | down |
| PSMB10 | 9.75E-07 | 2.5731843 | down |
| CXorf40B | 2.33E-07 | 2.5725036 | down |
| VMA21 | 9.69E-07 | 2.569525 | down |
| BCCIP | 3.43E-07 | 2.569512 | down |
| DSCC1 | 5.57E-07 | 2.569202 | down |
| TFB2M | 9.12E-07 | 2.569173 | down |
| CMC2 | 3.44E-07 | 2.5691218 | down |
| POP1 | 2.23E-07 | 2.5678167 | down |
| CREM | 9.68E-07 | 2.5662477 | down |
| CXorf40B | 1.16E-06 | 2.5651324 | down |
| GRB2 | 2.57E-07 | 2.5624886 | down |
| MRGBP | 1.48E-07 | 2.5597444 | down |
| TNS3 | 8.03E-07 | 2.5590963 | down |
| DNAH14 | 1.33E-07 | 2.5585737 | down |
| IQSEC2 | 4.44E-07 | 2.5570953 | down |
| AVPI1 | 8.40E-07 | 2.5560045 | down |
| LYAR | 2.16E-07 | 2.552293 | down |
| MRPS33 | 8.16E-07 | 2.55131 | down |
| SCARA3 | 1.14E-06 | 2.5502968 | down |
| C1orf43 | 4.02E-07 | 2.5478826 | down |
| LOC388242 | 1.30E-06 | 2.53966 | down |
| BPNT1 | 6.40E-07 | 2.5368829 | down |
| CYC1 | 4.51E-07 | 2.5368183 | down |
| LOC730102 | 1.22E-06 | 2.5359354 | down |
| MIF4GD | 4.47E-07 | 2.5348384 | down |
| FCRLB | 2.16E-07 | 2.5309446 | down |
| CSNK2A2 | 6.92E-07 | 2.5261097 | down |
| BOLA1 | 1.16E-06 | 2.525464 | down |
| ISOC1 | 2.46E-07 | 2.52281 | down |
| C11orf70 | 4.54E-07 | 2.5213163 | down |
| TIGD2 | 3.18E-07 | 2.5212924 | down |
| HKDC1 | 3.59E-07 | 2.518596 | down |
| CSNK1D | 2.71E-07 | 2.5169942 | down |
| COPS7B | 1.41E-06 | 2.5158703 | down |
| AP1S3 | 7.59E-07 | 2.5102715 | down |
| KIAA0101 | 3.20E-07 | 2.5056648 | down |
| PRADC1 | 1.24E-06 | 2.5009758 | down |
| POLM | 7.63E-07 | 2.4972866 | down |
| LRRC20 | 9.56E-07 | 2.497013 | down |
| TRMT2B | 6.14E-07 | 2.4960136 | down |
| C11orf68 | 3.60E-07 | 2.4935713 | down |
| SLC30A1 | 5.80E-07 | 2.49281 | down |
| METTL21A | 1.13E-06 | 2.4894984 | down |
| RAB35 | 2.32E-07 | 2.4887013 | down |
| UCHL5 | 6.76E-07 | 2.4867134 | down |
| METTL12 | 6.34E-07 | 2.4858878 | down |
| PYCRL | 4.71E-07 | 2.485483 | down |
| XAGE3 | 3.63E-07 | 2.4825442 | down |
| FDXR | 3.24E-07 | 2.482447 | down |
| TCOF1 | 5.62E-07 | 2.4817665 | down |
| ERLIN1 | 6.97E-07 | 2.4814222 | down |
| BNIP3 | 2.74E-07 | 2.4798176 | down |
| GUK1 | 8.22E-07 | 2.4793463 | down |
| HIST1H2AB | 1.04E-06 | 2.4778936 | down |
| ADI1 | 1.16E-06 | 2.476417 | down |
| BPNT1 | 1.03E-06 | 2.474204 | down |
| FAM131A | 4.47E-07 | 2.4668043 | down |
| LSM10 | 1.93E-07 | 2.4664323 | down |
| MORN4 | 2.26E-07 | 2.4661725 | down |
| USP46-AS1 | 1.41E-06 | 2.4633183 | down |
| CHCHD7 | 5.83E-07 | 2.4587753 | down |
| PYCR1 | 7.89E-07 | 2.4582503 | down |
| CHPT1 | 1.45E-06 | 2.4579592 | down |
| RPRD2 | 9.59E-07 | 2.4557793 | down |
| EXOSC5 | 8.12E-07 | 2.4555073 | down |
| HSPA1B | 8.22E-07 | 2.452901 | down |
| MTX1 | 3.19E-07 | 2.4497297 | down |
| TSPAN33 | 2.39E-07 | 2.4428635 | down |
| PPP1CC | 1.11E-06 | 2.4423285 | down |
| TMEM116 | 1.11E-06 | 2.4411376 | down |
| C1orf43 | 4.10E-07 | 2.437909 | down |
| TMCO4 | 1.13E-06 | 2.437807 | down |
| PLIN2 | 7.18E-07 | 2.4370697 | down |
| HERC4 | 3.41E-07 | 2.4370098 | down |
| C17orf62 | 6.29E-07 | 2.4370062 | down |
| PIN4 | 8.75E-07 | 2.436235 | down |
| LINC00482 | 2.70E-07 | 2.4329743 | down |
| HYLS1 | 7.79E-07 | 2.4300303 | down |
| TBC1D2 | 7.54E-07 | 2.4291048 | down |
| C12orf4 | 6.40E-07 | 2.4281304 | down |
| CD47 | 3.30E-07 | 2.4197721 | down |
| TFEB | 4.88E-07 | 2.4135075 | down |
| APEX2 | 2.23E-07 | 2.412436 | down |
| SLC25A28 | 7.14E-07 | 2.4095101 | down |
| CYTL1 | 1.39E-06 | 2.4080846 | down |
| PRTFDC1 | 3.32E-07 | 2.4054532 | down |
| GALK1 | 1.04E-06 | 2.4012485 | down |
| RCOR3 | 1.29E-06 | 2.400455 | down |
| GPS1 | 3.97E-07 | 2.400061 | down |
| LOC101929494 | 3.08E-07 | 2.3997319 | down |
| ARHGAP23 | 7.40E-07 | 2.3995848 | down |
| MRPL3 | 6.61E-07 | 2.3990233 | down |
| MET | 6.36E-07 | 2.3979535 | down |
| IDS | 7.31E-07 | 2.3970456 | down |
| APOA1BP | 4.30E-07 | 2.3967443 | down |
| DAGLB | 1.44E-06 | 2.3956482 | down |
| DTL | 2.82E-07 | 2.3946338 | down |
| C8orf59 | 6.02E-07 | 2.3918161 | down |
| TIPIN | 5.27E-07 | 2.3917146 | down |
| ARHGAP23 | 4.20E-07 | 2.390567 | down |
| CCNE1 | 1.13E-06 | 2.3891668 | down |
| MDM4 | 6.20E-07 | 2.3825064 | down |
| CIRH1A | 6.38E-07 | 2.3774962 | down |
| SORL1 | 1.15E-06 | 2.376103 | down |
| DCAF13 | 1.07E-06 | 2.3729959 | down |
| ISG20L2 | 5.79E-07 | 2.3728786 | down |
| CDCA3 | 3.29E-07 | 2.37204 | down |
| DEF8 | 6.64E-07 | 2.3712823 | down |
| GNPDA2 | 6.29E-07 | 2.3703127 | down |
| HEXIM2 | 7.45E-07 | 2.3685634 | down |
| COX15 | 2.54E-07 | 2.3684204 | down |
| CERS2 | 1.46E-06 | 2.3671734 | down |
| BAG2 | 1.05E-06 | 2.3669345 | down |
| METTL1 | 4.72E-07 | 2.3665478 | down |
| PPP1R16A | 2.47E-07 | 2.3658888 | down |
| PQLC2L | 8.72E-07 | 2.365785 | down |
| RITA1 | 4.76E-07 | 2.3656304 | down |
| SLC50A1 | 6.49E-07 | 2.3653307 | down |
| ASPSCR1 | 4.35E-07 | 2.3598387 | down |
| GPER1 | 1.44E-06 | 2.3592896 | down |
| HPRT1 | 7.50E-07 | 2.358742 | down |
| PRAME | 9.17E-07 | 2.3552387 | down |
| METTL2A | 1.21E-06 | 2.3547165 | down |
| CAPZA2 | 1.19E-06 | 2.3537405 | down |
| CENPE | 6.24E-07 | 2.352118 | down |
| SMAD9 | 1.15E-06 | 2.352097 | down |
| BMI1 | 5.73E-07 | 2.3514938 | down |
| RAB21 | 3.13E-07 | 2.3500562 | down |
| HSPE1 | 1.33E-06 | 2.3476248 | down |
| COA4 | 1.08E-06 | 2.3468926 | down |
| RHEB | 1.30E-06 | 2.3442864 | down |
| TERT | 5.24E-07 | 2.3442144 | down |
| COX17 | 9.47E-07 | 2.3436687 | down |
| ALDH3B1 | 1.33E-06 | 2.3435466 | down |
| METTL1 | 1.03E-06 | 2.342477 | down |
| PDE4A | 3.09E-07 | 2.3372085 | down |
| TIPARP | 4.82E-07 | 2.3356428 | down |
| SCG5 | 1.32E-06 | 2.333146 | down |
| ZNF692 | 7.91E-07 | 2.3300395 | down |
| NUTM2G | 5.99E-07 | 2.323434 | down |
| TCEB1 | 6.24E-07 | 2.3197815 | down |
| TLR3 | 9.84E-07 | 2.3195431 | down |
| DDX28 | 2.75E-07 | 2.317981 | down |
| PRIM1 | 8.62E-07 | 2.3164098 | down |
| SHC1 | 2.59E-07 | 2.314702 | down |
| NLE1 | 7.00E-07 | 2.3146563 | down |
| KANSL2 | 4.66E-07 | 2.3130202 | down |
| POLDIP2 | 5.78E-07 | 2.3118975 | down |
| C7orf49 | 6.32E-07 | 2.3114607 | down |
| IER5 | 9.16E-07 | 2.3104014 | down |
| KLHDC10 | 8.62E-07 | 2.3091173 | down |
| DCLRE1A | 1.01E-06 | 2.3066869 | down |
| TMEM167A | 1.01E-06 | 2.3065376 | down |
| STK3 | 5.14E-07 | 2.3050983 | down |
| VEGFC | 6.25E-07 | 2.3032913 | down |
| PSMD11 | 1.27E-06 | 2.3003187 | down |
| CHPF2 | 8.19E-07 | 2.2972598 | down |
| CKLF | 4.13E-07 | 2.2957249 | down |
| ASH1L-AS1 | 8.87E-07 | 2.2914908 | down |
| BET1L | 7.14E-07 | 2.289172 | down |
| NFATC1 | 3.17E-07 | 2.2874725 | down |
| DEDD | 1.24E-06 | 2.2870493 | down |
| H2AFJ | 8.32E-07 | 2.2854254 | down |
| FAM27C | 4.08E-07 | 2.2851343 | down |
| CYB5R1 | 4.10E-07 | 2.284824 | down |
| KAT2A | 1.01E-06 | 2.2824214 | down |
| EXOC7 | 6.69E-07 | 2.2764742 | down |
| A1BG | 1.02E-06 | 2.2727876 | down |
| SLC25A16 | 8.49E-07 | 2.2666333 | down |
| AMOTL2 | 7.90E-07 | 2.2659533 | down |
| HEATR1 | 1.33E-06 | 2.2646737 | down |
| PSEN2 | 1.15E-06 | 2.263591 | down |
| TOMM7 | 1.44E-06 | 2.262905 | down |
| EMP1 | 1.42E-06 | 2.260079 | down |
| GPN3 | 1.09E-06 | 2.2591414 | down |
| SLC25A37 | 9.17E-07 | 2.257747 | down |
| C11orf31 | 6.81E-07 | 2.2563894 | down |
| RGS10 | 5.15E-07 | 2.2507463 | down |
| ERCC8 | 1.18E-06 | 2.2503202 | down |
| LITAF | 9.82E-07 | 2.249772 | down |
| TOMM40 | 3.48E-07 | 2.2477067 | down |
| FASTK | 8.70E-07 | 2.2468734 | down |
| RDM1 | 4.38E-07 | 2.2461023 | down |
| TARBP2 | 3.26E-07 | 2.2453353 | down |
| RALBP1 | 7.24E-07 | 2.245066 | down |
| PSMD12 | 5.78E-07 | 2.2372622 | down |
| SUV39H1 | 1.00E-06 | 2.2370255 | down |
| SSSCA1 | 1.32E-06 | 2.235285 | down |
| CYHR1 | 5.96E-07 | 2.2307422 | down |
| HKDC1 | 7.31E-07 | 2.2304163 | down |
| C10orf2 | 9.92E-07 | 2.2302544 | down |
| PPM1F | 6.68E-07 | 2.2301328 | down |
| L1CAM | 5.58E-07 | 2.2287307 | down |
| PMVK | 3.21E-07 | 2.2269099 | down |
| CWF19L1 | 5.16E-07 | 2.2257013 | down |
| SARDH | 1.46E-06 | 2.225543 | down |
| DNAJC19 | 5.06E-07 | 2.2223785 | down |
| TOMM40 | 1.01E-06 | 2.2208774 | down |
| XLOC_l2_001559 | 1.09E-06 | 2.2208524 | down |
| ARFIP2 | 5.85E-07 | 2.2202206 | down |
| RAD1 | 7.82E-07 | 2.2197013 | down |
| RAB28 | 1.12E-06 | 2.2136831 | down |
| CDCA5 | 4.99E-07 | 2.2099617 | down |
| P3H2 | 6.49E-07 | 2.2089925 | down |
| ITGB1BP1 | 5.42E-07 | 2.207557 | down |
| LPAR1 | 8.87E-07 | 2.2070856 | down |
| TOR3A | 6.71E-07 | 2.2069354 | down |
| AURKAPS1 | 8.61E-07 | 2.206454 | down |
| VAMP4 | 7.44E-07 | 2.2060595 | down |
| NOP14 | 4.14E-07 | 2.198059 | down |
| IRS2 | 1.04E-06 | 2.1979775 | down |
| LOC93622 | 7.20E-07 | 2.1976106 | down |
| BOD1 | 8.02E-07 | 2.192815 | down |
| GRINA | 9.84E-07 | 2.192138 | down |
| PIP4K2B | 1.01E-06 | 2.191612 | down |
| C10orf76 | 5.78E-07 | 2.19158 | down |
| ADRM1 | 1.14E-06 | 2.1909688 | down |
| BAIAP2 | 1.11E-06 | 2.1906173 | down |
| ZFYVE27 | 8.56E-07 | 2.187004 | down |
| PLA2G15 | 6.26E-07 | 2.186435 | down |
| LOC646762 | 4.17E-07 | 2.1818302 | down |
| ARL2BP | 7.83E-07 | 2.180062 | down |
| SLC25A44 | 1.03E-06 | 2.1800318 | down |
| KCNK1 | 1.24E-06 | 2.1764677 | down |
| IMPDH1 | 1.10E-06 | 2.1741228 | down |
| ABCF3 | 1.42E-06 | 2.1735945 | down |
| EMC8 | 9.23E-07 | 2.1728327 | down |
| RPS6KC1 | 9.77E-07 | 2.1709917 | down |
| FNDC3B | 8.47E-07 | 2.169405 | down |
| SLC10A3 | 8.45E-07 | 2.1691937 | down |
| MRPS12 | 1.02E-06 | 2.1675382 | down |
| HSPB11 | 9.49E-07 | 2.167378 | down |
| MAFG | 8.58E-07 | 2.1669788 | down |
| HMOX2 | 7.61E-07 | 2.1660016 | down |
| ALG8 | 1.14E-06 | 2.164119 | down |
| POLR3E | 1.08E-06 | 2.1640182 | down |
| THAP11 | 4.78E-07 | 2.1622148 | down |
| RRP8 | 1.18E-06 | 2.1611714 | down |
| PIM2 | 1.05E-06 | 2.160243 | down |
| B9D2 | 6.80E-07 | 2.1584902 | down |
| SNX8 | 9.71E-07 | 2.158258 | down |
| FAM195A | 4.72E-07 | 2.1513052 | down |
| ALKBH2 | 6.89E-07 | 2.1471522 | down |
| RPL39L | 1.04E-06 | 2.1468034 | down |
| FAM27C | 6.27E-07 | 2.1446345 | down |
| TMEM39B | 7.15E-07 | 2.1407337 | down |
| USB1 | 1.14E-06 | 2.1397421 | down |
| PLAU | 1.18E-06 | 2.1375494 | down |
| SMUG1 | 6.08E-07 | 2.1349194 | down |
| MAP7D3 | 6.13E-07 | 2.133205 | down |
| CCDC96 | 1.20E-06 | 2.1330094 | down |
| TPRA1 | 8.85E-07 | 2.1326551 | down |
| EXOSC4 | 5.88E-07 | 2.129417 | down |
| ZWINT | 8.43E-07 | 2.1285079 | down |
| HAUS7 | 7.71E-07 | 2.1246767 | down |
| HIST2H3D | 1.39E-06 | 2.1218977 | down |
| HIST1H2BJ | 1.29E-06 | 2.121685 | down |
| MALSU1 | 8.03E-07 | 2.1141784 | down |
| SNAP47 | 7.08E-07 | 2.1141229 | down |
| LOC158435 | 5.65E-07 | 2.1121042 | down |
| BCAP29 | 8.29E-07 | 2.1074998 | down |
| EBPL | 8.10E-07 | 2.106594 | down |
| MCFD2 | 6.33E-07 | 2.1061444 | down |
| DCAF12L2 | 7.60E-07 | 2.1028447 | down |
| FAM129A | 6.33E-07 | 2.0995064 | down |
| FN3KRP | 9.39E-07 | 2.0994453 | down |
| POLG2 | 1.22E-06 | 2.0912476 | down |
| PPAT | 5.54E-07 | 2.0893877 | down |
| NOC2L | 5.14E-07 | 2.085881 | down |
| WDR74 | 9.31E-07 | 2.084362 | down |
| BCL7B | 1.07E-06 | 2.0823908 | down |
| SLC35A2 | 7.81E-07 | 2.0822542 | down |
| VDAC1 | 1.35E-06 | 2.0822523 | down |
| LOC648987 | 1.21E-06 | 2.0813072 | down |
| TMEM201 | 1.27E-06 | 2.0805554 | down |
| CLTB | 1.15E-06 | 2.079056 | down |
| NOTCH2 | 1.01E-06 | 2.0772824 | down |
| PCID2 | 1.31E-06 | 2.0697272 | down |
| PDE4DIP | 8.67E-07 | 2.0664535 | down |
| NQO2 | 1.18E-06 | 2.0626488 | down |
| SAP30BP | 8.16E-07 | 2.0622208 | down |
| RPP40 | 1.31E-06 | 2.0605922 | down |
| COQ9 | 9.90E-07 | 2.0570705 | down |
| KLHL36 | 1.01E-06 | 2.0556324 | down |
| ICAM3 | 7.88E-07 | 2.0450277 | down |
| ZDHHC9 | 9.85E-07 | 2.0431492 | down |
| RNF135 | 1.09E-06 | 2.036111 | down |
| WDR3 | 1.12E-06 | 2.0323043 | down |
| ZNF511 | 8.19E-07 | 2.027623 | down |
| EBPL | 7.97E-07 | 2.0259852 | down |
| CTBP1 | 8.21E-07 | 2.0245104 | down |
| C1orf35 | 8.28E-07 | 2.0244346 | down |
| FLAD1 | 1.06E-06 | 2.0230527 | down |
| DIMT1 | 1.03E-06 | 2.0173717 | down |
| ARL4A | 1.21E-06 | 2.0069852 | down |
| ATAD2 | 1.31E-06 | 2.005915 | down |
| RPS4Y1 | 2.37E-11 | 7098.2056 | up |
| RPS4Y2 | 9.04E-09 | 5582.307 | up |
| FBLIM1 | 1.51E-10 | 2612.0752 | up |
| TMEM98 | 4.70E-08 | 2224.5547 | up |
| PAX2 | 2.54E-11 | 2169.9333 | up |
| DMKN | 1.73E-10 | 1990.9258 | up |
| CDH11 | 4.36E-11 | 1886.6547 | up |
| CCND2 | 6.03E-08 | 1485.8367 | up |
| IGFBP5 | 1.37E-08 | 1296.1914 | up |
| LRRC17 | 2.09E-11 | 1147.7485 | up |
| FAM83G | 2.50E-08 | 1137.3861 | up |
| THY1 | 9.21E-11 | 1122.9631 | up |
| SOX11 | 1.21E-11 | 1017.1942 | up |
| CNN1 | 2.43E-09 | 931.7241 | up |
| AEBP1 | 4.30E-11 | 858.2026 | up |
| NEFH | 5.32E-10 | 713.5673 | up |
| AUTS2 | 5.10E-11 | 677.8362 | up |
| CELF2 | 4.81E-11 | 638.9432 | up |
| NKAIN4 | 1.78E-07 | 633.0603 | up |
| IGFBP2 | 6.09E-10 | 537.85394 | up |
| KIF1A | 7.90E-10 | 534.06836 | up |
| EMX2 | 9.66E-11 | 518.46497 | up |
| LRFN4 | 1.80E-08 | 468.8585 | up |
| BMP7 | 4.17E-09 | 467.11618 | up |
| NTN1 | 5.50E-10 | 465.32315 | up |
| RBP1 | 9.09E-11 | 462.2555 | up |
| PDPN | 3.18E-11 | 456.28058 | up |
| ABAT | 8.34E-11 | 442.66852 | up |
| NEFM | 6.04E-09 | 436.01566 | up |
| DDX3Y | 2.47E-11 | 424.61063 | up |
| HS3ST3B1 | 2.82E-11 | 419.8721 | up |
| SERP2 | 1.83E-08 | 378.17398 | up |
| PNOC | 1.25E-08 | 370.04895 | up |
| EMX2OS | 1.71E-10 | 351.79413 | up |
| NCAM1 | 3.14E-11 | 346.8767 | up |
| GREM1 | 3.55E-10 | 340.3599 | up |
| MYO1D | 2.38E-10 | 335.17175 | up |
| SNHG18 | 8.78E-11 | 326.0694 | up |
| GSTT1 | 5.89E-08 | 316.5134 | up |
| HS3ST3A1 | 2.22E-11 | 314.4561 | up |
| LTB | 8.34E-08 | 313.70618 | up |
| DMKN | 1.15E-06 | 303.36548 | up |
| CDKN2A | 4.27E-07 | 303.13675 | up |
| GALC | 8.70E-11 | 301.21857 | up |
| NPPB | 6.38E-10 | 292.4666 | up |
| HS6ST2 | 9.55E-11 | 278.43997 | up |
| CYP27A1 | 2.27E-10 | 270.9562 | up |
| IRX2 | 1.20E-07 | 241.90948 | up |
| IRX1 | 9.41E-11 | 234.12361 | up |
| NPPB | 1.71E-09 | 229.57861 | up |
| ALPK3 | 1.74E-08 | 228.03294 | up |
| HLA-DQB1 | 8.68E-11 | 227.88194 | up |
| CLEC18B | 1.81E-11 | 219.80841 | up |
| PDLIM4 | 6.80E-10 | 209.65633 | up |
| SRSF12 | 2.29E-10 | 208.559 | up |
| MFAP2 | 3.19E-08 | 194.8526 | up |
| KCNJ15 | 5.71E-11 | 188.39659 | up |
| DMRT3 | 2.12E-10 | 163.6612 | up |
| ZNF667-AS1 | 7.55E-10 | 158.13817 | up |
| NKAIN4 | 2.71E-10 | 155.62561 | up |
| IRX4 | 7.87E-10 | 149.71037 | up |
| COL1A1 | 4.69E-10 | 149.64354 | up |
| PROM1 | 1.68E-10 | 147.77289 | up |
| SEMA5A | 2.98E-09 | 146.12418 | up |
| EPPK1 | 1.09E-08 | 144.66664 | up |
| MAFB | 3.34E-10 | 143.96751 | up |
| CLDN6 | 1.48E-09 | 140.44955 | up |
| BEND5 | 1.45E-10 | 137.637 | up |
| NLRP2 | 1.19E-06 | 134.32175 | up |
| KCNJ12 | 7.36E-07 | 132.38864 | up |
| DSP | 5.95E-10 | 130.77077 | up |
| DIRAS2 | 7.09E-10 | 129.30058 | up |
| CTHRC1 | 7.94E-10 | 127.76428 | up |
| NEDD9 | 6.57E-10 | 123.91684 | up |
| TENM2 | 6.80E-09 | 120.24336 | up |
| JAM3 | 5.82E-09 | 120.20915 | up |
| KLHDC8A | 1.15E-06 | 119.15476 | up |
| SBK1 | 1.88E-08 | 115.79462 | up |
| TSHZ3 | 2.34E-10 | 115.56556 | up |
| TENM2 | 1.78E-09 | 106.30447 | up |
| COL1A2 | 4.08E-09 | 105.54672 | up |
| USP9Y | 2.04E-10 | 104.09145 | up |
| ACTG2 | 5.72E-10 | 103.83196 | up |
| DSP | 6.46E-09 | 103.64646 | up |
| SORCS3 | 9.16E-08 | 102.89075 | up |
| CELF2 | 4.12E-10 | 101.11675 | up |
| SDR42E1 | 5.57E-07 | 99.05444 | up |
| DCDC2 | 3.97E-11 | 97.89436 | up |
| TTTY15 | 3.44E-07 | 97.57373 | up |
| LAD1 | 7.85E-10 | 94.79162 | up |
| FAM110C | 5.91E-10 | 93.70035 | up |
| TMEM25 | 1.39E-08 | 90.997215 | up |
| CCDC8 | 1.18E-07 | 90.97875 | up |
| NRG3 | 2.68E-10 | 90.9366 | up |
| TCEAL7 | 7.61E-08 | 89.5128 | up |
| ACTA1 | 4.04E-08 | 88.86904 | up |
| EDIL3 | 2.93E-08 | 84.88338 | up |
| ZNF521 | 4.95E-10 | 84.01901 | up |
| BEX5 | 4.08E-10 | 83.80385 | up |
| MUM1L1 | 1.35E-09 | 82.412506 | up |
| ZFP42 | 4.33E-08 | 82.14589 | up |
| BCL11A | 7.59E-10 | 79.01015 | up |
| ARHGAP44 | 6.48E-09 | 78.255646 | up |
| KCTD15 | 6.47E-09 | 78.23281 | up |
| EIF1AY | 3.84E-10 | 78.060196 | up |
| CYS1 | 5.85E-07 | 77.78025 | up |
| NRK | 1.04E-09 | 77.42688 | up |
| WNT5A | 3.22E-08 | 75.5299 | up |
| ELTD1 | 1.93E-10 | 74.74166 | up |
| GAS1 | 1.49E-08 | 74.11496 | up |
| TIMP3 | 2.68E-08 | 73.96758 | up |
| CHRDL1 | 1.01E-09 | 73.7415 | up |
| NAP1L3 | 2.96E-10 | 71.85877 | up |
| FZD8 | 1.46E-09 | 71.50736 | up |
| TTTY14 | 3.83E-10 | 68.75981 | up |
| ADAMTS5 | 5.74E-08 | 67.786194 | up |
| POSTN | 1.67E-09 | 67.48111 | up |
| ZFY | 9.30E-10 | 67.354454 | up |
| SST | 7.32E-07 | 63.497334 | up |
| IL11 | 1.79E-10 | 63.27731 | up |
| PCSK9 | 5.87E-10 | 62.19587 | up |
| SPOCD1 | 3.47E-10 | 61.627434 | up |
| KCNJ15 | 1.51E-08 | 61.1489 | up |
| COLEC12 | 3.91E-09 | 60.93492 | up |
| TNS1 | 2.51E-08 | 60.318806 | up |
| MGP | 7.58E-08 | 60.097797 | up |
| FGF13 | 5.58E-10 | 59.82646 | up |
| SLCO2A1 | 3.95E-07 | 57.59603 | up |
| SGCD | 1.26E-08 | 57.467415 | up |
| NCF2 | 1.07E-08 | 57.307175 | up |
| MTMR9LP | 2.41E-08 | 57.21043 | up |
| PRSS8 | 4.58E-09 | 56.651505 | up |
| CSPG4 | 6.73E-07 | 56.442924 | up |
| FNDC1 | 1.14E-09 | 55.574585 | up |
| ERC2 | 5.26E-09 | 55.489437 | up |
| TBX3 | 5.54E-09 | 55.381977 | up |
| NLGN4X | 4.75E-09 | 55.062588 | up |
| CFAP58 | 3.84E-09 | 54.36181 | up |
| ZNF730 | 8.20E-10 | 53.872433 | up |
| SCEL | 2.05E-09 | 53.686523 | up |
| KCTD15 | 1.15E-09 | 53.103 | up |
| NEFL | 9.52E-10 | 52.908222 | up |
| KRBOX1 | 1.36E-09 | 52.17125 | up |
| FAM155B | 7.48E-08 | 51.901344 | up |
| FBLIM1 | 1.62E-07 | 51.39726 | up |
| ITGA11 | 1.63E-09 | 50.830635 | up |
| FIGNL2 | 6.92E-10 | 50.569675 | up |
| NFATC4 | 7.02E-10 | 50.2736 | up |
| MSX2P1 | 6.09E-10 | 50.028893 | up |
| TMEM233 | 8.39E-10 | 49.077656 | up |
| RSPO4 | 1.39E-08 | 49.006413 | up |
| MGC12916 | 2.56E-10 | 48.949562 | up |
| LOXL1 | 2.50E-10 | 48.75366 | up |
| AKAP12 | 1.79E-10 | 48.640106 | up |
| EPCAM | 7.43E-10 | 48.06434 | up |
| LIMS2 | 6.33E-09 | 47.317627 | up |
| SLIT3 | 7.97E-11 | 47.03961 | up |
| DIRC3 | 4.97E-10 | 46.677498 | up |
| LBH | 6.50E-10 | 46.1565 | up |
| MDFI | 8.64E-09 | 45.938602 | up |
| KCNG1 | 9.42E-10 | 45.245785 | up |
| ZNF667-AS1 | 1.20E-08 | 44.172848 | up |
| INSR | 1.22E-08 | 43.812668 | up |
| MAF | 1.85E-08 | 43.24013 | up |
| CXADR | 2.23E-07 | 42.656673 | up |
| BMP2 | 3.03E-08 | 42.649498 | up |
| NOX4 | 1.40E-08 | 42.554184 | up |
| MGAT3 | 1.16E-09 | 41.5374 | up |
| AQP1 | 2.03E-08 | 41.458805 | up |
| FAM19A5 | 8.15E-10 | 40.032665 | up |
| C11orf96 | 3.30E-08 | 39.868904 | up |
| SCOC-AS1 | 1.32E-08 | 39.59162 | up |
| CADM1 | 4.73E-09 | 39.488937 | up |
| SLC13A3 | 2.07E-09 | 39.476933 | up |
| SERPING1 | 9.47E-09 | 38.920395 | up |
| TNC | 6.15E-09 | 38.388477 | up |
| DIRAS3 | 1.26E-08 | 38.360977 | up |
| MEIOB | 7.24E-09 | 37.57011 | up |
| AKAP12 | 2.61E-09 | 37.35043 | up |
| GABBR2 | 2.35E-10 | 37.276287 | up |
| ELTD1 | 6.40E-07 | 37.145805 | up |
| TLL1 | 1.30E-09 | 37.084473 | up |
| AUTS2 | 1.02E-08 | 36.886276 | up |
| ZNF671 | 2.24E-09 | 36.823265 | up |
| GPC4 | 3.98E-08 | 36.64364 | up |
| DCN | 4.10E-09 | 36.46373 | up |
| CYP27C1 | 1.10E-08 | 35.999947 | up |
| F2RL1 | 2.36E-09 | 35.907673 | up |
| AKAP12 | 2.28E-10 | 35.47935 | up |
| NES | 5.05E-07 | 35.469078 | up |
| REC8 | 1.31E-09 | 35.273724 | up |
| NLGN4Y | 1.49E-09 | 35.102783 | up |
| NRG3 | 9.45E-09 | 34.827606 | up |
| GALNT3 | 1.85E-08 | 34.426254 | up |
| FAM132B | 1.96E-07 | 34.30728 | up |
| F3 | 3.92E-09 | 34.30362 | up |
| COL22A1 | 1.45E-09 | 34.22094 | up |
| WNT5B | 6.56E-07 | 34.16403 | up |
| SPOCK2 | 3.96E-10 | 33.945206 | up |
| SERPING1 | 4.07E-08 | 33.761013 | up |
| CADM1 | 1.42E-07 | 33.67847 | up |
| SULT1C4 | 4.75E-09 | 33.665516 | up |
| RAP1GAP | 1.01E-07 | 33.59105 | up |
| GREM1 | 1.58E-09 | 33.170643 | up |
| SPINT1 | 1.57E-07 | 32.938213 | up |
| CNTNAP2 | 1.98E-09 | 32.796467 | up |
| CCDC8 | 4.49E-08 | 32.65702 | up |
| UCA1 | 2.36E-08 | 32.304073 | up |
| NEFH | 6.22E-09 | 32.24359 | up |
| LRFN4 | 9.06E-09 | 31.864716 | up |
| KRT17 | 6.73E-08 | 31.691067 | up |
| FAM84A | 3.72E-09 | 31.250574 | up |
| TMEM178A | 3.00E-09 | 31.197447 | up |
| CDKN2B | 1.38E-08 | 30.975576 | up |
| SCN3B | 7.20E-10 | 30.87304 | up |
| SYK | 4.29E-08 | 30.553398 | up |
| NTF3 | 2.30E-08 | 30.538769 | up |
| XKR4 | 9.42E-10 | 30.270445 | up |
| PABPC5 | 5.39E-07 | 30.008385 | up |
| IFITM1 | 2.50E-08 | 29.766722 | up |
| SSC5D | 1.81E-08 | 29.66298 | up |
| PRSS3P2 | 1.42E-08 | 29.59711 | up |
| IFITM1 | 3.63E-09 | 29.585726 | up |
| LAMC2 | 4.79E-10 | 29.50329 | up |
| HS6ST2 | 4.18E-08 | 29.299732 | up |
| KBTBD11 | 2.03E-09 | 29.247786 | up |
| FAM150B | 8.58E-09 | 28.847057 | up |
| FZD10 | 1.85E-09 | 28.817307 | up |
| SORCS3 | 8.56E-08 | 28.70984 | up |
| EMILIN2 | 1.75E-08 | 28.693277 | up |
| LCP1 | 3.14E-10 | 28.677397 | up |
| BEX1 | 4.28E-10 | 28.667276 | up |
| LOC102723456 | 1.08E-09 | 28.399529 | up |
| LYPD1 | 1.11E-09 | 28.385338 | up |
| LIMS2 | 3.99E-07 | 28.25791 | up |
| COBL | 1.44E-08 | 28.073095 | up |
| LIPG | 8.36E-08 | 27.217241 | up |
| GPR182 | 2.26E-07 | 27.092361 | up |
| RASGRF2 | 6.41E-09 | 26.770847 | up |
| SLC24A3 | 7.41E-09 | 26.463879 | up |
| HMBOX1 | 2.24E-07 | 26.33448 | up |
| MEG3 | 1.33E-08 | 26.158632 | up |
| LPHN3 | 2.85E-07 | 26.01908 | up |
| WNK4 | 7.76E-08 | 25.886688 | up |
| ALDH1A2 | 4.74E-09 | 25.806856 | up |
| FOXL2 | 6.37E-09 | 25.725544 | up |
| CKB | 1.47E-08 | 25.699257 | up |
| PSORS1C3 | 1.58E-09 | 25.411228 | up |
| KLHL30 | 3.80E-08 | 25.228664 | up |
| JAG1 | 2.08E-09 | 25.131384 | up |
| TRIM71 | 2.44E-09 | 25.122879 | up |
| ARL10 | 2.10E-09 | 25.037176 | up |
| ADAM12 | 1.32E-06 | 24.870712 | up |
| COL7A1 | 1.13E-07 | 24.804234 | up |
| LOC101928880 | 1.56E-08 | 24.742462 | up |
| UTY | 8.07E-09 | 24.609083 | up |
| TNFSF10 | 3.46E-09 | 24.36854 | up |
| PROM2 | 1.06E-06 | 24.22369 | up |
| TMEM25 | 1.88E-08 | 24.204466 | up |
| DCN | 3.64E-08 | 23.897823 | up |
| TNS1 | 8.22E-10 | 23.859894 | up |
| LYPD1 | 5.11E-09 | 23.681416 | up |
| LARGE | 1.24E-09 | 23.583193 | up |
| TMEM132D | 9.58E-09 | 23.565865 | up |
| lnc-SNX24-1 | 1.38E-06 | 23.54022 | up |
| MYO1D | 3.38E-09 | 23.26899 | up |
| TLL2 | 1.14E-08 | 23.219742 | up |
| GPR179 | 8.73E-07 | 23.1623 | up |
| ZNF469 | 7.20E-10 | 23.033876 | up |
| SEMA6A | 2.65E-08 | 22.76239 | up |
| ZBTB1 | 3.67E-07 | 22.604185 | up |
| MMP7 | 5.07E-10 | 22.556837 | up |
| XDH | 3.87E-08 | 22.465107 | up |
| CRISPLD2 | 2.68E-07 | 22.456488 | up |
| CCDC144A | 9.64E-09 | 22.244034 | up |
| LINC00667 | 4.71E-07 | 21.906572 | up |
| CECR6 | 9.00E-07 | 21.739157 | up |
| ZNF204P | 6.25E-09 | 21.569147 | up |
| TNFSF10 | 1.77E-08 | 21.521791 | up |
| CD69 | 1.10E-08 | 21.48837 | up |
| XYLT1 | 4.97E-09 | 21.425402 | up |
| LAMC2 | 3.61E-09 | 21.332157 | up |
| EGR3 | 2.09E-09 | 21.092382 | up |
| PRSS3 | 3.05E-07 | 20.905067 | up |
| HLA-DQB1 | 6.23E-08 | 20.83234 | up |
| MAOB | 4.87E-07 | 20.704685 | up |
| SYT11 | 7.06E-09 | 20.587164 | up |
| CD86 | 1.95E-07 | 20.170395 | up |
| SLC18A2 | 5.24E-08 | 20.114254 | up |
| LTBP1 | 1.01E-06 | 19.922115 | up |
| ABCA1 | 1.28E-09 | 19.871485 | up |
| PHYHD1 | 1.25E-08 | 19.778406 | up |
| ZNF521 | 1.10E-06 | 19.731337 | up |
| EPHB1 | 9.01E-08 | 19.686747 | up |
| UCN2 | 4.91E-10 | 19.67656 | up |
| EFHD1 | 2.41E-07 | 19.63051 | up |
| FLJ41170 | 5.91E-07 | 19.573587 | up |
| BCL11A | 1.38E-08 | 19.548859 | up |
| MPZL3 | 5.37E-10 | 19.461035 | up |
| HOMER2 | 6.63E-09 | 19.374054 | up |
| CRIP1 | 2.61E-07 | 19.365059 | up |
| CLEC4E | 5.94E-07 | 19.311554 | up |
| MMP1 | 4.12E-09 | 19.307596 | up |
| BMF | 2.06E-09 | 19.075384 | up |
| CLDN4 | 4.25E-09 | 18.884592 | up |
| FOXL2NB | 1.28E-07 | 18.7658 | up |
| BMP7 | 5.38E-09 | 18.495876 | up |
| ID4 | 5.62E-10 | 18.451609 | up |
| MYOZ3 | 3.22E-08 | 18.333431 | up |
| NACAD | 2.54E-09 | 18.29835 | up |
| KCNMA1 | 2.43E-07 | 18.1771 | up |
| C14orf37 | 1.87E-07 | 18.127201 | up |
| IFNLR1 | 7.82E-07 | 18.11978 | up |
| WSCD1 | 3.99E-09 | 18.02444 | up |
| PIK3R5 | 1.43E-07 | 17.93201 | up |
| FOXS1 | 2.70E-08 | 17.856167 | up |
| KIF5A | 1.10E-07 | 17.80519 | up |
| SPANXN3 | 3.02E-08 | 17.725937 | up |
| HLA-DQA1 | 5.22E-09 | 17.653519 | up |
| SOCS2 | 1.99E-09 | 17.578514 | up |
| GLIPR2 | 9.21E-09 | 17.54319 | up |
| PWAR1 | 2.07E-07 | 17.456892 | up |
| ZNF439 | 1.01E-08 | 17.413874 | up |
| MDK | 2.40E-09 | 17.393423 | up |
| GPR143 | 4.93E-09 | 17.367613 | up |
| PSG2 | 1.06E-08 | 17.341269 | up |
| PDE4D | 1.52E-08 | 17.309898 | up |
| REP15 | 2.71E-07 | 17.29232 | up |
| LOC101929056 | 2.53E-07 | 17.274218 | up |
| TMEM30B | 3.99E-08 | 17.225899 | up |
| LHX1 | 1.95E-09 | 17.161444 | up |
| GALC | 1.06E-08 | 16.891987 | up |
| RASIP1 | 7.63E-07 | 16.855803 | up |
| NSUN7 | 4.10E-09 | 16.80503 | up |
| LPAR5 | 2.91E-07 | 16.80006 | up |
| CDK15 | 1.23E-08 | 16.629013 | up |
| FUT9 | 1.17E-07 | 16.447298 | up |
| TUBB2B | 1.47E-09 | 16.319698 | up |
| CHST1 | 2.00E-09 | 16.315617 | up |
| PCDHB5 | 4.14E-08 | 16.234713 | up |
| LOC101928076 | 4.05E-08 | 16.128948 | up |
| CFTR | 1.13E-08 | 16.11975 | up |
| DYSF | 8.25E-08 | 16.076689 | up |
| PTPRN2 | 5.35E-09 | 16.06488 | up |
| MISP | 6.22E-08 | 16.044643 | up |
| STMN2 | 3.23E-08 | 15.991274 | up |
| NTNG1 | 9.95E-09 | 15.920088 | up |
| lnc-C11orf39-3 | 3.24E-09 | 15.915142 | up |
| MYCL | 2.89E-09 | 15.850955 | up |
| SPEN | 6.63E-07 | 15.791183 | up |
| FXYD6 | 1.11E-07 | 15.788856 | up |
| SEMA7A | 6.34E-08 | 15.712286 | up |
| CPXM2 | 4.79E-09 | 15.608398 | up |
| RCOR2 | 7.58E-07 | 15.431734 | up |
| ZNF135 | 5.31E-07 | 15.371077 | up |
| C14orf37 | 4.43E-09 | 15.284225 | up |
| SPNS2 | 1.16E-08 | 15.223802 | up |
| ZBTB46 | 7.45E-08 | 15.167778 | up |
| CYP27C1 | 2.13E-08 | 15.060839 | up |
| DNAJA4 | 2.37E-09 | 14.830475 | up |
| AIF1L | 6.31E-10 | 14.829585 | up |
| EDN1 | 9.33E-09 | 14.816994 | up |
| LEPREL2 | 1.22E-09 | 14.727476 | up |
| NUDT10 | 7.56E-08 | 14.703545 | up |
| LOC100129397 | 1.05E-06 | 14.398135 | up |
| LINGO2 | 9.22E-10 | 14.385864 | up |
| ITGB3 | 6.31E-08 | 14.356399 | up |
| C1orf54 | 2.28E-08 | 14.350257 | up |
| APCDD1L | 2.63E-08 | 14.119308 | up |
| KIT | 4.89E-09 | 14.097315 | up |
| IGSF1 | 1.38E-07 | 14.087812 | up |
| PTN | 1.07E-08 | 14.076056 | up |
| SGCD | 8.07E-08 | 13.876693 | up |
| JAM2 | 4.92E-08 | 13.804123 | up |
| TBX3 | 1.43E-06 | 13.585943 | up |
| NTN4 | 1.51E-07 | 13.585463 | up |
| LOC729860 | 5.37E-08 | 13.565369 | up |
| L1TD1 | 2.55E-09 | 13.493499 | up |
| ESRP2 | 6.11E-09 | 13.489346 | up |
| C4orf51 | 2.42E-07 | 13.475262 | up |
| PDGFB | 7.08E-09 | 13.472294 | up |
| HPGD | 1.48E-08 | 13.426594 | up |
| BMP7 | 1.24E-06 | 13.202074 | up |
| ZNF542P | 2.50E-08 | 13.1506 | up |
| ABCA1 | 6.92E-08 | 13.039741 | up |
| KL | 5.07E-07 | 12.922984 | up |
| PDE4D | 1.31E-06 | 12.884527 | up |
| ADAM12 | 6.56E-07 | 12.710247 | up |
| POMC | 4.52E-08 | 12.707695 | up |
| COL5A1 | 1.99E-08 | 12.62181 | up |
| CDC42BPG | 1.34E-06 | 12.598941 | up |
| MAPK13 | 7.44E-08 | 12.593763 | up |
| CYYR1 | 8.52E-07 | 12.538419 | up |
| PPP1R14C | 2.96E-09 | 12.506908 | up |
| TAGLN | 3.85E-09 | 12.465818 | up |
| ARVCF | 3.29E-09 | 12.464644 | up |
| SLC6A8 | 9.03E-09 | 12.396974 | up |
| CDH13 | 8.15E-10 | 12.366452 | up |
| PAPLN | 2.75E-07 | 12.331343 | up |
| TET1 | 2.59E-08 | 12.316185 | up |
| CACNA2D3 | 2.84E-07 | 12.227741 | up |
| SNORD116-19 | 8.31E-09 | 12.204782 | up |
| MYO5C | 1.32E-06 | 12.181686 | up |
| PTPRU | 3.77E-09 | 12.176419 | up |
| ERP27 | 1.00E-08 | 12.164022 | up |
| VGF | 1.13E-09 | 12.151555 | up |
| RNF175 | 3.24E-08 | 12.148547 | up |
| PPAN-P2RY11 | 1.22E-07 | 12.118219 | up |
| ABCB4 | 1.10E-08 | 12.118003 | up |
| PODXL | 5.10E-09 | 12.089357 | up |
| ANXA8L1 | 9.43E-08 | 12.034825 | up |
| PPP1R1A | 1.22E-06 | 12.031524 | up |
| SLC6A10P | 2.56E-07 | 12.022219 | up |
| ABCG4 | 7.43E-08 | 11.98874 | up |
| CAMTA1 | 1.47E-08 | 11.958754 | up |
| GPR87 | 7.12E-09 | 11.944174 | up |
| VASH1 | 1.69E-08 | 11.882957 | up |
| TMC6 | 1.04E-06 | 11.877544 | up |
| HS3ST2 | 8.02E-08 | 11.784266 | up |
| RBP7 | 1.97E-08 | 11.784143 | up |
| VSNL1 | 9.61E-09 | 11.782961 | up |
| NYNRIN | 2.06E-07 | 11.763674 | up |
| CSMD2 | 6.52E-09 | 11.717302 | up |
| FILIP1L | 7.50E-09 | 11.686987 | up |
| DOK7 | 7.95E-09 | 11.676066 | up |
| CCDC81 | 4.48E-09 | 11.67134 | up |
| HES4 | 1.50E-09 | 11.66832 | up |
| DSC2 | 1.08E-07 | 11.668297 | up |
| EDN1 | 3.33E-08 | 11.643775 | up |
| TAGLN | 3.60E-09 | 11.634117 | up |
| FAM150B | 8.75E-08 | 11.612874 | up |
| MEX3A | 9.17E-10 | 11.5382 | up |
| SHC4 | 5.56E-08 | 11.474473 | up |
| NFASC | 2.99E-09 | 11.469296 | up |
| GLIPR2 | 2.89E-09 | 11.442238 | up |
| LINC00312 | 1.53E-08 | 11.411936 | up |
| MAST4 | 1.41E-08 | 11.398846 | up |
| KDF1 | 2.25E-07 | 11.397538 | up |
| LOC100128242 | 3.05E-07 | 11.311417 | up |
| GDNF | 4.29E-08 | 11.261334 | up |
| GPA33 | 2.30E-08 | 11.163141 | up |
| GATM | 1.12E-07 | 11.1492 | up |
| STRA6 | 2.74E-07 | 11.130964 | up |
| PPP1R14A | 1.50E-08 | 11.123182 | up |
| lnc-FAM133B-1 | 3.56E-08 | 11.080586 | up |
| IL4I1 | 1.83E-09 | 11.07237 | up |
| NPR2 | 8.93E-08 | 11.051179 | up |
| KCNJ6 | 1.63E-07 | 11.030495 | up |
| SLC6A8 | 2.98E-09 | 11.022553 | up |
| CFAP46 | 1.20E-06 | 10.98483 | up |
| MIR4697HG | 5.80E-08 | 10.972385 | up |
| CABP7 | 7.16E-07 | 10.947148 | up |
| GYG2 | 6.51E-07 | 10.922617 | up |
| RPS23 | 1.43E-09 | 10.880847 | up |
| KALRN | 8.22E-08 | 10.83054 | up |
| OLFML2A | 4.60E-08 | 10.824901 | up |
| TEX12 | 1.65E-08 | 10.819038 | up |
| SULT4A1 | 1.14E-07 | 10.806875 | up |
| ACP5 | 2.74E-08 | 10.805989 | up |
| PGM2L1 | 4.89E-09 | 10.720828 | up |
| BACH2 | 7.92E-08 | 10.718252 | up |
| KCNMA1 | 7.55E-07 | 10.63747 | up |
| SNAI2 | 4.02E-08 | 10.621428 | up |
| FBLN1 | 1.32E-08 | 10.568066 | up |
| SIM2 | 2.41E-08 | 10.541462 | up |
| IL17RD | 2.29E-08 | 10.536267 | up |
| KCNH2 | 7.00E-08 | 10.496634 | up |
| FBN1 | 2.12E-09 | 10.484777 | up |
| NAP1L2 | 7.80E-07 | 10.44505 | up |
| FBLL1 | 7.37E-07 | 10.434098 | up |
| SSC5D | 2.26E-07 | 10.375037 | up |
| PDZRN3 | 2.45E-08 | 10.371123 | up |
| RNF183 | 4.78E-07 | 10.283304 | up |
| CRMP1 | 1.41E-08 | 10.276055 | up |
| LOC653602 | 1.45E-08 | 10.270805 | up |
| HSPB2 | 2.00E-08 | 10.256827 | up |
| NOX4 | 5.43E-08 | 10.252775 | up |
| SLC43A1 | 4.14E-07 | 10.242229 | up |
| CPXM1 | 7.61E-07 | 10.212667 | up |
| HOXA7 | 1.26E-07 | 10.16655 | up |
| ZNF667 | 9.00E-08 | 10.120791 | up |
| CHADL | 1.17E-06 | 10.10189 | up |
| SLITRK4 | 6.68E-09 | 10.09251 | up |
| GBP1 | 1.23E-07 | 10.080206 | up |
| ZNF681 | 9.65E-08 | 10.058204 | up |
| ESPNL | 6.38E-07 | 10.042461 | up |
| CERCAM | 2.94E-09 | 9.963496 | up |
| FBLN5 | 1.84E-08 | 9.942923 | up |
| TMEM63C | 1.09E-06 | 9.928772 | up |
| ADAM18 | 1.30E-07 | 9.912458 | up |
| FAM174B | 1.01E-07 | 9.901591 | up |
| MME | 5.58E-08 | 9.901095 | up |
| NLGN4Y | 4.74E-07 | 9.827568 | up |
| IQCA1 | 2.56E-09 | 9.771896 | up |
| ZNF506 | 5.96E-08 | 9.760702 | up |
| B3GAT1 | 1.27E-07 | 9.75946 | up |
| lnc-THNSL1-2 | 2.09E-08 | 9.745422 | up |
| RRAGD | 5.39E-08 | 9.744804 | up |
| BCL2 | 3.36E-08 | 9.736779 | up |
| KIAA1841 | 1.01E-07 | 9.70037 | up |
| SFMBT2 | 1.12E-07 | 9.696526 | up |
| EFNB1 | 1.35E-08 | 9.68236 | up |
| GLDC | 8.46E-08 | 9.675964 | up |
| LTBP2 | 6.04E-09 | 9.645136 | up |
| PDLIM3 | 3.11E-07 | 9.607471 | up |
| TNF | 3.00E-07 | 9.606977 | up |
| IL17RA | 5.51E-08 | 9.606616 | up |
| MTSS1 | 1.06E-08 | 9.599177 | up |
| TSTD1 | 1.59E-08 | 9.590768 | up |
| SERPINA3 | 1.05E-07 | 9.564667 | up |
| PLD5 | 6.97E-07 | 9.552226 | up |
| PLEKHO1 | 4.19E-09 | 9.539486 | up |
| PSG3 | 4.43E-07 | 9.517252 | up |
| IGFBP4 | 6.06E-09 | 9.476856 | up |
| RABGAP1L | 6.65E-08 | 9.468573 | up |
| FADS2 | 2.08E-09 | 9.45743 | up |
| ZNF876P | 7.97E-08 | 9.413562 | up |
| TC2N | 2.42E-08 | 9.396896 | up |
| NETO1 | 1.66E-07 | 9.376404 | up |
| CDH6 | 3.98E-09 | 9.325485 | up |
| LRRC34 | 1.99E-09 | 9.321754 | up |
| SKAP1 | 3.32E-07 | 9.320079 | up |
| EFNB3 | 1.56E-07 | 9.314001 | up |
| PEG3 | 5.89E-07 | 9.255297 | up |
| HRK | 1.88E-07 | 9.250609 | up |
| IGDCC4 | 1.46E-07 | 9.245033 | up |
| MXRA8 | 4.00E-08 | 9.241451 | up |
| ARID3A | 1.47E-08 | 9.171965 | up |
| KLF13 | 1.57E-08 | 9.171764 | up |
| ANKRD35 | 7.30E-08 | 9.151552 | up |
| NOTCH3 | 1.69E-08 | 9.138975 | up |
| ALPK2 | 9.17E-08 | 9.128014 | up |
| JAM2 | 3.49E-09 | 9.126855 | up |
| KANK4 | 9.12E-09 | 9.084839 | up |
| CISH | 1.03E-07 | 9.0831175 | up |
| TMSB15A | 2.78E-09 | 9.054492 | up |
| GABRB1 | 1.51E-08 | 9.043528 | up |
| PWARSN | 2.94E-07 | 9.02155 | up |
| ZNF582-AS1 | 3.19E-07 | 9.010379 | up |
| LPL | 3.47E-07 | 8.997885 | up |
| FAM101B | 1.58E-07 | 8.982499 | up |
| NTNG2 | 4.18E-07 | 8.97337 | up |
| TSPAN2 | 3.69E-07 | 8.946828 | up |
| ELMO1 | 4.15E-07 | 8.943671 | up |
| GRASP | 1.78E-07 | 8.89176 | up |
| UTY | 7.48E-07 | 8.844495 | up |
| PRSS1 | 8.43E-08 | 8.841317 | up |
| TRHDE-AS1 | 3.01E-08 | 8.826782 | up |
| ASXL3 | 9.46E-07 | 8.824086 | up |
| JAM2 | 7.56E-07 | 8.821118 | up |
| ANKRD30BP2 | 1.72E-08 | 8.818717 | up |
| ZNF253 | 1.86E-08 | 8.796709 | up |
| FGF11 | 6.93E-07 | 8.76925 | up |
| PTHLH | 1.13E-07 | 8.717588 | up |
| ZFP2 | 7.24E-08 | 8.685998 | up |
| C4orf26 | 3.69E-08 | 8.643629 | up |
| HLA-DQB1 | 1.18E-08 | 8.556858 | up |
| PARD6G | 1.17E-07 | 8.542819 | up |
| SAMD14 | 3.13E-07 | 8.537498 | up |
| ABCA7 | 5.98E-08 | 8.52638 | up |
| USP9Y | 2.71E-08 | 8.524063 | up |
| ACKR3 | 1.56E-07 | 8.517015 | up |
| UNC5B | 1.62E-07 | 8.513559 | up |
| LINC00900 | 7.41E-08 | 8.489972 | up |
| ARSI | 1.15E-06 | 8.48533 | up |
| SHANK3 | 3.99E-08 | 8.456412 | up |
| GAL | 1.26E-08 | 8.391877 | up |
| LPAR2 | 8.08E-09 | 8.386959 | up |
| FAM221A | 8.83E-07 | 8.349732 | up |
| SPON2 | 4.15E-09 | 8.343176 | up |
| C16orf86 | 1.13E-07 | 8.34095 | up |
| SMTNL2 | 2.99E-07 | 8.283728 | up |
| LMCD1 | 2.62E-08 | 8.252293 | up |
| SLC7A7 | 7.77E-08 | 8.239555 | up |
| B3GALT5-AS1 | 7.52E-09 | 8.221494 | up |
| TNFRSF19 | 5.58E-07 | 8.217222 | up |
| TRIM36 | 1.92E-07 | 8.211389 | up |
| KLK6 | 2.69E-07 | 8.166711 | up |
| COL5A1 | 1.69E-08 | 8.122619 | up |
| RNF208 | 2.06E-08 | 8.085962 | up |
| PSG10P | 4.25E-08 | 8.070838 | up |
| HOXC4 | 1.02E-07 | 8.045732 | up |
| BTBD11 | 8.39E-09 | 8.041948 | up |
| LSR | 5.13E-07 | 8.032349 | up |
| CXCL12 | 2.69E-07 | 8.016334 | up |
| CGB | 7.68E-09 | 7.990685 | up |
| UTY | 1.61E-08 | 7.989267 | up |
| FAM167B | 3.22E-08 | 7.9791517 | up |
| ADCY4 | 4.16E-08 | 7.9536586 | up |
| HID1 | 4.96E-09 | 7.952439 | up |
| STXBP6 | 5.01E-07 | 7.9328523 | up |
| ASPHD2 | 6.20E-09 | 7.888412 | up |
| ZNF542P | 6.57E-09 | 7.874832 | up |
| SYT7 | 6.28E-08 | 7.858171 | up |
| PKNOX2 | 1.52E-07 | 7.855376 | up |
| SUSD1 | 2.05E-08 | 7.830142 | up |
| INA | 2.71E-08 | 7.8016005 | up |
| IL32 | 5.82E-08 | 7.7546563 | up |
| BSN | 7.90E-07 | 7.7467265 | up |
| RNF208 | 3.43E-08 | 7.7316055 | up |
| PCDH7 | 1.74E-08 | 7.718039 | up |
| CHST15 | 5.96E-08 | 7.639945 | up |
| CD274 | 9.01E-08 | 7.633376 | up |
| MAGI2-IT1 | 1.11E-07 | 7.624295 | up |
| PLBD1 | 4.90E-07 | 7.6232924 | up |
| MMP9 | 4.89E-09 | 7.619261 | up |
| PALM | 4.75E-08 | 7.5976954 | up |
| ANKRD65 | 6.38E-07 | 7.5915446 | up |
| TNFRSF10D | 4.34E-09 | 7.5826836 | up |
| ZBTB46 | 6.26E-07 | 7.562288 | up |
| LAMP5 | 1.28E-07 | 7.555713 | up |
| ITGA1 | 6.05E-08 | 7.523354 | up |
| NTNG1 | 6.33E-07 | 7.5110345 | up |
| DACT2 | 3.51E-07 | 7.494525 | up |
| KCNMA1 | 6.52E-09 | 7.494456 | up |
| BCL11A | 3.07E-07 | 7.4792867 | up |
| NANOS3 | 3.55E-08 | 7.4668083 | up |
| CADM4 | 3.15E-07 | 7.4598856 | up |
| DACT1 | 6.49E-08 | 7.4392214 | up |
| PDE1C | 5.47E-08 | 7.424542 | up |
| PCK2 | 2.28E-07 | 7.419111 | up |
| TTC3 | 1.81E-08 | 7.417158 | up |
| VWCE | 2.06E-08 | 7.4009776 | up |
| PRDX2 | 4.84E-08 | 7.3822002 | up |
| JUP | 5.08E-09 | 7.3681836 | up |
| ADAM19 | 5.85E-09 | 7.3533626 | up |
| C19orf18 | 4.52E-07 | 7.2974033 | up |
| EFNB2 | 3.53E-08 | 7.247416 | up |
| DHRS4L1 | 7.96E-07 | 7.217787 | up |
| MYO5B | 3.49E-07 | 7.2121925 | up |
| HS3ST3B1 | 4.45E-08 | 7.208695 | up |
| GNAZ | 1.17E-07 | 7.201877 | up |
| F2R | 2.64E-08 | 7.175935 | up |
| PLEKHO1 | 7.59E-08 | 7.1735754 | up |
| lnc-ZNF717-1 | 3.10E-08 | 7.170942 | up |
| KCNK6 | 1.29E-08 | 7.1631837 | up |
| IL32 | 1.02E-07 | 7.1101418 | up |
| FA2H | 1.01E-07 | 7.090825 | up |
| COL3A1 | 1.44E-07 | 7.063225 | up |
| SOCS2-AS1 | 5.39E-09 | 7.0631423 | up |
| ERICH5 | 6.28E-08 | 7.0340967 | up |
| RTKN2 | 4.99E-07 | 7.0263834 | up |
| SLC25A41 | 5.65E-07 | 7.0114107 | up |
| CORO2B | 1.25E-07 | 7.002093 | up |
| EDNRA | 3.02E-07 | 6.9923863 | up |
| ACOT4 | 1.26E-06 | 6.9841304 | up |
| ART5 | 3.24E-09 | 6.9769816 | up |
| C2CD4B | 5.62E-08 | 6.9719367 | up |
| CBX2 | 1.60E-08 | 6.9633465 | up |
| PRSS23 | 3.06E-08 | 6.962133 | up |
| TMEM88 | 6.80E-08 | 6.947776 | up |
| SIX4 | 1.81E-07 | 6.921992 | up |
| PRR5-ARHGAP8 | 7.94E-07 | 6.9102793 | up |
| MPZL2 | 2.10E-07 | 6.8916874 | up |
| HDAC9 | 7.62E-08 | 6.874025 | up |
| SFRP1 | 8.59E-08 | 6.855062 | up |
| FBLN1 | 6.03E-09 | 6.852562 | up |
| MFSD7 | 5.52E-07 | 6.84386 | up |
| ARID3B | 3.92E-09 | 6.8395424 | up |
| PRICKLE2 | 1.32E-08 | 6.83314 | up |
| FLNA | 3.95E-07 | 6.832974 | up |
| TGFBR1 | 2.21E-07 | 6.8268642 | up |
| EXPH5 | 3.59E-08 | 6.816925 | up |
| NEXN | 1.85E-08 | 6.8114986 | up |
| FAM20C | 2.39E-08 | 6.8045444 | up |
| SARM1 | 8.20E-07 | 6.7689457 | up |
| MCAM | 3.45E-09 | 6.762767 | up |
| SDK2 | 2.11E-07 | 6.7594967 | up |
| TNFAIP3 | 8.57E-08 | 6.7576637 | up |
| INSIG1 | 3.48E-07 | 6.7059097 | up |
| SH3BGRL2 | 1.54E-07 | 6.691805 | up |
| DCLK2 | 4.88E-07 | 6.6637063 | up |
| THSD4 | 2.03E-07 | 6.649686 | up |
| SYTL1 | 4.10E-08 | 6.6463666 | up |
| FGF9 | 1.35E-06 | 6.621802 | up |
| CECR1 | 3.59E-07 | 6.5861883 | up |
| GPC6 | 7.37E-09 | 6.5524306 | up |
| LOXL3 | 2.20E-08 | 6.551346 | up |
| SLC2A5 | 6.13E-08 | 6.548229 | up |
| BMPR2 | 1.09E-07 | 6.532921 | up |
| ZNF260 | 1.17E-08 | 6.531402 | up |
| TENM4 | 1.33E-08 | 6.53132 | up |
| TOX3 | 1.01E-07 | 6.503179 | up |
| LOC644189 | 1.48E-07 | 6.500729 | up |
| SERINC2 | 5.83E-08 | 6.4952784 | up |
| RNF122 | 9.02E-08 | 6.485432 | up |
| HLA-DQB2 | 6.74E-07 | 6.4655156 | up |
| DSC3 | 1.59E-07 | 6.458763 | up |
| HAVCR2 | 1.19E-07 | 6.4418125 | up |
| CDH1 | 1.86E-07 | 6.4238524 | up |
| ARHGEF16 | 1.02E-07 | 6.415411 | up |
| CBLB | 2.32E-07 | 6.408398 | up |
| EDA | 1.45E-06 | 6.4015574 | up |
| GRAMD1B | 5.59E-07 | 6.3943276 | up |
| COL25A1 | 1.05E-06 | 6.3923473 | up |
| TPP1 | 4.36E-07 | 6.3595347 | up |
| BAZ2B | 5.44E-08 | 6.3589463 | up |
| FZD3 | 4.17E-07 | 6.322253 | up |
| DDX58 | 1.11E-08 | 6.301307 | up |
| KLF12 | 3.01E-07 | 6.3005886 | up |
| NREP | 9.60E-09 | 6.298595 | up |
| PXDN | 7.47E-09 | 6.2976427 | up |
| COBLL1 | 1.30E-07 | 6.2567 | up |
| ASPHD2 | 1.80E-07 | 6.2532525 | up |
| NREP | 7.51E-09 | 6.2461996 | up |
| AHNAK | 6.90E-09 | 6.217164 | up |
| ITGAV | 4.83E-09 | 6.182646 | up |
| CENPV | 9.31E-08 | 6.170906 | up |
| CAND2 | 3.26E-07 | 6.120367 | up |
| COL6A3 | 3.99E-07 | 6.1189218 | up |
| PXDN | 7.76E-09 | 6.105905 | up |
| ARHGEF40 | 1.39E-07 | 6.0981655 | up |
| TGFB1I1 | 3.92E-09 | 6.0763097 | up |
| CHST6 | 1.36E-06 | 6.0542145 | up |
| GOLGA8A | 1.06E-06 | 6.0513825 | up |
| CLEC11A | 7.48E-08 | 6.049174 | up |
| CELSR1 | 6.17E-09 | 6.0276103 | up |
| DPH3 | 9.60E-09 | 6.0243864 | up |
| PAPPA | 2.87E-08 | 6.020243 | up |
| MEI1 | 1.30E-06 | 5.995134 | up |
| TNS1 | 2.95E-07 | 5.9804916 | up |
| NNAT | 6.24E-08 | 5.968015 | up |
| TESC | 1.07E-08 | 5.9665666 | up |
| SEMA3B | 8.30E-08 | 5.9524612 | up |
| ACSM1 | 7.08E-08 | 5.940704 | up |
| DBNDD2 | 2.04E-08 | 5.937139 | up |
| GUCY1A2 | 4.66E-08 | 5.9363604 | up |
| SORT1 | 4.58E-08 | 5.9183483 | up |
| ATP10A | 1.58E-08 | 5.9078827 | up |
| M1AP | 8.16E-08 | 5.9015136 | up |
| PADI2 | 4.30E-09 | 5.892697 | up |
| HIGD1A | 7.72E-07 | 5.888752 | up |
| ZFHX2 | 1.37E-07 | 5.8869143 | up |
| GCNT4 | 7.45E-07 | 5.858186 | up |
| DNAH10 | 2.94E-07 | 5.8544683 | up |
| CD69 | 1.85E-07 | 5.8499866 | up |
| FN1 | 1.51E-07 | 5.8231297 | up |
| PMEPA1 | 3.08E-08 | 5.8152266 | up |
| DISP2 | 3.70E-08 | 5.811622 | up |
| FUT1 | 8.23E-08 | 5.8005276 | up |
| SPINT2 | 3.17E-08 | 5.793711 | up |
| LOXL3 | 1.21E-06 | 5.770476 | up |
| SLC4A11 | 2.88E-07 | 5.768609 | up |
| FOXP1 | 1.08E-07 | 5.7647285 | up |
| MMP13 | 6.50E-07 | 5.7634463 | up |
| ZNF558 | 8.04E-08 | 5.761019 | up |
| BEX4 | 3.40E-07 | 5.7467284 | up |
| RTN4R | 1.16E-08 | 5.7395387 | up |
| CSRP1 | 1.15E-07 | 5.7183657 | up |
| CEP44 | 7.66E-07 | 5.7104063 | up |
| NCMAP | 1.28E-06 | 5.7052364 | up |
| FZD4 | 3.67E-08 | 5.68709 | up |
| CCDC176 | 7.60E-07 | 5.6687803 | up |
| ITGB1BP1 | 2.09E-07 | 5.6531854 | up |
| GPRIN2 | 8.56E-08 | 5.634359 | up |
| FBXL16 | 4.38E-08 | 5.6201844 | up |
| KRT14 | 1.45E-06 | 5.603545 | up |
| MCHR1 | 4.59E-07 | 5.6021137 | up |
| ZDHHC3 | 2.90E-07 | 5.59161 | up |
| HSD17B1 | 9.70E-08 | 5.5762815 | up |
| ZNF345 | 7.23E-08 | 5.5513463 | up |
| S100A2 | 1.58E-08 | 5.54717 | up |
| LOC101929089 | 1.25E-07 | 5.545944 | up |
| MYH9 | 1.40E-07 | 5.54366 | up |
| BDH1 | 7.73E-08 | 5.5419736 | up |
| SNRPN | 3.97E-07 | 5.526876 | up |
| FAHD1 | 1.96E-08 | 5.51145 | up |
| C1QTNF4 | 1.89E-07 | 5.4870234 | up |
| FOXP2 | 9.40E-07 | 5.481463 | up |
| PLA2G4C | 2.91E-08 | 5.473066 | up |
| LINC00685 | 2.46E-07 | 5.4627104 | up |
| EFR3B | 2.74E-08 | 5.4433713 | up |
| MRGPRF | 7.27E-07 | 5.4402347 | up |
| MYOM2 | 3.46E-07 | 5.419232 | up |
| ACTR1B | 7.13E-07 | 5.415363 | up |
| KLHL13 | 7.31E-07 | 5.400609 | up |
| PRICKLE1 | 2.21E-08 | 5.38497 | up |
| B3GNT4 | 2.60E-07 | 5.3808393 | up |
| ARID5B | 2.03E-08 | 5.3758206 | up |
| ADPRH | 2.66E-07 | 5.362964 | up |
| MTSS1 | 7.14E-07 | 5.3008933 | up |
| ITPR2 | 1.75E-07 | 5.297878 | up |
| MGC20647 | 6.66E-07 | 5.2962174 | up |
| RIPK3 | 4.33E-07 | 5.2961674 | up |
| SP9 | 1.32E-07 | 5.2860503 | up |
| TMEM133 | 9.56E-08 | 5.2839265 | up |
| PDLIM3 | 3.42E-07 | 5.2835336 | up |
| CALD1 | 8.27E-08 | 5.278585 | up |
| MNX1 | 1.69E-07 | 5.265464 | up |
| CPE | 1.70E-08 | 5.2383604 | up |
| CMPK2 | 4.98E-07 | 5.2333694 | up |
| CHST2 | 6.36E-07 | 5.2207274 | up |
| DEF6 | 2.19E-07 | 5.2057943 | up |
| FZD7 | 1.04E-07 | 5.195226 | up |
| ZFP30 | 8.66E-08 | 5.194147 | up |
| ADAMTS9 | 3.83E-07 | 5.1827765 | up |
| SALL2 | 3.82E-08 | 5.1762414 | up |
| EGR2 | 1.80E-07 | 5.171964 | up |
| CRYAB | 2.18E-07 | 5.1330366 | up |
| CRB3 | 2.66E-07 | 5.127734 | up |
| FRMD4B | 4.65E-08 | 5.111581 | up |
| DSEL | 1.19E-07 | 5.0778255 | up |
| HSPA2 | 2.14E-08 | 5.0612335 | up |
| GPR126 | 4.43E-08 | 5.05929 | up |
| ENPP1 | 5.44E-08 | 5.0543804 | up |
| AGT | 2.75E-07 | 5.047042 | up |
| GJB2 | 7.31E-07 | 5.0444417 | up |
| COL18A1 | 1.14E-08 | 5.037473 | up |
| CGN | 9.15E-09 | 5.035107 | up |
| NDN | 3.27E-08 | 5.0254173 | up |
| SORBS2 | 1.40E-07 | 5.0155535 | up |
| KCNMA1 | 1.73E-07 | 5.0104136 | up |
| MAPK13 | 2.00E-07 | 4.9808187 | up |
| GLCCI1 | 8.67E-08 | 4.964261 | up |
| ANKRD20A2 | 4.24E-08 | 4.960597 | up |
| CBS | 2.33E-08 | 4.959448 | up |
| TP53INP2 | 7.12E-07 | 4.948188 | up |
| ALOX5AP | 1.81E-07 | 4.9451475 | up |
| KCNMA1 | 6.75E-07 | 4.944434 | up |
| PACS2 | 2.63E-07 | 4.93821 | up |
| FNBP1L | 7.21E-08 | 4.935937 | up |
| HBEGF | 2.51E-08 | 4.928561 | up |
| KIAA2022 | 4.01E-07 | 4.9283643 | up |
| EPHA1 | 8.51E-07 | 4.9198036 | up |
| SRSF8 | 9.11E-09 | 4.91512 | up |
| SOCS4 | 1.54E-07 | 4.9080243 | up |
| CHKB | 4.13E-08 | 4.90299 | up |
| TMEM240 | 7.28E-07 | 4.898261 | up |
| MYL7 | 1.26E-06 | 4.864258 | up |
| PRODH | 1.18E-06 | 4.835586 | up |
| ZNF788 | 1.32E-06 | 4.827903 | up |
| PLXNA3 | 1.78E-08 | 4.826485 | up |
| KALRN | 4.06E-08 | 4.8260403 | up |
| SLC16A8 | 6.57E-07 | 4.8224583 | up |
| ANKRD1 | 2.51E-08 | 4.816152 | up |
| EGR1 | 9.07E-09 | 4.811161 | up |
| HOXD1 | 2.12E-07 | 4.798506 | up |
| FAM155A | 7.60E-07 | 4.79528 | up |
| COL9A3 | 3.70E-07 | 4.767984 | up |
| HIVEP2 | 1.74E-07 | 4.7671213 | up |
| PIANP | 3.83E-07 | 4.757457 | up |
| IP6K2 | 1.62E-08 | 4.7563624 | up |
| AIF1L | 7.31E-07 | 4.7483335 | up |
| FAM63B | 2.84E-08 | 4.7445827 | up |
| PDIA3 | 6.01E-07 | 4.6987433 | up |
| BGN | 1.31E-06 | 4.69816 | up |
| PHACTR1 | 1.65E-07 | 4.692931 | up |
| GALNT6 | 6.15E-08 | 4.685088 | up |
| ST6GAL1 | 7.56E-08 | 4.6823745 | up |
| HCG18 | 8.74E-07 | 4.674039 | up |
| HMGCR | 1.07E-08 | 4.6554656 | up |
| HIP1R | 2.04E-07 | 4.6442075 | up |
| RPL27A | 4.43E-08 | 4.64227 | up |
| CELSR2 | 4.09E-07 | 4.640679 | up |
| TBC1D13 | 6.11E-07 | 4.6335545 | up |
| SHBG | 1.86E-07 | 4.6320715 | up |
| IER5L | 1.22E-08 | 4.628868 | up |
| ZBTB34 | 2.44E-08 | 4.6276445 | up |
| TNFSF12 | 2.01E-07 | 4.626359 | up |
| VPS13C | 1.60E-07 | 4.6210723 | up |
| NR2F1-AS1 | 1.43E-06 | 4.619185 | up |
| NKAIN1 | 8.65E-07 | 4.6144667 | up |
| CD99 | 4.15E-08 | 4.6138077 | up |
| SNAI3-AS1 | 8.26E-08 | 4.610768 | up |
| PDE6B | 1.35E-07 | 4.606015 | up |
| TAGLN3 | 3.32E-07 | 4.589313 | up |
| MEGF9 | 3.32E-08 | 4.581831 | up |
| PDGFA | 3.70E-08 | 4.580558 | up |
| CAMK2N1 | 9.86E-08 | 4.577628 | up |
| AKAP5 | 8.57E-08 | 4.5527396 | up |
| ARG2 | 3.71E-08 | 4.5511756 | up |
| MICALCL | 6.51E-07 | 4.5468516 | up |
| MAGI2 | 1.21E-06 | 4.540322 | up |
| ALDH1B1 | 3.22E-08 | 4.538961 | up |
| DDAH1 | 3.50E-08 | 4.538821 | up |
| SUSD4 | 2.15E-07 | 4.5360336 | up |
| TTC39A | 7.69E-07 | 4.5346565 | up |
| KCNIP1 | 6.08E-08 | 4.5331373 | up |
| BMP1 | 1.82E-07 | 4.5269847 | up |
| SCD | 2.09E-07 | 4.5160866 | up |
| NR2F1 | 6.74E-07 | 4.491287 | up |
| PDE1C | 1.09E-06 | 4.487513 | up |
| SLC6A9 | 8.93E-08 | 4.44373 | up |
| ZNF501 | 5.35E-08 | 4.441654 | up |
| TMEM200A | 3.16E-08 | 4.436535 | up |
| COL4A1 | 2.02E-07 | 4.423049 | up |
| SEC14L2 | 1.83E-07 | 4.418337 | up |
| MTMR9 | 2.88E-08 | 4.416545 | up |
| LOC729683 | 1.75E-07 | 4.4145684 | up |
| CRELD1 | 1.36E-07 | 4.4054403 | up |
| SLC2A6 | 7.71E-08 | 4.397283 | up |
| DSEL | 7.56E-07 | 4.3972416 | up |
| ZNF454 | 1.21E-06 | 4.394176 | up |
| MYH10 | 1.55E-07 | 4.380433 | up |
| SYNC | 1.07E-07 | 4.375871 | up |
| ZBTB42 | 2.64E-08 | 4.369688 | up |
| CST6 | 7.35E-08 | 4.3614454 | up |
| SERPINA1 | 1.23E-07 | 4.3459563 | up |
| SPOCK1 | 5.92E-07 | 4.3434863 | up |
| ZNF69 | 7.74E-08 | 4.339909 | up |
| RPL23AP7 | 2.98E-07 | 4.3324537 | up |
| ZNF606 | 4.01E-07 | 4.328725 | up |
| ITM2C | 6.68E-07 | 4.319741 | up |
| EPB41L5 | 3.44E-07 | 4.317048 | up |
| FRMD5 | 5.45E-08 | 4.316634 | up |
| LAMB2 | 5.43E-08 | 4.315765 | up |
| SPSB1 | 1.41E-07 | 4.311218 | up |
| KLF12 | 1.28E-08 | 4.304907 | up |
| CSRP2 | 5.02E-08 | 4.3018184 | up |
| IFFO2 | 3.86E-08 | 4.2989836 | up |
| TAS2R30 | 8.13E-07 | 4.2952204 | up |
| YBEY | 6.27E-07 | 4.2883873 | up |
| HOXA11-AS | 4.25E-08 | 4.2847137 | up |
| RINL | 2.50E-07 | 4.2827215 | up |
| BLOC1S6 | 1.92E-07 | 4.273573 | up |
| ZNF112 | 1.42E-07 | 4.266481 | up |
| CDH11 | 1.45E-07 | 4.265701 | up |
| RHOB | 3.76E-08 | 4.263196 | up |
| QPCT | 5.40E-08 | 4.262102 | up |
| SOS2 | 6.61E-08 | 4.2574043 | up |
| FLNB | 1.03E-07 | 4.2444553 | up |
| ANO8 | 1.32E-07 | 4.2420535 | up |
| MICAL1 | 5.55E-08 | 4.238294 | up |
| ANK3 | 6.72E-07 | 4.2323484 | up |
| SIPA1L1 | 1.40E-07 | 4.2265806 | up |
| RHOBTB1 | 6.50E-08 | 4.220623 | up |
| PGBD1 | 6.55E-07 | 4.2113442 | up |
| F2RL2 | 4.35E-08 | 4.2110586 | up |
| SYNE2 | 8.01E-07 | 4.207934 | up |
| MEGF8 | 3.21E-08 | 4.2041225 | up |
| RDH11 | 7.59E-07 | 4.198581 | up |
| MMP23B | 3.59E-07 | 4.195893 | up |
| QPCT | 1.72E-07 | 4.188316 | up |
| TPTE | 6.23E-07 | 4.187213 | up |
| CRIP1 | 5.82E-08 | 4.1805825 | up |
| CCDC171 | 1.86E-07 | 4.176396 | up |
| ACTR10 | 1.29E-06 | 4.1708903 | up |
| LINC00087 | 2.78E-08 | 4.169519 | up |
| MARVELD3 | 9.92E-07 | 4.1671085 | up |
| MAVS | 1.38E-06 | 4.16545 | up |
| HIP1R | 6.17E-08 | 4.1627903 | up |
| GARNL3 | 3.74E-07 | 4.1442623 | up |
| MMP15 | 5.16E-07 | 4.129373 | up |
| BRINP1 | 3.41E-07 | 4.1154747 | up |
| PROS1 | 5.83E-07 | 4.1139493 | up |
| FNIP2 | 4.01E-08 | 4.113732 | up |
| PDGFC | 2.50E-07 | 4.1044407 | up |
| NPM2 | 1.18E-07 | 4.090131 | up |
| LMO7 | 5.23E-07 | 4.079605 | up |
| HIVEP3 | 1.69E-07 | 4.0755844 | up |
| IRF5 | 1.65E-07 | 4.07368 | up |
| NR4A3 | 1.28E-06 | 4.0727725 | up |
| lnc-ANP32A-3 | 1.65E-07 | 4.065538 | up |
| LRRN2 | 7.41E-07 | 4.0587044 | up |
| SACS | 7.12E-08 | 4.054675 | up |
| NUAK2 | 3.49E-08 | 4.0387917 | up |
| MT1F | 4.17E-08 | 4.035997 | up |
| UNC5B-AS1 | 1.81E-07 | 4.0296817 | up |
| RANBP2 | 1.04E-07 | 4.025498 | up |
| ROCK2 | 7.40E-07 | 4.0236382 | up |
| DKK3 | 1.13E-07 | 4.0232573 | up |
| ZNF572 | 8.51E-07 | 4.014597 | up |
| ITGA6 | 1.10E-07 | 4.0094647 | up |
| APOC1 | 1.25E-07 | 4.0023236 | up |
| IMPACT | 5.22E-07 | 4.0000978 | up |
| ULK2 | 2.25E-07 | 3.9950318 | up |
| SIX2 | 1.81E-07 | 3.986483 | up |
| MEF2A | 4.45E-08 | 3.9838433 | up |
| CAMSAP3 | 4.48E-07 | 3.9811344 | up |
| FNDC4 | 1.20E-06 | 3.9795492 | up |
| HEY1 | 1.34E-07 | 3.9719303 | up |
| PDZD7 | 3.38E-07 | 3.97158 | up |
| GHDC | 1.34E-07 | 3.959141 | up |
| CYP2S1 | 6.24E-08 | 3.9566991 | up |
| TNFRSF11B | 2.14E-07 | 3.955396 | up |
| KLHL24 | 1.54E-07 | 3.94815 | up |
| TRIM46 | 1.28E-07 | 3.9461176 | up |
| CTTNBP2 | 2.90E-07 | 3.9438097 | up |
| EPB41L4A-AS2 | 6.96E-08 | 3.943593 | up |
| STAP2 | 3.06E-08 | 3.9417145 | up |
| SIRT5 | 1.03E-06 | 3.9416075 | up |
| PTP4A3 | 1.04E-06 | 3.9408638 | up |
| SORCS2 | 4.79E-07 | 3.9391928 | up |
| LPAR6 | 1.92E-07 | 3.9368012 | up |
| NT5DC2 | 2.90E-07 | 3.9322088 | up |
| GJA1 | 3.37E-08 | 3.9303188 | up |
| ARSJ | 9.37E-08 | 3.9268909 | up |
| SOX4 | 8.84E-08 | 3.9238782 | up |
| PLEKHG3 | 3.35E-08 | 3.9025939 | up |
| SLC25A23 | 3.08E-07 | 3.8940806 | up |
| DENND2A | 2.50E-07 | 3.8928075 | up |
| TIA1 | 2.27E-07 | 3.8864439 | up |
| LOC100131289 | 3.32E-07 | 3.8785472 | up |
| SULF2 | 4.84E-07 | 3.8757584 | up |
| TRPC4 | 1.83E-07 | 3.8753974 | up |
| PLEKHG3 | 2.69E-08 | 3.872353 | up |
| CD200 | 1.41E-06 | 3.8572474 | up |
| ST3GAL5 | 1.96E-07 | 3.85715 | up |
| LINC00641 | 8.73E-07 | 3.8557193 | up |
| PTPRK | 1.31E-06 | 3.8513515 | up |
| FOXD1 | 1.59E-07 | 3.845659 | up |
| RTKN2 | 2.91E-07 | 3.8430648 | up |
| ZBTB8A | 1.41E-07 | 3.8427434 | up |
| ZNF33B | 8.63E-07 | 3.8375633 | up |
| KLHL23 | 8.27E-08 | 3.8333857 | up |
| CTNND2 | 1.61E-07 | 3.8199415 | up |
| REEP2 | 1.28E-07 | 3.8171277 | up |
| ZSCAN9 | 1.21E-07 | 3.8162763 | up |
| TEP1 | 5.33E-07 | 3.814121 | up |
| STARD10 | 3.57E-08 | 3.7934794 | up |
| F2R | 1.04E-07 | 3.79335 | up |
| NID2 | 1.58E-07 | 3.7860358 | up |
| SLC20A2 | 1.40E-06 | 3.781441 | up |
| ZSCAN29 | 1.15E-06 | 3.7811346 | up |
| MYADM | 1.56E-07 | 3.7797806 | up |
| SALL1 | 6.26E-07 | 3.7769434 | up |
| ZBTB21 | 1.07E-07 | 3.774846 | up |
| HES6 | 2.72E-08 | 3.7625897 | up |
| CLIC4 | 6.12E-08 | 3.7601383 | up |
| SGK1 | 6.39E-08 | 3.7578428 | up |
| CEP128 | 1.21E-07 | 3.750286 | up |
| FAM226A | 1.52E-07 | 3.742032 | up |
| SPSB1 | 2.33E-07 | 3.7417276 | up |
| HAUS2 | 7.96E-07 | 3.7401798 | up |
| TINAG | 1.53E-07 | 3.7393012 | up |
| SLC26A6 | 8.43E-08 | 3.736733 | up |
| LOC100131541 | 2.06E-07 | 3.73122 | up |
| TIAM2 | 5.86E-08 | 3.730526 | up |
| NOG | 1.17E-06 | 3.728593 | up |
| FAM69B | 3.59E-07 | 3.7269912 | up |
| FUT8-AS1 | 3.81E-07 | 3.7257128 | up |
| TPM1 | 2.04E-07 | 3.7196512 | up |
| COL4A2 | 5.18E-08 | 3.7134848 | up |
| PDLIM5 | 1.81E-07 | 3.7097178 | up |
| ENC1 | 5.61E-08 | 3.6957157 | up |
| CDS1 | 4.51E-07 | 3.6956828 | up |
| FAM179B | 1.68E-07 | 3.6945486 | up |
| FAM210B | 8.87E-08 | 3.6815038 | up |
| ZNF615 | 3.97E-07 | 3.6811688 | up |
| ZDHHC8 | 1.18E-07 | 3.678205 | up |
| ALDH4A1 | 3.43E-08 | 3.6748335 | up |
| MIB1 | 3.19E-07 | 3.6738098 | up |
| MARCH4 | 3.94E-07 | 3.6691298 | up |
| GATSL3 | 6.95E-08 | 3.6653903 | up |
| DMKN | 6.29E-07 | 3.660155 | up |
| TOP2B | 4.58E-08 | 3.6565516 | up |
| SLC1A4 | 5.16E-08 | 3.6562564 | up |
| DOCK11 | 3.98E-07 | 3.6559 | up |
| ARHGAP31 | 3.63E-07 | 3.645004 | up |
| CD82 | 1.18E-07 | 3.641176 | up |
| PHOSPHO2-KLHL23 | 9.30E-07 | 3.6388886 | up |
| ZNF792 | 2.50E-07 | 3.633459 | up |
| TCEA3 | 5.71E-07 | 3.6212602 | up |
| ENO3 | 3.65E-07 | 3.6171563 | up |
| GLB1L | 5.73E-07 | 3.6128001 | up |
| SSTR1 | 1.61E-07 | 3.6114888 | up |
| KIF3C | 1.72E-07 | 3.6110742 | up |
| TMEM8A | 7.30E-07 | 3.6039777 | up |
| AGRN | 3.74E-07 | 3.6019704 | up |
| TPM4 | 8.95E-07 | 3.5995831 | up |
| RHOD | 4.89E-07 | 3.5967796 | up |
| COL4A6 | 1.41E-07 | 3.5842173 | up |
| TMEM154 | 6.24E-07 | 3.583478 | up |
| ANKRD20A12P | 1.06E-06 | 3.5832956 | up |
| BEAN1 | 1.21E-06 | 3.5826313 | up |
| PRKCD | 1.62E-07 | 3.5778732 | up |
| USP54 | 4.45E-07 | 3.575336 | up |
| POMT2 | 4.09E-08 | 3.5646732 | up |
| GCNT1 | 2.13E-07 | 3.5635552 | up |
| ARHGAP5 | 4.15E-07 | 3.563093 | up |
| EDNRA | 4.21E-08 | 3.5625641 | up |
| ANXA3 | 8.42E-08 | 3.5621085 | up |
| ACAA2 | 1.12E-07 | 3.5575554 | up |
| TAMM41 | 6.90E-07 | 3.556731 | up |
| TRIM36 | 4.93E-07 | 3.5494087 | up |
| IGFBP1 | 8.98E-07 | 3.5464122 | up |
| RIC1 | 1.48E-07 | 3.5442965 | up |
| KIAA0408 | 5.44E-08 | 3.5419888 | up |
| ZMYM2 | 2.31E-07 | 3.5405815 | up |
| TJP2 | 8.18E-07 | 3.5376348 | up |
| GRB14 | 6.40E-08 | 3.5373673 | up |
| FSCN1 | 7.74E-08 | 3.5262778 | up |
| LRRFIP1 | 1.06E-06 | 3.516484 | up |
| TRIM2 | 1.90E-07 | 3.512511 | up |
| FOXN3 | 5.57E-07 | 3.5112922 | up |
| TMF1 | 6.65E-08 | 3.5087237 | up |
| ELOVL6 | 5.72E-07 | 3.5065384 | up |
| FOXP1 | 6.05E-07 | 3.4981856 | up |
| EMC10 | 1.45E-07 | 3.4965165 | up |
| CD59 | 9.70E-08 | 3.4952273 | up |
| TPM4 | 9.96E-08 | 3.4868464 | up |
| FERMT2 | 1.68E-07 | 3.4815414 | up |
| SNURF | 8.59E-08 | 3.4805748 | up |
| MFGE8 | 1.07E-07 | 3.4760354 | up |
| CTNNB1 | 2.54E-07 | 3.467719 | up |
| MAP4K5 | 3.91E-07 | 3.4512863 | up |
| GOLGA2P5 | 5.27E-07 | 3.4491072 | up |
| MYRF | 8.95E-07 | 3.4404628 | up |
| ANG | 1.45E-07 | 3.439741 | up |
| CD200 | 4.87E-07 | 3.4351165 | up |
| MEIS2 | 1.47E-07 | 3.4338396 | up |
| LRRC8A | 7.63E-08 | 3.4218686 | up |
| PPP2R3A | 4.70E-07 | 3.4156435 | up |
| NINL | 7.25E-08 | 3.4127855 | up |
| PTPRK | 1.63E-07 | 3.4123938 | up |
| CST5 | 2.69E-07 | 3.4119034 | up |
| FAM84B | 9.82E-08 | 3.411853 | up |
| LRRFIP1 | 5.51E-07 | 3.4002304 | up |
| AGMAT | 9.10E-08 | 3.3950512 | up |
| SNX22 | 1.23E-06 | 3.393647 | up |
| ZFYVE1 | 1.59E-07 | 3.391597 | up |
| SMPDL3A | 6.50E-07 | 3.3836632 | up |
| PCYOX1 | 6.90E-08 | 3.3771574 | up |
| FAM126B | 8.25E-08 | 3.3741696 | up |
| ZNF616 | 5.02E-07 | 3.3735404 | up |
| TPM1 | 6.93E-08 | 3.3687663 | up |
| SH3BGRL2 | 4.60E-07 | 3.3637974 | up |
| PTPN13 | 5.29E-07 | 3.3541055 | up |
| NEK9 | 8.68E-08 | 3.3534977 | up |
| BCAR3 | 1.93E-07 | 3.3521876 | up |
| FERMT2 | 9.23E-07 | 3.351406 | up |
| CACNA1H | 3.26E-07 | 3.3479652 | up |
| PLEKHA5 | 2.51E-07 | 3.3408132 | up |
| BICD1 | 2.38E-07 | 3.3373654 | up |
| ADM2 | 1.50E-07 | 3.33645 | up |
| TLR2 | 5.25E-07 | 3.3347197 | up |
| TMEM132A | 1.68E-07 | 3.3244803 | up |
| RHOB | 1.25E-06 | 3.3176653 | up |
| GLIDR | 3.07E-07 | 3.3170683 | up |
| LOC285043 | 3.78E-07 | 3.3160052 | up |
| CST3 | 1.86E-07 | 3.3145614 | up |
| TMOD1 | 7.06E-07 | 3.3112016 | up |
| PLXND1 | 1.15E-06 | 3.307494 | up |
| ZNF165 | 4.60E-07 | 3.2991374 | up |
| SPHK1 | 2.78E-07 | 3.2980287 | up |
| MICA | 3.36E-07 | 3.2957346 | up |
| LOC389831 | 1.94E-07 | 3.2937832 | up |
| HOXD10 | 3.06E-07 | 3.2879815 | up |
| RBM41 | 2.59E-07 | 3.2862477 | up |
| BVES | 8.31E-07 | 3.2833595 | up |
| CLTCL1 | 1.45E-07 | 3.2821622 | up |
| HMGCS1 | 4.70E-07 | 3.2821574 | up |
| KLF9 | 2.14E-07 | 3.2786026 | up |
| EFNA1 | 7.33E-08 | 3.2784066 | up |
| CDIP1 | 1.23E-07 | 3.274401 | up |
| AK3 | 1.03E-07 | 3.2736201 | up |
| ZNF93 | 7.25E-08 | 3.2711046 | up |
| TNFRSF21 | 4.33E-08 | 3.2706597 | up |
| SIX1 | 5.88E-08 | 3.2700293 | up |
| UNC13B | 1.76E-07 | 3.2660685 | up |
| PTBP3 | 1.32E-07 | 3.2608638 | up |
| PELI2 | 1.42E-06 | 3.2580905 | up |
| FRMD6 | 2.80E-07 | 3.2517264 | up |
| SHROOM3 | 1.17E-07 | 3.2512422 | up |
| ARHGAP5 | 5.41E-07 | 3.241134 | up |
| GLT8D1 | 1.88E-07 | 3.227715 | up |
| CD248 | 9.29E-07 | 3.21494 | up |
| KALRN | 1.22E-06 | 3.211568 | up |
| ARPP19 | 1.24E-06 | 3.2083216 | up |
| PRKCZ | 1.19E-07 | 3.2068603 | up |
| PKN2 | 8.52E-08 | 3.2053845 | up |
| KHNYN | 1.14E-07 | 3.2052276 | up |
| SAMD4A | 2.32E-07 | 3.2020864 | up |
| CLASP1 | 2.28E-07 | 3.195722 | up |
| TMEM158 | 1.16E-06 | 3.1909213 | up |
| INHBA | 1.07E-07 | 3.187122 | up |
| CLIC3 | 1.84E-07 | 3.1859264 | up |
| TES | 8.25E-07 | 3.1813936 | up |
| CNN3 | 1.33E-07 | 3.180491 | up |
|  | 4.31E-07 | 3.17188 | up |
| BNIP2 | 1.68E-07 | 3.1657734 | up |
| GLIPR1 | 1.06E-07 | 3.1634943 | up |
| LRIG3 | 1.16E-06 | 3.1606083 | up |
| LOC151174 | 2.48E-07 | 3.1586847 | up |
| TLR4 | 1.18E-06 | 3.1570344 | up |
| DOCK9-AS2 | 3.17E-07 | 3.1452084 | up |
| DNAJB5 | 2.28E-07 | 3.1446989 | up |
| TAPBP | 9.07E-08 | 3.119625 | up |
| OTUD1 | 2.07E-07 | 3.1138468 | up |
| PMEPA1 | 1.43E-06 | 3.1081216 | up |
| DST | 6.76E-07 | 3.1045282 | up |
| NEDD4L | 2.34E-07 | 3.1008806 | up |
| H2AFY2 | 2.00E-07 | 3.090319 | up |
| RAB11FIP1 | 2.81E-07 | 3.0856009 | up |
| SLC8A1 | 5.35E-07 | 3.0812457 | up |
| DLST | 1.18E-06 | 3.0811183 | up |
| CLIC4 | 1.18E-07 | 3.0798624 | up |
| FAM76B | 1.23E-07 | 3.0771673 | up |
| PLEKHG2 | 4.47E-07 | 3.0769572 | up |
| IGSF9B | 4.34E-07 | 3.074807 | up |
| RAB3B | 4.84E-07 | 3.0741565 | up |
| SPATA7 | 6.28E-07 | 3.0738425 | up |
| CD2AP | 5.12E-07 | 3.059299 | up |
| LPHN2 | 2.82E-07 | 3.0569155 | up |
| TMEM30A | 8.01E-07 | 3.0559976 | up |
| HDAC9 | 4.70E-07 | 3.055727 | up |
| LDLR | 1.66E-07 | 3.0557148 | up |
| SETD2 | 6.17E-08 | 3.0548902 | up |
| TMCC1 | 3.59E-07 | 3.0500917 | up |
| ZNF555 | 4.64E-07 | 3.0489519 | up |
| PELI1 | 7.17E-08 | 3.0462017 | up |
| PCBP4 | 7.56E-08 | 3.0420594 | up |
| GALNT7 | 1.08E-07 | 3.0384793 | up |
| MOXD1 | 9.78E-08 | 3.037614 | up |
| FARP1 | 6.23E-07 | 3.0375793 | up |
| SH3PXD2A | 2.82E-07 | 3.034896 | up |
| FOSB | 9.34E-07 | 3.0283742 | up |
| PNPLA3 | 1.08E-07 | 3.0281022 | up |
| BOK | 6.57E-07 | 3.022375 | up |
| DOCK9 | 2.80E-07 | 3.0186152 | up |
| C3orf18 | 9.00E-08 | 3.012017 | up |
| RC3H1 | 1.69E-07 | 3.0104306 | up |
| KMT2E | 8.69E-07 | 3.0089793 | up |
| DICER1 | 1.15E-07 | 3.007435 | up |
| GLT8D1 | 3.43E-07 | 3.0018072 | up |
| C6orf120 | 5.07E-07 | 2.9998188 | up |
| CDH3 | 1.15E-06 | 2.999308 | up |
| ATXN3 | 1.98E-07 | 2.9978619 | up |
| AGPS | 1.16E-07 | 2.996668 | up |
| RGPD5 | 8.58E-07 | 2.996143 | up |
| SPG11 | 2.76E-07 | 2.9956887 | up |
| COL5A2 | 3.40E-07 | 2.9956553 | up |
| TXLNGY | 1.37E-06 | 2.993905 | up |
| FUT8 | 1.63E-07 | 2.9825153 | up |
| CYP17A1-AS1 | 9.06E-07 | 2.9799106 | up |
| ATXN1 | 4.72E-07 | 2.979156 | up |
| TTF1 | 1.96E-07 | 2.9758637 | up |
| ITGB8 | 1.31E-06 | 2.968328 | up |
| NOSTRIN | 9.22E-07 | 2.967063 | up |
| PRSS22 | 5.05E-07 | 2.9650705 | up |
| DCLK1 | 9.42E-08 | 2.9570618 | up |
| LOC100129473 | 6.38E-07 | 2.9545813 | up |
| LINC00504 | 1.22E-06 | 2.949397 | up |
| VPS4B | 4.69E-07 | 2.9486523 | up |
| ATP2B4 | 8.42E-08 | 2.9475007 | up |
| ZNF250 | 3.02E-07 | 2.9447467 | up |
| LINC00662 | 3.67E-07 | 2.943039 | up |
| VPS53 | 5.17E-07 | 2.9409823 | up |
| ERAP2 | 2.83E-07 | 2.9368324 | up |
| EXD3 | 6.21E-07 | 2.9337285 | up |
| SLC44A3 | 1.28E-06 | 2.928477 | up |
| SAMD4A | 4.99E-07 | 2.9269104 | up |
| NIN | 1.15E-06 | 2.9244413 | up |
| CHD2 | 9.94E-08 | 2.923761 | up |
| FBXL17 | 3.94E-07 | 2.92373 | up |
| TCF4 | 1.45E-06 | 2.923269 | up |
| PLGRKT | 1.24E-07 | 2.9213848 | up |
| LOC100131564 | 8.62E-07 | 2.9204023 | up |
| EP300 | 3.87E-07 | 2.9180443 | up |
| RFTN1 | 1.22E-07 | 2.9179347 | up |
| SUSD6 | 8.04E-08 | 2.9153762 | up |
| ACTN1 | 3.95E-07 | 2.9100478 | up |
| C11orf52 | 6.36E-07 | 2.9079146 | up |
| ARSA | 4.37E-07 | 2.905458 | up |
| BTBD7 | 1.79E-07 | 2.9050062 | up |
| AGO4 | 1.35E-06 | 2.9005218 | up |
| HTRA1 | 2.49E-07 | 2.8970704 | up |
| SETX | 7.15E-07 | 2.8963978 | up |
| RCAN2 | 8.26E-07 | 2.8962886 | up |
| SCGB3A2 | 1.03E-06 | 2.890477 | up |
| LRRN1 | 2.40E-07 | 2.8866549 | up |
| LOC102723428 | 1.75E-07 | 2.8856084 | up |
| EMP2 | 1.01E-06 | 2.8855796 | up |
| RNF19A | 8.98E-07 | 2.8835883 | up |
| SCARB2 | 1.39E-07 | 2.8828332 | up |
| CDH2 | 6.18E-07 | 2.8768902 | up |
| DST | 1.34E-06 | 2.8752954 | up |
| PLCD1 | 9.55E-08 | 2.8730278 | up |
| ACSL3 | 1.01E-06 | 2.8651469 | up |
| CGNL1 | 1.80E-07 | 2.8603282 | up |
| DPP7 | 4.84E-07 | 2.8601556 | up |
| CHAC1 | 1.61E-07 | 2.8595147 | up |
| ZNF555 | 9.43E-07 | 2.853358 | up |
| BRK1 | 1.69E-07 | 2.8529 | up |
| PTPN21 | 1.29E-07 | 2.8523204 | up |
| ZNF655 | 2.14E-07 | 2.8490658 | up |
| PKN3 | 2.92E-07 | 2.8480165 | up |
| LOXL4 | 3.91E-07 | 2.8478727 | up |
| EIF2S1 | 6.85E-07 | 2.8478415 | up |
| CASK | 8.57E-07 | 2.8477745 | up |
| ZNF133 | 7.13E-07 | 2.8463504 | up |
| SEPT11 | 8.26E-08 | 2.8460238 | up |
| WISP3 | 7.42E-07 | 2.8458087 | up |
| TTLL1 | 1.46E-07 | 2.8454444 | up |
| DOPEY2 | 2.97E-07 | 2.8443267 | up |
| MAP7 | 6.32E-07 | 2.8442714 | up |
| USP8 | 1.28E-06 | 2.843903 | up |
| CCNG2 | 5.01E-07 | 2.843348 | up |
| TTF1 | 6.60E-07 | 2.834751 | up |
| PCBP4 | 1.42E-07 | 2.8344738 | up |
| TTLL1 | 4.69E-07 | 2.8324258 | up |
| RTN2 | 3.09E-07 | 2.8315144 | up |
| CIRBP | 8.37E-08 | 2.8281229 | up |
| CTGF | 6.20E-07 | 2.8268409 | up |
| RICTOR | 1.72E-07 | 2.8211746 | up |
| LINC00504 | 6.59E-07 | 2.8205566 | up |
| ZGLP1 | 7.99E-07 | 2.8113937 | up |
| PURB | 8.39E-08 | 2.808189 | up |
| SPTBN1 | 1.07E-06 | 2.807989 | up |
| PKI55 | 3.25E-07 | 2.8069363 | up |
| ARHGAP27 | 3.26E-07 | 2.8036168 | up |
| BPHL | 4.71E-07 | 2.7990577 | up |
| ICAM1 | 1.45E-07 | 2.7956793 | up |
| ADAM10 | 1.15E-07 | 2.7948713 | up |
| MMAB | 1.20E-06 | 2.790267 | up |
| RAMP1 | 4.67E-07 | 2.7886596 | up |
| PLEKHA7 | 3.95E-07 | 2.7865984 | up |
| ASB2 | 2.77E-07 | 2.7850258 | up |
| LIF | 3.47E-07 | 2.7836692 | up |
| CDK19 | 1.86E-07 | 2.7783766 | up |
| NHS | 7.62E-07 | 2.7777674 | up |
| HOXD11 | 9.42E-07 | 2.7752757 | up |
| WDR90 | 5.57E-07 | 2.7735848 | up |
| ZNF580 | 2.56E-07 | 2.7729654 | up |
| LIMA1 | 1.39E-07 | 2.772908 | up |
| SNN | 1.16E-06 | 2.7709453 | up |
| LRRC49 | 1.13E-07 | 2.7696912 | up |
| TGIF2 | 8.60E-07 | 2.769653 | up |
| ENPP1 | 1.00E-06 | 2.7687454 | up |
| TSPYL5 | 9.71E-08 | 2.7680552 | up |
| SON | 1.12E-06 | 2.763801 | up |
| ZNF100 | 3.66E-07 | 2.762638 | up |
| PPP1R13L | 1.19E-07 | 2.7616389 | up |
| HYAL3 | 5.08E-07 | 2.7606595 | up |
| MAPK11 | 2.46E-07 | 2.759323 | up |
| GNAI3 | 6.05E-07 | 2.757046 | up |
| HAUS6 | 3.07E-07 | 2.7562375 | up |
| ZNF264 | 3.30E-07 | 2.7531333 | up |
| STRBP | 3.13E-07 | 2.752577 | up |
| AGL | 3.02E-07 | 2.7491112 | up |
| MYO1E | 4.70E-07 | 2.746336 | up |
| UQCR10 | 7.27E-07 | 2.7418222 | up |
| GATSL3 | 1.59E-07 | 2.736293 | up |
| CYP51A1 | 4.05E-07 | 2.7359216 | up |
| SAV1 | 1.99E-07 | 2.7266166 | up |
| RASSF4 | 1.42E-07 | 2.722053 | up |
| RFX7 | 1.76E-07 | 2.7218928 | up |
| NIPBL | 1.09E-06 | 2.7216325 | up |
| FAR2 | 2.76E-07 | 2.7157128 | up |
| SREBF1 | 1.75E-07 | 2.7138493 | up |
| MARCKSL1 | 7.98E-07 | 2.7135742 | up |
| C20orf194 | 1.63E-07 | 2.713318 | up |
| DPP4 | 1.14E-06 | 2.7126615 | up |
| NMNAT1 | 2.73E-07 | 2.7122633 | up |
| C7orf73 | 1.75E-07 | 2.7115715 | up |
| EFEMP2 | 3.78E-07 | 2.7111645 | up |
| RPL23AP53 | 9.98E-07 | 2.7107334 | up |
| TADA3 | 9.24E-07 | 2.7077174 | up |
| WLS | 1.93E-07 | 2.7076578 | up |
| ATRX | 4.87E-07 | 2.6986032 | up |
| NEURL3 | 4.18E-07 | 2.6967802 | up |
| GUCY1A3 | 3.21E-07 | 2.6944516 | up |
| EPB41L4A | 8.27E-07 | 2.6933844 | up |
| FRMD5 | 6.47E-07 | 2.6865 | up |
| IRAK1BP1 | 3.07E-07 | 2.685266 | up |
| SLC1A1 | 1.79E-07 | 2.673368 | up |
| OPTN | 9.44E-07 | 2.6701014 | up |
| NR6A1 | 8.65E-07 | 2.6674585 | up |
| C1orf233 | 5.84E-07 | 2.6643832 | up |
| CRLF1 | 9.32E-07 | 2.6629703 | up |
| RSF1 | 2.47E-07 | 2.6627014 | up |
| RLN2 | 2.23E-07 | 2.65354 | up |
| DPYSL3 | 3.03E-07 | 2.652405 | up |
| PTPRK | 6.52E-07 | 2.6524029 | up |
| CDK6 | 3.64E-07 | 2.6448061 | up |
| S100A3 | 6.85E-07 | 2.6418986 | up |
| lnc-AF131215,3,1-1 | 1.13E-06 | 2.640235 | up |
| UFL1 | 5.51E-07 | 2.6363554 | up |
| PIGK | 4.68E-07 | 2.6325972 | up |
| LRRFIP1 | 8.12E-07 | 2.6304867 | up |
| PCM1 | 7.77E-07 | 2.626667 | up |
| TMX4 | 8.11E-07 | 2.6176383 | up |
| TEAD3 | 2.26E-07 | 2.617048 | up |
| PIK3C2A | 3.33E-07 | 2.6137998 | up |
| TMEM59L | 1.03E-06 | 2.6134028 | up |
| SSC4D | 4.65E-07 | 2.6102698 | up |
| HSPA12A | 4.33E-07 | 2.6092787 | up |
| AIF1L | 3.41E-07 | 2.6075547 | up |
| MRAS | 8.24E-07 | 2.6064644 | up |
| MIDN | 3.22E-07 | 2.601707 | up |
| PLCXD1 | 3.48E-07 | 2.596672 | up |
| CTTNBP2NL | 2.84E-07 | 2.5956566 | up |
| FAM71E1 | 1.20E-06 | 2.593932 | up |
| FHOD3 | 9.18E-07 | 2.593166 | up |
| PRDM2 | 4.80E-07 | 2.5931232 | up |
| DZIP1 | 6.49E-07 | 2.5915496 | up |
| DAPK3 | 7.18E-07 | 2.588318 | up |
| RNF125 | 7.72E-07 | 2.5846367 | up |
| FBXO2 | 2.24E-07 | 2.5830402 | up |
| UBLCP1 | 3.36E-07 | 2.570388 | up |
| ATP5S | 1.00E-06 | 2.5692875 | up |
| KLF6 | 8.95E-07 | 2.56899 | up |
| KIAA1549L | 1.68E-07 | 2.567715 | up |
| ABCD4 | 3.64E-07 | 2.566488 | up |
| SCAF11 | 1.18E-06 | 2.5589247 | up |
| MAP3K9 | 1.34E-06 | 2.5575318 | up |
| OSTF1 | 1.40E-06 | 2.554572 | up |
| EVL | 1.82E-07 | 2.5521586 | up |
| ADAM10 | 6.29E-07 | 2.5519943 | up |
| SLC25A21 | 1.30E-06 | 2.5506265 | up |
| ST3GAL1 | 2.34E-07 | 2.549523 | up |
| SLC25A29 | 9.33E-07 | 2.5479326 | up |
| CAMK2N2 | 1.29E-06 | 2.5447814 | up |
| CLSTN1 | 9.10E-07 | 2.542677 | up |
| KIAA0586 | 7.40E-07 | 2.5412817 | up |
| IQGAP1 | 1.51E-07 | 2.540021 | up |
| SLC16A9 | 7.08E-07 | 2.5395808 | up |
| ANKRD6 | 1.25E-06 | 2.5363312 | up |
| TJP1 | 4.75E-07 | 2.5286157 | up |
| KRT19P2 | 4.23E-07 | 2.5259578 | up |
| MAPK1 | 2.04E-07 | 2.52372 | up |
| RASL11B | 9.29E-07 | 2.5168717 | up |
| RAMP1 | 3.01E-07 | 2.513626 | up |
| ZDHHC8 | 2.19E-07 | 2.5070555 | up |
| APLP2 | 2.25E-07 | 2.5029984 | up |
| ZDHHC17 | 9.86E-07 | 2.5026894 | up |
| TMEM217 | 9.14E-07 | 2.5011828 | up |
| FAM172A | 5.59E-07 | 2.5006533 | up |
| FAM110B | 5.59E-07 | 2.500578 | up |
| PTGER4 | 2.27E-07 | 2.5003114 | up |
| SLC38A1 | 4.79E-07 | 2.4943874 | up |
| KCNMB4 | 4.09E-07 | 2.4936163 | up |
| LINC00174 | 5.92E-07 | 2.4905581 | up |
| C14orf159 | 1.22E-06 | 2.4902039 | up |
| ZNF99 | 6.57E-07 | 2.4836433 | up |
| CAP1 | 2.81E-07 | 2.4829884 | up |
| PEX6 | 1.34E-06 | 2.4822872 | up |
| MEST | 7.41E-07 | 2.481925 | up |
| C18orf25 | 9.50E-07 | 2.4776058 | up |
| KIAA1522 | 6.50E-07 | 2.471384 | up |
| ZEB1 | 3.57E-07 | 2.4698706 | up |
| GUSB | 3.26E-07 | 2.4687235 | up |
| CCDC136 | 5.24E-07 | 2.4672923 | up |
| ST7-AS1 | 1.04E-06 | 2.4662588 | up |
| PTPLAD1 | 2.62E-07 | 2.4612541 | up |
| LRFN3 | 5.04E-07 | 2.4602149 | up |
| BOD1L1 | 4.65E-07 | 2.4597518 | up |
| BRK1 | 6.07E-07 | 2.4595745 | up |
| ZNF618 | 8.68E-07 | 2.4589221 | up |
| NAA35 | 2.22E-07 | 2.4578135 | up |
| C3orf52 | 5.48E-07 | 2.456706 | up |
| CDKN1C | 9.08E-07 | 2.4553516 | up |
| MICA | 1.81E-07 | 2.450044 | up |
| SLC44A2 | 6.57E-07 | 2.444976 | up |
| APPL1 | 7.53E-07 | 2.4448783 | up |
| EIF4G3 | 3.74E-07 | 2.4415827 | up |
| BIK | 1.43E-06 | 2.438854 | up |
| GLIDR | 9.62E-07 | 2.4365942 | up |
| ATP8A2 | 1.34E-06 | 2.4327462 | up |
| HOXA11 | 4.27E-07 | 2.4311004 | up |
| ATF3 | 1.07E-06 | 2.4251854 | up |
| ZNF605 | 2.62E-07 | 2.41795 | up |
| FAM117B | 1.06E-06 | 2.417527 | up |
| ABHD14A | 4.78E-07 | 2.4175224 | up |
| MICAL3 | 1.02E-06 | 2.4048 | up |
| MEX3C | 5.30E-07 | 2.402507 | up |
| BNIP3L | 2.93E-07 | 2.4016888 | up |
| VCL | 7.04E-07 | 2.4013543 | up |
| UNC13D | 6.09E-07 | 2.3999355 | up |
| FAM214A | 1.45E-06 | 2.3991187 | up |
| FSTL3 | 8.82E-07 | 2.397934 | up |
| ANKRD13A | 7.43E-07 | 2.3977506 | up |
| TMSB15B | 4.55E-07 | 2.3963528 | up |
| JMY | 8.01E-07 | 2.3905618 | up |
| TAPBP | 9.89E-07 | 2.3889189 | up |
| LOC728392 | 3.02E-07 | 2.3866923 | up |
| AKAP13 | 2.93E-07 | 2.3866065 | up |
| ZNF362 | 1.27E-06 | 2.3788266 | up |
| GANC | 1.16E-06 | 2.3761907 | up |
| BACE1 | 5.54E-07 | 2.3753405 | up |
| ARHGEF18 | 6.31E-07 | 2.3722713 | up |
| KLF3 | 1.15E-06 | 2.3720214 | up |
| ATRN | 7.19E-07 | 2.368798 | up |
| AGPAT4 | 7.57E-07 | 2.3634896 | up |
| FNDC3A | 5.16E-07 | 2.3619406 | up |
| FAM175A | 5.82E-07 | 2.3570328 | up |
| PHACTR2 | 9.04E-07 | 2.356921 | up |
| CDH2 | 1.31E-06 | 2.3566215 | up |
| RMDN1 | 1.00E-06 | 2.3520548 | up |
| TRAK1 | 3.63E-07 | 2.350425 | up |
| CCDC174 | 6.99E-07 | 2.3489494 | up |
| MAP4 | 1.02E-06 | 2.34866 | up |
| SVIL | 5.97E-07 | 2.3462398 | up |
| TMEM129 | 1.24E-06 | 2.343285 | up |
| RALA | 5.89E-07 | 2.3380904 | up |
| HMGXB4 | 3.46E-07 | 2.337165 | up |
| CCNB1IP1 | 6.24E-07 | 2.3316603 | up |
| HNRNPA0 | 2.77E-07 | 2.3284295 | up |
| TBC1D19 | 4.53E-07 | 2.3272235 | up |
| ME3 | 6.78E-07 | 2.32682 | up |
| SFN | 4.14E-07 | 2.326151 | up |
| SLC4A3 | 8.41E-07 | 2.3222291 | up |
| EDN2 | 8.97E-07 | 2.3217795 | up |
| HSPA5 | 8.50E-07 | 2.3138494 | up |
| NPHP1 | 1.05E-06 | 2.3111923 | up |
| CEP170B | 1.37E-06 | 2.3081017 | up |
| OS9 | 7.64E-07 | 2.3062282 | up |
| PLA2G12A | 1.21E-06 | 2.2911093 | up |
| ZNF286B | 6.86E-07 | 2.2894413 | up |
| C15orf52 | 4.23E-07 | 2.2873256 | up |
| MICU3 | 1.20E-06 | 2.286392 | up |
| CORO2A | 9.41E-07 | 2.2752678 | up |
| FKBP1B | 1.16E-06 | 2.2726493 | up |
| DGKA | 7.32E-07 | 2.2717962 | up |
| PVRL2 | 6.59E-07 | 2.2704952 | up |
| H2BFM | 1.19E-06 | 2.2678525 | up |
| IRS1 | 8.37E-07 | 2.2675698 | up |
| DECR1 | 9.86E-07 | 2.2675297 | up |
| RTF1 | 3.64E-07 | 2.2667096 | up |
| DSTN | 1.45E-06 | 2.266652 | up |
| CMTM3 | 4.71E-07 | 2.266203 | up |
| HSPG2 | 1.28E-06 | 2.2660558 | up |
| DIRAS1 | 8.41E-07 | 2.2646182 | up |
| CTPS2 | 5.28E-07 | 2.2641048 | up |
| PHIP | 5.00E-07 | 2.2639291 | up |
| MPDZ | 9.56E-07 | 2.263038 | up |
| C2orf69 | 1.19E-06 | 2.260717 | up |
| NT5E | 8.62E-07 | 2.2573645 | up |
| DIXDC1 | 6.69E-07 | 2.2544556 | up |
| SNX14 | 1.13E-06 | 2.2542112 | up |
| SUMF1 | 6.88E-07 | 2.2487614 | up |
| SMC1A | 9.22E-07 | 2.2463982 | up |
| ZNF649 | 1.42E-06 | 2.2427812 | up |
| ATG2B | 1.43E-06 | 2.238073 | up |
| LOXL2 | 1.13E-06 | 2.2365863 | up |
| NCOA7 | 7.13E-07 | 2.233341 | up |
| VCAN | 5.29E-07 | 2.2293448 | up |
| ZNF24 | 1.23E-06 | 2.2289438 | up |
| PICALM | 1.02E-06 | 2.2282329 | up |
| ZADH2 | 5.69E-07 | 2.2274017 | up |
| AGER | 1.12E-06 | 2.2253706 | up |
| RAB15 | 1.15E-06 | 2.2220757 | up |
| BCAT1 | 1.14E-06 | 2.2207427 | up |
| SETDB2 | 6.22E-07 | 2.2167666 | up |
| RCAN1 | 1.28E-06 | 2.2138865 | up |
| ANO10 | 9.13E-07 | 2.2073944 | up |
| TST | 9.00E-07 | 2.20661 | up |
| ZBTB21 | 8.56E-07 | 2.200605 | up |
| BAG1 | 1.14E-06 | 2.1983755 | up |
| TP53I3 | 6.55E-07 | 2.1963558 | up |
| LRRC49 | 1.38E-06 | 2.1939723 | up |
| BEGAIN | 9.30E-07 | 2.19384 | up |
| FAHD1 | 7.13E-07 | 2.1920888 | up |
| NUBPL | 6.52E-07 | 2.1892266 | up |
| MAP1B | 1.08E-06 | 2.1885138 | up |
| PRR5L | 7.20E-07 | 2.186886 | up |
| HIGD1A | 1.29E-06 | 2.175202 | up |
| CDCP1 | 8.08E-07 | 2.1630514 | up |
| ZNF729 | 1.10E-06 | 2.1623633 | up |
| ZNF254 | 9.82E-07 | 2.1619391 | up |
| TET2 | 1.23E-06 | 2.1538987 | up |
| TMEM242 | 1.43E-06 | 2.1454704 | up |
| TP53I3 | 1.16E-06 | 2.1443937 | up |
| YLPM1 | 9.43E-07 | 2.143995 | up |
| STARD4 | 9.13E-07 | 2.1416633 | up |
| BAG1 | 1.29E-06 | 2.1388125 | up |
| TMEM246 | 7.89E-07 | 2.1342063 | up |
| PPP1R13B | 1.10E-06 | 2.1118329 | up |
| AREL1 | 6.76E-07 | 2.1104183 | up |
| TCF7 | 7.53E-07 | 2.1097825 | up |
| KLHL25 | 6.85E-07 | 2.1090515 | up |
| PGPEP1 | 1.26E-06 | 2.1066396 | up |
| PDLIM7 | 1.05E-06 | 2.1051328 | up |
| YBEY | 7.59E-07 | 2.095948 | up |
| MOB1B | 8.36E-07 | 2.0939186 | up |
| MICAL3 | 1.19E-06 | 2.0922282 | up |
| AP1G2 | 1.19E-06 | 2.0916674 | up |
| FAHD2A | 1.28E-06 | 2.0908263 | up |
| CNTLN | 1.43E-06 | 2.0814054 | up |
| FAIM | 1.43E-06 | 2.0733757 | up |
| C17orf49 | 6.72E-07 | 2.0700378 | up |
| ZNF714 | 8.35E-07 | 2.0411067 | up |
| HDHD2 | 6.22E-07 | 2.038198 | up |
| LYSMD4 | 1.42E-06 | 2.036362 | up |
| RBM12 | 1.01E-06 | 2.0333502 | up |
| SRP14 | 1.02E-06 | 2.0326307 | up |
| ARSD | 1.42E-06 | 2.0320973 | up |
| DNPH1 | 1.25E-06 | 2.0316112 | up |
| C4orf46 | 1.00E-06 | 2.0303416 | up |
| CD9 | 1.05E-06 | 2.0198524 | up |
| AKAP2 | 1.13E-06 | 2.0190206 | up |
| SMC2 | 1.37E-06 | 2.012616 | up |
| EPHX2 | 1.36E-06 | 2.009087 | up |
