# Supplementary material for: Comparative Gene Expression Profiling of Primary and Metastatic Renal Cell Carcinoma Stem Cell-Like Cancer Cells
Source: PLoS One. 2016 Nov 3;11(11):e0165718. doi: 10.1371/journal.pone.0165718 (PMC5094751; doi:10.1371/journal.pone.0165718)
Supplement: S3 Table — (DOCX) [file pone.0165718.s005.docx]

**Supplementary table S3:** Common differentially (up-and down-regulated) expressed genes between CD105(Caki-2) and CD105(ACHN)

| GeneSymbol | FC (ASE Vs CD105 (ACHN)) | Regulation in CD105 (ACHN) | FC (ASE Vs CD105 (CAKI-2)) | Regulation in CD105 (CAKI-2) |
| --- | --- | --- | --- | --- |
| RGS10 | 2.0026596 | down | 2.2507463 | down |
| RFX7 | 2.0127137 | down | 2.7218928 | up |
| SUMF1 | 2.0154164 | down | 2.2487614 | up |
| BOD1L1 | 2.0332294 | down | 2.4597518 | up |
| UBE2L6 | 2.0742888 | down | 5.558738 | down |
| C7orf49 | 2.0769212 | down | 2.3114607 | down |
| NEK9 | 2.1021955 | down | 3.3534977 | up |
| JMY | 2.109487 | down | 2.3905618 | up |
| AKIP1 | 2.1176066 | down | 3.3256226 | down |
| TLCD1 | 2.1219707 | down | 5.0374656 | down |
| MT1F | 2.1395712 | down | 4.035997 | up |
| SPSB2 | 2.143123 | down | 4.2272196 | down |
| RNASEK | 2.1439276 | down | 2.9386148 | down |
| C10orf2 | 2.1443744 | down | 2.2302544 | down |
| C1orf43 | 2.1521454 | down | 2.437909 | down |
| ZEB1 | 2.162674 | down | 2.4698706 | up |
| PRIM1 | 2.1647704 | down | 2.3164098 | down |
| TMEM98 | 2.1974936 | down | 2224.5547 | up |
| CCDC169 | 2.2078364 | down | 2.9827483 | down |
| SULF2 | 2.215531 | down | 3.8757584 | up |
| SERTAD4 | 2.2313168 | down | 3.5272963 | down |
| HPS5 | 2.2609587 | down | 3.096604 | down |
| MAFG-AS1 | 2.2731557 | down | 5.1028867 | down |
| C7orf73 | 2.2879627 | down | 2.7115715 | up |
| AMZ2P1 | 2.3020935 | down | 4.462686 | down |
| EPHX2 | 2.3026352 | down | 2.009087 | up |
| ADM2 | 2.312137 | down | 3.33645 | up |
| MPDZ | 2.3151662 | down | 2.263038 | up |
| CTTNBP2 | 2.3172364 | down | 3.9438097 | up |
| ALDH3B1 | 2.3218772 | down | 2.3435466 | down |
| UNC5B-AS1 | 2.3292258 | down | 4.0296817 | up |
| SLC9A5 | 2.3380582 | down | 2.8125317 | down |
| ZNF605 | 2.3382463 | down | 2.41795 | up |
| NUBPL | 2.3546891 | down | 2.1892266 | up |
| CRNDE | 2.370482 | down | 3.3783467 | down |
| GMPR | 2.3750496 | down | 4.153266 | down |
| KLHDC4 | 2.3901024 | down | 2.7238872 | down |
| SNHG8 | 2.3910227 | down | 3.1651065 | down |
| EGR1 | 2.3923068 | down | 4.811161 | up |
| NLRC5 | 2.394491 | down | 2.6911895 | down |
| KIAA0408 | 2.3947024 | down | 3.5419888 | up |
| LPAR1 | 2.4125886 | down | 2.2070856 | down |
| HTATIP2 | 2.430165 | down | 2.9373877 | down |
| NT5C3A | 2.4360924 | down | 2.8973348 | down |
| SPR | 2.4441562 | down | 3.306882 | down |
| QPCT | 2.4575903 | down | 4.262102 | up |
| PCBD1 | 2.459206 | down | 3.059947 | down |
| RNF213 | 2.4649167 | down | 7.5874043 | down |
| RAB28 | 2.4680605 | down | 2.2136831 | down |
| COA6 | 2.4772127 | down | 4.4087405 | down |
| ARL4A | 2.482018 | down | 2.0069852 | down |
| HKDC1 | 2.4846523 | down | 2.2304163 | down |
| MRPS33 | 2.4893715 | down | 2.55131 | down |
| MDM4 | 2.4961135 | down | 2.3825064 | down |
| TRADD | 2.4963818 | down | 2.8986523 | down |
| FDXR | 2.5062284 | down | 2.482447 | down |
| CAPG | 2.5120707 | down | 4.250876 | down |
| TOMM7 | 2.5153592 | down | 2.262905 | down |
| DBNDD2 | 2.518014 | down | 5.937139 | up |
| C17orf67 | 2.5260406 | down | 3.831794 | down |
| ADAM10 | 2.5296829 | down | 2.7948713 | up |
| C7orf13 | 2.5566564 | down | 3.067436 | down |
| TBC1D2 | 2.5612104 | down | 2.4291048 | down |
| TMEM242 | 2.5694304 | down | 2.1454704 | up |
| FAM175A | 2.5745208 | down | 2.3570328 | up |
| GPRC5C | 2.5757816 | down | 4.6922913 | down |
| PIR | 2.5802946 | down | 8.104712 | down |
| FAM102A | 2.5864499 | down | 2.879419 | down |
| SNAPC5 | 2.588189 | down | 2.898294 | down |
| SNAPC5 | 2.59015 | down | 2.8863978 | down |
| SP140 | 2.5925255 | down | 6.5740333 | down |
| TLR1 | 2.613111 | down | 11.918064 | down |
| CYB561 | 2.623159 | down | 4.1695957 | down |
| MET | 2.6259527 | down | 2.3979535 | down |
| ARHGEF19 | 2.6286006 | down | 3.0134437 | down |
| FAM89A | 2.6529098 | down | 3.3194168 | down |
| MGMT | 2.691011 | down | 4.7225046 | down |
| RAB11FIP1 | 2.6973486 | down | 3.0856009 | up |
| H2AFJ | 2.7018871 | down | 2.2854254 | down |
| ACSL3 | 2.7041574 | down | 2.8651469 | up |
| HOXB4 | 2.714363 | down | 4.672986 | down |
| PHLDA3 | 2.7164083 | down | 2.8027303 | down |
| ATRX | 2.716793 | down | 2.6986032 | up |
| ASB9 | 2.7176237 | down | 7.453373 | down |
|  | 2.7316642 | down | 3.308024 | down |
| TRAK1 | 2.7449837 | down | 2.350425 | up |
| LRIG1 | 2.76753 | down | 3.1510222 | down |
| CDK19 | 2.7769382 | down | 2.7783766 | up |
| FAM117B | 2.792722 | down | 2.417527 | up |
| POLG2 | 2.7962213 | down | 2.0912476 | down |
| MYO5B | 2.8059762 | down | 7.2121925 | up |
| PRRG1 | 2.8199978 | down | 4.681301 | down |
| TRAM1 | 2.8245533 | down | 2.6638253 | down |
| DCLRE1A | 2.8369331 | down | 2.3066869 | down |
| OLFML2A | 2.854577 | down | 10.824901 | up |
| GUSB | 2.8568583 | down | 2.4687235 | up |
| IMPA2 | 2.8715763 | down | 3.2968497 | down |
| SLC25A37 | 2.875416 | down | 2.257747 | down |
| FBXL16 | 2.877874 | down | 5.6201844 | up |
| PCM1 | 2.879563 | down | 2.626667 | up |
| AVPI1 | 2.8939507 | down | 2.5560045 | down |
| TTC39A | 2.897109 | down | 4.5346565 | up |
| MPZL2 | 2.904044 | down | 6.8916874 | up |
| DBP | 2.9155815 | down | 5.543903 | down |
| LRRFIP1 | 2.9257126 | down | 3.516484 | up |
| CCDC148 | 2.9481487 | down | 3.515791 | down |
| COX15 | 2.9543197 | down | 2.3684204 | down |
| GDF15 | 2.9920907 | down | 3.715446 | down |
| ATF3 | 2.9938917 | down | 2.4251854 | up |
| GATM | 2.9985938 | down | 11.1491995 | up |
| FAM66A | 3.0012069 | down | 2.6928866 | down |
| PTPN13 | 3.0081205 | down | 3.3541055 | up |
| VPS13C | 3.0174656 | down | 4.6210723 | up |
| MAP3K14 | 3.0249195 | down | 6.1308312 | down |
| CD55 | 3.0312777 | down | 4.8166556 | down |
| NOL6 | 3.0317104 | down | 7.519188 | down |
| SCAF11 | 3.0384612 | down | 2.5589247 | up |
| KMT2E | 3.0393395 | down | 3.0089793 | up |
| PBX1 | 3.044785 | down | 3.8711665 | down |
| TMEM180 | 3.0501437 | down | 3.6401608 | down |
| EPB41L1 | 3.0593112 | down | 3.90106 | down |
| GEM | 3.0801306 | down | 2.9006426 | down |
| CCDC102A | 3.0837162 | down | 2.7303658 | down |
| GALNT18 | 3.0876515 | down | 4.065036 | down |
| SMAD6 | 3.0911283 | down | 2.8737679 | down |
| KCNIP1 | 3.0975797 | down | 4.5331373 | up |
| RGPD5 | 3.0985837 | down | 2.996143 | up |
| BCAT1 | 3.1065092 | down | 2.2207427 | up |
| IRAK1BP1 | 3.127303 | down | 2.685266 | up |
| C11orf1 | 3.137425 | down | 2.6173255 | down |
| IFI35 | 3.1393254 | down | 3.4667654 | down |
| USP40 | 3.1623533 | down | 5.656783 | down |
| CCDC144A | 3.1709273 | down | 22.244034 | up |
| DCDC2 | 3.1777427 | down | 97.89436 | up |
| CSNK2A2 | 3.1782928 | down | 2.5261097 | down |
| DOK7 | 3.1866403 | down | 11.676066 | up |
| NOL3 | 3.196138 | down | 3.4000604 | down |
| MDK | 3.202939 | down | 17.393423 | up |
| EEF1A2 | 3.2142 | down | 4.3302927 | down |
| BAZ2B | 3.2163599 | down | 6.3589463 | up |
| ADAP2 | 3.2179077 | down | 2.7697642 | down |
| BLOC1S2 | 3.228312 | down | 2.728631 | down |
| SAV1 | 3.2309644 | down | 2.7266166 | up |
| IGFLR1 | 3.2696724 | down | 3.8270597 | down |
| DPP9-AS1 | 3.3047688 | down | 7.7612824 | down |
| TRIM55 | 3.3167126 | down | 4.4411225 | down |
| TMF1 | 3.3211915 | down | 3.5087237 | up |
| ARAP3 | 3.3227556 | down | 3.7862291 | down |
| ANKRD6 | 3.3266418 | down | 2.5363312 | up |
| IER5L | 3.332363 | down | 4.628868 | up |
| PPAP2C | 3.3327 | down | 3.434713 | down |
| MPZL1 | 3.3449056 | down | 2.651038 | down |
| STAMBPL1 | 3.348862 | down | 10.692004 | down |
| SPNS2 | 3.3598585 | down | 15.223802 | up |
| LINC00857 | 3.3672218 | down | 5.684693 | down |
| SLC6A9 | 3.3713238 | down | 4.44373 | up |
| KATNAL2 | 3.4002411 | down | 3.8701582 | down |
| LOC100131564 | 3.4140694 | down | 2.9204023 | up |
| C2orf42 | 3.4384038 | down | 3.8485441 | down |
| LOC646762 | 3.4486768 | down | 2.1818302 | down |
| HCP5 | 3.4585483 | down | 2.9164822 | down |
| CYB5R2 | 3.4876533 | down | 3.7463527 | down |
| TRIM16L | 3.488036 | down | 5.1921334 | down |
| HEXIM2 | 3.492933 | down | 2.3685634 | down |
|  | 3.5000374 | down | 6.1448026 | down |
| CAV3 | 3.5271697 | down | 2.769074 | down |
| DOCK10 | 3.5365348 | down | 3.8748736 | down |
| COL16A1 | 3.5377645 | down | 2.7391982 | down |
| SNHG1 | 3.5507524 | down | 4.0181255 | down |
| MPC2 | 3.5546484 | down | 2.8130753 | down |
| PDE6B | 3.555956 | down | 4.606015 | up |
| FA2H | 3.559024 | down | 7.090825 | up |
| MYO5C | 3.567542 | down | 12.181686 | up |
| CYB5R2 | 3.577931 | down | 3.4960341 | down |
| RTP4 | 3.5862508 | down | 16.128202 | down |
| AZIN2 | 3.5893037 | down | 2.958189 | down |
| DLX2 | 3.612097 | down | 7.2136908 | down |
| EFNB3 | 3.634388 | down | 9.314001 | up |
| BRINP1 | 3.652001 | down | 4.1154747 | up |
| IFIT1 | 3.666249 | down | 2.7405732 | down |
| MFSD7 | 3.6731474 | down | 6.84386 | up |
|  | 3.710141 | down | 5.308851 | down |
| NCOA7 | 3.7124171 | down | 2.233341 | up |
| ZFAND2A | 3.7218864 | down | 6.737977 | down |
| LINC00923 | 3.7352154 | down | 9.539703 | down |
| MIF4GD | 3.7364984 | down | 2.5348384 | down |
| PIK3C2A | 3.792523 | down | 2.6137998 | up |
| SLC38A1 | 3.799482 | down | 2.4943874 | up |
| EPB41L1 | 3.8117378 | down | 2.8449667 | down |
| TSPAN33 | 3.8205469 | down | 2.4428635 | down |
| NPM2 | 3.8444836 | down | 4.090131 | up |
| ADAM8 | 3.85003 | down | 4.872669 | down |
| IMPACT | 3.864807 | down | 4.0000978 | up |
| GAB3 | 3.8662527 | down | 5.5513015 | down |
| TEAD4 | 3.8839526 | down | 2.9578173 | down |
| LINC01116 | 3.9360518 | down | 2.9734972 | down |
| SPATA7 | 3.9512339 | down | 3.0738425 | up |
| FAM63B | 3.9811363 | down | 4.7445827 | up |
| AZIN2 | 3.9841847 | down | 3.8237543 | down |
| RNF213 | 3.99604 | down | 10.130569 | down |
| FBLN5 | 4.0410943 | down | 9.942923 | up |
| ATP6V0E2-AS1 | 4.0494647 | down | 2.7160707 | down |
| PARP10 | 4.0498843 | down | 4.6481094 | down |
| PDE4A | 4.0509815 | down | 2.3372085 | down |
| LOC101927345 | 4.081176 | down | 49.18205 | down |
| NPHP1 | 4.096157 | down | 2.3111923 | up |
| NTF3 | 4.1031923 | down | 30.538769 | up |
| MAP3K15 | 4.1064286 | down | 3.929638 | down |
| ZGLP1 | 4.1179447 | down | 2.8113937 | up |
| BMP4 | 4.1194563 | down | 9.252166 | down |
| ZNF692 | 4.127923 | down | 2.3300395 | down |
| SGK2 | 4.147588 | down | 3.2832181 | down |
| SIRPG | 4.1482553 | down | 8.163807 | down |
| LINC00174 | 4.163276 | down | 2.4905581 | up |
| SLC26A11 | 4.175022 | down | 2.8099675 | down |
| HEBP1 | 4.1939225 | down | 2.9692836 | down |
| THY1 | 4.2060995 | down | 1122.9631 | up |
| C11orf70 | 4.2120137 | down | 2.5213163 | down |
| SARM1 | 4.240807 | down | 6.7689457 | up |
| NEURL3 | 4.2516522 | down | 2.6967802 | up |
| MORN4 | 4.2591133 | down | 2.4661725 | down |
| NAT8L | 4.264952 | down | 3.4187226 | down |
| IGFBP6 | 4.2713532 | down | 2.6048229 | down |
| TPP1 | 4.309736 | down | 6.3595347 | up |
| ADCY9 | 4.3115826 | down | 4.0905504 | down |
| RNF135 | 4.317709 | down | 2.036111 | down |
| KCNH2 | 4.323577 | down | 10.496634 | up |
| FGF2 | 4.3376427 | down | 3.520659 | down |
|  | 4.3664203 | down | 4.9639254 | up |
| FGF2 | 4.391761 | down | 3.42781 | down |
| MMAB | 4.3951836 | down | 2.790267 | up |
| TEX19 | 4.420746 | down | 10.471175 | down |
| PPP1R1C | 4.4517407 | down | 13.751307 | down |
| TMPRSS7 | 4.467377 | down | 3.3644772 | down |
| MGAT3 | 4.4929457 | down | 41.5374 | up |
| SUSD4 | 4.503987 | down | 4.5360336 | up |
| KLF2 | 4.5359826 | down | 5.245522 | down |
| SLC4A11 | 4.5817084 | down | 5.768609 | up |
| CD9 | 4.632782 | down | 2.0198524 | up |
| GPR39 | 4.6384954 | down | 3.7170022 | down |
| EPB41L4A | 4.6438336 | down | 2.6933844 | up |
| CPT1A | 4.661938 | down | 4.86068 | down |
| IL20RB | 4.6676135 | down | 54.34539 | down |
| METTL21A | 4.671471 | down | 2.4894984 | down |
| ITGBL1 | 4.706453 | down | 6.891749 | down |
| SEMA3B | 4.7084365 | down | 5.9524612 | up |
| RBM41 | 4.730296 | down | 3.2862477 | up |
| HLA-DPA1 | 4.749139 | down | 5.761582 | down |
| MEG3 | 4.750621 | down | 26.158632 | up |
| PLEKHB1 | 4.7620573 | down | 3.6532805 | down |
| TMEM79 | 4.77045 | down | 3.6262388 | down |
| ZNF655 | 4.7710114 | down | 2.8490658 | up |
| LOC644662 | 4.8571544 | down | 3.9763618 | down |
| PITX1 | 4.921071 | down | 1259.4725 | down |
| SORL1 | 4.931616 | down | 2.376103 | down |
| IFI44L | 4.946498 | down | 7.2048874 | down |
|  | 5.003912 | down | 5.1886787 | down |
| FLT3LG | 5.0355105 | down | 3.0727046 | down |
| TMEM229B | 5.0464454 | down | 6.7907305 | down |
| LOC388242 | 5.0532293 | down | 2.53966 | down |
| ZDHHC23 | 5.058918 | down | 4.182723 | down |
| RRAGD | 5.071825 | down | 9.744804 | up |
| LRRC75A | 5.0860167 | down | 2.6205044 | down |
| MSX1 | 5.1206384 | down | 5.596275 | down |
| HLA-DPB1 | 5.1267257 | down | 3.0280368 | down |
| IRS2 | 5.1394405 | down | 2.1979775 | down |
| SYNE2 | 5.191842 | down | 4.207934 | up |
| PLTP | 5.1957173 | down | 4.3971457 | down |
| TESC | 5.329006 | down | 5.9665666 | up |
| FOXL2 | 5.363645 | down | 25.725544 | up |
| MAGI2 | 5.399756 | down | 4.540322 | up |
| KCNJ8 | 5.4851093 | down | 2.7959607 | down |
| IFI44 | 5.488243 | down | 8.485934 | down |
| HAS3 | 5.6028976 | down | 15.276851 | down |
| KLF11 | 5.6137037 | down | 3.0603468 | down |
| PITX2 | 5.6177907 | down | 7.5270376 | down |
| SCG5 | 5.633117 | down | 2.333146 | down |
| LAMB3 | 5.761559 | down | 7.0079207 | down |
| RAB42 | 5.763748 | down | 9.200649 | down |
| SNCA | 5.799749 | down | 4.046579 | down |
| TNS3 | 5.8086157 | down | 2.5590963 | down |
| MSR1 | 5.8187857 | down | 6.440423 | down |
| ITGA1 | 5.8932886 | down | 7.523354 | up |
| TRIM36 | 5.9069033 | down | 3.5494087 | up |
| LOC399815 | 5.908022 | down | 3.839855 | down |
| TMCO4 | 5.946016 | down | 2.437807 | down |
| PROS1 | 5.9511786 | down | 4.1139493 | up |
| TLR3 | 5.954604 | down | 2.3195431 | down |
| ANKRD20A12P | 5.96342 | down | 63.203983 | down |
| BCL2 | 5.978878 | down | 9.736779 | up |
| SOCS3 | 5.98826 | down | 3.8092003 | down |
| CCDC88B | 5.9950647 | down | 7.77846 | down |
| GYG2 | 5.9961596 | down | 3.9337888 | down |
| XLOC_l2_003882 | 6.007137 | down | 5.0538197 | down |
| ADAMTS9 | 6.0315022 | down | 5.1827765 | up |
| PRDM8 | 6.041479 | down | 5.012612 | down |
| ROCK2 | 6.0478215 | down | 4.0236382 | up |
| EPAS1 | 6.0791893 | down | 3.224488 | down |
| CTSH | 6.085993 | down | 8.047358 | down |
| SORBS2 | 6.1114554 | down | 5.0155535 | up |
| C8orf46 | 6.141858 | down | 4.0896783 | down |
| LOC254896 | 6.2566648 | down | 8.568885 | down |
| TPK1 | 6.284525 | down | 12.383279 | down |
| GSTT2 | 6.2976756 | down | 9.670595 | down |
| NR1H3 | 6.3198576 | down | 4.4978304 | down |
| HKDC1 | 6.3234954 | down | 2.518596 | down |
| AKR1B1 | 6.337773 | down | 5.6352158 | down |
| CCDC88B | 6.3417006 | down | 8.86281 | down |
| lnc-GOLGA8J-3 | 6.3949986 | down | 2.871176 | down |
| FLVCR1-AS1 | 6.436513 | down | 11.111274 | down |
| DHRS3 | 6.4518347 | down | 8.701437 | down |
| SPINK13 | 6.4645286 | down | 51.228947 | down |
| HNF4G | 6.553965 | down | 7.3070226 | down |
| ZNF33B | 6.5585485 | down | 3.8375633 | up |
| ISCU | 6.6267533 | down | 2.772119 | down |
| PSMB9 | 6.631051 | down | 6.5239735 | down |
| FETUB | 6.642195 | down | 25.632988 | down |
| PDGFRL | 6.642305 | down | 7.125563 | down |
| LPHN2 | 6.700689 | down | 3.0569155 | up |
| FAM129A | 6.742299 | down | 2.0995064 | down |
| HS3ST1 | 6.7547383 | down | 2.804102 | down |
| XK | 6.7610207 | down | 5.28331 | down |
| JAKMIP3 | 6.8263645 | down | 7.990258 | down |
| FOXL2NB | 6.8471975 | down | 18.7658 | up |
| ANK3 | 6.9131074 | down | 4.2323484 | up |
| ELF3 | 6.9451365 | down | 3.70797 | down |
| ALDH3B1 | 6.951395 | down | 4.2344337 | down |
| AKR1C4 | 7.078494 | down | 35.627872 | down |
| TMEM116 | 7.1478763 | down | 2.4411376 | down |
| IFRD1 | 7.162051 | down | 5.2341886 | down |
| MAL2 | 7.198837 | down | 3.0671227 | down |
| SLC48A1 | 7.226373 | down | 3.841643 | down |
| CHRDL1 | 7.252875 | down | 73.7415 | up |
| SKAP1 | 7.295215 | down | 9.320079 | up |
| LACTB2 | 7.562068 | down | 4.0726056 | down |
|  | 7.670023 | down | 7.8090577 | down |
| GDPD3 | 7.6719894 | down | 5.6407604 | down |
| GOLGA8A | 7.7573943 | down | 6.0513825 | up |
| XLOC_l2_009136 | 7.899079 | down | 20.421412 | down |
| C10orf54 | 7.914843 | down | 6.765697 | down |
| SNHG12 | 7.924334 | down | 2.815954 | down |
| RARRES3 | 7.9290423 | down | 5.4270444 | down |
| SULT2B1 | 7.9316335 | down | 6.550812 | down |
| COBL | 7.9379745 | down | 28.073095 | up |
| HLA-DPB1 | 7.98352 | down | 4.252635 | down |
| CREG1 | 7.9990797 | down | 4.7324157 | down |
| IL22RA1 | 8.067759 | down | 11.036603 | down |
| LOC440028 | 8.125183 | down | 4.2641354 | down |
| HERC5 | 8.148515 | down | 10.389185 | down |
| RNLS | 8.1596365 | down | 3.3408759 | down |
| ENO3 | 8.314254 | down | 3.6171563 | up |
| KL | 8.331986 | down | 12.922984 | up |
| EMP1 | 8.363301 | down | 2.260079 | down |
| GPRC5B | 8.379993 | down | 3.6448293 | down |
| DHX58 | 8.487634 | down | 2.8946135 | down |
| MGST1 | 8.601858 | down | 4.8384037 | down |
| PKP2 | 8.613732 | down | 3.6508083 | down |
| CIART | 8.673841 | down | 4.790145 | down |
| C5orf58 | 8.6999855 | down | 22.129433 | down |
| CASP10 | 8.716641 | down | 6.9322915 | down |
| LOXL4 | 8.785339 | down | 2.8478727 | up |
| L1CAM | 8.847532 | down | 2.2287307 | down |
| NFIB | 8.869779 | down | 7.4396834 | down |
| SLC19A3 | 8.874515 | down | 8.376531 | down |
| PAPLN | 8.875075 | down | 12.331343 | up |
| BATF3 | 9.092173 | down | 3.1898663 | down |
| HLA-F | 9.093075 | down | 6.199139 | down |
| LMO1 | 9.130773 | down | 18.730963 | down |
| TOX2 | 9.199413 | down | 6.7536345 | down |
| DOCK11 | 9.227396 | down | 3.6559 | up |
| FIGNL2 | 9.280198 | down | 50.569675 | up |
| SOD2 | 9.328696 | down | 2.6605809 | down |
| SLC16A4 | 9.408968 | down | 2.6101964 | down |
| RAB3IL1 | 9.425829 | down | 8.498176 | down |
| ITGB8 | 9.463654 | down | 2.968328 | up |
| TMEM139 | 9.493153 | down | 3.6180472 | down |
| FAM90A1 | 9.538456 | down | 7.713357 | down |
| ENTPD1 | 9.646805 | down | 4.8451138 | down |
| C10orf11 | 9.651108 | down | 3.130437 | down |
| SQRDL | 9.653444 | down | 4.791069 | down |
| DTX2 | 9.744384 | down | 3.4539533 | down |
| NSUN7 | 9.748438 | down | 16.80503 | up |
| GPR160 | 9.890151 | down | 3.6347575 | down |
| AREG | 10.112617 | down | 4.2920012 | down |
| SLC48A1 | 10.129703 | down | 3.8738222 | down |
| EPN3 | 10.223694 | down | 9.339803 | down |
| TIMD4 | 10.245795 | down | 5.8004727 | down |
| PRSS16 | 10.312248 | down | 2.8007948 | down |
| AKR1C3 | 10.414461 | down | 51.346424 | down |
| FOXE1 | 10.502795 | down | 7.951796 | down |
| ZFYVE28 | 10.61009 | down | 10.009983 | down |
| CA2 | 10.653746 | down | 13.929842 | down |
| LAT2 | 10.711355 | down | 8.376555 | down |
| C4orf19 | 10.717884 | down | 7.7715077 | down |
| LINC-PINT | 10.746725 | down | 8.3891735 | down |
| IFIT2 | 10.856954 | down | 3.4710083 | down |
| NFIB | 10.882864 | down | 10.231256 | down |
| LAT | 10.927293 | down | 4.2533417 | down |
| GBP2 | 11.088305 | down | 3.3311555 | down |
| CHST9 | 11.17184 | down | 6.6346903 | down |
| PRODH | 11.278088 | down | 4.835586 | up |
| TIMD4 | 11.311499 | down | 6.3004155 | down |
| MANEAL | 11.386104 | down | 10.87538 | down |
| NECAB2 | 11.52723 | down | 5.3129115 | down |
| BCHE | 11.56983 | down | 4.021937 | down |
| TSPAN15 | 11.785295 | down | 7.3546715 | down |
| CASP8 | 11.787992 | down | 5.7850504 | down |
| TRGV7 | 11.872721 | down | 5.807063 | down |
| TNFRSF14 | 11.911657 | down | 3.149845 | down |
| GSTT2B | 12.008932 | down | 20.061398 | down |
| MEIG1 | 12.052906 | down | 8.773432 | down |
| C1orf186 | 12.16692 | down | 20.122213 | down |
| SHOX2 | 12.250553 | down | 14.893936 | down |
| IL7 | 12.301673 | down | 16.436995 | down |
| PTK2B | 12.397391 | down | 4.487769 | down |
| SLC13A3 | 12.43862 | down | 39.476933 | up |
| DAW1 | 12.475558 | down | 3.7520103 | down |
| HLA-DMA | 12.489492 | down | 9.822597 | down |
| CPLX1 | 12.663524 | down | 8.558904 | down |
| APBB1IP | 12.930275 | down | 22.131306 | down |
| MR1 | 13.077163 | down | 9.31652 | down |
| ALDH3B1 | 13.156933 | down | 6.2858057 | down |
| LOC100133669 | 13.615599 | down | 19.190052 | down |
| NFIB | 13.850648 | down | 8.903262 | down |
| PKP3 | 14.273475 | down | 7.5980988 | down |
| APBB1IP | 14.458037 | down | 24.469482 | down |
| TFPI | 14.7551775 | down | 6.9449887 | down |
| DCAF12L1 | 15.011521 | down | 16.487652 | down |
| MNX1 | 15.098404 | down | 5.265464 | up |
| GCNT3 | 15.109649 | down | 10.317641 | down |
| GSAP | 15.652812 | down | 4.3626313 | down |
| HAVCR1 | 15.679779 | down | 13.620075 | down |
| BIRC3 | 15.7442045 | down | 3.5718348 | down |
| CASP4 | 16.106361 | down | 6.3228006 | down |
| C15orf48 | 16.15271 | down | 9.661258 | down |
| B4GALNT1 | 16.193726 | down | 26.164381 | down |
| NID2 | 16.234047 | down | 3.7860358 | up |
| SPX | 16.425356 | down | 50.30046 | down |
| HGD | 16.60722 | down | 9.054109 | down |
| CASP5 | 16.637117 | down | 6.008223 | down |
| CXCL1 | 16.913652 | down | 5.690961 | down |
| C17orf67 | 17.064177 | down | 21.97195 | down |
| CLU | 17.349243 | down | 6.6612124 | down |
| HLA-DPB1 | 17.471207 | down | 6.567733 | down |
| lnc-RP3-377D14,1,1-3 | 17.547754 | down | 55.049667 | down |
| TM7SF3 | 17.56024 | down | 3.4405632 | down |
| PRAME | 18.195536 | down | 17.799816 | down |
| ABCC3 | 18.27827 | down | 6.070709 | down |
| ADAMTS18 | 18.360664 | down | 14.181519 | down |
| SLC3A1 | 18.656675 | down | 5.4883556 | down |
| CYP3A7 | 18.737495 | down | 7.4772515 | down |
| ULK4 | 18.977495 | down | 3.0912142 | down |
| SPX | 19.498913 | down | 25.100727 | down |
| EDN2 | 19.760836 | down | 2.3217795 | up |
| ANKRD2 | 20.26199 | down | 7.220253 | down |
| SOWAHD | 20.529434 | down | 8.893701 | down |
|  | 20.716383 | down | 25.229933 | down |
| OAS1 | 21.257868 | down | 15.8977375 | down |
| FOXA1 | 21.397179 | down | 18.105501 | down |
| CXCL1 | 21.641228 | down | 5.5978923 | down |
| IRF5 | 21.875374 | down | 4.07368 | up |
| C1R | 23.079014 | down | 20.6178 | down |
| DPYD | 23.314764 | down | 3.5149128 | down |
| WBSCR27 | 23.380804 | down | 4.369386 | down |
| C1R | 23.878038 | down | 17.357187 | down |
| VNN2 | 24.220984 | down | 5.2779293 | down |
| SCIN | 24.27898 | down | 9.646683 | down |
| SYTL2 | 24.595179 | down | 3.7329445 | down |
| CRIP1 | 24.74916 | down | 4.1805825 | up |
| FAM110C | 24.77161 | down | 93.70035 | up |
| SLC47A1 | 25.030191 | down | 5.984535 | down |
| WFDC21P | 25.306934 | down | 39.593243 | down |
| PDCD4-AS1 | 25.439539 | down | 2.8849719 | down |
| TFPI | 26.052132 | down | 30.63894 | down |
| HLA-DPA1 | 26.802366 | down | 30.001682 | down |
| SPIRE2 | 26.87499 | down | 7.743881 | down |
|  | 27.042137 | down | 22.767372 | down |
| TRABD2A | 27.06367 | down | 24.076899 | down |
| PLEKHG4 | 28.08761 | down | 14.570255 | down |
| PPFIBP2 | 28.153555 | down | 31.48052 | down |
| C6orf58 | 30.750689 | down | 10.41306 | down |
| DNAJC12 | 31.187342 | down | 24.55709 | down |
| DDIT3 | 31.892427 | down | 4.1937604 | down |
| IFI16 | 32.008144 | down | 11.526948 | down |
| ITGB4 | 32.054356 | down | 3.3703237 | down |
| PLEKHG4 | 33.406746 | down | 19.694304 | down |
| TNFSF14 | 33.620117 | down | 12.393551 | down |
| EREG | 33.7053 | down | 119.66313 | down |
| DLX1 | 34.07825 | down | 35.022217 | down |
| THNSL2 | 34.141483 | down | 12.17379 | down |
| PRKCE | 35.216515 | down | 10.429932 | down |
| TMEM130 | 35.508244 | down | 25.743525 | down |
| DDO | 35.57625 | down | 5.866687 | down |
| LOC388282 | 36.30359 | down | 4.5387673 | down |
| STAT4 | 36.54354 | down | 6.7688875 | down |
| TMEM255A | 37.44646 | down | 8.898723 | down |
| RHBDL2 | 38.664875 | down | 49.803474 | down |
| MANSC1 | 40.97112 | down | 10.512441 | down |
| SOX2 | 43.4763 | down | 9.518933 | down |
| C3 | 43.492958 | down | 4.1678386 | down |
| CAPS2 | 45.277485 | down | 9.263916 | down |
| C8orf31 | 47.395653 | down | 14.291888 | down |
| CRIP1 | 47.483253 | down | 19.365059 | up |
| SAA4 | 47.88241 | down | 82.1694 | down |
| FGFR2 | 48.616947 | down | 9.257834 | down |
| SAA2 | 49.860035 | down | 443.8247 | down |
| TSPAN8 | 51.02195 | down | 10.272833 | down |
| FABP6 | 52.342274 | down | 19.679903 | down |
| C3 | 53.363293 | down | 4.9307427 | down |
| SECTM1 | 56.331234 | down | 45.566254 | down |
| IL6 | 57.337566 | down | 4.3486967 | down |
| CXCL2 | 60.913734 | down | 8.440975 | down |
| HOXD13 | 62.76816 | down | 6.487314 | down |
| SOD3 | 62.97636 | down | 10.509057 | down |
| PDZK1IP1 | 63.04496 | down | 17.852224 | down |
| FGFR2 | 69.97136 | down | 21.828024 | down |
| ALDH1A1 | 76.811646 | down | 14.318216 | down |
| SMTNL2 | 77.41427 | down | 8.283728 | up |
| CXCL8 | 81.80528 | down | 5.3475456 | down |
| TFAP2A | 84.66701 | down | 89.26121 | down |
| MAPT | 88.10559 | down | 7.830533 | down |
| EFHB | 94.08367 | down | 14.906687 | down |
| MSR1 | 94.83834 | down | 38.73383 | down |
| HOXA13 | 101.23336 | down | 178.23175 | down |
| SLFN11 | 105.37484 | down | 81.23137 | down |
| CARD17 | 108.63285 | down | 281.20456 | down |
| SAMD9L | 111.32013 | down | 27.159569 | down |
| C1S | 120.72365 | down | 23.200611 | down |
| IL18 | 123.006905 | down | 40.215225 | down |
| SAA2 | 123.27198 | down | 103.14898 | down |
| PPP2R2C | 128.22264 | down | 20.149107 | down |
| SLC47A1 | 133.75429 | down | 13.08543 | down |
| QRFPR | 135.95412 | down | 11.16069 | down |
| ACKR4 | 151.2928 | down | 114.31113 | down |
| RHBDL2 | 162.57365 | down | 61.418987 | down |
| TBX15 | 167.008 | down | 479.57794 | down |
| CCM2L | 170.88846 | down | 64.18205 | down |
| SAA1 | 175.55038 | down | 1030.5543 | down |
| CXCL2 | 212.30475 | down | 20.4736 | down |
| TM4SF18 | 220.66495 | down | 385.1344 | down |
| SP5 | 251.61511 | down | 57.23676 | down |
| PLEK | 375.0206 | down | 13.031936 | down |
| AFAP1-AS1 | 375.07092 | down | 1256.9475 | down |
| DDC | 375.71884 | down | 9.328867 | down |
| NUPR1 | 451.8799 | down | 49.677166 | down |
| CASP1 | 522.1344 | down | 410.08685 | down |
| CDH1 | 544.15845 | down | 6.4238524 | up |
| WISP2 | 581.8608 | down | 99.078156 | down |
| HOXB13 | 635.8253 | down | 237.4759 | down |
| C1S | 644.703 | down | 87.05888 | down |
| IGFN1 | 748.5548 | down | 16.630629 | down |
| ITGB2 | 1037.9517 | down | 6.9944763 | down |
| ALDH1A1 | 1633.7367 | down | 268.71854 | down |
| LCN2 | 11514.873 | down | 23.906965 | down |
| POMT2 | 2.0097785 | up | 3.5646732 | up |
| MCAM | 2.0114324 | up | 6.762767 | up |
| SRP14 | 2.0117507 | up | 2.0326307 | up |
| SVIL | 2.0305574 | up | 2.3462398 | up |
| BRK1 | 2.0317326 | up | 2.8529 | up |
| HSPA14 | 2.0576508 | up | 2.7078032 | down |
| AOX1 | 2.0587862 | up | 3.727258 | down |
| CDCP1 | 2.0596771 | up | 2.1630514 | up |
| BRK1 | 2.0605273 | up | 2.4595745 | up |
| PRICKLE2 | 2.0605998 | up | 6.83314 | up |
| HSPB11 | 2.0860088 | up | 2.167378 | down |
| EBPL | 2.1372187 | up | 2.0259852 | down |
| NOTCH2 | 2.141511 | up | 2.0772824 | down |
| ICAM3 | 2.1449485 | up | 2.0450277 | down |
| MEIS2 | 2.145523 | up | 3.4338396 | up |
| TMCC1 | 2.1517873 | up | 3.0500917 | up |
|  | 2.1588256 | up | 3.0635035 | down |
| BEAN1 | 2.1608405 | up | 3.5826313 | up |
| COX17 | 2.1656651 | up | 2.3436687 | down |
| MRPL3 | 2.1677961 | up | 2.3990233 | down |
| MIDN | 2.1784623 | up | 2.601707 | up |
| NAA50 | 2.1825058 | up | 3.0623002 | down |
| VDAC1 | 2.1851273 | up | 2.0822523 | down |
| FN3KRP | 2.1872075 | up | 2.0994453 | down |
| SNHG18 | 2.1966884 | up | 326.0694 | up |
| FSCN1 | 2.20144 | up | 3.5262778 | up |
|  | 2.2023993 | up | 2.3452692 | up |
| EXOSC4 | 2.2139416 | up | 2.129417 | down |
| SIX1 | 2.222749 | up | 3.2700293 | up |
| GINS2 | 2.2258794 | up | 2.798236 | down |
| C8orf59 | 2.2320073 | up | 2.3918161 | down |
| WLS | 2.2419894 | up | 2.7076578 | up |
| WWTR1 | 2.2595193 | up | 4.7868667 | down |
| LPAR2 | 2.2649171 | up | 8.386959 | up |
|  | 2.2734618 | up | 12.244665 | up |
| PPP1R13L | 2.3104463 | up | 2.7616389 | up |
| SEPT11 | 2.3173952 | up | 2.8460238 | up |
| ZFYVE1 | 2.3182645 | up | 3.391597 | up |
| P3H2 | 2.3285193 | up | 2.2089925 | down |
| MAP4 | 2.328881 | up | 2.34866 | up |
| CYC1 | 2.3289888 | up | 2.5368183 | down |
| ZNF580 | 2.331911 | up | 2.7729654 | up |
| TMEM154 | 2.342406 | up | 3.583478 | up |
|  | 2.345172 | up | 2.3099957 | down |
| VASH1 | 2.3476274 | up | 11.882957 | up |
| CST3 | 2.3539095 | up | 3.3145614 | up |
| TP53I3 | 2.3599093 | up | 2.1963558 | up |
| EBPL | 2.370049 | up | 2.106594 | down |
| ZBTB7B | 2.3732247 | up | 3.9080977 | down |
| C4orf46 | 2.375443 | up | 2.0303416 | up |
| PRR5L | 2.3820848 | up | 2.186886 | up |
| CGN | 2.3856013 | up | 5.035107 | up |
| PSMD12 | 2.3908195 | up | 2.2372622 | down |
| CTPS2 | 2.4050183 | up | 2.2641048 | up |
| RPS23 | 2.409151 | up | 10.880847 | up |
| TP53I3 | 2.422786 | up | 2.1443937 | up |
| COL4A2 | 2.4347057 | up | 3.7134848 | up |
| SH3BGRL2 | 2.4393127 | up | 3.3637974 | up |
| ANO10 | 2.4624665 | up | 2.2073944 | up |
| FOSL1 | 2.475023 | up | 4.766869 | down |
| TCEB1 | 2.4873083 | up | 2.3197815 | down |
| DOPEY2 | 2.4902108 | up | 2.8443267 | up |
|  | 2.4945397 | up | 10.127399 | up |
| DEF6 | 2.5134234 | up | 5.2057943 | up |
| SLC26A6 | 2.5274286 | up | 3.736733 | up |
| ERAP2 | 2.5415146 | up | 2.9368324 | up |
| LOC158435 | 2.5424383 | up | 2.1121042 | down |
| TROAP | 2.5543172 | up | 2.6454282 | down |
| TIPIN | 2.555 | up | 2.3917146 | down |
| ACAA2 | 2.5566304 | up | 3.5575554 | up |
| MRGBP | 2.572989 | up | 2.5597444 | down |
| CDCA3 | 2.5767012 | up | 2.37204 | down |
| TRPC4 | 2.5902414 | up | 3.8753974 | up |
| SLC25A23 | 2.5914352 | up | 3.8940806 | up |
| FOXP1 | 2.5980067 | up | 5.7647285 | up |
| ARFIP2 | 2.6182241 | up | 2.2202206 | down |
| CSNK1D | 2.620531 | up | 2.5169942 | down |
| FUT8 | 2.6211698 | up | 2.9825153 | up |
| HMGA2 | 2.6246393 | up | 3.2139518 | down |
| CST5 | 2.6392472 | up | 3.4119034 | up |
| PYCRL | 2.647077 | up | 2.485483 | down |
| CEP170B | 2.6472678 | up | 2.3081017 | up |
| CERCAM | 2.6545622 | up | 9.963496 | up |
| EID3 | 2.679765 | up | 4.2746468 | down |
| KIAA0101 | 2.683779 | up | 2.5056648 | down |
| SELM | 2.6905863 | up | 3.1271255 | down |
| lnc-ANP32A-3 | 2.693881 | up | 4.065538 | up |
| SAMD14 | 2.6955605 | up | 8.537498 | up |
| PKN3 | 2.7178948 | up | 2.8480165 | up |
| FAM27C | 2.7265968 | up | 2.2851343 | down |
| LOC645166 | 2.72964 | up | 3.8758771 | down |
| VEGFC | 2.7312112 | up | 2.3032913 | down |
| DCAF13 | 2.7331586 | up | 2.3729959 | down |
| KRT19P2 | 2.7360075 | up | 2.5259578 | up |
| SALL1 | 2.7415524 | up | 3.7769434 | up |
| JUP | 2.7451167 | up | 7.3681836 | up |
| UNC13B | 2.7487655 | up | 3.2660685 | up |
| SNAI3-AS1 | 2.753163 | up | 4.610768 | up |
| MAPK13 | 2.756279 | up | 4.9808187 | up |
| VMA21 | 2.7596042 | up | 2.569525 | down |
| STARD10 | 2.760053 | up | 3.7934794 | up |
| PDLIM5 | 2.77833 | up | 3.7097178 | up |
| TPM1 | 2.7996948 | up | 3.3687663 | up |
| PTER | 2.8034422 | up | 3.4994164 | down |
| FCRLB | 2.8419507 | up | 2.5309446 | down |
| FZD7 | 2.849549 | up | 5.195226 | up |
| DCAF13 | 2.8508449 | up | 2.5825877 | down |
| HAUS7 | 2.8606486 | up | 2.1246767 | down |
| SLC38A10 | 2.8661785 | up | 2.8505487 | down |
| SLC1A1 | 2.869042 | up | 2.673368 | up |
| CDCA5 | 2.8710928 | up | 2.2099617 | down |
| TPM1 | 2.8720036 | up | 3.7196512 | up |
| IRS1 | 2.8801348 | up | 2.2675698 | up |
| TMEM132A | 2.8813643 | up | 3.3244803 | up |
| BOK | 2.8861506 | up | 3.022375 | up |
| DCAF13P3 | 2.902856 | up | 2.6572928 | down |
| LOC653602 | 2.9070475 | up | 10.270805 | up |
| MPP1 | 2.9076104 | up | 2.8112016 | down |
| MYRF | 2.9121172 | up | 3.4404628 | up |
| C11orf52 | 2.918595 | up | 2.9079146 | up |
| PRKCD | 2.9226844 | up | 3.5778732 | up |
| GBP1 | 2.9395995 | up | 10.080206 | up |
| CDKN1C | 2.9477897 | up | 2.4553516 | up |
| HOXA11 | 2.9640448 | up | 2.4311004 | up |
| EMC10 | 2.969257 | up | 3.4965165 | up |
|  | 2.9702923 | up | 3.1794205 | down |
| SUV39H1 | 2.9923992 | up | 2.2370255 | down |
| MAPK13 | 3.0090353 | up | 12.593763 | up |
| PLGRKT | 3.0195382 | up | 2.9213848 | up |
| FBLL1 | 3.0230365 | up | 10.434098 | up |
| DSTN | 3.0499392 | up | 2.266652 | up |
| PLEKHG2 | 3.0504005 | up | 3.0769572 | up |
| NANOS3 | 3.0797353 | up | 7.4668083 | up |
| FCHO1 | 3.0847392 | up | 11.921249 | down |
| SIX4 | 3.0909772 | up | 6.921992 | up |
| LOC101928076 | 3.0918624 | up | 16.128948 | up |
| SSC4D | 3.1209803 | up | 2.6102698 | up |
| HIP1R | 3.1297462 | up | 4.1627903 | up |
| TEAD3 | 3.132882 | up | 2.617048 | up |
| SNN | 3.1361153 | up | 2.7709453 | up |
| TNFAIP3 | 3.1389966 | up | 6.7576637 | up |
| NACAD | 3.1554646 | up | 18.29835 | up |
| EP400NL | 3.1564713 | up | 3.4390454 | down |
| LYPD1 | 3.1595042 | up | 23.681416 | up |
| KALRN | 3.2424393 | up | 3.211568 | up |
| JAM2 | 3.242755 | up | 13.804123 | up |
| BIRC5 | 3.249363 | up | 3.0416696 | down |
| RABGAP1L | 3.2745435 | up | 9.468573 | up |
| SOCS2 | 3.2969346 | up | 17.578514 | up |
| AURKAPS1 | 3.3173618 | up | 2.206454 | down |
| KRT15 | 3.3265827 | up | 4.95596 | down |
| DKK3 | 3.3524218 | up | 4.0232573 | up |
| MICA | 3.3559973 | up | 2.450044 | up |
| SPTBN1 | 3.3837893 | up | 2.807989 | up |
| BCOR | 3.3844535 | up | 3.2412128 | down |
| KCNMA1 | 3.4073048 | up | 18.1771 | up |
| KBTBD11 | 3.4131155 | up | 29.247786 | up |
| FBLN1 | 3.414735 | up | 10.568066 | up |
| CHKB | 3.4197845 | up | 4.90299 | up |
| CDH2 | 3.4198434 | up | 2.8768902 | up |
| LOC102723456 | 3.4334993 | up | 28.399529 | up |
| RTKN2 | 3.4344504 | up | 7.0263834 | up |
| CSRP2 | 3.451917 | up | 4.3018184 | up |
| TMEM25 | 3.467809 | up | 90.997215 | up |
| PRADC1 | 3.4742098 | up | 2.5009758 | down |
| HTRA1 | 3.4854574 | up | 2.8970704 | up |
| LYPD1 | 3.4869063 | up | 28.385338 | up |
| CRYAB | 3.4883466 | up | 5.1330366 | up |
| MYCL | 3.5296535 | up | 15.850955 | up |
| CLIC4 | 3.535522 | up | 3.0798624 | up |
| HMGCR | 3.5433326 | up | 4.6554656 | up |
| SPINT1 | 3.5460992 | up | 32.938213 | up |
| TMEM201 | 3.5534081 | up | 2.0805554 | down |
| WNT5B | 3.5676954 | up | 34.16403 | up |
| DLST | 3.5852375 | up | 3.0811183 | up |
| CDIP1 | 3.5924044 | up | 3.274401 | up |
| SPSB1 | 3.6206152 | up | 4.311218 | up |
| CDH3 | 3.6218455 | up | 2.999308 | up |
| SPSB1 | 3.6456227 | up | 3.7417276 | up |
| PCGF3 | 3.6657043 | up | 5.137052 | down |
| MMP23B | 3.680381 | up | 4.195893 | up |
| CECR6 | 3.6822972 | up | 21.739157 | up |
| MICAL1 | 3.708232 | up | 4.238294 | up |
| PDLIM4 | 3.7221303 | up | 209.65633 | up |
| FNIP2 | 3.7270012 | up | 4.113732 | up |
| AGRN | 3.744726 | up | 3.6019704 | up |
| ARID5B | 3.7652512 | up | 5.3758206 | up |
| MICALCL | 3.7779262 | up | 4.5468516 | up |
| NAA35 | 3.7817452 | up | 2.4578135 | up |
| C3orf52 | 3.786085 | up | 2.456706 | up |
| SCGB3A2 | 3.786699 | up | 2.890477 | up |
| NKAIN4 | 3.8130836 | up | 155.62561 | up |
| LSP1 | 3.820023 | up | 2.663609 | down |
| DSCC1 | 3.8409297 | up | 2.569202 | down |
| ARHGAP44 | 3.8436997 | up | 78.255646 | up |
| TMEM217 | 3.8686664 | up | 2.5011828 | up |
| EVL | 3.881475 | up | 2.5521586 | up |
| ABCB4 | 3.9091005 | up | 12.118003 | up |
| LRFN4 | 3.9492154 | up | 468.8585 | up |
| RECQL5 | 3.9521632 | up | 3.7145913 | down |
| PRSS22 | 3.9734178 | up | 2.9650705 | up |
| SSSCA1 | 3.9799576 | up | 2.235285 | down |
| DNAJA4 | 3.9908173 | up | 14.830475 | up |
| CD59 | 3.9965572 | up | 3.4952273 | up |
| HIST1H4C | 4.0092206 | up | 2.640355 | down |
| ZDHHC8 | 4.0140777 | up | 3.678205 | up |
| PNPLA3 | 4.015095 | up | 3.0281022 | up |
| TOMM40 | 4.01643 | up | 2.2477067 | down |
| ARSJ | 4.016568 | up | 3.9268909 | up |
| CTNNB1 | 4.0326757 | up | 3.467719 | up |
| NKAIN1 | 4.0365987 | up | 4.6144667 | up |
| CDH2 | 4.047875 | up | 2.3566215 | up |
| SIX2 | 4.056176 | up | 3.986483 | up |
| MMP15 | 4.058387 | up | 4.129373 | up |
| LYSMD4 | 4.086686 | up | 2.036362 | up |
| TOMM40 | 4.0986605 | up | 2.2208774 | down |
| IGSF9B | 4.1080394 | up | 3.074807 | up |
| MYH9 | 4.1189575 | up | 5.54366 | up |
| SACS | 4.1234527 | up | 4.054675 | up |
| ARHGEF18 | 4.1277137 | up | 2.3722713 | up |
| PRSS23 | 4.1327114 | up | 6.962133 | up |
| ARL10 | 4.1501017 | up | 25.037176 | up |
| TNS1 | 4.190632 | up | 23.859894 | up |
| RGS9 | 4.1908574 | up | 2.9107494 | down |
| E2F1 | 4.201539 | up | 3.6099818 | down |
| ACTG1P4 | 4.226524 | up | 3.367074 | down |
| HS3ST3A1 | 4.2319283 | up | 314.4561 | up |
| CYP17A1-AS1 | 4.253999 | up | 2.9799106 | up |
| CLTB | 4.2669163 | up | 2.079056 | down |
| VCL | 4.2869525 | up | 2.4013543 | up |
| ARID3A | 4.302092 | up | 9.171965 | up |
| LOC151174 | 4.312176 | up | 3.1586847 | up |
| STAP2 | 4.3244805 | up | 3.9417145 | up |
| SAMD4A | 4.352122 | up | 3.2020864 | up |
| RIC1 | 4.35525 | up | 3.5442965 | up |
| PID1 | 4.3655486 | up | 3.0143952 | down |
| LHX1 | 4.4148293 | up | 17.161444 | up |
| SAMD4A | 4.430562 | up | 2.9269104 | up |
| ITM2C | 4.4408407 | up | 4.319741 | up |
| NOC2L | 4.5411983 | up | 2.085881 | down |
| ARHGAP27 | 4.580292 | up | 2.8036168 | up |
| PARD6G | 4.580343 | up | 8.542819 | up |
| SLIT3 | 4.6387753 | up | 47.03961 | up |
| CORO2B | 4.6527224 | up | 7.002093 | up |
| CTNND2 | 4.658625 | up | 3.8199415 | up |
| HYAL3 | 4.672535 | up | 2.7606595 | up |
| SORCS2 | 4.68787 | up | 3.9391928 | up |
| FOXN3 | 4.693179 | up | 3.5112922 | up |
| CLEC11A | 4.713565 | up | 6.049174 | up |
| HES4 | 4.725362 | up | 11.66832 | up |
| IGFBP4 | 4.7431884 | up | 9.476856 | up |
| LRRFIP1 | 4.789611 | up | 3.4002304 | up |
| ST6GAL1 | 4.8054233 | up | 4.6823745 | up |
| LOC100132356 | 4.8057766 | up | 3.1006708 | down |
| KIF3C | 4.841147 | up | 3.6110742 | up |
| ABCA1 | 4.8520083 | up | 19.871485 | up |
| PLA2G4C | 4.881009 | up | 5.473066 | up |
| SNORA2A | 4.882843 | up | 3.5488102 | down |
| AGMAT | 4.8850965 | up | 3.3950512 | up |
| SLC2A6 | 4.906748 | up | 4.397283 | up |
| BEX5 | 4.9108186 | up | 83.80385 | up |
| RINL | 4.913448 | up | 4.2827215 | up |
| FOXP1 | 4.913523 | up | 3.4981856 | up |
| DAPK3 | 4.9247923 | up | 2.588318 | up |
| GFPT2 | 4.9556856 | up | 12.641689 | down |
| CAP1 | 4.980074 | up | 2.4829884 | up |
| ENC1 | 4.9805956 | up | 3.6957157 | up |
| SCARNA12 | 5.015455 | up | 4.034454 | down |
| TGFBR1 | 5.024669 | up | 6.8268642 | up |
| FLNB | 5.116086 | up | 4.2444553 | up |
| CDC42 | 5.1188955 | up | 4.317669 | down |
| EGR3 | 5.1311274 | up | 21.092382 | up |
| INSIG1 | 5.143347 | up | 6.7059097 | up |
| LOC101929494 | 5.18307 | up | 2.3997319 | down |
| DNAJB5 | 5.2082634 | up | 3.1446989 | up |
| COL9A3 | 5.218057 | up | 4.767984 | up |
| NQO2 | 5.2357726 | up | 2.0626488 | down |
| RPL39L | 5.2708173 | up | 2.1468034 | down |
| NREP | 5.3130846 | up | 6.2461996 | up |
| PGM2L1 | 5.3542786 | up | 10.720828 | up |
| LOC100131289 | 5.3813515 | up | 3.8785472 | up |
| JAM2 | 5.399533 | up | 9.126855 | up |
| KLHL25 | 5.4371605 | up | 2.1090515 | up |
| RASSF10 | 5.437661 | up | 8.204376 | down |
| VWCE | 5.4833984 | up | 7.4009776 | up |
| BVES | 5.4847794 | up | 3.2833595 | up |
| PLXNA3 | 5.5038924 | up | 4.826485 | up |
| HOXA11-AS | 5.521415 | up | 4.2847137 | up |
| PTPRK | 5.555767 | up | 2.6524029 | up |
| FBLN1 | 5.5831923 | up | 6.852562 | up |
| ARHGAP31 | 5.599137 | up | 3.645004 | up |
| ARHGAP23 | 5.62295 | up | 2.390567 | down |
| BEGAIN | 5.6363807 | up | 2.19384 | up |
| JAM2 | 5.6403008 | up | 8.821118 | up |
| NKAIN4 | 5.6455336 | up | 633.0603 | up |
| ADAM12 | 5.689684 | up | 24.870712 | up |
| SNAI1 | 5.7122326 | up | 4.439304 | down |
| DEF8 | 5.791333 | up | 2.3712823 | down |
| MEX3A | 5.816036 | up | 11.5382 | up |
| PLEKHG3 | 5.8325663 | up | 3.9025939 | up |
| GAL | 5.8332787 | up | 8.391877 | up |
| SPHK1 | 5.8519425 | up | 3.2980287 | up |
| S100A2 | 5.8748803 | up | 5.54717 | up |
| PLEKHG3 | 5.893583 | up | 3.872353 | up |
| ARG2 | 5.906965 | up | 4.5511756 | up |
| CD99 | 5.9096775 | up | 4.6138077 | up |
| TCF7 | 5.9303164 | up | 2.1097825 | up |
| FOXD1 | 5.9438562 | up | 3.845659 | up |
| CDH11 | 5.947595 | up | 4.265701 | up |
| MICA | 6.094856 | up | 3.2957346 | up |
| DSEL | 6.1983414 | up | 5.0778255 | up |
| CD82 | 6.204066 | up | 3.641176 | up |
| GPR143 | 6.2418427 | up | 17.367613 | up |
| NFASC | 6.265433 | up | 11.4692955 | up |
|  | 6.2685804 | up | 4.4485116 | up |
| INSR | 6.2892585 | up | 43.812668 | up |
| SEC14L2 | 6.303637 | up | 4.418337 | up |
| NTN1 | 6.328121 | up | 465.32315 | up |
| LOC100131541 | 6.3428526 | up | 3.73122 | up |
| SPC24 | 6.3628516 | up | 2.9107108 | down |
| SSC5D | 6.369294 | up | 29.66298 | up |
| IP6K2 | 6.380065 | up | 4.7563624 | up |
| MISP | 6.4036407 | up | 16.044643 | up |
| TMEM63C | 6.410703 | up | 9.928772 | up |
| ZNF454 | 6.4260836 | up | 4.394176 | up |
| MPZL3 | 6.4414535 | up | 19.461035 | up |
| ZNF521 | 6.5082474 | up | 84.01901 | up |
| LINC00087 | 6.549279 | up | 4.169519 | up |
| AUTS2 | 6.5632105 | up | 677.8362 | up |
| ZNF521 | 6.5922246 | up | 19.731337 | up |
| AQP1 | 6.6238084 | up | 41.458805 | up |
| CISH | 6.6266975 | up | 9.0831175 | up |
| TNS1 | 6.6298842 | up | 60.318806 | up |
| UCA1 | 6.6479025 | up | 32.304073 | up |
| SHBG | 6.656134 | up | 4.6320715 | up |
| EFR3B | 6.670789 | up | 5.4433713 | up |
| lnc-AF131215,3,1-1 | 6.7258954 | up | 2.640235 | up |
| GCNT4 | 6.7307687 | up | 5.858186 | up |
| S100A3 | 6.767165 | up | 2.6418986 | up |
| HS3ST3B1 | 6.767205 | up | 419.8721 | up |
| ASB2 | 6.8224974 | up | 2.7850258 | up |
| PMEPA1 | 6.8228154 | up | 5.8152266 | up |
| SCARNA16 | 6.8414574 | up | 9.40115 | down |
| PRDX2 | 6.8429317 | up | 7.3822002 | up |
| LRRN2 | 6.858187 | up | 4.0587044 | up |
| LOC648987 | 6.888811 | up | 2.0813072 | down |
| PCBP4 | 6.926283 | up | 2.8344738 | up |
| SLC16A8 | 6.9969816 | up | 4.8224583 | up |
| ART5 | 6.9989047 | up | 6.9769816 | up |
| PTHLH | 6.9989657 | up | 8.717588 | up |
| KALRN | 7.0453944 | up | 4.8260403 | up |
| GRASP | 7.0588317 | up | 8.89176 | up |
| PDGFB | 7.083788 | up | 13.472294 | up |
| KIAA2022 | 7.130117 | up | 4.9283643 | up |
| KIAA1549L | 7.1504364 | up | 2.567715 | up |
| RTKN2 | 7.1531606 | up | 3.8430648 | up |
| EDNRA | 7.172786 | up | 3.5625641 | up |
| RTN2 | 7.1849604 | up | 2.8315144 | up |
| POP1 | 7.240079 | up | 2.5678167 | down |
| LRRC8A | 7.252629 | up | 3.4218686 | up |
| CACNG6 | 7.2768335 | up | 46.953854 | down |
| LAMC2 | 7.279547 | up | 29.50329 | up |
| PPP1R14C | 7.2844605 | up | 12.506908 | up |
| PDLIM3 | 7.2987666 | up | 5.2835336 | up |
| LAMA4 | 7.3636966 | up | 7.277132 | down |
| REC8 | 7.3676686 | up | 35.273724 | up |
| CEP44 | 7.397968 | up | 5.7104063 | up |
| ANGPTL4 | 7.4323545 | up | 8.469397 | down |
| LOC729683 | 7.504147 | up | 4.4145684 | up |
| PMEPA1 | 7.507426 | up | 3.1081216 | up |
| PCBP4 | 7.53383 | up | 3.0420594 | up |
| LIN28B | 7.5474653 | up | 8.906838 | down |
| SPOCK2 | 7.569282 | up | 33.945206 | up |
| TBC1D19 | 7.577416 | up | 2.3272235 | up |
| ZNF667 | 7.592336 | up | 10.1207905 | up |
|  | 7.5931845 | up | 6.6592455 | up |
| CSRP1 | 7.6084046 | up | 5.7183657 | up |
| OSTF1 | 7.619447 | up | 2.554572 | up |
| ACTN1 | 7.6414223 | up | 2.9100478 | up |
| HSPA2 | 7.6837707 | up | 5.0612335 | up |
| CA3 | 7.7326226 | up | 61.56577 | down |
| RIPK3 | 7.758238 | up | 5.2961674 | up |
| RNF125 | 7.7861304 | up | 2.5846367 | up |
| TCEA3 | 7.826758 | up | 3.6212602 | up |
| C14orf37 | 7.8345637 | up | 15.2842245 | up |
| KDF1 | 7.899395 | up | 11.397538 | up |
| HOXD1 | 7.9016366 | up | 4.798506 | up |
| PIANP | 7.905065 | up | 4.757457 | up |
|  | 7.973397 | up | 2.7209778 | up |
| NCMAP | 8.001543 | up | 5.7052364 | up |
| COL5A2 | 8.002084 | up | 2.9956553 | up |
| SERPINA1 | 8.014078 | up | 4.3459563 | up |
| RNF122 | 8.026483 | up | 6.485432 | up |
| TMEM158 | 8.08551 | up | 3.1909213 | up |
| KRT14 | 8.116503 | up | 5.603545 | up |
| HEY1 | 8.128345 | up | 3.9719303 | up |
| FBLIM1 | 8.139618 | up | 51.39726 | up |
| MMP13 | 8.187754 | up | 5.7634463 | up |
| LIMA1 | 8.197415 | up | 2.772908 | up |
| PLCXD1 | 8.221141 | up | 2.596672 | up |
| M1AP | 8.320495 | up | 5.9015136 | up |
| SERINC2 | 8.336711 | up | 6.4952784 | up |
| B3GNT3 | 8.403227 | up | 14.153217 | down |
| LSR | 8.435187 | up | 8.032349 | up |
| JPH1 | 8.453844 | up | 3.593566 | down |
| GLIPR2 | 8.454258 | up | 17.54319 | up |
| MYL7 | 8.461902 | up | 4.864258 | up |
| TOX3 | 8.496836 | up | 6.503179 | up |
| CPE | 8.531974 | up | 5.2383604 | up |
| CD69 | 8.561541 | up | 5.8499866 | up |
| DSEL | 8.601591 | up | 4.3972416 | up |
| KCNK6 | 8.601723 | up | 7.1631837 | up |
| ADAM12 | 8.611002 | up | 12.710247 | up |
| GPER1 | 8.656955 | up | 2.3592896 | down |
| ST3GAL5 | 8.725065 | up | 3.85715 | up |
| SLITRK4 | 8.78304 | up | 10.09251 | up |
| TNFRSF21 | 8.812837 | up | 3.2706597 | up |
| BST2 | 8.82007 | up | 19.963318 | down |
| FAR2 | 8.829683 | up | 2.7157128 | up |
| AKAP12 | 8.887473 | up | 48.640106 | up |
| C14orf37 | 8.943627 | up | 18.127201 | up |
| HAVCR2 | 8.943878 | up | 6.4418125 | up |
| TINAG | 8.968016 | up | 3.7393012 | up |
| ZNF93 | 9.072358 | up | 3.2711046 | up |
| AKAP12 | 9.10381 | up | 37.35043 | up |
| FGFBP1 | 9.139853 | up | 110.26179 | down |
| SNAI2 | 9.144138 | up | 10.621428 | up |
| TBX3 | 9.250362 | up | 55.381977 | up |
| NREP | 9.265691 | up | 6.298595 | up |
| PTPRR | 9.291497 | up | 3.7625077 | down |
| IFFO2 | 9.298261 | up | 4.2989836 | up |
| BMP1 | 9.358431 | up | 4.5269847 | up |
| SHC4 | 9.374663 | up | 11.474473 | up |
| FBN1 | 9.426623 | up | 10.4847765 | up |
| GLIPR2 | 9.510084 | up | 11.442238 | up |
| THSD4 | 9.588088 | up | 6.649686 | up |
| HRCT1 | 9.68027 | up | 7.8042145 | down |
| TBX3 | 9.687283 | up | 13.585943 | up |
| HSD17B1 | 9.707934 | up | 5.5762815 | up |
| TPM4 | 9.7294655 | up | 3.4868464 | up |
| C2CD4B | 9.747587 | up | 6.9719367 | up |
| NNAT | 9.809561 | up | 5.968015 | up |
| FAM132B | 9.935636 | up | 34.30728 | up |
| DCLK2 | 9.951979 | up | 6.6637063 | up |
| COL3A1 | 9.999993 | up | 7.063225 | up |
|  | 10.101842 | up | 3.530656 | up |
| C19orf18 | 10.103929 | up | 7.2974033 | up |
| FAM110B | 10.114059 | up | 2.500578 | up |
| DSC2 | 10.1623535 | up | 11.668297 | up |
| FILIP1L | 10.198611 | up | 11.686987 | up |
| WNK4 | 10.252225 | up | 25.886688 | up |
| PDE1C | 10.258271 | up | 4.487513 | up |
| DACT2 | 10.334093 | up | 7.494525 | up |
| GALNT3 | 10.39269 | up | 34.426254 | up |
| XYLT1 | 10.432158 | up | 21.425402 | up |
| DSP | 10.45018 | up | 103.64646 | up |
| UCN2 | 10.493917 | up | 19.67656 | up |
| GJA1 | 10.52944 | up | 3.9303188 | up |
| EFHD1 | 10.530233 | up | 19.63051 | up |
| LAMC2 | 10.657039 | up | 21.332157 | up |
| AKAP12 | 10.670864 | up | 35.47935 | up |
| LMCD1 | 10.674363 | up | 8.252293 | up |
| PRRX1 | 10.696815 | up | 4.059406 | down |
| SDR42E1 | 10.810549 | up | 99.05444 | up |
| GAP43 | 10.819977 | up | 6.4653354 | down |
| ANKRD65 | 10.867415 | up | 7.5915446 | up |
| NRK | 10.872874 | up | 77.42688 | up |
| MMP7 | 10.956842 | up | 22.556837 | up |
| ABAT | 10.957562 | up | 442.66852 | up |
| SGK1 | 10.975938 | up | 3.7578428 | up |
| NCAM1 | 10.990428 | up | 346.8767 | up |
|  | 11.125362 | up | 9.152605 | up |
| IFITM1 | 11.161489 | up | 29.585726 | up |
| PDE1C | 11.186197 | up | 7.424542 | up |
| IFITM1 | 11.19669 | up | 29.766722 | up |
| TPM4 | 11.233032 | up | 3.5995831 | up |
| GYG2 | 11.268346 | up | 10.922617 | up |
| CPXM1 | 11.282278 | up | 10.212667 | up |
| UTY | 11.316974 | up | 7.989267 | up |
| ADCY4 | 11.488204 | up | 7.9536586 | up |
| TMSB15B | 11.516854 | up | 2.3963528 | up |
| BCL11A | 11.519622 | up | 7.4792867 | up |
| PKNOX2 | 11.57112 | up | 7.855376 | up |
| GLIPR1 | 11.648916 | up | 3.1634943 | up |
| HIVEP3 | 11.731078 | up | 4.0755844 | up |
| CGB | 11.756518 | up | 7.990685 | up |
| RNA5-8S5 | 11.903486 | up | 4.854636 | down |
| PSG10P | 11.909188 | up | 8.070838 | up |
| ANXA2R | 11.952174 | up | 5.013969 | down |
| ALDH1B1 | 12.026389 | up | 4.538961 | up |
| NT5DC2 | 12.075455 | up | 3.9322088 | up |
| LINC00900 | 12.084094 | up | 8.489972 | up |
| ARSI | 12.088943 | up | 8.48533 | up |
| LOC100129473 | 12.09607 | up | 2.9545813 | up |
| KLK6 | 12.197357 | up | 8.166711 | up |
| GPRIN2 | 12.209845 | up | 5.634359 | up |
| PTGER4 | 12.252284 | up | 2.5003114 | up |
| KLF12 | 12.392991 | up | 6.3005886 | up |
| FAM43A | 12.413718 | up | 15.229722 | down |
| PRSS1 | 12.421468 | up | 8.841317 | up |
| DSP | 12.432557 | up | 130.77077 | up |
| TIAM2 | 12.4905615 | up | 3.730526 | up |
| PRSS8 | 12.572027 | up | 56.651505 | up |
| GABRB1 | 12.642059 | up | 9.043528 | up |
| FKBP1B | 12.748929 | up | 2.2726493 | up |
| PEG3 | 12.816487 | up | 9.255297 | up |
| KANK4 | 12.943934 | up | 9.084839 | up |
| ARID3B | 12.961038 | up | 6.8395424 | up |
| ASXL3 | 13.025158 | up | 8.824086 | up |
| UTY | 13.119806 | up | 8.844495 | up |
| HOMER2 | 13.196139 | up | 19.374054 | up |
| TNFRSF11B | 13.2602215 | up | 3.955396 | up |
| PSG3 | 13.28377 | up | 9.517252 | up |
| MARCKSL1 | 13.495071 | up | 2.7135742 | up |
| NLGN4Y | 13.594249 | up | 9.827568 | up |
| LPL | 13.624017 | up | 8.997885 | up |
| BHMT2 | 13.629919 | up | 45.731857 | down |
| PELI2 | 13.768128 | up | 3.2580905 | up |
|  | 13.824711 | up | 9.518526 | up |
| SFMBT2 | 13.848661 | up | 9.696526 | up |
| ESPNL | 14.066545 | up | 10.042461 | up |
| IL4I1 | 14.29791 | up | 11.07237 | up |
| NAP1L2 | 14.402844 | up | 10.44505 | up |
| ADAM18 | 14.492985 | up | 9.912458 | up |
| RNF183 | 14.606747 | up | 10.283304 | up |
| FADS2 | 14.66791 | up | 9.45743 | up |
| PDZRN3 | 14.722113 | up | 10.371123 | up |
| SLCO2A1 | 14.756784 | up | 57.59603 | up |
| ZNF681 | 14.766514 | up | 10.058204 | up |
|  | 15.000841 | up | 4.7280717 | down |
| KCNJ12 | 15.059012 | up | 132.38864 | up |
| CABP7 | 15.154467 | up | 10.947148 | up |
| KCNJ6 | 15.203087 | up | 11.030495 | up |
| LOXL3 | 15.326272 | up | 6.551346 | up |
| GPA33 | 15.496196 | up | 11.163141 | up |
| EDNRA | 15.58192 | up | 6.9923863 | up |
| CRLF1 | 15.616175 | up | 2.6629703 | up |
| TEX12 | 15.842311 | up | 10.819038 | up |
| ESRP2 | 15.923325 | up | 13.489346 | up |
| ID1 | 16.052015 | up | 2.9397871 | down |
| LOC100128242 | 16.144747 | up | 11.311417 | up |
| A1BG | 16.190086 | up | 2.2727876 | down |
| PCDH7 | 16.22289 | up | 7.718039 | up |
| CSMD2 | 16.23314 | up | 11.717302 | up |
| LBH | 16.45038 | up | 46.1565 | up |
|  | 16.531757 | up | 11.894958 | up |
| BCL11A | 16.588211 | up | 79.01015 | up |
| CD200 | 16.637217 | up | 3.4351165 | up |
| POMC | 16.656975 | up | 12.707695 | up |
| CDA | 16.667944 | up | 4.2823863 | down |
| PPP1R1A | 16.76363 | up | 12.031524 | up |
|  | 16.802504 | up | 9.04684 | up |
| COL25A1 | 16.972782 | up | 6.3923473 | up |
| HS3ST2 | 16.983889 | up | 11.7842655 | up |
| LARGE | 17.090157 | up | 23.583193 | up |
| LOXL1 | 17.231085 | up | 48.75366 | up |
| VSNL1 | 17.236246 | up | 11.782961 | up |
| CCDC81 | 17.241636 | up | 11.67134 | up |
| PTPRK | 17.542784 | up | 3.8513515 | up |
| TNC | 17.616402 | up | 38.388477 | up |
| CECR1 | 17.627094 | up | 6.5861883 | up |
| KCNMB4 | 17.636768 | up | 2.4936163 | up |
| BGN | 17.859194 | up | 4.69816 | up |
| KIT | 17.94305 | up | 14.097315 | up |
| LY96 | 18.001543 | up | 6.464535 | down |
| DPYSL3 | 18.040009 | up | 2.652405 | up |
| IL17RD | 18.066162 | up | 10.536267 | up |
| CD274 | 18.096653 | up | 7.633376 | up |
| IFI27 | 18.18886 | up | 93.01528 | down |
| LIPG | 18.461082 | up | 27.217241 | up |
| FN1 | 18.746315 | up | 5.8231297 | up |
| LAMP5 | 18.824368 | up | 7.555713 | up |
| LOXL2 | 18.871601 | up | 2.2365863 | up |
| PADI2 | 19.077356 | up | 5.892697 | up |
| L1TD1 | 19.203737 | up | 13.493499 | up |
| THBS2 | 19.264875 | up | 16.473103 | down |
| CFTR | 19.353477 | up | 16.11975 | up |
| LOC729860 | 19.439133 | up | 13.565369 | up |
| SERPINE1 | 19.459131 | up | 5.474392 | down |
| C4orf51 | 19.512327 | up | 13.475262 | up |
| lnc-FAM133B-1 | 19.622843 | up | 11.080586 | up |
| PLEKHO1 | 19.622921 | up | 7.1735754 | up |
| HPGD | 19.685608 | up | 13.426594 | up |
| CXCL12 | 19.743992 | up | 8.016334 | up |
| SGCD | 19.839184 | up | 13.876693 | up |
| JAG1 | 20.109228 | up | 25.131384 | up |
| BACH2 | 20.155428 | up | 10.718252 | up |
| SNORA23 | 20.234097 | up | 4.384781 | down |
|  | 20.524576 | up | 5.906922 | up |
| REP15 | 20.65612 | up | 17.29232 | up |
| FAM101B | 20.820784 | up | 8.982499 | up |
| FOXS1 | 20.83907 | up | 17.856167 | up |
| ZNF69 | 21.091755 | up | 4.339909 | up |
| PLEKHO1 | 21.34836 | up | 9.539486 | up |
| RAP1GAP | 21.435602 | up | 33.59105 | up |
| NUDT10 | 21.441837 | up | 14.703545 | up |
| CYP27C1 | 21.602964 | up | 15.060839 | up |
|  | 22.213331 | up | 15.296636 | up |
| PTGS1 | 22.275583 | up | 3.698989 | down |
| TSPYL5 | 22.362585 | up | 2.7680552 | up |
| DACT1 | 22.44472 | up | 7.4392214 | up |
| STMN2 | 22.458513 | up | 15.991274 | up |
| CHST1 | 22.778196 | up | 16.315617 | up |
| ITGB3 | 22.84977 | up | 14.356399 | up |
| CPXM2 | 22.97028 | up | 15.608398 | up |
| DKK1 | 23.126623 | up | 39.250668 | down |
| ZNF439 | 23.200642 | up | 17.413874 | up |
| BMF | 23.254866 | up | 19.075384 | up |
| RPL27A | 23.603521 | up | 4.64227 | up |
| LPAR5 | 23.794338 | up | 16.80006 | up |
| BMP7 | 23.845694 | up | 13.202074 | up |
| CRMP1 | 24.07141 | up | 10.276055 | up |
| MFGE8 | 24.091402 | up | 3.4760354 | up |
| CDK15 | 24.10429 | up | 16.629013 | up |
| PSG2 | 24.289783 | up | 17.341269 | up |
| F2RL2 | 24.43335 | up | 4.2110586 | up |
| ZNF135 | 24.458776 | up | 15.371077 | up |
| MDFI | 25.024277 | up | 45.938602 | up |
| UGT1A6 | 25.227497 | up | 27.670906 | down |
| UNC13C | 25.26549 | up | 9.26097 | down |
| BMP7 | 25.478222 | up | 18.495876 | up |
| SPANXN3 | 25.572601 | up | 17.725937 | up |
| PDLIM7 | 25.718836 | up | 2.1051328 | up |
| ABCA1 | 26.19639 | up | 13.039741 | up |
| F2R | 26.307043 | up | 7.175935 | up |
| UGT1A6 | 26.440737 | up | 14.132948 | down |
| MIR100HG | 26.781822 | up | 27.538528 | down |
| LOC101929056 | 27.235672 | up | 17.274218 | up |
| C11orf96 | 27.480192 | up | 39.868904 | up |
| LPHN3 | 27.534624 | up | 26.01908 | up |
| ST3GAL1 | 28.029547 | up | 2.549523 | up |
| LINC00312 | 28.176231 | up | 11.411936 | up |
| RASIP1 | 28.244978 | up | 16.855803 | up |
| CENPVP2 | 28.416523 | up | 4.601197 | down |
| UGT1A8 | 28.48048 | up | 18.237585 | down |
|  | 28.89838 | up | 20.164316 | up |
|  | 28.983253 | up | 20.370428 | up |
| TNF | 29.414822 | up | 9.606977 | up |
| CD69 | 29.589128 | up | 21.48837 | up |
| HS6ST2 | 29.971958 | up | 278.43997 | up |
| ZNF204P | 30.292244 | up | 21.569147 | up |
| CD200 | 30.71527 | up | 3.8572474 | up |
| GABBR2 | 31.00523 | up | 37.276287 | up |
| PRSS3 | 31.38987 | up | 20.905067 | up |
| MMP9 | 31.546629 | up | 7.619261 | up |
| LOC101928880 | 31.55977 | up | 24.742462 | up |
| FLRT2 | 31.775637 | up | 4.3543873 | down |
| MYOZ3 | 32.92045 | up | 18.333431 | up |
| DCN | 33.03018 | up | 23.897823 | up |
| TDRD9 | 33.54546 | up | 11.549288 | down |
| TMEM132D | 33.926586 | up | 23.565865 | up |
| IRX1 | 33.932484 | up | 234.12361 | up |
| SFN | 34.030006 | up | 2.326151 | up |
| NEDD9 | 34.438557 | up | 123.91684 | up |
| NLGN4X | 34.807034 | up | 55.062588 | up |
| ADAM19 | 34.863346 | up | 7.3533626 | up |
| FAM50B | 35.274647 | up | 2.6430182 | down |
| UTY | 35.43921 | up | 24.609083 | up |
| LAMA4 | 36.36339 | up | 10.674629 | down |
| FAM174B | 37.235714 | up | 9.901591 | up |
| CENPV | 37.274563 | up | 6.170906 | up |
| RAMP1 | 37.44207 | up | 2.513626 | up |
| ICAM1 | 37.483227 | up | 2.7956793 | up |
| FZD8 | 37.54654 | up | 71.50736 | up |
| RASGRF2 | 37.577763 | up | 26.770847 | up |
| SLC24A3 | 37.855267 | up | 26.463879 | up |
| ITM2A | 38.006687 | up | 15.246884 | down |
| ACTA1 | 38.038757 | up | 88.86904 | up |
| WNT5A | 38.10628 | up | 75.5299 | up |
| ALDH1A2 | 38.223328 | up | 25.806856 | up |
| MTSS1 | 38.302433 | up | 5.3008933 | up |
| SFRP1 | 38.377098 | up | 6.855062 | up |
| MFAP2 | 39.52456 | up | 194.8526 | up |
| LRRN1 | 39.578262 | up | 2.8866549 | up |
| F2R | 39.771263 | up | 3.79335 | up |
|  | 40.506878 | up | 14.811604 | up |
| FZD10 | 40.665882 | up | 28.817307 | up |
| EFNB2 | 42.547462 | up | 7.247416 | up |
| ZNF606 | 43.00658 | up | 4.328725 | up |
| NR2F1 | 44.534527 | up | 4.491287 | up |
| NEFH | 44.59452 | up | 32.24359 | up |
|  | 44.892155 | up | 18.734144 | up |
| EFNB1 | 45.536415 | up | 9.68236 | up |
| CNTNAP2 | 45.61191 | up | 32.796467 | up |
| MAF | 45.752464 | up | 43.24013 | up |
| SPOCD1 | 45.80005 | up | 61.627434 | up |
| NTNG1 | 45.86496 | up | 15.920088 | up |
| ACTG2 | 46.391804 | up | 103.83196 | up |
| COL22A1 | 47.208824 | up | 34.22094 | up |
| SULT1C4 | 47.278122 | up | 33.665516 | up |
| GDNF | 47.307274 | up | 11.261334 | up |
| PRSS3P2 | 47.627766 | up | 29.59711 | up |
| SPEN | 47.776318 | up | 15.791183 | up |
| NRG3 | 47.98559 | up | 34.827606 | up |
| LOC100129397 | 48.6476 | up | 14.398135 | up |
| XDH | 48.864986 | up | 22.465107 | up |
| ZNF469 | 49.149094 | up | 23.033876 | up |
| MGC12916 | 49.210278 | up | 48.949562 | up |
| NLGN4Y | 50.03337 | up | 35.102783 | up |
| PTPLAD2 | 50.253284 | up | 4.721687 | down |
| CYP27C1 | 51.99148 | up | 35.999947 | up |
| ZNF667-AS1 | 53.033962 | up | 44.172848 | up |
| TLL1 | 53.44993 | up | 37.084473 | up |
| ANKRD1 | 53.75897 | up | 4.816152 | up |
| SCOC-AS1 | 55.94328 | up | 39.59162 | up |
| KRT34 | 56.94978 | up | 5.280153 | down |
| MRGPRF | 57.331955 | up | 5.4402347 | up |
| C4orf26 | 57.53583 | up | 8.643629 | up |
| EDN1 | 58.12707 | up | 11.643775 | up |
| F3 | 58.17074 | up | 34.30362 | up |
| MYOM2 | 58.842243 | up | 5.419232 | up |
| AFP | 59.258663 | up | 15.531939 | down |
| NOTCH3 | 59.534092 | up | 9.138975 | up |
| CTHRC1 | 60.139744 | up | 127.764275 | up |
| MGC20647 | 61.247486 | up | 5.2962174 | up |
| HBEGF | 64.87882 | up | 4.928561 | up |
| MEIOB | 65.55756 | up | 37.57011 | up |
| BMP2 | 65.96892 | up | 42.649498 | up |
| PQLC2L | 66.52395 | up | 2.365785 | down |
| DYSF | 66.72023 | up | 16.076689 | up |
| GPR87 | 66.761215 | up | 11.944174 | up |
| LTB | 67.27282 | up | 313.70618 | up |
| DIRC3 | 67.548676 | up | 46.677498 | up |
| NOX4 | 67.9212 | up | 10.252775 | up |
| MSX2P1 | 69.44816 | up | 50.028893 | up |
| ZNF876P | 69.74836 | up | 9.413562 | up |
| ANKRD35 | 70.241234 | up | 9.151552 | up |
| TMEM233 | 70.32545 | up | 49.077656 | up |
| LRRC34 | 70.41674 | up | 9.321754 | up |
| NFATC4 | 70.840195 | up | 50.2736 | up |
| RSPO4 | 71.07355 | up | 49.006413 | up |
| MAOB | 72.05074 | up | 20.704685 | up |
| FNDC1 | 73.0385 | up | 55.574585 | up |
| MME | 73.90935 | up | 9.901095 | up |
| CLIC3 | 74.71523 | up | 3.1859264 | up |
| KRBOX1 | 74.98335 | up | 52.17125 | up |
| ZNF730 | 75.2125 | up | 53.872433 | up |
| SCEL | 78.45914 | up | 53.686523 | up |
| VPS9D1-AS1 | 79.75225 | up | 4.667249 | down |
| SYK | 82.00911 | up | 30.553398 | up |
| FGF13 | 84.200294 | up | 59.82646 | up |
| DCN | 85.463295 | up | 36.46373 | up |
| SGCD | 86.3672 | up | 57.467415 | up |
| SLC2A5 | 87.31256 | up | 6.548229 | up |
| THBS2 | 88.86851 | up | 25.680382 | down |
| KCNJ15 | 89.727646 | up | 61.1489 | up |
| TIMP3 | 90.46507 | up | 73.96758 | up |
| EDN1 | 91.24608 | up | 14.816994 | up |
| LAD1 | 91.49767 | up | 94.79162 | up |
| PHACTR1 | 91.5268 | up | 4.692931 | up |
| RCAN2 | 92.26575 | up | 2.8962886 | up |
| CHST2 | 92.3112 | up | 5.2207274 | up |
| POSTN | 97.10501 | up | 67.48111 | up |
| ZFY | 97.26964 | up | 67.354454 | up |
| ID4 | 97.395546 | up | 18.451609 | up |
| TTTY14 | 97.79492 | up | 68.75981 | up |
| TMSB15A | 98.392944 | up | 9.054492 | up |
| FAM101A | 98.917885 | up | 9.228537 | down |
| COL6A3 | 100.142876 | up | 6.1189218 | up |
| CDH13 | 100.55534 | up | 12.366452 | up |
| SELENBP1 | 100.790794 | up | 2.7947292 | down |
| SEMA7A | 101.79193 | up | 15.712286 | up |
| ELTD1 | 103.45271 | up | 74.74166 | up |
| IL7R | 106.81078 | up | 6.948062 | down |
| EIF1AY | 107.98555 | up | 78.060196 | up |
| ZNF649 | 109.15837 | up | 2.2427812 | up |
| ITGA11 | 111.90539 | up | 50.830635 | up |
| NR2F1-AS1 | 113.64907 | up | 4.619185 | up |
| MUM1L1 | 116.38032 | up | 82.412506 | up |
| NIN | 118.87657 | up | 2.9244413 | up |
| STRA6 | 121.20266 | up | 11.130964 | up |
| lnc-SNX24-1 | 123.29643 | up | 23.54022 | up |
| GNAS-AS1 | 127.1275 | up | 2.580258 | down |
| ZFP42 | 128.12143 | up | 82.14589 | up |
| NRG3 | 131.40103 | up | 90.9366 | up |
| CELF2 | 141.05788 | up | 101.11675 | up |
| RBP1 | 143.20576 | up | 462.2555 | up |
| RAMP1 | 143.26295 | up | 2.7886596 | up |
| KALRN | 145.83266 | up | 10.83054 | up |
| USP9Y | 146.37286 | up | 104.09145 | up |
| COL5A1 | 152.43863 | up | 8.122619 | up |
| SSTR1 | 153.59613 | up | 3.6114888 | up |
| PPP1R14A | 161.00612 | up | 11.123182 | up |
| COL1A2 | 161.4505 | up | 105.546715 | up |
| SORCS3 | 162.14645 | up | 102.89075 | up |
|  | 164.08963 | up | 21.170265 | up |
| CD86 | 164.99081 | up | 20.170395 | up |
| COL5A1 | 174.96568 | up | 12.62181 | up |
| DIRAS2 | 178.32709 | up | 129.30058 | up |
| ACP5 | 181.03477 | up | 10.805989 | up |
| CELF2 | 181.43587 | up | 638.9432 | up |
| GAS1 | 184.00092 | up | 74.11496 | up |
| GPR179 | 185.90695 | up | 23.1623 | up |
| TRHDE-AS1 | 186.16145 | up | 8.826782 | up |
| DZIP1 | 194.88834 | up | 2.5915496 | up |
| ANKRD30BP2 | 201.14366 | up | 8.818717 | up |
| IRX4 | 205.81421 | up | 149.71037 | up |
| CLDN6 | 205.93063 | up | 140.44955 | up |
| PROM1 | 206.87164 | up | 147.77289 | up |
| EPPK1 | 206.9886 | up | 144.66664 | up |
| CAND2 | 209.88625 | up | 6.120367 | up |
| MGP | 212.20514 | up | 60.097797 | up |
| SST | 217.76277 | up | 63.497334 | up |
| ZNF542P | 219.02779 | up | 13.1506 | up |
| PDLIM3 | 220.75854 | up | 9.607471 | up |
| ZNF667-AS1 | 223.61188 | up | 158.13817 | up |
| DMRT3 | 237.76941 | up | 163.6612 | up |
| NEFM | 239.6413 | up | 436.01566 | up |
| HMBOX1 | 252.04044 | up | 26.33448 | up |
| KCNJ15 | 274.61285 | up | 188.39659 | up |
| CYTL1 | 304.39978 | up | 2.4080846 | down |
| SLC7A7 | 317.3231 | up | 8.239555 | up |
| TAGLN | 325.5919 | up | 11.634117 | up |
| GREM1 | 339.11642 | up | 33.170643 | up |
| NOX4 | 346.67224 | up | 42.554184 | up |
| PTN | 347.07166 | up | 14.076056 | up |
| MTSS1 | 348.25293 | up | 9.599177 | up |
| CLDN4 | 354.12265 | up | 18.884592 | up |
| ZNF671 | 357.13074 | up | 36.823265 | up |
|  | 358.14447 | up | 259.06287 | up |
| EMX2OS | 361.1416 | up | 351.79413 | up |
| APCDD1L | 379.23993 | up | 14.119308 | up |
| TAGLN | 382.85748 | up | 12.465818 | up |
| LRRC17 | 386.16226 | up | 1147.7485 | up |
| MMP1 | 435.6564 | up | 19.307596 | up |
| MXRA8 | 439.5212 | up | 9.241451 | up |
| GREM1 | 482.3497 | up | 340.3599 | up |
| SPON2 | 490.4718 | up | 8.343176 | up |
| GSTT1 | 518.86163 | up | 316.5134 | up |
| IGFBP2 | 518.994 | up | 537.85394 | up |
| CLEC18B | 540.80414 | up | 219.80841 | up |
| PNOC | 555.95966 | up | 370.04895 | up |
| DDX3Y | 589.87506 | up | 424.61063 | up |
| CNN1 | 590.5972 | up | 931.7241 | up |
| SRGN | 622.7358 | up | 9.850788 | down |
| PDPN | 656.44574 | up | 456.28058 | up |
| NPPB | 658.9935 | up | 229.57861 | up |
| BMP7 | 685.6174 | up | 467.11618 | up |
| NPPB | 685.7674 | up | 292.4666 | up |
| EMX2 | 718.6404 | up | 518.46497 | up |
| SPINT2 | 906.4438 | up | 5.793711 | up |
| KIF1A | 966.8521 | up | 534.06836 | up |
| COL1A1 | 1013.88055 | up | 149.64354 | up |
| CDH6 | 1073.3291 | up | 9.325485 | up |
| BTBD11 | 1204.9498 | up | 8.041948 | up |
| AEBP1 | 1218.3971 | up | 858.2026 | up |
| NDN | 1303.5846 | up | 5.0254173 | up |
| SOX11 | 1414.277 | up | 1017.19415 | up |
| IL11 | 1536.8881 | up | 63.27731 | up |
| LCP1 | 1549.8038 | up | 28.677397 | up |
| TENM2 | 2037.7706 | up | 106.304474 | up |
| CDH11 | 2039.2178 | up | 1886.6547 | up |
| ALPK3 | 2217.4985 | up | 228.03294 | up |
| IGFBP5 | 4112.031 | up | 1296.1914 | up |
| RPS4Y2 | 9231.681 | up | 5582.307 | up |
| MYADM | 9740.568 | up | 3.7797806 | up |
| RPS4Y1 | 10168.842 | up | 7098.2056 | up |
